# Autophagy and intratumoral bacteria abundance influence the prognosis of patients with pancreatic carcinoma

**DOI:** 10.1038/s41419-026-09256-1

**Published:** 2026-10-27

**Authors:** Yifan Zhang, Toohina L. Gobin, Zhenhua Huang, Bingwen Miao, Roma Kurilov, Haitao Sun, Christine Tjaden, Ulrike Heger, Thilo Hackert, Ulf Hinz, Michael Schaefer, Frank Bergmann, Christoph Springfeld, Sadaf S. Mughal, Benedikt Brors, Patrick Schaal, Bachar Cheaib, Danilo Licastro, Valerio Iebba, Franco Fortunato

**Affiliations:** 1 Department of General, Visceral and Transplantation Surgery, Heidelberg, Germany; 2 Translational Research Center, University Clinic Heidelberg, Heidelberg, Germany; 3 Division of Applied Bioinformatics, The German Cancer Research Center (DKFZ), Heidelberg, Germany; 4 Technical University Munich, Clinic for Surgery, Munich, Germany; 5 Department of General, Visceral and Thoracic Surgery, University Hospital Hamburg-Eppendorf, Hamburg, Germany; 6 Institute of Pathology, University Clinic Heidelberg, Heidelberg, Germany; 7 Department of Medical Oncology, National Center for Tumor Diseases, University Clinic Heidelberg, Heidelberg, Germany; 8 Department of Infectious Diseases, Medical Microbiology and Hygiene, University Clinic Heidelberg, Heidelberg, Germany; 9 Translational Lung Research Center (TLRC) Heidelberg, German Center for Lung Research(DZL), University Clinic Heidelberg, Heidelberg, Germany; 10 AREA Science Park, 34149 Trieste, Italy; 11 Gustave Roussy Cancer Campus, ClinicObiome, Villejuif, France; 12 Department of Medical, Surgical, and Health Sciences, University of Trieste, Trieste, Italy

**Keywords:** Pancreatic cancer, Macroautophagy

## Abstract

The biological and clinical significance of the intratumoral microbiota in pancreatic ductal adenocarcinoma (PDAC) remains poorly defined. Using oncogenic Kras-driven mouse models of pancreatic tumorigenesis and human pancreatic tissue from patients with non-metastatic PDAC treated with neoadjuvant (*n* = 62) or adjuvant therapy (*n* = 101), along with samples from intraductal papillary mucinous neoplasms (IPMN, *n* = 24), chronic pancreatitis (CP, *n* = 33), and healthy donors (*n* = 9), we determined tissue lipopolysaccharide (LPS), 16S rRNA sequencing, high-resolution quantitative multiplex immunofluorescence of autophagy markers (LAMP2, LC3B) and LPS, 16S rRNA fluorescence in situ hybridization (FISH) staining, and TLR4 signaling. In KrasCre mice, we identified significant bacterial accumulation in the pancreas, indicated by elevated LPS, increased FISH signals, enhanced TLR4 signaling, and activation of autophagy. Administration of exogenous LPS further amplified autophagy and TLR4 signaling, supporting a mechanistic link between microbial sensing and autophagy. In human tissue, both adjuvant and neoadjuvant PDAC samples showed significantly increased bacterial alpha- and beta diversity compared with controls, while CP showed a similar enrichment pattern that IPMN lacks; the underlying microbial diversity differs fundamentally across these conditions. Increased LPS levels, along with elevated autophagy markers and abundant FISH-positive bacteria, support the presence of intratumoral bacteria. Across four independent transcriptomic datasets, PDAC samples showed elevated expression of bacteria-associated pathways (including TLR4) and autophagy-related genes. Clinically, higher autophagy-associated LAMP2-LC3B colocalization and increased densities of FISH-positive cells were independently associated with prolonged survival in both adjuvant and neoadjuvant or combined PDAC patients. Collectively, these findings link intratumoral microbial enrichment to increased autophagy-associated signaling and improved patient outcomes, suggesting that microbial signals within the tumor microenvironment were associated with favorable survival.

## Introduction

Pancreatic adenocarcinoma (PDAC) is a highly aggressive cancer with an overall 5-year survival rate of approximately 13%, and median survival remains low at approximately 4 months across all tumor stages [1, 2]. Despite advances in surgery and systemic therapy, PDAC remains the fourth leading cause of cancer-related death and is projected to become the second leading cause of cancer-associated mortality in developed countries within the next decade [1]. Thus, there is an urgent need to identify novel biomarkers and therapeutic targets to improve patient outcomes. In recent years, there has been growing interest in the role of the microbiota, as dysbiosis has been implicated in cancer initiation and progression, including PDAC [3]. The pancreas, once regarded as a sterile organ, is now recognized as a microbiologically active environment, capable of modulating disease pathogenesis and severity [4]. Bacterial colonization of the pancreas is thought to occur via retrograde translocation from the duodenum through the pancreatic duct [5, 6].

Microbes can penetrate pancreatic epithelial cells through well-established invasion pathways, leading to intracellular localization [7]. An evolutionarily conserved antibacterial autophagy pathway-xenophagy-eliminates intracellular pathogens by directing them to lysosomal degradation [8]. Beyond maintaining cellular homeostasis, xenophagy is a key host defense mechanism, limiting microbial persistence and infection [9]. Accordingly, xenophagy represents a critical component of the innate immune response, while many pathogens have evolved sophisticated strategies to subvert or inhibit xenophagy, thereby promoting intracellular persistence and immune evasion [10–12]. Chronic microbial persistence may therefore drive sustained inflammation and oncogenic signaling, increasing susceptibility to malignant transformation, as exemplified by *Helicobacter pylori* [13].

The intratumoral microbiota of PDAC is distinct from that of the healthy pancreas and varies across disease stage and metastatic progression [3, 14, 15]. Recent experimental evidence demonstrates that microbial components such as LPS remodel the tumor microenvironment and synergize with PD-L1 blockade to suppress tumor growth [16]. Notably, increased microbial diversity has been associated with improved survival, suggesting that enrichment of intratumoral microbiota correlates with higher inflammatory responses and better survival. In addition, fecal microbiota transplantation experiments demonstrate that microbiota derived from long-term survivors can change the tumoral microbiota, leading to reduced tumor growth, enhanced immune cell infiltration, and reduced PDAC tumorigenesis [17]. Of note, emerging clinical studies suggest that probiotic supplementation may modestly extend survival in PDAC patients [18].

Autophagy plays a complex role in PDAC tumorigenesis. Genetic inhibition of autophagy leads to pancreatitis and pre-malignant lesions in mouse models, indicating a tumor-suppressive function in early disease [19–21]. Deletion of autophagy genes in hepatocytes promotes liver tumors, suggesting that autophagy loss promotes tumorigenesis [22]. Inhibition of autophagy exacerbates LPS-induced cell death by apoptosis and necroptosis, NFκB, and pro-inflammatory cytokines, thereby increasing local pancreatic tissue damage [20].

Beyond antimicrobial defense, xenophagy also shapes host immunity by modulating microbial antigen presentation and constraining excessive inflammatory cascades [23]. However, although autophagy is elevated in PDAC, several clinical trials targeting autophagy (e.g. hydroxychloroquine, HCQ) have shown limited efficacy and do not improve survival [4, 24–30]. Importantly, the relationship between autophagy/xenophagy, the intratumoral microbiota, and clinical outcome in PDAC remains unclear.

In this study, we integrated 16S rRNA (16S ribosomal RNA) sequencing, a FISH assay, and endotoxin (LPS) measurements to quantify intratumoral microbiota. To assess the link between bacterial burden and autophagy, we analyzed LAMP2_LC3B colocalization as a surrogate marker of autophagy [31, 32]. We show that autophagy correlates with the regulation of intratumoral microbial burden, and this is associated with improved patient survival, revealing a functional microbiota-autophagy axis in PDAC.

## Results

### Accumulation of pancreatic bacteria in KrasCre mice

**Fig. 1:**
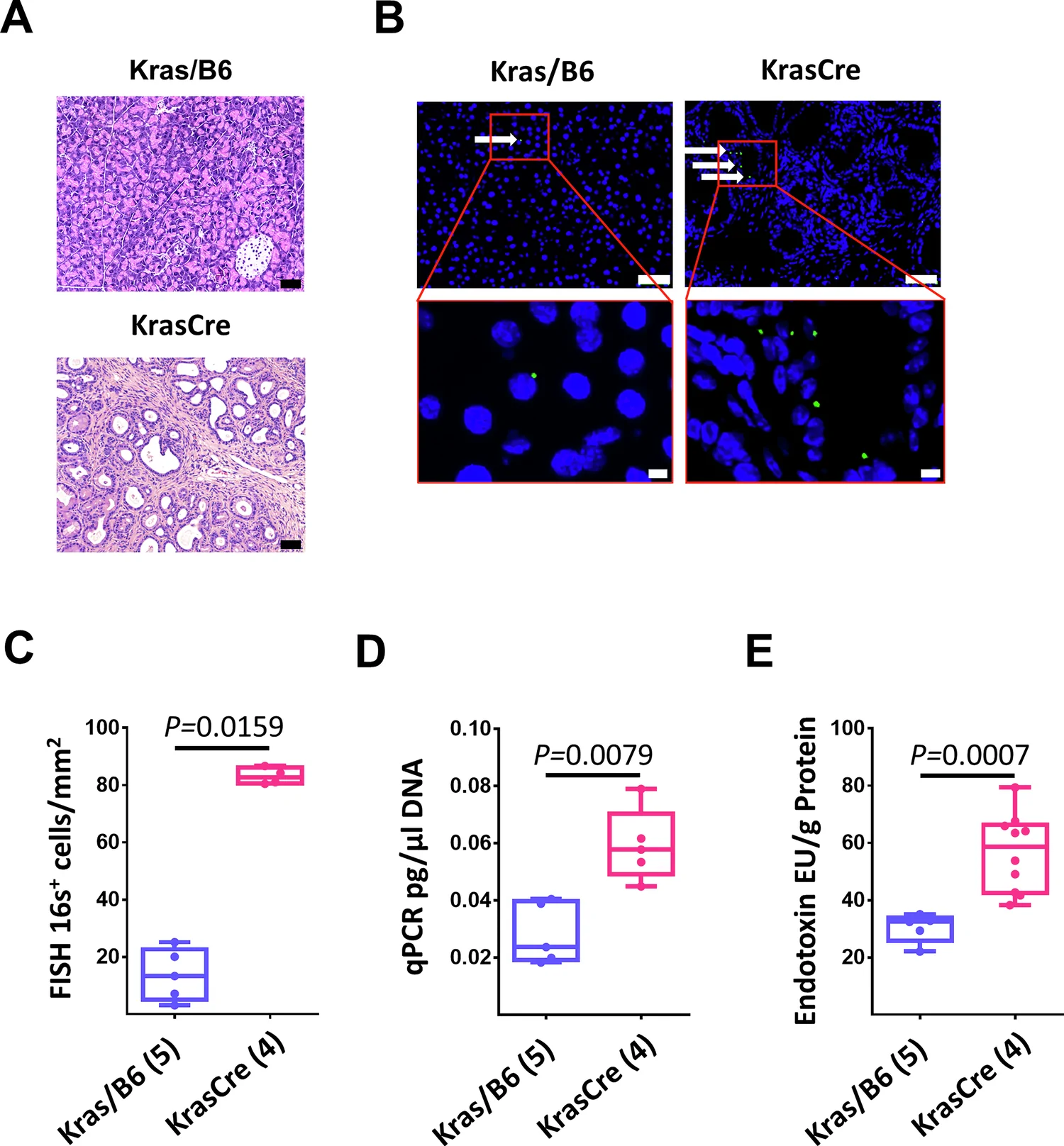
Increased bacterial colonization in mouse models of pancreatic preneoplastic lesions. **A** Representative H&E-stained pancreatic mouse tissue sections from KrasCre (PDAC precursor) mice and Kras/B6 control mice. Pancreatic disease exhibiting pancreatitis features with fibrosis, ADMs, PanIN lesions, and pancreatic tumor precursor lesions. H&E staining images; scale bar = 50 µm. **B** Representative fluorescence in situ hybridization (FISH) detecting bacterial 16S rRNA (green) in pancreatic tissue from KrasCre and Kras/B6 mice. Nuclei counterstained with 4′,6-diamidino-2-phenylindole (DAPI) (blue). Scale bar = 50 µm (overview) and 5 µm (magnified). **C** Box-and-whisker plots with all points/animals shown. Quantitation of 16S rRNA FISH-positive cells per mm^2^ pancreatic tissue in KrasCre and Kras/B6 mice. Differences between groups were assessed via the Mann-Whitney *U* test. Significance levels (*P* values) are displayed above the respective bars/plots. **D** Box-and-whisker plots with all points/animals shown. Quantitation of pancreatic bacterial DNA by qPCR as relative 16S rRNA signals per µl of tissue DNA in KrasCre and Kras/B6 mice. Differences between groups were assessed via the Mann-Whitney *U* test. Significance levels (*P* values) are displayed above the respective bars/plots. **E** Box-and-whisker plots with all points/animals shown. Endotoxin concentration in pancreatic extracts expressed as Endotoxin Units (EU) per g tissue from KrasCre and Kras/B6 mice. Differences between groups were assessed via the Mann-Whitney *U* test. Significance levels (*P* values) are displayed above the respective bars/plots for 4 to 5 mice per group.

We analysed a Kras-driven PDAC mouse model (KrasCre) alongside Kras/B6 controls without LPS treatment. H&E staining revealed morphological alterations and tumor precursor lesions, including acinar-to-ductal metaplasia (ADM) and pancreatic intraepithelial neoplasia (PanIN) [33] (Fig. 1A). At older ages, KrasCre mice frequently develop the full spectrum of PanIN to invasive PDAC [34]. Fluorescent in situ hybridization (FISH) detection of 16S rRNA (Fig. 1B) demonstrated a marked increase in bacterial abundance in KC pancreata (~6-fold) compared with control littermates (Fig. 1C). PCR confirmed an elevated bacterial load, with a 2.4-fold increase in rRNA in KC compared with controls [14]. (Fig. 1D). Consistently, a tissue endotoxin (LPS) enzymatic assay showed a 1.8-fold increase in KrasCre mice compared with controls (Fig. 1E). These findings demonstrate that bacterial accumulation is an early feature of pancreatic tumorigenesis.

### Elevated endotoxin activates pancreatic TLR4 signaling

**Fig. 2:**
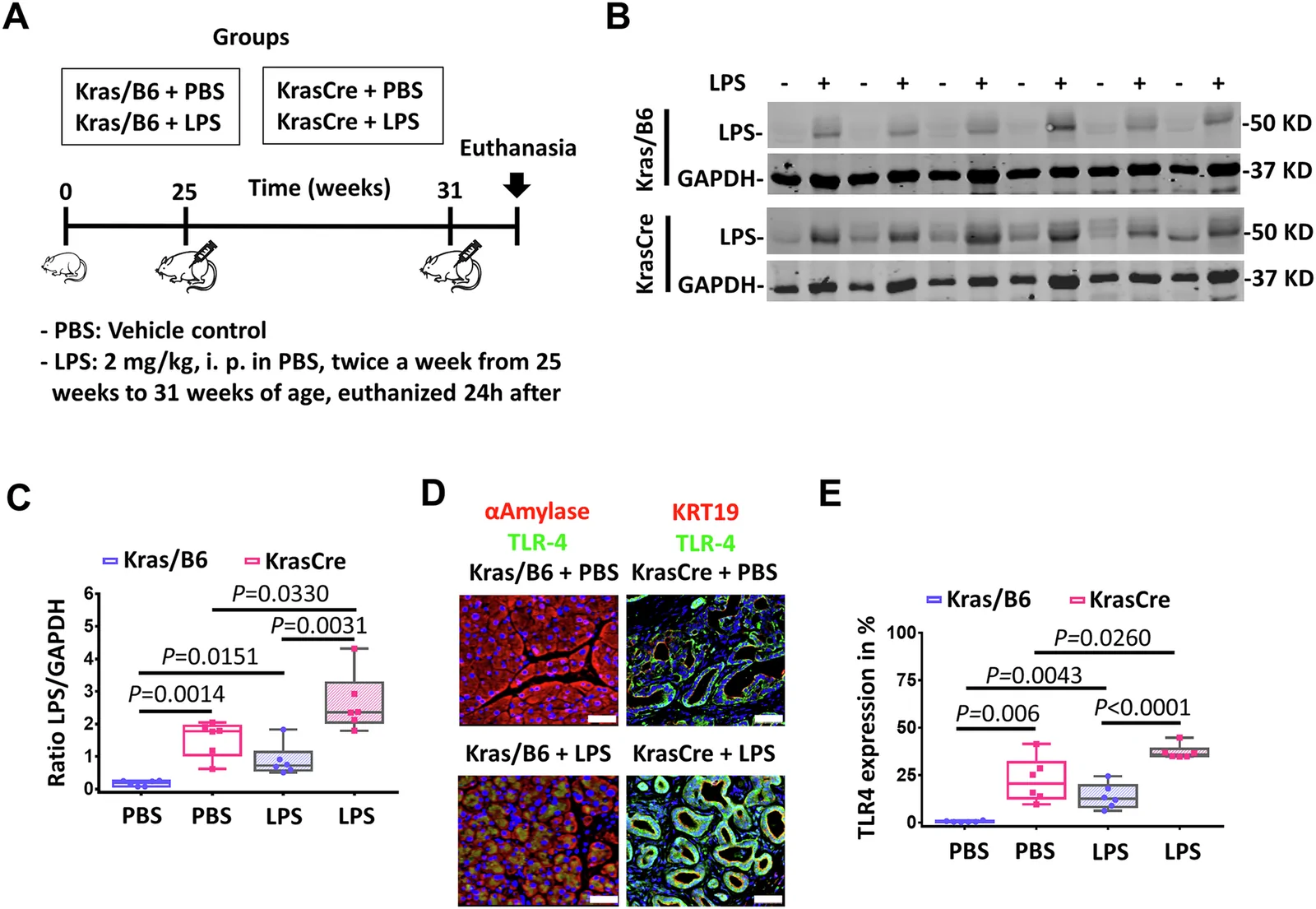
LPS enhances pancreatic TLR4 signaling. **A** Schematic illustration of the experimental procedure for performing LPS injection (timeline). **B** Representative immunoblot images showing LPS and GAPDH (loading control) in KrasCre and Kras/B6 mice pancreata ± LPS treatment. **C** Box-and-whisker plots with all points/animals shown. Densitometric quantification of LPS normalized to GAPDH (LPS/GAPDH ratio) in KrasCre and Kras/B6 mice with or without LPS treatment. Differences between groups were assessed via the Mann-Whitney U test. Significance levels (*P* values) are displayed above the respective bars/plots. **D** Representative immunofluorescence (IF) images showing stained TLR4 co-stained with α-Amylase (Kras/B6 controls) or KRT19 (KrasCre). Scale bar, 50 µm. **E** Box-and-whisker plots with all points/animals shown. Quantitation of TLR4 IF signal in KrasCre and Kras/B6 control mice. Differences between groups were assessed via the Mann-Whitney *U* test. Significance levels (*P* values) are displayed above the respective bars/plots for 4 to 5 mice per group.

To assess the functional relevance, we quantified intrapancreatic LPS levels in KrasCre and Kras/B6 mice with or without LPS treatment (Fig. 2A). Intraperitoneal (i.p.) LPS administration increased pancreatic LPS levels, with a stronger effect in KrasCre mice. Basal LPS levels without exogenous LPS treatment were ~9-fold higher in KrasCre mice compared with Kras/B6 controls (Fig. 2B, C). Moreover, expression of the LPS receptor Toll-like-receptor 4 (TLR4) was readily detectable and markedly elevated in KrasCre pancreata (Fig. 2D, E). Notably, TLR4 expression increased ~42-fold in KrasCre mice under basal conditions compared with controls. These data indicate that microbial accumulation activates the LPS-TLR4 signaling axis in tumor-prone pancreas, even in the absence of exogenous stimulation. Original BW images are provided in the supplemental material.

### Bacterial endotoxin enhances pancreatic autophagy

We assessed autophagy-related proteins in KrasCre and control mice ± LPS treatment (Supplementary Fig. 1A). KrasCre mice exhibited increased expression of ATG5 and BECLIN1, alongside reduced p62 levels, consistent with enhanced autophagy activation. LPS treatment further amplified autophagy marker expression, while promoting LC3-I to LC3-II conversion [35]. Basal microbial signals in precancerous KrasCre mice were sufficient to modestly activate autophagy, whereas LPS stimulation robustly enhanced this response (Supplementary Fig. 1B-F). These results collectively demonstrate that microbial products such as LPS and bacterial 16S rRNA, reflecting the accumulation of intrapancreatic microbes, drive autophagy activation in pancreatic tissue.

### Microbiota accumulation in patients with chronic pancreatitis and intraductal papillary mucinous neoplasm

**Fig. 3:**
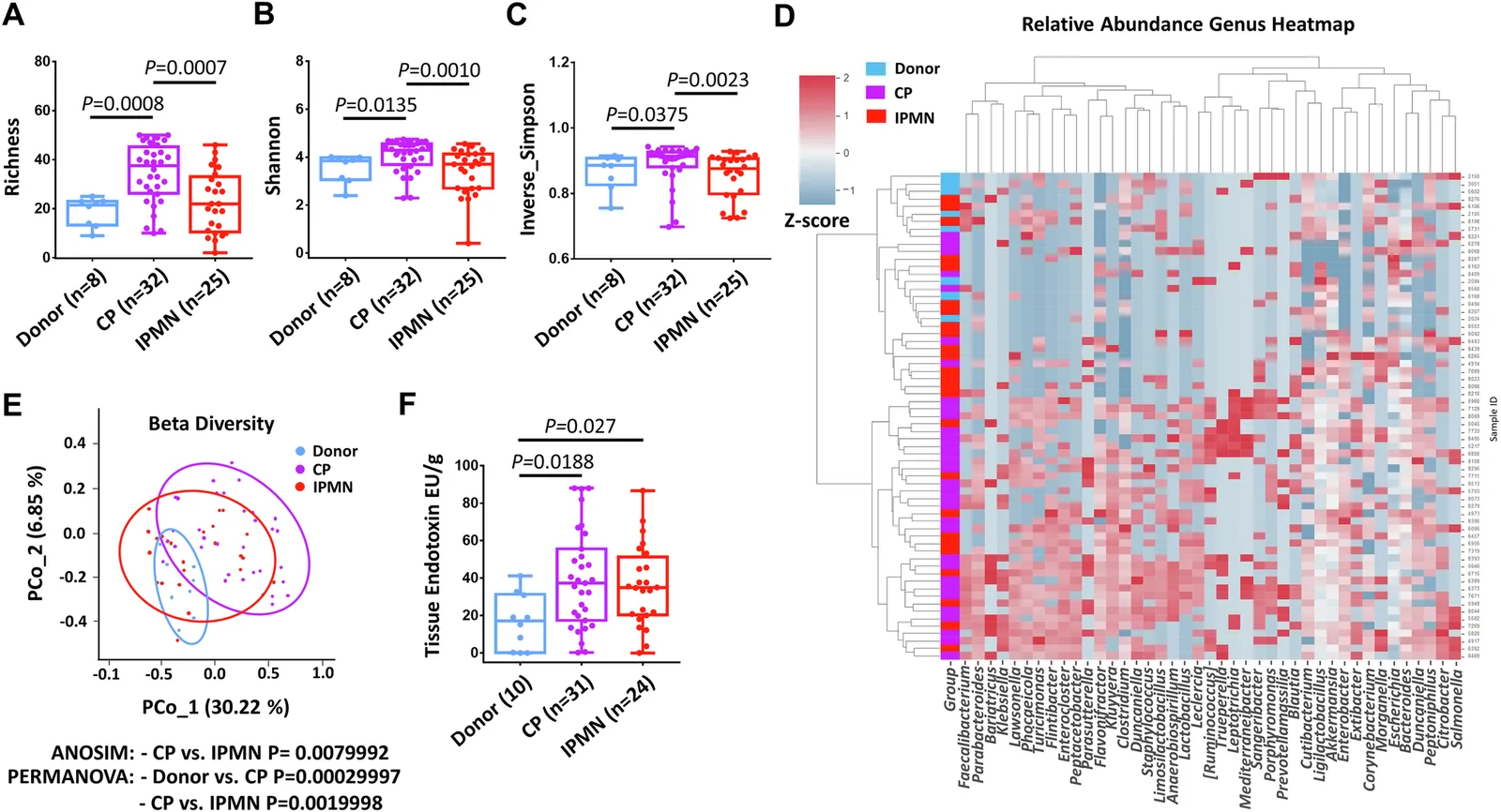
Distinct tissue microbiota profiles in chronic pancreatitis (CP) and intraductal papillary mucinous neoplasms (IPMN). **A** Box-and-whisker plots with all patients/points shown. Richness alpha diversity index values in donor, CP, and IPMN tissue. Differences between groups were assessed via the Mann-Whitney *U* test. Significance levels (*P* values) are displayed above the respective bars/plots. **B** Box-and-whisker plots with all patients/points shown. Shannon alpha diversity index values in donor, CP, and IPMN tissue. Differences between groups were assessed via the Mann-Whitney *U* test. Significance levels (*P* values) are displayed above the respective bars/plots. **C** Box-and-whisker plots with all patients/points shown. Inverse Simpson alpha diversity index values in donor, CP, and IPMN tissue. Differences between groups were assessed via the Mann-Whitney *U* test. Significance levels (*P* values) are displayed above the respective bars/plots. **D** Heatmap/Clustermap of *Z*-scored relative abundances of microbial genera showing distinct community patterns, with donor controls and IPMN co-clustering in cluster 1 and CP samples predominantly in cluster 2 (Freeman-Halton extension of Fisher´s exact test, two-tailed *P* = 3.28*10^-5^). **E** Principal coordinate analysis (PCoA) of beta diversity showed between-group differences in community composition among CP, IPMN, and donor controls. Significance was assessed using ANOSIM and PERMANOVA (999 permutations) and using Kruskal-Wallis and pairwise Mann-Whitney tests with *P* values. **F** Box-and-whisker plots with all patients/points shown. Endotoxin levels were measured by enzymatic assay in pancreatic tissue homogenate from donor controls, CP, and IPMN patients. Differences between groups were assessed via the Mann-Whitney *U* test. Significance levels (*P* values) are displayed above the respective bars/plots.

We profiled intrapancreatic microbiota in chronic pancreatitis (CP) (Table 1A) and intraductal papillary mucinous neoplasm (IPMN) patients (Table 1B), two conditions that increase the risk for developing PDAC. 16S sequencing revealed increased microbial alpha diversity in CP but not in IPMN compared with donor controls, as measured by Richness, Shannon, and Inverse Simpson indices (Fig. 3A-C). IPMN exhibited lower diversity than CP, consistent with a prior study [36]. However, at the genus level, the heatmap did not clearly visualize differences among the three groups (Fig. 3D). In contrast, beta diversity analysis showed distinct microbial communities in CP compared with controls and IPMN as determined by the ANOSIM and PERMANOVA indices (Fig. 3E). Endotoxin measurements revealed a ~2-fold increase of Gram-negative LPS (enzyme assay) in pancreatic tissue extracts from CP and IPMN patients compared with donor controls (Fig. 3F). These findings indicate that microbial accumulation and composition shifts occur early during pancreatic disease progression. Original BW images are provided in the supplemental material.

**Table 1:** Demographics, clinical, anamnestic, and clinical-pathological information features.

A		
Clinical parameter	Category / measuring unit	CP
**Total number**	N	33 (100.0%)
**Gender**	Male	20 (60.6%)
	Female	13 (39.4%)
**Age at operation**	Median (range) in years	56 (16–80)
**BMI**	Median (iqr) in Kg/m2	23.5 (20.6–26.6)
**ASA classification**	ASA 1	3 (9.1%)
	ASA 2	16 (48.5%)
	ASA 3	12 (36.4%)
	ASA X	2 (6.1%)
**CA 19-9 preoperative**	Median (iqr) in U/mL	18.7 (7.3–43.6)
**Diabetes mellitus**	no DM	20 (60.6%)
	NIDDM	3 (9.1%)
	IDDM	2 (24.2%)
	Missing data	2 (6.1%)
**Pain intensity**	None	2 (6.1%)
	Light	1 (3.0%)
	Medium	7 (21.2%)
	Heavy	18 (54.6%)
	Missing data	5 (15.2%)
**Pain frequency**	Never	2 (6.1%)
	Monthly	7 (21.2%)
	Weekly	1 (3.0%)
	Daily	15 (45.5%)
	Missing data	8 (24.2%)
**Episodes of acute pancreatitis**	0	1 (3.7%)
	1	9 33.3%)
	2 / 3	5 (18.5%)
	4 / 5	6 (22.2%)
	>5	6 (22.2%)
	Missing data	6
**Type of operation**	Pancreatoduodenectomy	21 (63.6%)
	Total pancreatectomy	5 (15.2%)
	Distal pancreatectomy	6 (18.2%)
	DPRHP	1 (3.0%)
**Experimental Procedure**		
Immunofluorescence (FFPE)		6 (18.1%)
Endotoxin Assay (cryo)		31 (93.9%)
Microbiome Analysis (cryo)		30 (90.9%)

B		
Clinical parameter	Category / measuring unit	IPMN
**Total number**	N	24 (100.0%)
**Gender**	Male	12 (50.0%)
	Female	12 (50.0%)
**Age at operation**	Median (range) in years	62 (31–83)
**BMI**	Median (iqr) in Kg/m2	23.6 (22.5–28.8)
**ASA classification**	ASA 1	2 (8.3%)
	ASA 2	13 (54.2%)
	ASA 3	7 (29.2%)
	ASA 4	1 (4.2%)
	ASA X	1 (4.2%)
**CA 19-9 preoperative**	Median (iqr) in U/mL	9.4 (7.8–13.8)
**Type of IPMN**	Main duct	2 (8.7%)
	Mixed type	14 (60.9%)
	Branch duct	7 (30.4%)
	Missing data	1
**Degree of dysplasia**	Adenoma	14 (58.3%)
	Borderline	1 (4.2%)
	Carcinoma in situ	8 (33.3%)
	Invasive carcinoma	1 (4.2%)
**Localization of lesion**	Pancreatic head	9 (37.5%)
	Pancreatic body	0 (0.0%)
	Pancreatic tail	7 (29.2%)
	Multifocal disease	8 (33.3%)
**Type of operation**	Pancreatoduodenectomy	8 (33.3%)
	Total pancreatectomy	8 (33.3%)
	Distal pancreatectomy	7 (29.2%)
	Enucleation	1 (4.2%)
**Vessel resection**	N	0 (0.0%)
**Experimental Procedure**		
Immunofluorescence (FFPE)		4 (16.6%)
Endotoxin Assay (cryo)		24 (100%)
Microbiome Analysis (cryo)		21 (87.4%)

C		
Neoadjuvant set	62 (100.0%)	0 (0.0%)
**Pre-operative chemotherapy**		
FOLFIRINOX	39 (62.9%)	
FOLFIRINOX + other	2 (3.2%)	
Gemcitabine/NAB-Paclitaxel	7 (11.3%)	
Gemcitabine mono	7 (11.3%)	
Gemcitabine combination	6 (9.7)	
Missing data	1 (1.6%)	
**Experimental Procedure**		
Immunofluorescence/FISH (FFPE)	48 (77.4%)	64 (63.3%)
Endotoxin Assay (cryo)	15 (24.1%)	35 (34.6%)
Microbiome Analysis (cryo)	13 (20.9%)	26 (25.7%)

(A) Clinical data of 33 patients diagnosed with chronic pancreatitis (CP). Tissue samples were used for analyzing endotoxin assay (cryo) and 16S sequencing (cryo). (B) Clinical data of 24 patients diagnosed with intraductal papillary mucinous neoplasms (IPMN). Tissue samples were used for analyzing endotoxin assay (cryo) and 16S sequencing (cryo). (C) Clinical data of 62 patients with neoadjuvant chemotherapy and 101 chemo-naive, adjuvant patients diagnosed with pancreatic ductal adenocarcinoma (PDAC). Tissue samples were used for analyzing IF/FISH (FFPE) and endotoxin assay (cryo) as well as 16S sequencing (cryo).

### Intratumoral microbiota are enriched in pancreatic adenocarcinoma

**Fig. 4:**
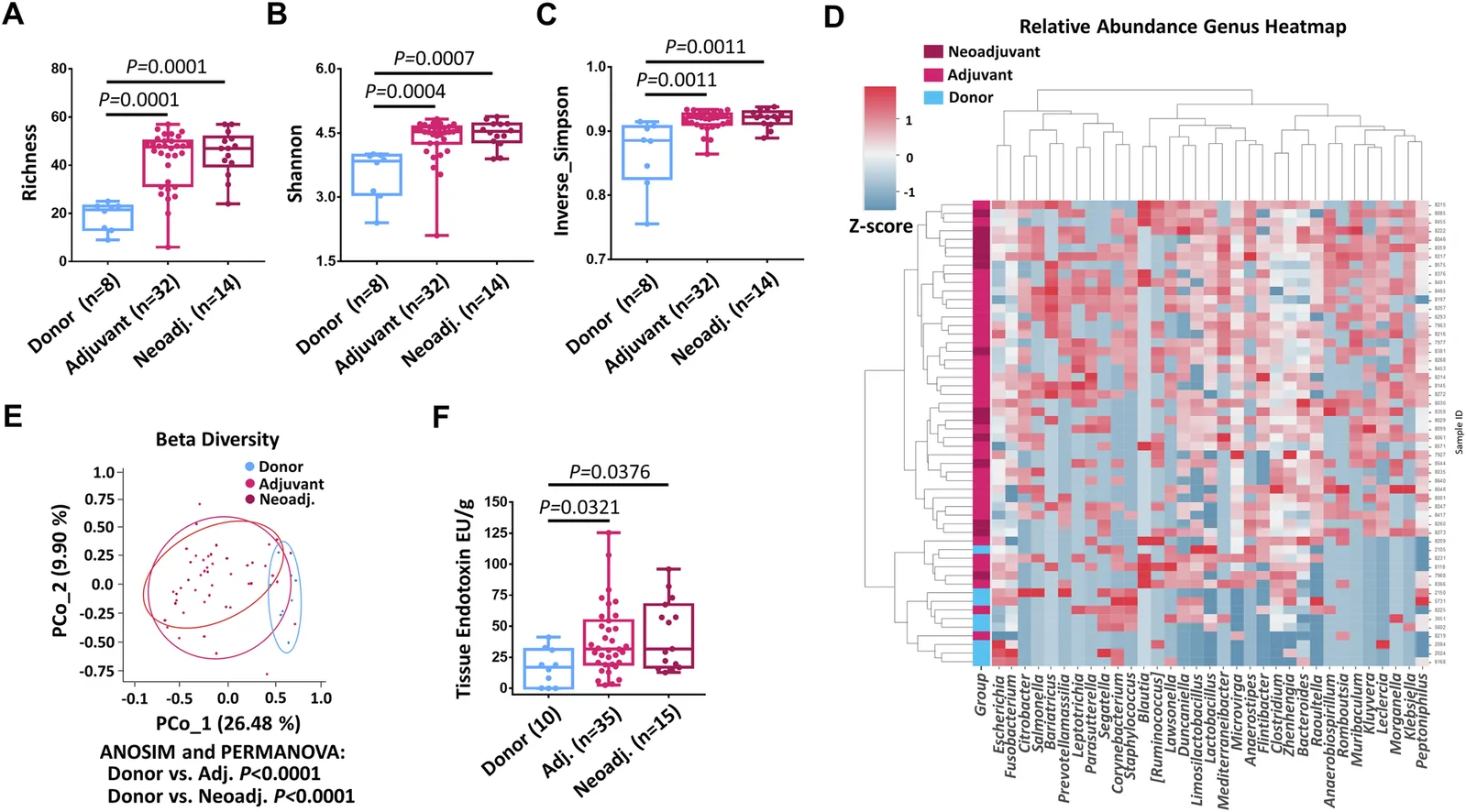
Distinct microbiota profiles in adjuvant (chemo-naive) and neoadjuvant pancreatic ductal adenocarcinoma (PDAC) patients. **A** Box-and-whisker plots with all patients/points shown. Richness alpha diversity index values in donor controls, adjuvant (chemo-naive), and neoadjuvant PDAC tissue. Differences between groups were assessed via the Mann-Whitney *U* test. Significance levels (*P* values) are displayed above the respective bars/plots. **B** Box-and-whisker plots with all patients/points shown. Shannon alpha diversity index values in donor controls, adjuvant (chemo-naive), and neoadjuvant PDAC tissue. Differences between groups were assessed via the Mann-Whitney *U* test. Significance levels (*P* values) are displayed above the respective bars/plots. **C** Box-and-whisker plots with all patients/points shown. Inverse Simpson alpha diversity index values in donor controls, adjuvant (chemo-naive), and neoadjuvant PDAC tissue. Differences between groups were assessed via the Mann-Whitney *U* test. Significance levels (*P* values) are displayed above the respective bars/plots. **D** Heatmap/Clustermap of *Z*-scored relative abundances of microbial genera showing co-clustering of adjuvant (chemo-naive) and neoadjuvant PDAC samples in cluster 1 (Freeman-Halton extension of Fisher´s exact test, two-tailed *P* = 1.04*10^−5^). **E** Principal coordinate analysis (PCoA) of beta diversity showing between-group differences in community composition among donor controls, adjuvant (chemo-naive) and neoadjuvant PDAC patients. Significance was assessed using ANOSIM and PERMANOVA (1000 permutations) and using Kruskal-Wallis and pairwise Mann-Whitney tests with *P* values. **F** Box-and-whisker plots with all patients/points shown. Endotoxin levels were measured by enzymatic assay in pancreatic tissue homogenate from donor controls, adjuvant (chemo-naive), and neoadjuvant PDAC patients. Differences between groups were assessed via the Mann-Whitney *U* test. Significance levels (*P* values) are displayed above the respective bars/plots.

We next analyzed intratumoral microbiota in tissue specimens from patients with adjuvant and neoadjuvant PDAC (Table 1C). This approach revealed significantly elevated microbiota alpha diversity in adjuvant and neoadjuvant PDAC samples compared with donors, as indicated by the Richness, Shannon, and Inverse Simpson indices (Fig. 4A-C). At the genus level, the heat map indicates an increase in the relative abundances of many bacteria in both adjuvant and neoadjuvant tissue compared with donor controls (Fig. 4D). Beta diversity, measured as the ANOSIM index, revealed significantly different microbiota species in adjuvant PDAC compared with donor controls, while the PERMANOVA index identified differences between neoadjuvant PDAC compared with donor controls (Fig. 4E). The accumulation of intratumoral bacteria was confirmed by an enzymatic Gram-negative LPS assay using tissue extract. LPS increased 2.3-fold in adjuvant and 2.6-fold in neoadjuvant PDAC tissues compared with donor pancreata (Fig. 4F). We measured similar LPS concentrations of approximately 40 EU (endotoxin units) per g tissue in murine *KrasCre* pancreata and human pancreata affected by pathologies (CP, IPMN, PDAC) (Fig. 3F, 4F). Additional microbial sensing pathways (TLR4, CD14, LBP, MyD88, MD2 mRNA expression) were almost all upregulated in PDAC transcriptomic datasets (Supplementary Fig. 2A). All five mRNA species directly involved in LPS signaling - with the exception of LBP - were significantly elevated in the volcano plot (Supplementary Fig. 2B) and markedly increased in PDAC tissue within the GSE15471 database (Supplementary Fig. 2C). This finding suggests that PDAC tissue shows enriched microbiota with elevated TLR4 signaling.

### Different microbiota species in PDAC, CP, and IPMN

We further compared the intrapancreatic alpha diversity of the microbiota in tissue specimens from patients with CP, IPMN, and both PDAC groups combined (Table 1A-C). We detected significantly elevated microbiota alpha diversity in PDAC samples compared with CP and IPMN, indicated by the Richness, Shannon, and Inverse Simpson indices (Supplementary Fig. 4A-C). At the genus level, the heat map indicates no visual differences within CP, IPMN and combined both PDAC groups (Supplementary Fig. 4D). Beta diversity measured as the ANOSIM and PERMANOVA index showed a significantly different microbiota species in PDAC compared with CP and IPMN with more homogeneous microbiota species in PDAC and more diverse microbiota species in CP and IPMN (Supplementary Fig. 4E). Moreover, a volcano plot with abundance-scaled species enrichment revealed higher accumulation of *Microvirga* and *Porphyromonas* mainly in PDAC tissue and *Escherichia* and *Ligilactobacillus* mainly in CP/IPMN tissue, suggesting that CP and IPMN with a high risk of developing PDAC show different intra-pancreatic bacterial species compared with PDAC (Supplementary Fig. 4F).

### Bacterial enrichment correlates with improved survival in adjuvant and neoadjuvant PDAC patients

**Fig. 5:**
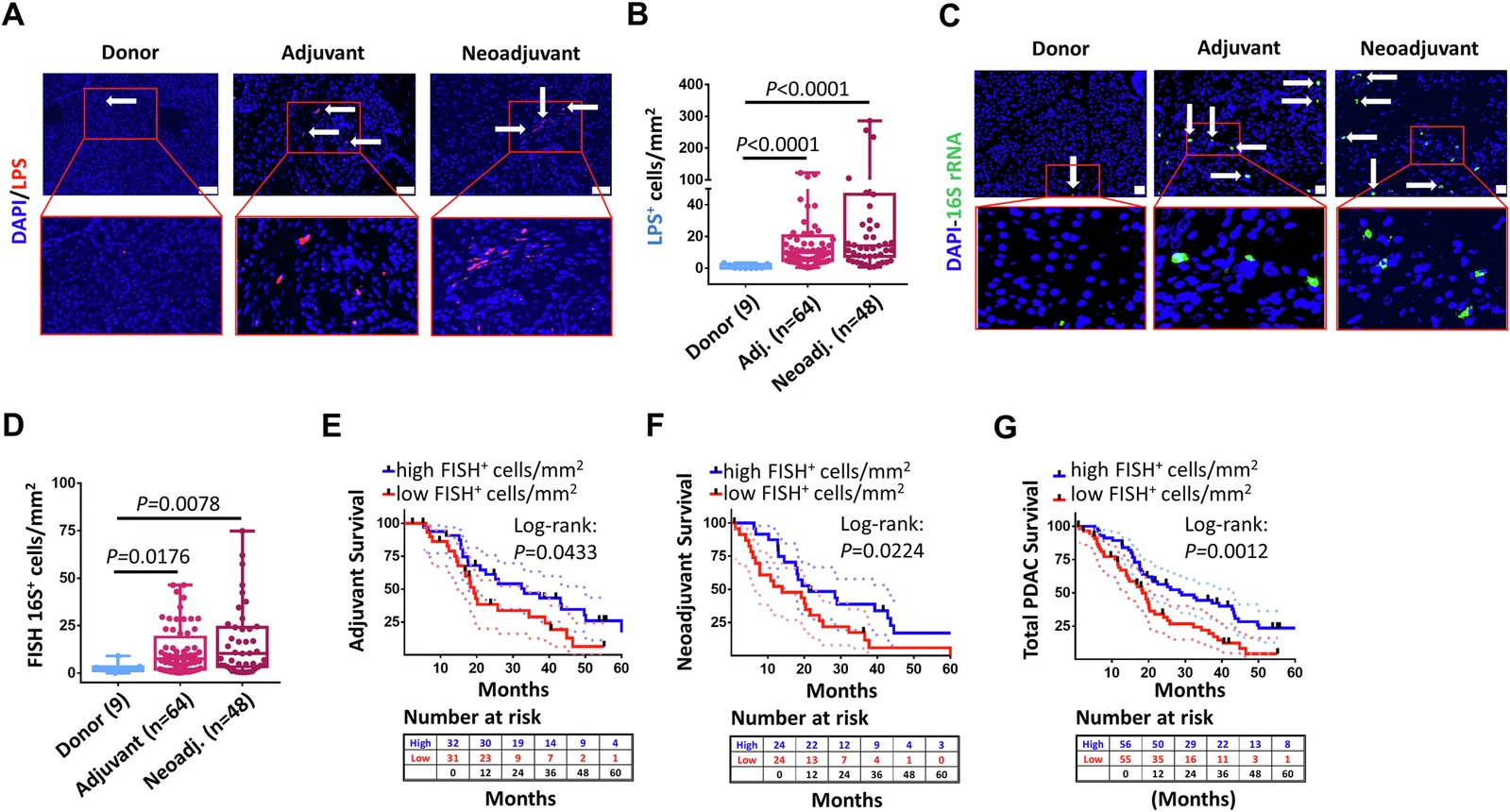
Elevated bacterial FISH signal is associated with improved survival for adjuvant and neoadjuvant pancreatic ductal adenocarcinoma (PDAC) patients. **A** Representative immunofluorescence images of pancreatic tissue from donor controls, adjuvant (chemo-naive), and neoadjuvant PDAC patients stained for LPS. Higher-magnification images highlight LPS-positive structures (scale bar = 50 µm). **B** Box-and-whisker plots with all patients/points shown. Quantitation of LPS-positive cells per mm^2^ of tissue from donor controls, adjuvant (chemo-naive), and neoadjuvant PDAC patients. Differences between groups were assessed via the Mann-Whitney U test. Significance levels (*P* values) are displayed above the respective bars/plots. **C** Representative 16S rRNA FISH images of donor controls, as well as adjuvant (chemo-naive) and neoadjuvant PDAC tissue, shown at high magnification. Arrows indicate 16S rRNA FISH-positive bacterial signals (Scale bar = 20 µm). **D** Box-and-whisker plots with all patients/points shown. Quantitation of 16S rRNA FISH-positive cells per mm^2^ tissue in donor controls, adjuvant (chemo-naive), and neoadjuvant PDAC tissue. Differences between groups were assessed via the Mann-Whitney U test. Significance levels (*P* values) are displayed above the respective bars/plots. **E** Kaplan-Meier survival analysis stratified by 16S rRNA FISH-positive cells per mm^2^ in adjuvant PDAC patients. Log-rank test *P* values and numbers at risk are shown. **F** Kaplan-Meier survival analysis stratified by 16S rRNA FISH-positive cells per mm^2^ in neoadjuvant (chemo-naive) PDAC patients. Log-rank test *P* values and numbers at risk are shown. **G** Kaplan-Meier survival analysis stratified by 16S rRNA FISH-positive cells per mm^2^ in the combined PDAC cohort. Log-rank test *P* values and numbers at risk are shown.

We further demonstrated independent, marked increases in bacterial signals using pancreatic LPS measurements and FISH in patients with adjuvant or neoadjuvant PDAC. IF signals for LPS substantially increased in adjuvant (20-fold) and neoadjuvant (40-fold) patients compared with donor controls (Fig. 5A, B), which is in accordance with the pancreatic enzymatic endotoxin/LPS concentration (Fig. 4F). 16S rRNA FISH-positive cells/mm^2^ tissue increased by 4.5-fold in adjuvant PDAC and 6.2-fold in neoadjuvant PDAC tissues compared with donor pancreata (Fig. 5C, D). Furthermore, high levels of 16S rRNA FISH-positive cells/mm^2^ stained cells above the median correlate with better survival in adjuvant PDAC with censored subjects included (median survival 33.08 months vs. 19.22) (Fig. 5E) and in neoadjuvant PDAC with censored subjects included (median survival 21.2 vs. 13.9 months) (Fig. 5F) or for the aggregate of both adjuvant and neoadjuvant PDAC with censored subjects included (median survival 29 vs. 18.3 months) (Fig. 5G). Multivariable Cox regression analysis including CA19-9, stage, therapy type, and treatment group confirmed that high intratumoral bacterial abundance (16S FISH^+^ cells/mm^2^) was independently associated with improved overall survival in PDAC patients (*P* = 0.008992, HR 0.54, 95% CI 0.34-0.86) (Supplementary Fig. 3D). Of note, 16S FISH signals similarly increased 3.5- to 6-fold in mice and 4.4- to 6.4-fold in human PDAC (Fig. 1C and Fig. 5D), suggesting similar enrichment of microbiota in mice and humans. We identified 16S positive rRNA in PDAC tissues mainly within, or in close proximity to KRT19-positive pancreatic neoplastic cells, as well as in the stromal compartment, consistent with the known capacity of intratumoral bacteria to colonize multiple tissue compartments within the PDAC microenvironment (Supplementary Fig. 5A). The 16S FISH shows specific tissue staining with higher magnification and with a competition assay (Supplementary Fig. 5B/C). 16S rRNA FISH-positive cells/mm^2^ also colocalized with a *Bacteroides*-specific FISH probe with remarkable co-staining patterns and specificity validation by competition assays with cold probes (Supplementary Fig. 6A-C), with higher levels of either Gram-positive or Gram-negative bacteria and higher relative abundances of Gram-negative *Bacteroides* and *Microvirga* in both adjuvant and neoadjuvant PDAC compared with normal pancreata (Supplementary Fig. 6D-G). These findings identify elevated intratumoral bacterial burden as a positive prognostic marker in PDAC patients.

### Autophagy is associated with improved survival in adjuvant and neoadjuvant PDAC patients

**Fig. 6:**
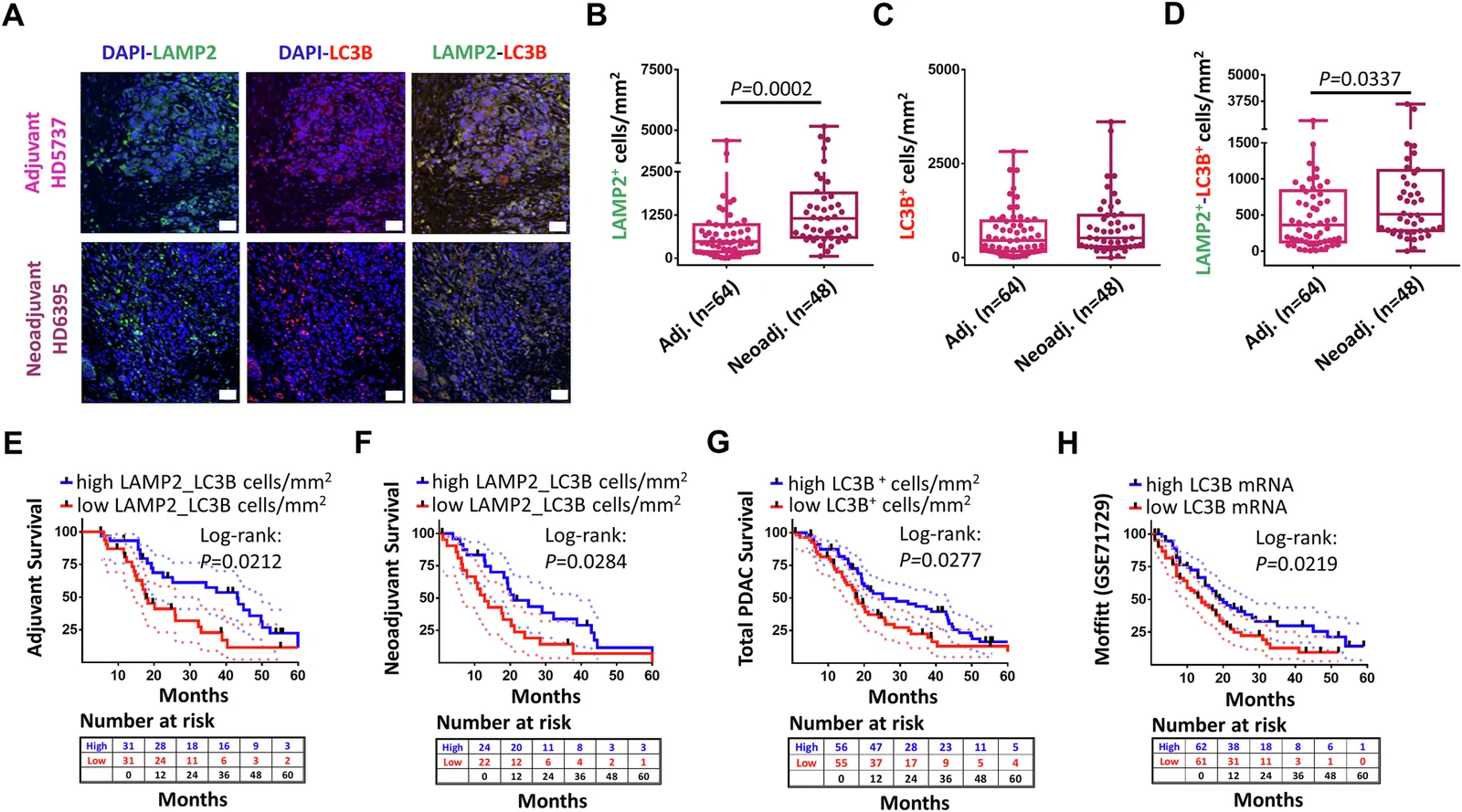
Increased autophagic markers LAMP2 and LC3B correlate with improved survival for adjuvant and neoadjuvant pancreatic ductal adenocarcinoma (PDAC) patients. **A** Representative immunofluorescence images of pancreatic tissue from donor controls, adjuvant (chemo-naive), and neoadjuvant PDAC patients stained for LAMP2 and LC3B (scale bar = 50 µm). **B** Box-and-whisker plots with all patients/points shown. Quantitation of LAMP2-positive cells per mm^2^ of tissue in adjuvant (chemo-naive), and neoadjuvant PDAC tissue. Differences between groups were assessed via the Mann-Whitney U test. Significance levels (*P* values) are displayed above the respective bars/plots. **C** Box-and-whisker plots with all patients/points shown. Quantitation of LC3B-positive cells per mm^2^ of tissue in adjuvant (chemo-naive) and neoadjuvant PDAC tissue. Differences between groups were assessed via the Mann-Whitney U test. Significance levels (*P* values) are displayed above the respective bars/plots. **D** Box-and-whisker plots with all patients/points shown. Quantitation of LAMP2 and LC3B double-positive (autophagosome-positive) cells per mm^2^ of tissue in adjuvant (chemo-naive) and neoadjuvant PDAC tissue. Differences between groups were assessed via the Mann-Whitney *U* test. Significance levels (*P* values) are displayed above the respective bars/plots. **E** Kaplan-Meier survival analysis stratified by LAMP2_LC3B double-positive cells per mm^2^ in adjuvant (chemo-naive) PDAC patients. Log-rank test *P* values and numbers at risk are shown. **F** Kaplan-Meier survival analysis stratified by LAMP2_LC3B double-positive cells per mm^2^ in neoadjuvant PDAC patients. Log-rank test *P* values and numbers at risk are shown. **G** Kaplan-Meier survival analysis stratified by LC3B positive cells per mm^2^ in combined adjuvant and neoadjuvant PDAC patients. Log-rank test *P* values and numbers at risk are shown. **H** Kaplan-Meier survival analysis stratified by LC3B mRNA expression profile using the publicly available Moffitt cohort (GSE71729) in adjuvant PDAC patients [37]. Log-rank test *P* values and numbers at risk are shown.

We further investigated autophagy as a potential mediator of microbiota-host interactions. Autophagy signaling is elevated in PDAC tissue [24, 25]. We next determined the expression of LAMP2 and LC3B in PDAC tissue (Fig. 6A). Autophagic activity, assessed by LAMP2_LC3B colocalization IF staining, was elevated particularly in neoadjuvant samples (Fig. 6B-D). High autophagy levels with colocalization of LAMP2_LC3B strongly correlated with prolonged survival across patient cohorts. In adjuvant PDAC patients, the median survival was 43.14 vs. 17.61 months, and in neoadjuvant PDAC, the median survival was 20.96 vs. 12.81 months, with both censored subjects included (Fig. 6E, F). When analyzing LC3B alone or colocalization of LAMP2_LC3B in the aggregation of both adjuvant and neoadjuvant PDAC the median survival was 25.13 vs. 17.61 months (Fig. 6G and Supplementary Fig. 3A). Moreover, this survival association was further supported by transcriptomic analysis, where elevated LC3B mRNA showed better survival in the well-recognized Moffitt cohort with a survival above the median of 20.96 months vs. 12.81 months below the median with censored subjects included [37, 38] (Fig. 6H**)**. Multivariable Cox regression analysis including CA19-9, stage, therapy type, and treatment group confirmed that increased LAMP2_LC3B cells/mm^2^ co-localization was independently associated with improved overall survival in PDAC patients (*P* = 0.006219, HR 0.52, 95% CI 0.33–0.83) (Supplementary Fig. 3E). In addition, triple staining for LPS_LAMP2_LC3B – indicative of autophagosomes containing bacterial LPS (xenophagy) – was significantly increased by ~8-fold in adjuvant and by 11.7-fold in neoadjuvant PDAC patients compared with controls (Supplementary Fig. 3B). Moreover, triple positive dots of LPS_LAMP2_LC3B (xenophagosomes) increased by ~3-fold in adjuvant and ~6-fold in neoadjuvant PDAC patients compared with controls (Supplementary Fig. 3C). LPS dots colocalized with LAMP2_LC3B dots in only 3 to 4.5% of the cases, suggesting that only few LPS-containing bacteria may be found in xenophagosomes and only LAMP2_LC3B autophagosome formation contributes to the better prognosis. Neither xenophagosomes (LPS_LAMP2_LC3B) nor LPS shows any survival correlation. Interestingly, LAMP2 and LAMP2_LC3B are both significantly higher expressed in neoadjuvant compared with adjuvant PDAC patients. We detected moderately elevated mRNA expression of autophagy markers in four publicly available GSE transcriptomic datasets (Supplementary Fig. 7A-C). These findings suggest that microbial enrichment and autophagy are functionally linked and jointly associated with favorable outcomes.

## Discussion

In this study, we demonstrate that intratumoral bacterial accumulation is a consistent feature of pancreatic ductal adenocarcinoma (PDAC) using multiple orthogonal approaches, including 16S sequencing of extracted DNA from cryo-conserved tissue, 16S rRNA fluorescence in situ hybridization (FISH) using FFPE tissue, and two different independent bacterial endotoxin detection assays (enzymatic and antibody-based IF). The concordance across methods minimizes the likelihood of contamination and strengthens the robustness of our findings. Notably, high intratumoral bacterial load was associated with prolonged survival in both adjuvant and neoadjuvant PDAC patients, consistent with previous reports [14, 17]. Those studies similarly linked specific bacterial taxa to improved survival, supporting a functional role of microbiota in pancreatic tumor biology [17, 39]. Emerging evidence further indicates that specific commensals can enhance antitumor immunity through activation of tumor-associated macrophages (TAMs), which could represent a novel clinical target to establish a microbiota-targeted therapeutic paradigm for cancer intervention [40]. For instance, *Limosilactobacillus* and *Blautia* have been shown to correlate with better prognosis, and both species have been associated with antitumor properties [41, 42]. In our study, we were unable to confirm these findings. However, the prognostic role of microbial diversity remains debated, likely due to the low biomass and technical variability inherent to pancreatic microbiome studies [14, 43–48]. In our cohort, we did not age-match the patients, and we were unable to correlate alcohol consumption with microbiota abundance. Our results demonstrate a progressive microbial enrichment and elevated  species diversity across disease stages, indicating dynamic remodeling of pancreatic microbiota during tumor evolution. The marked expansion of the intratumoral microbial community in PDAC and intra-pancreatic disease, such as CP and IPMN, likely reflects translocation from the intestinal tract into a permissive oncogenic niche characterized by immune evasion and metabolic byproduct accumulation. This expansion was confirmed by elevated alpha diversity indices, which were significantly higher in PDAC, as well as by a distinct beta diversity compared with CP, IPMN, and healthy donor controls. We observed for the first time the rising gradient of bacterial accumulation with different species from the precursor disease (CP and IPMN) to PDAC, suggesting that microbial colonization increases in tandem with tumor development. This is accompanied by activation of the LPS-TLR4 signaling axis, highlighting functional engagement of microbial sensing pathways. The concordance across these diversity measures indicates that PDAC harbors a more abundant but more homogenous microbial community compared with non-malignant pancreatic states [15, 49].

Autophagy plays a complex role in PDAC tumorigenesis. Genetic inhibition of autophagy leads to pancreatitis, exacerbates LPS-induced pancreatitis and pre-malignant lesions in mouse models, indicating a tumor-suppressive function in the early disease stage [19–21]. Deletion of autophagy genes in hepatocytes promotes liver tumors, suggesting that autophagy loss promotes tumorigenesis [22]. Beyond antimicrobial defense, xenophagy also shapes host immunity by modulating microbial antigen presentation and constraining excessive inflammatory cascades [23].

We further investigated autophagy as a potential mediator of microbiota-host interactions. Increased autophagic activity strongly correlated with improved survival across the PDAC patient cohorts. In PDAC tissue, we observed elevated LPS colocalization with the autophagic markers LAMP2_LC3B, indicating the existence of xenophagosomes (autophagosomes containing bacterial LPS). These findings suggest a functional link between microbial enrichment and autophagy, both of which are jointly associated with favorable patient outcomes. Although autophagy has context-dependent roles in cancer, our results consistently show elevated autophagy markers and elevated 16S rRNA FISH-positive cells/mm^2^ in PDAC with a positive survival association. Both Kaplan-Meier survival analysis and multivariable Cox regression revealed that high intratumoral bacterial abundance (FISH 16S^+^ cells/mm^2^) and increased LAMP2_LC3B^+^ cells/mm^2^ co-localization were independently associated with improved overall survival, indicating that bacterial abundance and autophagy represent independent prognostic factors in PDAC. Patients exhibiting high levels of both markers have the most favorable prognosis, supporting a model in which bacterial sensing and autophagy represent complementary biological processes associated with tumor control and prognosis.

Our study showed for the first time a direct correlation between intratumoral bacterial burden, autophagic LAMP2_LC3B co-localization, and patient survival across two independent PDAC cohorts using orthogonal tissue-based methods. Methodological controls, including multiple negative controls and cross-validation across techniques, support the validity of our microbiome analysis.

The mechanisms by which the microbiota influences PDAC prognosis remain incompletely understood. We propose that autophagy facilitates the processing of intratumoral microbes, thereby modulating immune responses. Microbial signaling through TLR4 may enhance immunogenic pathways, thereby promoting antitumor immunity. Despite reports of immunosuppressive roles of autophagy, our results support a model in which, in the context of high microbial burden, autophagy contributes to a more immunologically active antitumor microenvironment. Collectively, our findings define a bidirectional microbiota-autophagy axis that shapes PDAC biology and clinical outcome.

The mechanistic link between intratumoral bacteria and autophagy can be most plausibly framed through the LPS-TLR4 signaling axis. Gram-negative bacteria, including *Bacteroides*, *Porphyromonas*, and *Fusobacterium*, which are enriched in PDAC tumors, release LPS during bacterial growth and turnover [3, 5]. LPS is recognized by the CD14/MD-2-TLR4 receptor complex on tumor and stromal cells, triggering predominantly MyD88-dependent signaling. MyD88 recruits IRAK kinases and TRAF6, which activate the TAK1-IKK signaling cascade, leading to IκBα degradation and nuclear translocation of NF-κB (p65/RelA). NF-κB can then transcriptionally upregulate autophagy-related genes: p65/RelA binds a conserved NF-κB response element in the BECN1 promoter, directly increasing BECLIN1 expression [50], while the IKK/NF-κB axis more broadly promotes ATG5 and other autophagy-related proteins [51]. Components of this cascade have been demonstrated in PDAC cells [16, 52, 53], and the TLR4-TRAF6-autophagy link has also been shown in lung cancer cells [54]. We fully acknowledge that definitive rescue experiments - for example, genetic ablation of ATG5 or BECLIN1 in PDAC cells combined with controlled manipulation of selected intratumoral bacterial load - would provide the strongest mechanistic evidence for a bacteria-autophagy-survival axis. Experimental models of pancreatic autophagy depletion show that autophagy can support the survival of Kras-driven pancreatic precancerous cells while also maintaining normal pancreatic tissue integrity. Consequently, autophagy inhibition may suppress tumor progression in some contexts but exacerbate disease in others, particularly when p53-dependent stress checkpoints are absent [55, 56]. Riquelme et al. showed that fecal microbiota transplantation from long-term PDAC survivors into germ-free, Kras-driven pancreatic cancer/precancerous mice conferred a survival advantage and enhanced antitumor immunity, directly implicating microbiota composition in oncologic outcome [17]. In addition, antibiotic-mediated depletion of the gut microbiota significantly reduced pancreatic carcinogenesis in Kras-driven mice [57]. In germ-free C57BL/6 J mice orthotopically implanted with Pan02 PDAC cells, the gut microbiota suppresses intratumoral NK-cell infiltration and IFN-γ expression, and NK-cell depletion reversed the tumor-protective effect of microbiota ablation [58].

Indirect clinical support for the functional relevance of autophagy in PDAC comes from multiple phase II trials combining the autophagy inhibitor hydroxychloroquine (HCQ), which also influences lysosomal trafficking, with gemcitabine with or without nab-paclitaxel. These trials and others support the premise that autophagy can be pharmacologically targeted in PDAC, but they have not shown a consistent overall survival benefit as reported for several other trials [28–30]. Taken together, the preclinical microbiota studies and the clinical HCQ data provide complementary evidence that both microbiota composition and autophagy are functionally relevant in PDAC. Our correlational findings extend this framework to the specific association between intratumoral bacteria, autophagy-related signatures, and patients’ survival across two independent PDAC cohorts.

## Methods

### Patient characteristics

The study was approved by the Ethics Committee of Heidelberg University for the use of human tissue samples (Approval No. S-141/2019 and S-083/2021), according to the Helsinki Declaration. This study included a retrospective patient cohort with FFPE tissue to determine LPS and autophagy by IF staining and cryo tissue to isolate tissue DNA (16S rRNA intra-pancreatic microbiota) and protein for tissue LPS (enzymatic assay). This study also included a prospective cohort, registered under DRKS00028995 at the German Clinical Trials Register. All tissues were collected and processed by the institutional tissue bank of the Department of Surgery Clinic at Heidelberg University. Sample availability differed between experiments due to the limited availability of matched FFPE and cryopreserved tissue. All patients provided written informed consent, and samples were pseudo-anonymized according to the ethical guidelines. Patients were classified into five groups, excluding those with metastatic PDAC or those who received chemoradiation therapy: i) neoadjuvant PDAC patients with locally advanced or borderline unresectable tumors who received mFOLFIRINOX or gemcitabine-based therapy prior to surgical resection (*n* = 62; ii) adjuvant chemo-naïve PDAC patients with resectable tumor at diagnosis (*n* = 101; iii) patients diagnosed with intraductal papillary mucinous neoplasm (IPMN) (n = 24); iv) patients diagnosed with chronic pancreatitis (CP) (*n* = 33); v) healthy donor pancreata (*n* = 9) (Table 1A-C). All diagnoses were confirmed by histopathological evaluation. After surgical resection, tissues were either processed for formalin-fixed, paraffin-embedded (FFPE) tissues or snap frozen in liquid nitrogen. FFPE or cryo tissue was stained with Hematoxylin & Eosin (H&E) and was evaluated by a pancreas pathologist (FB) for staging and tumor content in the Department of Pathology as described recently [59]. Clinical data are summarized in Table 1A-C.

### Animals

Mice were maintained under standard semi-pathogen-free conditions. All experiments were approved by the Institutional Animal Care and Use Committee at Heidelberg University in accordance with guidelines issued by the Federal Presiding Board for Animal Care, Karlsruhe, Germany (G-110/20, G-24/17). LSL-Kras^G12D^ mice were crossed with Ptf1a/p48-Cre mice, which have a p48 promoter-driven Cre recombinase, to generate Kras-Crep48 (KrasCre) mice, which were kindly provided by David Tuveson [33]. All mice were maintained on a C57BL/6 (C57 Black/6) background. No randomization or blinding was applied for the experimental groups.

### Animal experimental design

KrasCre and control Kras/B6 mice were used to assess intrapancreatic bacterial burden and autophagy signaling. Mice received intraperitoneal injections of LPS (2 mg/kg) or PBS twice weekly for 7 weeks starting at 25 weeks of age. Mice were euthanized at 32 weeks of age, and pancreas tissue was collected. Control Kras/B6 mice were treated with the same volume of PBS. Tissues were washed in sterile cold PBS, sterilely cut into two pieces, either snap frozen in liquid nitrogen and kept at -80 °C or immersed in 4% phosphate-buffered formaldehyde solution for preparing formalin-fixed-paraffin-embedded (FFPE) tissue sections.

### Genotyping

DNA was extracted using a standard tissue-to-PCR protocol (Thermo Fisher Scientific, Waltham, MA, USA). PCR amplification was performed using specific primers for the detection of Kras and p48-Cre, followed by standard DNA electrophoresis, as described previously [20, 60, 61]. The primers used in the PCR reaction are listed: Kras forward: 5’-CCTTTACAAGCGCACGCAGACTGTAGA-3’, Kras reverse: 5’-AGCTAGCCACCATGGCTTGAGTAAGTCTGCA-3’; p48-Cre forward: 5’-ACCGTCAGTACGTGAGATATCTT-3’, p48-Cre reverse: 5’-ACCTGAAGATGTTCGCGATTATCT-3’.

### Endotoxin (LPS) assay

Frozen pancreatic tissue was homogenized, and endotoxin levels were quantified using a chromogenic endotoxin quant kit (Thermo Fisher Scientific) in microplate format according to the manufacturer’s instructions and as described previously [20]. For our samples, we measured Endotoxin Units (EU) per g tissue, which describes the biological impact of the endotoxins.

### Fluorescence in situ hybridization (FISH)

A FISH assay was performed on human and mouse FFPE tissue sections using a universal 16S rRNA probe (Alexa/650-5’-GCTGCCTCCCGTAGGAGT-3’) and a *Bacteroides*-specific probe (Bfra602 ATTO594-5´-GAGCCGCAAACTTTCACAA-3´); both FISH probes have been described previously [14, 62, 63]. After standard pre-treatment and hybridization, whole tissue sections were captured and analyzed using a TissueFAXS Fluorescence Imaging System (Tissue-Gnostics). Images were acquired using either an Axio Observa.Z1 fluorescence microscope unit (Zeiss) equipped with a Lumencor Sola SE III LED light source) or a TissueFAXS LS slide loader high-throughput imaging system (Tissue-Gnostics). Data analysis was performed using (Tissue-Gnostics)StrataQuest software version 7.0 (TissueGnostics), as described recently [20, 59, 60].

### Western blotting (WB)

Human and mouse pancreas tissues were prepared under standard conditions, and protein levels were analyzed by SDS-PAGE and immunoblotting. ImageJ software was adopted for semi-quantification of the target protein by calculating target fragment intensity divided by the loading/housekeeping control GAPDH, as described [60]. The antibodies used in this study are listed in Supplementary Table 1. We acknowledge that GAPDH is an imperfect loading control in heterogeneous PDAC tissue, where glycolytic activity and HIF-1α-mediated transcriptional upregulation introduce inter-sample variability independent of protein loading. For future experiments, total protein staining (e.g., REVERT^TM^ or Stain-Free technology) represents a more robust normalization strategy for heterogeneous tumor tissue. Full and uncropped western blot images are provided in the supplementary material.

### Immunofluorescence (IF)

Immunofluorescence staining was performed to assess tissue expression of LPS, LAMP2, and LC3B, and their colocalization was assessed using 4-µm-thick FFPE tissue sections. After antibody testing, the anti-LAMP2 and anti-LC3B antibodies were directly labeled using the SiteClick Alexa Fluor 647 sDIBO Alkyne (C20029) for LC3B and SiteClick Alexa Fluor 555 sDIBO (C20028) for LAMP2 according to the instructions. The anti-LPS was used with a secondary anti-mouse-AF 488 antibody. After the labeling procedure, directly and indirectly labeled antibodies were also tested for functionality (Supplementary Table 1). Images were acquired using a TissueFAXS Fluorescence Imaging System (Tissue-Gnostics), with a fluorescence microscope unit, either an Observer Z1 (Zeiss and a Lumencor Sola SE III LED light source) and TissueFAXS LS slide loader high-throughput systems for up to 120 slides. Captured images were analyzed to calculate fluorescence signal intensity within every single cell across the entire tissue sections, as described recently [59].

### DNA extraction

DNA was extracted from human pancreatic cryo tissue under sterile conditions using Qiagen QIAamp DNA Mini Kits under rigorous contamination control according to the instructions. The tissue pieces were homogenized with an electric Teflon homogenizer, sterilized for every tissue piece in order to avoid cross-contamination and to disrupt the bacterial cell wall. Extensive negative controls were included to monitor contamination at extraction and PCR stages. To detect lab-introduced contaminants, we processed a total of 615 negative controls alongside our samples. This included 308 DNA extraction controls to monitor for contamination during the initial DNA isolation steps (EB, elution buffer *n* = 154; nFW, nuclease-free water *n* = 154), and 307 no-template controls (NTCs) to check for contamination during the PCR amplification (PCRmix, PCR reagents with no template *n* = 154; PCRmix_w_nfW, PCR reagents with nuclease-free water *n* = 153). To also address potential contamination from FFPE slides, we ran an additional 153 paraffin-only controls that were collected from paraffin blocks, without any tissue. Bacterial species retrieved from blank samples were used to compute a pooled threshold at the 99th percentile of the relative abundance distribution, as described recently by Nejman et al. [14], and results (PDF with graphs, Excel file with statistics and raw data, text with explanation of Excel file tabs, whole analysis log file) are provided here: https://github.com/valerioiebba/PDAC_blank_threshold/tree/main/PDAC_intratumoral_UPD. R script, along with necessary files and instructions to reproduce the output files for the blank threshold, is provided within the GitHub repository: https://github.com/valerioiebba/PDAC_blank_threshold/tree/main. Only species in positive samples whose relative abundance was higher than or equal to the 99th percentile of blanks were kept for further analysis.

### Real-time PCR

Bacterial abundance was quantified by qPCR targeting 16S rRNA, as described recently [14]. The relative quantification was expressed as 2^(-ΔCt) as described previously [60]. The primers for the real-time PCR reaction are listed in Supplementary Table 1. We also estimated bacterial DNA content in the tissue by determining bacterial 16S rRNA with a standard curve, which was calculated with increasing amounts of E. coli bacterial DNA, spiked into 150 ng of human DNA. qPCR amplifications were made according to MIQE 2.0 guidelines [64].

### 16S rRNA gene sequencing

Microbiota analysis was performed as described recently [5, 65]. DNA extracted from pancreatic tissue samples obtained  from donor control, PDAC, IPMN, and CP patients was used in the 16S rRNA protocol [66, 67]. DNA quality was verified via NanoDrop spectrophotometry (A260/280 ratios), while quantification used Qubit fluorometry to ensure sufficient yield for 16S rRNA amplification (targeting V3-V4 regions). The 16S rRNA metagenomic library was prepared per the Illumina protocol (San Diego, CA, USA), amplifying the V3-V4 regions with denaturing primers in limited-cycle PCR, followed by AMPure XP bead purification (Beckman Coulter, Brea, CA, USA) [68]. Dual-indexed adapters (Nextera XT Index Kit) were attached in a second PCR, with additional bead clean-up. Library concentration was quantified via Qubit 2.0 fluorometry (Invitrogen, Carlsbad, CA, USA) and validated on a Bioanalyzer DNA 1000 chip (1:50 dilution). After calculating nM concentrations based on amplicon size, libraries were pooled equimolarly and sequenced on an Illumina NovaSeq 6000 (2×250 bp, 5% PhiX spike-in) [69]. Negative controls were included by applying the exact same extraction without clinical samples. (See “DNA Extraction” paragraph for specifications). PCR products were ligated to the sequencing adapters and paired-end sequenced on an Illumina MiSeq system (250 cycles).

### Data analysis

Raw FASTQ files were analyzed with the DADA2 pipeline v.1.14 for quality check and filtering (sequencing errors, denoising, chimerae detection). Filtering parameters were as follows: truncLen = 0, minLen = 100, maxN = 0, maxEE = 2, truncQ = 11, trimLeft = 15. All the other parameters in the DADA2 pipeline for paired-end were left at default. Bioinformatic and statistical analyses on recognized ASVs were performed with Python v.3.8.2. Each ASV sequence underwent a nucleotide BLAST using the National Center for Biotechnology Information (NCBI) BLAST software (ncbi-blast-2.3.0) and the latest NCBI 16S Microbial Database (https://ftp.ncbi.nlm.nih.gov/blast/db/). Microbial species that did not have a biological meaning (environmental, rumen, extra-mammals, food, etc.) were excluded from the raw data. In addition, a strict prevalence cutoff of 20% was employed to increase the robustness of the differential abundance analysis (DA) [70–72], resulting in 73 species being considered for subsequent statistical analyses. Measurements of alpha diversity (within-sample diversity) such as Richness and the Shannon index were calculated at the species level using the SciKit-learn package v1.0.1, starting from raw read counts. Data matrices were processed for beta diversity and differential analysis (DA) following three seminal papers which benchmarked against “ground truth” the compositional data analysis in high- and low-complexity microbiome datasets [46, 70, 71, 73]. Data matrices were normalized and standardized using Quantile-Transformer and StandardScaler methods from the Sci-Kit learn package v1.0.1 [74], ruling out total sum-scaling (TSS) or centered log-ratio (CLR) data transformations in order to avoid paradoxical results, especially in low-biomass or low-complexity microbial ecosystems. Normalization using the output_distribution = ‘normal’ option transforms each variable to a Gaussian-like shaped distribution, whilst the standardization results in each normalized variable having a mean of zero and variance of one [74]. Exploratory ecological analysis of beta diversity (between-sample diversity) was calculated using the Bray-Curtis measure of dissimilarity and represented in Principal Coordinate Analyses (PCoA), along with methods to compare groups of multivariate sample units (analysis of similarities - ANOSIM, permutational multivariate analysis of variance - PERMANOVA) to assess significance in data point clustering. ANOSIM and PERMANOVA were automatically calculated after 999 permutations, implemented with custom scripts (Python v3.8.2, Seaborn v0.11.2, SciKit-learn v1.0.1) [75]. We implemented Partial Least Squares Discriminant Analysis (PLS-DA) and the subsequent Variable Importance Plot (VIP) as a supervised analysis, wherein the VIP values (equal or higher than 1, default settings) are used to identify the most discriminant bacterial species among the cohorts. The Mann-Whitney U test and Kruskal-Wallis test were employed to assess significance for pairwise or multiple comparisons, respectively, considering a *P* value ≤ 0.05 as significant. Clustermaps of microbial abundances were generated with Euclidean distance and Ward linkage algorithms by means of custom scripts (Python v3.8.2, Seaborn v0.11.2, SciKit-learn v1.0.1). Where clearly stated, all *P* values were corrected for multiple hypothesis testing using a two-stage Benjamini-Hochberg FDR at 10% [76].

### Statistical analysis and GEO database

We downloaded from the GEO database (https://www.ncbi.nlm.nih.gov/geo/) 4 publicly available PDAC expression datasets containing tumor and control samples (GSE15471 [77], GSE56560 [78], GSE62165 [79], and GSE62452 [80], with 254 PDAC and 120 controls without survival data. We also included the Moffitt cohort (GSE71729) with 123 PDAC cases because of the available PDAC survival data [37]. We applied the software GraphPad Prism 6 for survival analysis, using Kaplan-Meier and Log-rank Mantel-Cox tests. Multivariant cox regression analysis was performed with 16S FISH^+^ cells/mm^2^ and LAMP2_LC3B^+^ cells/mm^2^ data in correlation with the clinical data, such as preoperative CA19-9, tumor stage, and survival, using R software package. All expression data were presented as box-and-whisker plots (Min to Max showing all points or 10-90 percentiles). Statistical significance analysis was performed by Student’s *t* Test, nonparametric Mann-Whitney test comparing two groups analysis or for multiple testing using Mann-Whitney test with Holm-Bonferroni correction. The option of “identify outliers” of each target was also performed with this software. Results with a *P* value ≤ 0.05 were considered to be statistically significant.

## Supplementary information

Supple. Figure and Legends

WesternBlot original

Source Data Fig. 1-6

Source Data Fig. 1-7

## Data Availability

Raw FASTQ data of 16S targeted sequencing are available at NCBI Sequence Read Archive - BioProject ID: PRJNA1310990. The 16S rRNA sequencing data generated in this study are deposited in the NCBI BioProject database under accession number PRJNA1310990. Transcriptomic datasets analyzed in this study are publicly available from the NCBI GEO database (GSE15471, GSE56560, GSE62165, GSE62452, GSE71729). All other data supporting the findings of this study are available from the corresponding author upon reasonable request.
