# Supplementary material for: Autophagy and intratumoral bacteria abundance influence the prognosis of patients with pancreatic carcinoma
Source: Cell Death Dis. 2026 Oct 27;17(1):916. doi: 10.1038/s41419-026-09256-1 (PMC13720179; doi:10.1038/s41419-026-09256-1)
Supplement: Supplementary file 1 [file 41419_2026_9256_MOESM1_ESM.pdf]

Supplemental Figures

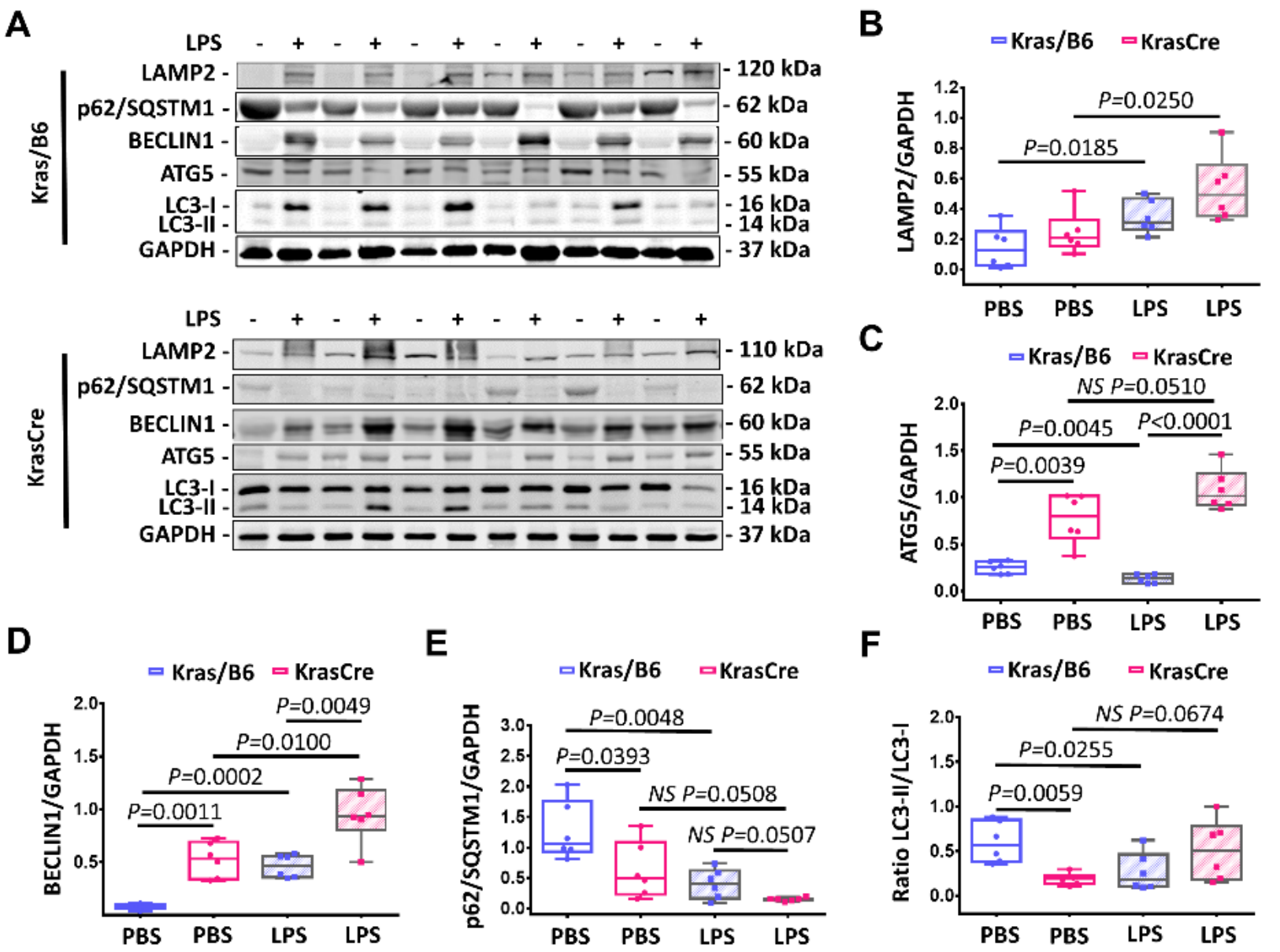

Supple. Figure 1. LPS induces autophagy-related signaling in pancreatic tumor precursor lesions. (A) Representative immunoblots from KrasCre and Kras/B6 controls mice +/- LPS Treatment showing LAMP2, p62/SQSTM1, BECLIN1, ATG5, LC3B, and GAPDH (loading control). **(B-F)** Densitometric quantification of LAMP2/GAPDH ratio (B), ATG5/GAPDH ratio (C), BECLIN1/GAPDH ratio (D), p62/SQSTM1/GAPDH ratio (E), and semi-quantitation of LC3-II/LC3-I ratio (F). Statistical significance was assessed using the nonparametric Mann-Whitney test and presented as box-and-whisker plots; min-max with all points/animals shown. Significance levels (P-values) are displayed above the respective bars/plots.

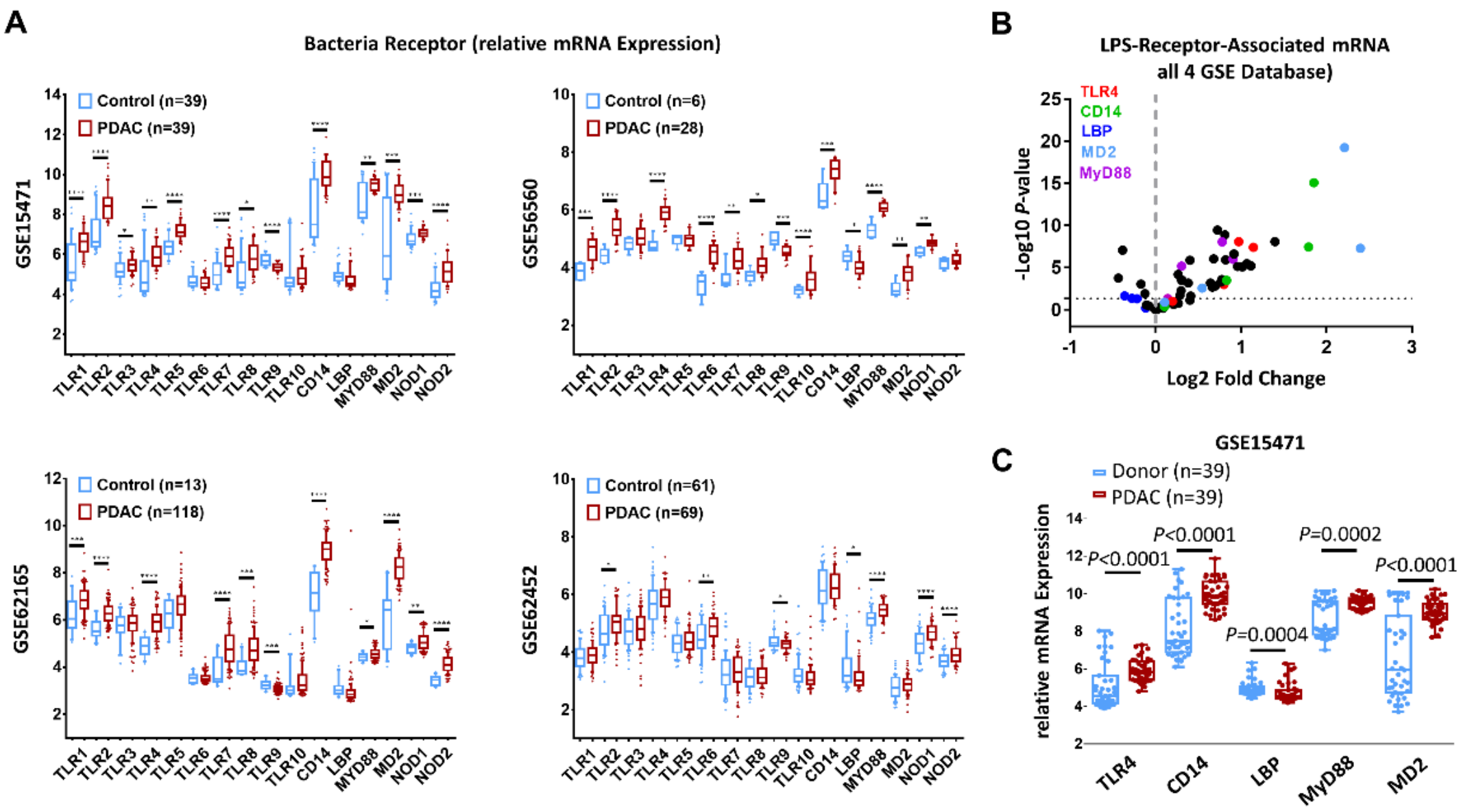

Supple. Figure 2. Meta-analysis of LPS-associated receptors/adaptors in pancreatic ductal adenocarcinoma (PDAC) patients vs. donor tissue. (A) Microarray expression data from four GEO (GSE) datasets comparing PDAC and donor controls (box-and-whisker plots, 10-90 percentile; * $P < 0.05$, ** $P < 0.01$, *** $P < 0.001$, **** $P < 0.0001$). **(B)** Volcano plot highlighting differential enrichment of LPS-associated receptor/adaptor genes in PDAC vs. donor samples (log2 fold change > 1 and −log10 (P-values >2). **(C)** Expression of TLR4, CD14, LBP, MYD88, and MD2 in PDAC vs. controls in the GSE15471 dataset (box-and-whisker plots, min-max all patients/points). Statistical significance was assessed using the nonparametric Mann-Whitney test and presented as box-and-whisker plots; min-max with all points/patients shown. Significance levels (P-values) are displayed above the respective bars/plots.

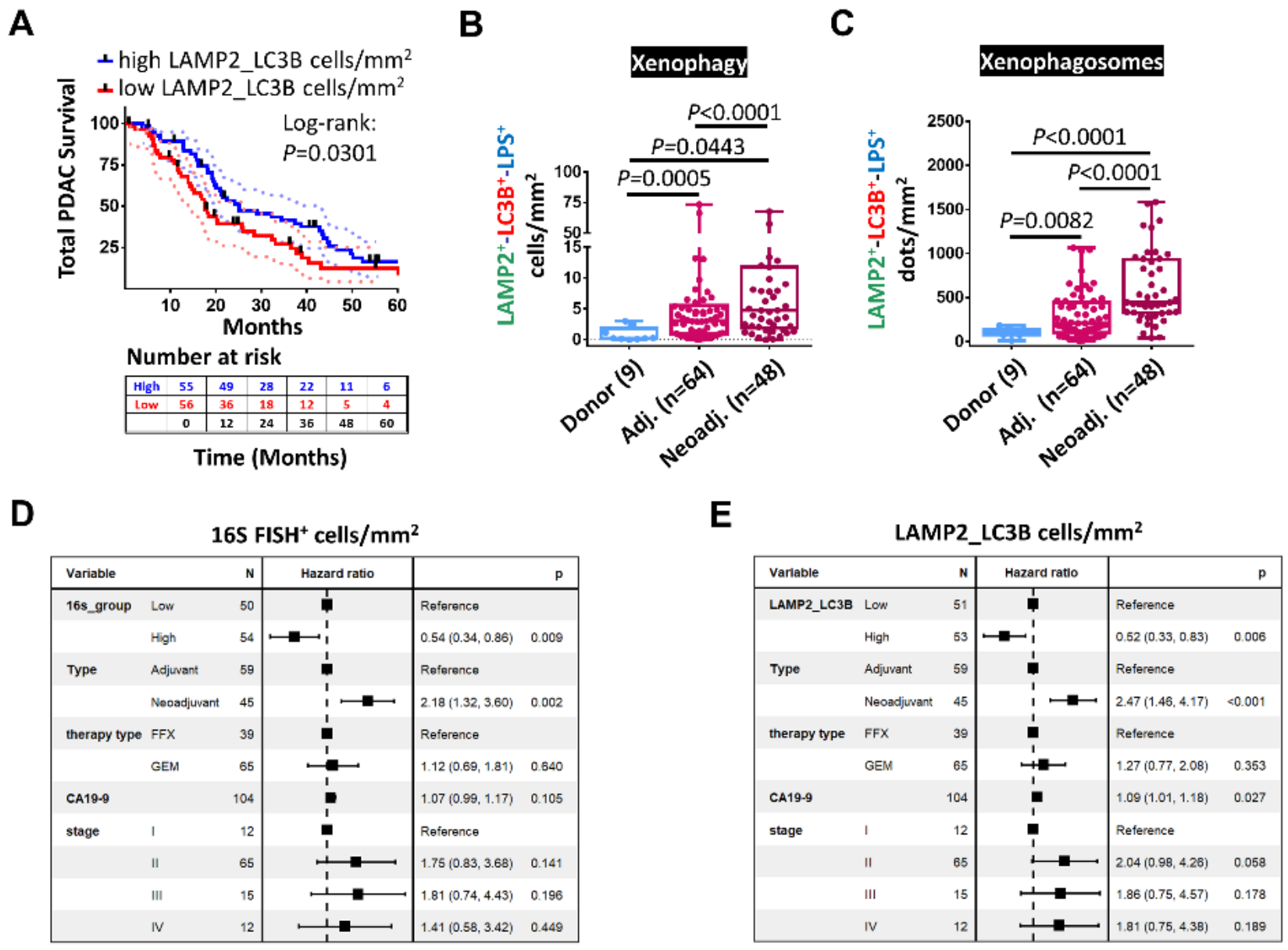

Supple. Figure 3. Survival analysis across pancreatic ductal adenocarcinoma (PDAC) patients tissue samples. (A). Kaplan-Meier survival analysis in the combined PDAC cohort stratified by LAMP2_LC3B double-positive cells per mm². Log-rank test P-values and numbers at risk are shown. **(B)** Box-and-whisker plots with all patients/points shown. Quantitation of xenophagy of LAMP2_LC3B_LPS positive cells per mm^2 tissue in adjuvant (chemo-naive), neoadjuvant PDAC and donor tissue. Differences between groups were assessed via the Mann-Whitney U test. Significance levels (*P*-values) are displayed above the respective bars/plots. **(C)** Box-and-whisker plots with all patients/points shown. Quantitation of xenophagosomes of triple-positive LAMP2_LC3B_LPS dots per mm^2 tissue in adjuvant (chemo-naive), neoadjuvant PDAC, and donor tissue. Differences between groups were assessed via the Mann-Whitney U test. Significance levels (*P*-values) are displayed above the respective bars/plots. **(D)** Multivariant cox regression analysis including the variables CA19-9, tumor stage, therapy type, and treatment group in correlation to FISH $16S^+$ cell/mm^2 showing variable (left), hazard ratio (middle), and values of hazard ratio, 95% CI, and *P*-values (right). **(E)** Multivariant cox regression analysis including the variables CA19-9, tumor stage, therapy type, and treatment group in correlation to LAMP2_LC3B$^+$ cells /mm^2 co-localization showing variable (left), hazard ratio (middle), and values of hazard ratio, 95% CI, and *P*-values (right).

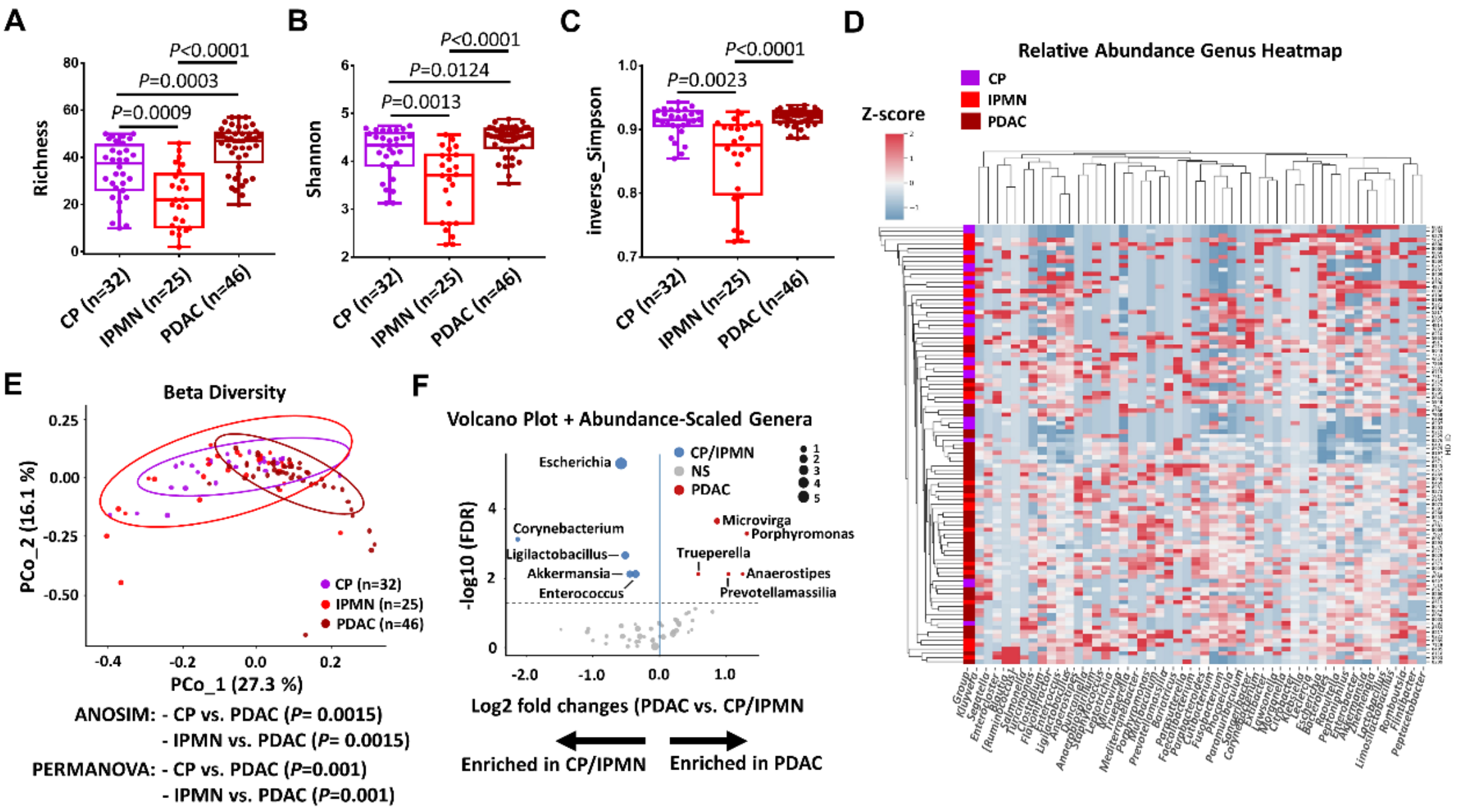

Supple. Figure 4. Distinct tissue microbiota profiles in chronic pancreatitis (CP) and intraductal papillary mucinous neoplasms (IPMN) and pancreatic ductal adenocarcinoma (PDAC) patients. (A) Richness alpha diversity index in CP, IPMN and PDAC tissue. Statistical significance was assessed using the nonparametric Mann-Whitney test and presented as box-and-whisker plots; min-max with all patients/points shown. Significance levels (P-values) are displayed above the respective bars/plots. **(B)** Shannon alpha diversity index in CP, IPMN, and PDAC tissue. Statistical significance was assessed using the nonparametric Mann-Whitney test and presented as box-and-whisker plots; min-max with all patients/points shown. Significance levels (P-values) are displayed above the respective bars/plots. **(C)** Inverse Simpson alpha diversity index in CP, IPMN, and PDAC tissue. Statistical significance was assessed using the nonparametric Mann-Whitney test and presented as box-and-whisker plots; min-max with all patients/points shown. Significance levels (P-values) are displayed above the respective bars/plots. **(D)** Heatmap/Clustermap of Z-scored relative abundances of microbial genera showing distinct community patterns in CP, IPMN and PDAC samples. **(E)** Principal coordinate analysis (PCoA) of beta diversity showing between-group differences in community composition among CP, IPMN, and PDAC patients. Significance was assessed using ANOSIM and PERMANOVA (1000 permutations). **(F)** Volcano plot (plot log2 fold changes and -log10

P -values) and abundance-scaled genus value with the top 5 genera in PDAC and CP/IPMN patients. The right site indicates genera enriched in PDAC. The left side indicates the genera enriched in CP/IPMN.

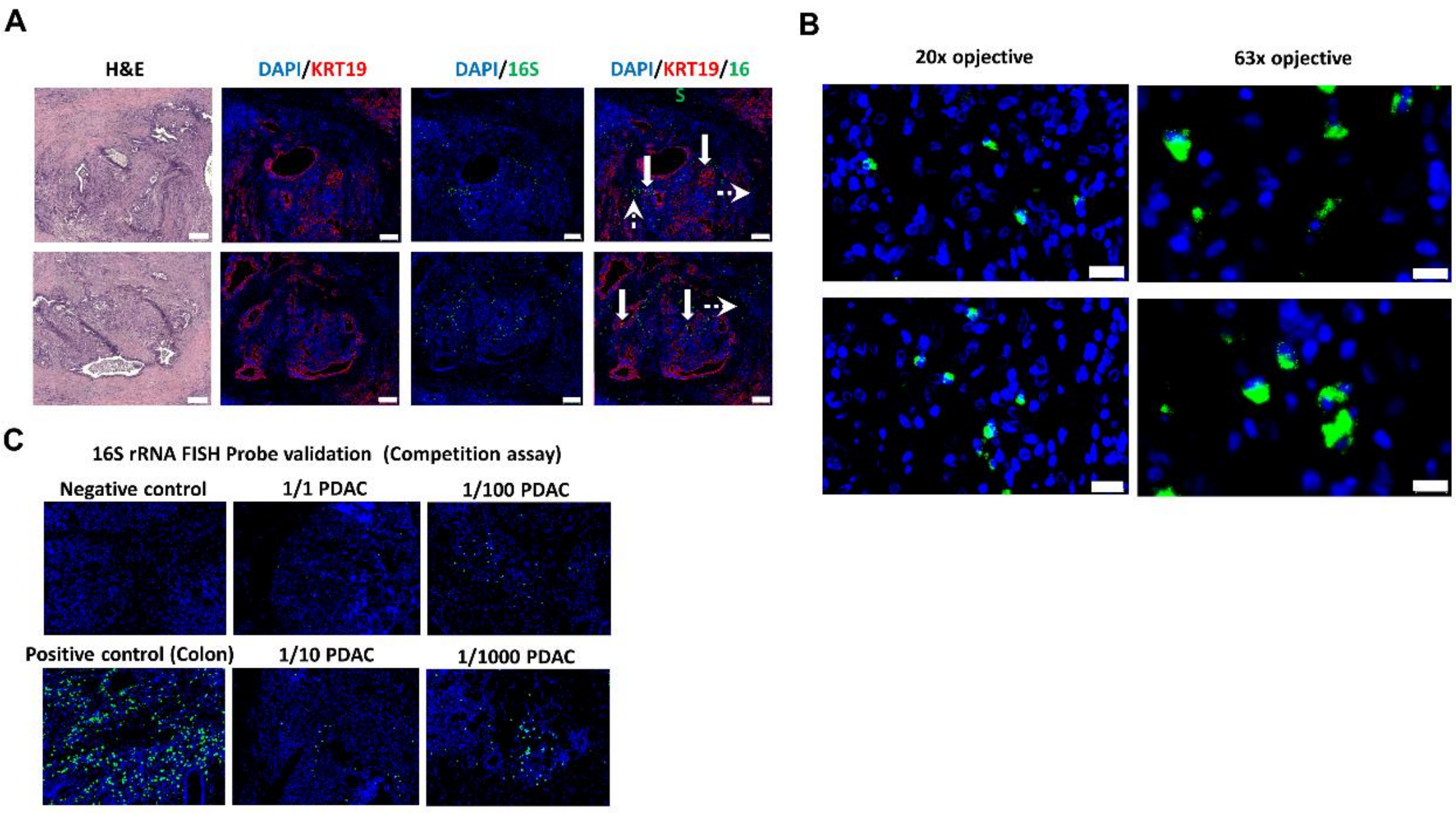

Supple. Figure 5. Validation of the 16S rRNA FISH assay. (A) Representative PDAC tissue stained with H&E and combined KRT19/16S rRNA FISH. Open arrows indicate FISH-positive stromal cells; closed arrows indicate FISH-positive KRT19-positive neoplastic cells. Scale bar, 50 μm. **(B)** Representative 16S rRNA FISH images at low (scale bar, 50 μm) and high magnification (scale bar, 10 μm). **(C)** Competition assay using increasing concentrations of unlabeled ("cold") probe to validate the specificity of the labeled 16S rRNA FISH probe.

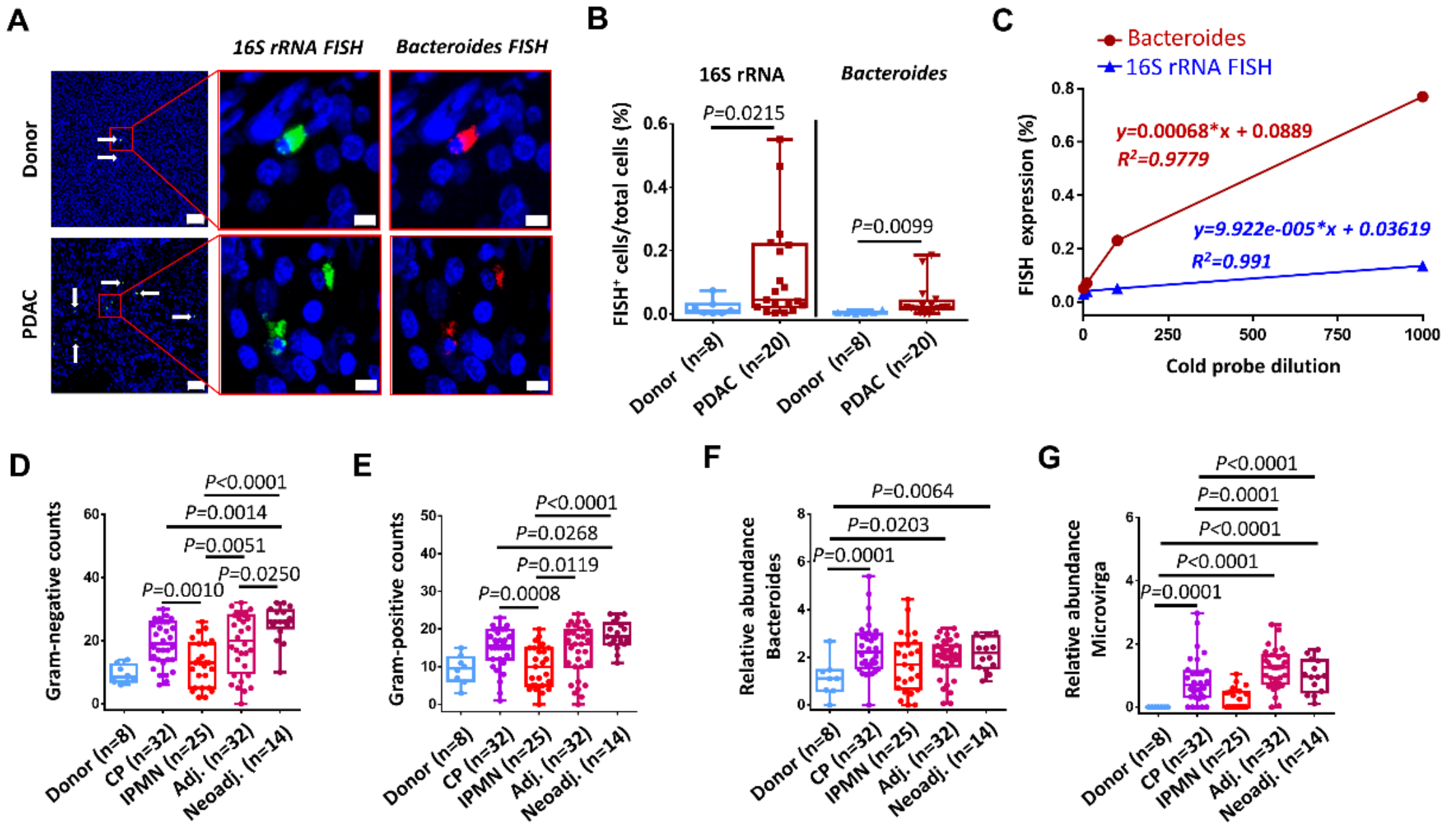

Supple. Figure 6. Enrichment of specific taxa in adjuvant (chemo-naïve) and neoadjuvant PDAC. (A) Representative 16S rRNA and Bacteroides FISH images at low (scale bar, 50 μm) and high magnification (scale bar, 5 μm). **(B)** Quantification of 16S rRNA FISH-positive cells and Bacteroides FISH-positive cells per mm^2 tissue. Statistical significance was assessed using the nonparametric Mann-Whitney test and presented as box-and-whisker plots; min-max with all patients/points shown. Significance levels (*P*-values) are displayed above the respective bars/plots. **(C)** Representative analysis of the competition assay (cold unlabeled vs. labeled probe) showing the corresponding regression slope (R^2) for 16S rRNA and Bacteroides FISH. Statistical significance was assessed using pairwise t tests with False Discovery Rate (FDR) correction for multiple testing and presented as *P*-values over the corresponding bar chart (box and whisker plots). **(D)** Quantification of Gram-negative counts obtained from the 16S microbiota sequencing results in donor, CP, IPMN, adjuvant and neoadjuvant patients. Statistical significance was assessed using the nonparametric Mann-Whitney test and presented as box-and-whisker plots; min-max with all patients/points shown. Significance levels (*P*-values) are displayed above the respective bars/plots. **(E)** Quantification of Gram-positive counts obtained from the 16S microbiota sequencing results in donor, CP, IPMN, adjuvant and neoadjuvant patients. Statistical significance was assessed using the nonparametric Mann-Whitney test and

presented as box-and-whisker plots; min-max with all patients/points shown. Significance levels (*P*-values) are displayed above the respective bars/plots. **(F)** Quantification of the relative abundance values of *Bacteroides* obtained from the 16S microbiota sequencing results in donor, CP, IPMN, adjuvant, and neoadjuvant patients. Statistical significance was assessed using the nonparametric Mann-Whitney test and presented as box-and-whisker plots; min-max with all patients/points shown. Significance levels (*P*-values) are displayed above the respective bars/plots. **(G)** Quantification of the relative abundance values of *Microvirga* obtained from the 16S microbiota sequencing results in donor, CP, IPMN, adjuvant, and neoadjuvant patients. Statistical significance was assessed using the nonparametric Mann-Whitney test and presented as box-and-whisker plots; min-max with all patients/points shown. Significance levels (*P*-values) are displayed above the respective bars/plots.

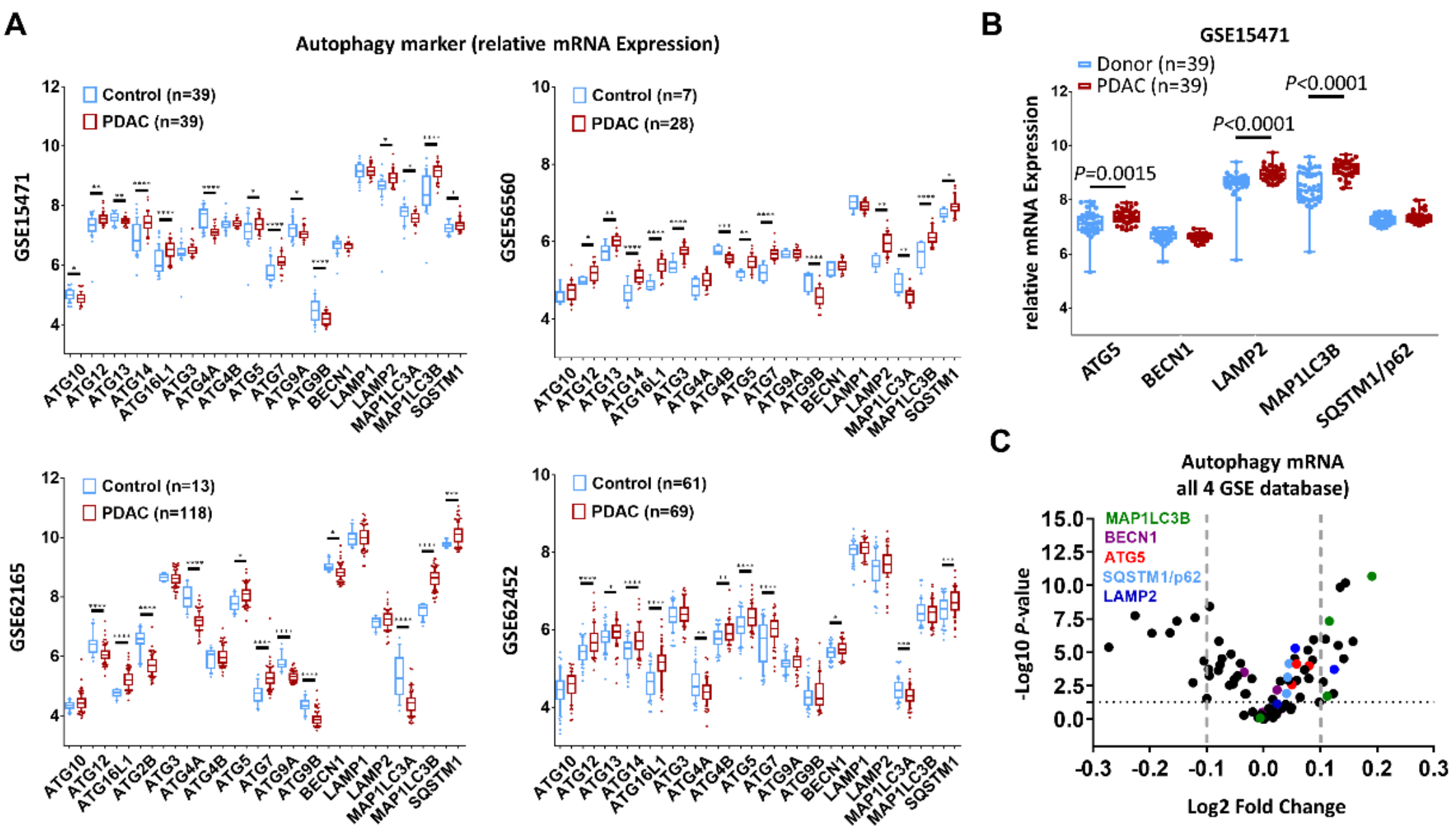

Supple. Figure 7. Meta-analysis of autophagy-related gene expression in PDAC vs. donor tissue. (A) mRNA expression data from four GEO (GSE) datasets comparing PDAC and donor controls Microarray expression data from four GEO (GSE) datasets comparing PDAC and donor controls (box-and-whisker plots, 10-90 percentile; *$P < 0.05$, ** $P < 0.01$, *** $P < 0.001$, **** $P < 0.0001$). **(B)** mRNA expression of ATG5, BECN1, LAMP2, LC3, and p62 in PDAC vs. controls in the GSE15471

dataset box-and-whisker plots, min-max all points). Statistical significance was assessed using the nonparametric Mann-Whitney test and presented as box-and-whisker plots; min-max with all patients/points shown. Significance levels (*P*-values) are displayed above the respective bars/plots. **(C)** Volcano plot showing differential enrichment of selected autophagy genes in PDAC vs. donor samples (Log2 fold change > 1 and -Log10 (*P*-values) >2).

Supple. Table 1A

The list of primary antibodies for IF			
**Primary Antibodies**	**Catalog No.**	**Dilution Ratio**	**Source**
LAMP2	PA1-655 (ThermoFisher)	direct labbeled Alexa 647	Rabbit
LC3B	NB600-1384 (Biotechne)	direct labbeled Alexa 555	Rabbit
LPS	ab35654 (Abcam)	anti-Mouse Alexa 488	Mouse
TLR4	ab13556	anti-Rabbit Alexa 555	Rabbit
αAmylase	Santa Cruz Biot. sc-46657	anti-Mouse Alexa 647	Mouse
Cytokeratin-19	Abcam ab52625	anti-Rabbit Alexa 555	Rabbit

The list of primary antibodies for WB				
**Antibodies**	**Catalog No.**	**Dilution Ratio**	**Molecular Wt.**	**Source**
ATG5	Abgent AP-1812A	1/1000	32 kDa	Rabbit
BECLIN1	Santa Cruz Biot. sc-48381	1/200	60 kDa	Mouse
LAMP2	Sigma-Aldrich L0668	1/1000	110 kDa	Rabbit
LC3B	Cell Signaling cs2775s	1/1000	14/16 kDa	Rabbit
SQSTM1, p62	Cell Signaling cs5114	1/1000	62 kDa	Rabbit
LPS	Abcam ab-35654	1/100	50 kDa	Mouse
GAPDH	Cell Signaling cs-2118	1/1000	37 kDa	Rabbit

Supple. Table 1B

Gene	Primer Sequence	Reference
16S rRNA	Forward: 5´-AGAGTTTGATCMTGGCTCAG-3	(Yang, Chen et al., 2015)
	Reverse: 5``-TGCTGCCTCCCGTAGGAGT-3’	

Supple. Table 1. (A) Antibodies used for immunofluorescence (IF) and western blot (WB) experiments. **(B)** 16S rRNA primer sequences and references for PCR-based 16S expression analysis.
