# Supplementary material for: Autophagy and intratumoral bacteria abundance influence the prognosis of patients with pancreatic carcinoma
Source: Cell Death Dis. 2026 Oct 27;17(1):916. doi: 10.1038/s41419-026-09256-1 (PMC13720179; doi:10.1038/s41419-026-09256-1)
Supplement: Supplementary file 2 [file 41419_2026_9256_MOESM3_ESM.pdf]

Source Data Figure 1C	
16S FISH	
Kras/B6	KrasCre
7,097195	84,24721
20,0572	86,68729
3,113899	80,48389
25,16269	81,03748
13,3339	

Source Data Figure 1D	
16S rRNA qPCR	
Kras/B6	KrasCre
0,0389	0,079
0,0405	0,0578
0,0183	0,0617
0,0199	0,0534
0,0237	0,0449

Source Data Figure 1E	
LPS<br>EU/g protein	
Kras/B6	KrasCre
32,7675	65,9635
35,083	53,8315
22,0815	49,077
29,3835	64,1605
32,4115	67,603
	63,5045
	79,4075
	41,699
	38,256
	42,683

Source Data Figure 2C

LPS	Kras/B6		LPS	KrasCre
-	4.240.832	1	-	14.929.551
+	26.906.028	2	+	58.090.780
-	2.471.711	3	-	21.554.927
+	22.441.007	4	+	50.998.839
-	6.966.317	5	-	24.295.362
+	23.156.179	6	+	78.149.065
-	2.283.790	7	-	24.613.978
+	55.400.212	8	+	60.284.002
-	7.260.217	9	-	9.440.823
+	27.839.756	10	+	33.538.362
-	6.266.217	11	-	24.263.543
+	37.063.969	12	+	51.299.082

GAPDH

LPS	Kras/B6		LPS	KrasCre
-	31.165.936	1	-	12.641.610
+	39.044.087	2	+	19.867.752
-	32.415.773	3	-	11.327.368
+	37.758.744	4	+	21.718.288
-	29.466.480	5	-	11.879.246
+	45.352.886	6	+	18.112.317
-	30.369.530	7	-	13.738.489
+	30.297.652	8	+	25.444.317
-	29.274.288	9	-	15.271.711
+	37.004.602	10	+	18.697.075
-	24.170.702	11	-	13.732.761
+	40.699.279	12	+	24.125.045

KrasCre PBS	Kras/6 LPS	KrasCre LPS
1,18098494	0,68911915	2,92387
1,90290692	0,5943261	2,3482
2,04519395	0,51057785	4,31469
1,79160736	1,82853153	2,36925
0,61819026	0,75233226	1,79378
1,76683647	0,91067876	2,12638

Source Data Figure 2E

TLR4 tissue IF

Mouse	Treatment	Sample ID	TLR4 %
Kras-B6	PBS	583 1968-1316	0,3270
Kras-B6	PBS	584 1968-1317	0,3579
Kras-B6	PBS	585 1968-1319	0,7468
Kras-B6	PBS	586 1968-1320	1,2046
Kras-B6	PBS	587 1968-1531	0,3968
Kras-B6	PBS	588 1968-1545	0,1886
KrasCre	PBS	559 1970-680	15,8100
KrasCre	PBS	560 1970-681	9,5900
KrasCre	PBS	561 1970-654	13,8800
KrasCre	PBS	562 1970-662	41,4000
KrasCre	PBS	563 1970-666	25,2000
KrasCre	PBS	564 1970-621	28,6200
Kras-B6	LPS	591 1968-1231	8,8868
Kras-B6	LPS	592 1968-1233	11,9841
Kras-B6	LPS	593 1968-1238	17,9371
Kras-B6	LPS	594 1968-1312	6,2289
Kras-B6	LPS	595 2605-429	13,1988
Kras-B6	LPS	596 2605-430	24,4693
KrasCre	LPS	551 1970-663	35,0600
KrasCre	LPS	552 1970-668	34,7800
KrasCre	LPS	553 1970-642	44,7800
KrasCre	LPS	554 1970-601	34,9500
KrasCre	LPS	555 1970-606	36,6300
KrasCre	LPS	556 1970-588	36,8700

TLR4 tissue IF

Kras/6 PBS	KrasCre PBS	Kras/6 LPS	KrasCre LPS
0,327031	15,81	8,886822	35,06
0,357921	9,59	11,98406	34,78
0,746759	13,88	17,93714	44,78
1,204643	41,4	6,22887	34,95
0,396849	25,2	13,19879	36,63
0,188587	28,62	24,4693	36,87

Source Data Figure 3A, B, C

Group	Richness	Shannon	inverse_Simpson
Donor	14	3,03281281	0,8196841
Donor	9	2,4007041	0,75549804
Donor	21	3,87887731	0,886234588
Donor	25	4,00802917	0,903969372
Donor	23	3,98501282	0,914863825
Donor	22	3,81056796	0,884724046
Donor	23	3,96130596	0,908403687
Donor	13	3,1434776	0,845762725
CP	26	3,1290194	0,77323454
CP	41	4,46765272	0,920593208
CP	27	3,96370062	0,905432985
CP	49	4,69408293	0,931260783
CP	49	4,64671987	0,918764986
CP	29	3,8828009	0,88647339
CP	39	4,52831543	0,936832861
CP	43	4,64627171	0,932495453
CP	31	4,18260852	0,91561778
CP	11	2,2915482	0,697829801
CP	50	4,74500432	0,929521554
CP	48	4,66352081	0,932169607
CP	23	3,6238419	0,878448143
CP	36	4,1817919	0,90649998
CP	47	4,68106098	0,927070998
CP	48	4,48682086	0,913883867
CP	21	3,52211895	0,87261361
CP	50	4,65749612	0,920908856
CP	23	3,37345998	0,809732621
CP	42	4,50977687	0,923874701
CP	39	4,43244426	0,924194368
CP	33	4,20213442	0,915084015
CP	17	3,40330696	0,861752383
CP	31	4,10721054	0,906811097
CP	33	4,49150132	0,942823914
CP	29	3,92544386	0,897104006
CP	43	4,33028014	0,910944273
CP	40	4,25553864	0,908618957
CP	10	2,31003226	0,711830225
CP	46	4,57648998	0,929906616
CP	39	4,34392216	0,912214228
CP	12	3,1313209	0,854486401
IPMN	28	3,91979513	0,902012931
IPMN	30	4,04702528	0,90789401
IPMN	46	4,55980093	0,928094068
IPMN	27	3,72223438	0,86891889
IPMN	10	2,42300432	0,738084173
IPMN	40	4,46437094	0,925235001

IPMN	32	4,12406122	0,902760749
IPMN	43	4,34523733	0,910022595
IPMN	27	3,81733515	0,881026857
IPMN	13	3,12208614	0,861957701
IPMN	37	4,25256725	0,910845339
IPMN	34	4,1360434	0,903602246
IPMN	38	4,31577215	0,916877627
IPMN	22	3,88419891	0,900652851
IPMN	19	3,51290201	0,870197791
IPMN	8	2,56037333	0,792040955
IPMN	20	3,7058566	0,886816398
IPMN	11	2,69404288	0,79535179
IPMN	19	3,399053	0,845533553
IPMN	10	2,25999058	0,724201653
IPMN	2	0,40430567	0,148214858
IPMN	22	3,62504004	0,860990727
IPMN	14	2,70330591	0,725483565
IPMN	7	2,26570081	0,741610821
IPMN	9	2,70750729	0,806950149

	**Richness**	**Shannon**	**inverse_Simpson**
**Mean CP**	34,53125	4,0746012	0,890907194
**Mean DONOR**	18,75	3,52759846	0,864892548
**Mean IPMN**	22,72	3,39886443	0,826215092
**SEM CP**	2,13457727	0,11719638	0,010769046
**SEM DONOR**	2,07665597	0,21102915	0,019430751
**SEM IPMN**	2,50633863	0,19201429	0,031180793
**KW**	0,0003426	0,00169916	0,004126026
**P_MW CP vs DONOR**	0,00164491	0,01562317	0,037573659
**P_MW CP vs IPMN**	0,00097292	0,00133524	0,00230769
**P_MW DONOR vs IPMN**	0,58464542	0,9832423	0,659149127

Source Data Figure 3D

HD ID	Group	Akkermansia	Anaerobiospi	Anaerostipes	Bacteroides	Bariatricus
2024	Donor	1,05896779	0	0	1,1035559	0
2084	Donor	3,19818685	0	0	1,10803324	0
2105	Donor	0,72388533	0	0,3778047	1,40162658	0
2150	Donor	1,68027211	0	0	0,62585034	0
3051	Donor	0,70716811	0	0	0	0
5602	Donor	0,61006739	0	0	0,59985706	0
5731	Donor	1,80963364	0	0	1,47254502	0
6168	Donor	2,47054043	0	0	2,67370987	0
4914	CP	1,84165817	0	0	1,70574244	0
4917	CP	2,18602328	0	0	3,33474202	0
5217	CP	1,37483292	0	0	1,4957673	0
5502	CP	1,31245533	0,40283282	0,15593529	2,69313235	0,27938406
5646	CP	1,16540096	0,27463342	0,47523523	2,34990686	0,08119597
5703	CP	1,72384844	0	0	1,28169251	0
5820	CP	2,16983401	0	0	1,50589367	0,07216743
5960	CP	1,79048043	0	0	3,09905101	0
6086	CP	2,32616308	0,48149075	0,27513757	1,71335668	0
6278	CP	0	0	0	5,39708266	0
6373	CP	1,3916059	0,09336653	0	2,4831051	0
6389	CP	1,85128983	0,12139605	0,13353566	3,15326252	0,16084977
6493	CP	2,54535025	0	0	1,26909013	0
6800	CP	2,22680412	0,26804124	0	3,02061856	0
7129	CP	1,64604477	0,12234117	0,45043793	2,03809259	0
7671	CP	1,51581893	0,04923173	0,18656233	1,7464307	0
7733	CP	2,24106204	0	0	1,39984227	0
8044	CP	1,35423384	0,30507466	0	2,85480431	0
8068	CP	0	0,43681747	0	4,06656266	0
8069	CP	1,24536301	0	0	1,86091068	0
8073	CP	1,4498124	0	0	2,76992913	0
8108	CP	2,72669258	0	0,11602947	2,09433196	0
8221	CP	1,5847348	0	0	1,30983182	0
8279	CP	1,46200196	0	0	1,77495446	0
8295	CP	0,78912186	0,31630091	0	1,31085532	0
8390	CP	2,94138099	0,38940268	0	2,9831027	0
8393	CP	1,42240918	0	0	2,37424691	0,05703896
8405	CP	2,27330519	0,60011189	0	2,89884555	0
8409	CP	0	0,45580335	0	4,65570568	0
8469	CP	1,59778598	0	0,29520295	2,82656827	0,03690037
8513	CP	1,53398276	0	0	1,84749748	0,17915127
8560	CP	1,74808038	0,53912759	0	0	0
4973	IPMN	3,63067836	0,052115	0	4,42977504	0
5045	IPMN	1,83038788	0,35574937	0	2,6394308	0,26394308
5948	IPMN	1,29385087	0,19563381	0	2,53434707	0
6106	IPMN	2,0926883	0	0	0,71168347	0
6163	IPMN	0	0	0	1,09907121	0
6392	IPMN	1,43345822	0	0	2,1762502	0

6457	IPMN	1,60004169	0	0	2,5225413	0
6715	IPMN	1,91195175	0,14770443	0,38977557	0,48003939	0,25437985
6956	IPMN	1,93852602	0	0	1,22249389	0
7089	IPMN	3,32351015	0	0	3,0124427	0
7269	IPMN	1,87568289	0	0,30957873	2,94403302	0,16996479
7319	IPMN	1,52905199	0	0,08293163	2,25470378	0
7711	IPMN	1,58420984	0	0,24239276	2,15123577	0
8023	IPMN	1,4235882	0	0	2,05524654	0
8066	IPMN	1,81567212	0	0	4,00297303	0
8092	IPMN	4,05405405	0	0	3,04716481	0
8198	IPMN	0,95327886	0	0	1,08946155	0
8207	IPMN	1,21991793	0	0	0,16635245	0
8210	IPMN	0,48821024	0	0	1,53734289	0
8265	IPMN	5,1627907	0	0	0	0
8267	IPMN	0	0	0	0	0
8276	IPMN	0,42254251	0	0	0,96951472	0,18664455
8439	IPMN	1,17179742	0	0	0,63555114	0
8494	IPMN	1,51320852	0	0	1,69274173	0
8503	IPMN	1,28141942	0	0	0,54213898	0

Blautia	Citrobacter	Clostridium	Corynebacter	Cutibacteriur	Duncaniella	Enterobacter
0	0	0	0,67996879	1,27076134	0	0
0	0	0	0	0	0	0
0	0	0,582569	0,56814905	0,74695737	0	0
0	0,12244898	0,897959	0,53741497	1,42857143	0	0
0	0	0,455373	0,39376406	1,09557484	0,30536805	0
0	0	0,546253	0,89595671	0,66111905	0	0
0	0	0,691919	3,05153908	1,52576954	0,45240841	0,48789142
0	0	0	0,61763511	0	0	0
0	0,01359157	0,652396	0,53686714	1,94359497	0	0,3058104
0	0,09604672	0,603173	0,39187061	0	0,19977717	0,1959353
1,13932913	0	0	0,45827764	1,164789	0	0
0	0,00974596	1,104541	0,40608148	0,91936846	0,17217855	0,63998441
0	0,02149305	0,599417	0,16716817	0,49434016	0,21015427	0,5707599
0,87871495	0,01679073	1,108188	0	0	0,3190239	0,29663626
0	0,71205196	0	0,52441665	1,06326678	0	0,55809478
0	0,05189798	1,256673	0	1,33081257	0	0,16310795
0,93171586	0,04377189	1,738369	0,43146573	0	0	0,71910955
0	0,66450567	0	0	0	0	0,76175041
0,06002134	0	1,469412	0,36012805	0,81806865	0,175618	0,58465232
0	0	0,61912	0,55235205	0,73444613	0,90136571	0,16084977
1,39098014	0,00717	0	0,05019001	0	0	1,0109701
0	0,01030928	0,804124	1,08247423	2,1443299	0	0,69072165
0	0,01946337	0,728486	0,31419436	0,84526623	0,51994995	0,43097456
0,05700516	0,018138	0,342031	0	0,71774674	0,27466121	0,40421838
0	0	0,308885	0,50604627	0	0	0,82150368
0	0,02976338	0,935066	0,35716057	0,6523141	0,73168312	0,30011409
0	0	0,083203	1,32085283	0	0	0,29121165
0,00815295	0	0,03465	0,27108557	0,65019771	0	0
0	0	0,727222	0,44930289	1,10704525	0,2825513	0,33350317
0	0	0,690375	0,20885305	1,57800081	0,77739746	0
0,66300129	0	0,565977	0	1,45536869	0	0
0	0	0,85011	0,26157224	0	0	0,8547807
0,02608667	0	0,453256	0,27391007	0,63912349	0	0,29999674
0	0	1,008275	0,36158821	0,05562895	0,41026354	1,17516167
0	0,00712987	0,30302	0,12477274	0,96253253	0,1212078	0,35292859
1,0934242	0	0	0,21359915	0,3356558	0,28479886	0,82896811
0,3744099	0	0	0	0	0,87904932	0
0	0,04797048	0,380074	0,12546125	1,36900369	0,80442804	0,15498155
0,01492927	0	0,291121	0	0,97413504	0	0,26499459
0	0	0	0,91488319	1,47034798	0	0
0	0	0,138973	0	0,12160167	0,32137584	0,98149917
0	0	0,120496	0,25820519	1,4000459	0	0,23525361
0	0,03556978	0,578009	0,53799297	1,2982971	0,3690365	0,41794496
0	0,05083453	1,059053	0,51681776	1,08447005	0,14403118	0,16097602
0,20123839	0	1,362229	0,68111455	0	0	0
0	0,01954716	0,026063	0,43981104	0,62225118	0,19221372	0,47238964

0	0	1,334237	0,40652525	0	0	0,45864387
0	0	0,484142	0,11898412	1,15701801	0,05744061	0,67697862
0,76842473	0	0,61998	0	1,03038771	0	0,0523926
0	0	0	0,32743942	2,99607073	0	0,66306483
1,06227996	0,09105257	0,613087	0	1,16547287	0,12140342	0,2306665
0	0,00518323	1,218058	0	1,28544032	0	0,47167366
0	0	1,034498	0	1,27689045	0,68822231	0,17313769
0,25454888	0	0	0,94277364	0,98048459	0	0,55623645
0,80696539	0	0	2,09173922	0	0	0,89190911
0	0	0	0	0	0	1,11287758
0	0	0	1,01613241	1,53991201	0	0
0,07763114	0,12199179	0,443607	0,38815571	1,57480315	0	0
0,94525813	0	0	0	0,85177106	0	0,21813649
0	0	0	3,79844961	0	0	0,79069767
0	0	0	0	0	0	0
0,30848196	0	0,209975	0,13479884	0,39921195	0,13479884	0
0	0,21847071	0	0,19860973	0	0	0,45680238
0	0	0,74378	0	0	0	0
0	0	0	0	1,19927715	0	0

Enterocloster	Enterococcus	Escherichia	Extibacter	Faecalibacter	Flavonifracto	Flintibacter
0	1,24846728	8,1373314	0	0	0,92520343	0
0	2,71971796	10,2241249	0	0	0	0
0	0,82194151	4,80763685	0	0,44125281	0	0,53354098
0	2,15646259	6,56462585	0	0	0	0
0,02678667	1,29915354	2,40812172	0	0	0	0
0	0,86022054	3,35664693	0	0,28333674	0	0
0	2,89186552	6,83935066	0	0,53224519	0	0
0	2,62494921	6,99715563	0	0	0,84518488	0
0,06795787	2,47366633	13,9041794	0	0	0,53007136	0,36697248
0,16135848	1,81720389	6,7347958	0,24972146	0,16520035	1,05651389	0,59164778
0,63649672	2,12589905	5,12379861	0	0	0,42008784	0
0,03248652	1,67305568	4,96718862	0	0,15268664	0,58475733	0,0877136
0,09791279	1,0698763	5,44012991	0,10746525	0,08358409	0,78091417	0,19104934
0,34700845	2,06525998	5,64168579	0	0	0,4813343	0
0,08660091	3,47365889	5,75414963	0,22612461	0	1,43372624	1,38080346
0,66355279	2,12411032	5,52342823	0,15198695	0	0,27431791	0,35587189
0,4002001	2,31365683	6,39694847	0,16258129	0	1,85092546	0,6128064
0	0	10,5510535	0	0	0	0
0,07780544	1,70505068	5,23741775	0	0,40681131	0,62688956	0,44237951
0,03338392	2,07283763	5,5660091	0,15174507	0,31562974	1,00151745	0
0	2,05062021	8,0375708	0	0	2,31591023	0
0,07731959	2,46391753	7,60824742	0,61340206	0	0,48453608	0
0	1,77394689	5,80564438	0,13624357	0	0,57834005	0,3364382
0,05700516	1,52877465	5,17192237	0,16583318	0,30057264	0,52341098	0,09846345
0	3,10856993	6,01340694	0,446898	0	0	0
0,49109579	1,6865916	5,6972072	0	0,23314649	0,52085917	0,38444367
0,1976079	0,07280291	9,79719189	0	0	0,54082163	0
0,07133831	1,21886592	4,08462761	0	0,12840895	0,58701235	0,56866822
0	1,83889944	4,3957571	0	0,26402335	0,26402335	0,23159943
0	3,10958984	6,24238557	0	0	0,89342693	0,5047282
0	2,60349288	7,55174644	0	0	0	0
0,09808959	1,9524499	4,77369331	0,22420477	0,35499089	0,45308048	0,16815358
0,28043173	1,72498125	2,11954218	0	0	0,36521342	0
0,07648981	2,94833461	7,00924831	0	0	0	1,5437035
0,04634416	1,96784428	4,95169513	0	0,10694806	0,38501301	0,39214288
0	2,8530743	6,43848853	0,45771245	0,19325637	0,64079744	0,10679957
0	0	13,1857399	0	0	1,95344294	0
0,08856089	1,82287823	4,53874539	0,46125461	0,29889299	0,38376384	0,35793358
0	1,23166499	5,00877095	0,328444	0,26872691	0,56358004	0
0	3,08773076	6,51854272	0	0,58813919	1,89511518	0
0,052115	3,25718753	8,25154174	0,64275167	0	2,63180752	2,16277252
0	2,10006885	4,99196695	0,21803994	0	0	0,32132201
0,56467031	2,44097639	5,02867814	0,14227913	0,37348273	0,30678938	0
0	2,87215115	7,78615606	0	0,64390409	0,17792087	0
0	4,55108359	14,4891641	0	0	2,2755418	0
0,02606288	1,60612478	4,16354455	0	0	0,68740837	0,18895586

0,0416949	1,73555011	5,84249752	0,2345338	0	0,29707615	0,50555063
0,03282321	1,68629221	6,0107496	0	0,06564641	0,65236122	0,11077832
0,27069508	2,29654209	7,69297939	0	0	0,76842473	0,41914076
0	2,07924034	5,24721676	0	0	0	0
0,14568411	2,14277043	5,94269758	0,15782445	0,12747359	0,32171907	0,17603496
0,04146582	1,69491525	5,76374851	0,26952781	0	0,20214586	0,66345307
0	2,0213825	5,3239839	0	0	0,74449206	0,32896161
0	2,45121146	5,92061846	0	0	0,69765249	0
0	2,39966023	7,42195795	0	0	0	1,6351667
0	0	9,88341282	0	0	0	0
0	0,86947413	6,7043788	0	0,30379216	0	0,83804735
0	0	3,92591771	0	0	0,44360652	0
0	1,080295	6,24285863	0	0,50898515	0	0,15581178
0	0,27906977	11,4883721	2,10852713	0	0	0
0	0	17,0242537	0	0	0	1,49253731
0	0,64547905	2,57413936	0	0	0	0
0	1,6285998	8,14299901	0	0	0,67527309	0
0	1,15414209	6,18107207	0	0	0	0
0	1,0021357	3,63068835	0	0	0	0

Fusobacteriu	Klebsiella	Kluyvera	Lactobacillus	Lawsonella	Leclercia	Leptotrichia
2,2071118	0	0	0	0	0	0
1,10803324	0	0	0	0	0,4784689	0
0,69504528	0	0	0,44990483	0,22495241	0	0
1,46938776	0	0	0	0	0	0
0,77413479	0	0	0,07500268	0,36965606	0	0
0,89085154	0,06381458	0	0,24760057	0	0	0
1,81850439	0	0	0	0	0	0
1,9341731	0	0	0	0,16253555	0	0
0,39415562	0	0	0	0,24464832	0,06795787	0
1,74420838	0,05378616	0	0	0,1344654	0	0
1,55941697	0	0,08274457	0	0	0,21640889	0,08274457
0,89337925	0,05522708	0,10395686	0	0,22415697	0,06822169	0,14943798
0,5994173	0	0,143287	0,09552467	0,07880785	0,07641974	0
0	0	0,10074439	0,30223317	0	0,12313203	0
1,76569642	0,02405581	0,08660091	0	0,47630503	0,13471253	0
1,75711744	0	0	0,57829181	0,29655991	0	0,20759193
0,05002501	0	0,00625313	0	0,53151576	0	0
1,76661264	1,13452188	0	0	0	0,19448947	0
0,95367242	0,10670461	0,12004268	0,23786235	0,38235817	0,04446025	0
1,93323217	0,13050076	0,11229135	0,50075873	0,09408194	0,09104704	0
1,87137019	0	0	0,48756005	0	0	0
0,02061856	0	0,32474227	0	0,42268041	0,07731959	0,65979381
1,30682608	0	0,24468233	0,10565828	0,0583901	0,01112192	0,10009732
0,73588475	0	0,00518229	0,77216075	0,44308553	0,06218744	0
0,88065195	0	0	0,4008938	0	0	0,40746583
1,14340989	0	0,18354085	0,2033831	0,08432958	0	0
0	0	0	0,5200208	0	0,44721789	0
0,82752436	0	0,02242061	0	0,3057356	0,05095593	0,24051201
0,41687897	0,0787438	0,15285562	0,26402335	0	0,18064755	0
2,03051575	0	0,20885305	0,28427221	0,48732378	0,19144863	0
0,59831824	0	0	0	0	0	0
0,04670933	0,02335466	0,25223037	0	0	0	0
1,91410963	0,04565168	0	0,1434767	0,41738677	0	0
0	0	0,26423754	0,44503164	0	0	0
0,89123382	0,36005846	0,09981819	0,12833767	0	0,00712987	0
0,04068555	0,0864568	0	0,21359915	0	0,07628541	0,54416925
0	0	0,24418037	0	0	0	0
0,80811808	0	0,08487085	0	0,10701107	0,02214022	0
0,97040272	0	0,23513604	0,4329489	0,26499459	0,08584332	0
0	0	0	0	0	0	0
0	0	0,50377834	0,40823417	0	0	0
1,73858159	0	0	0,3327978	0,07459261	0	0,36148726
1,7251345	0,04446223	0,05335468	0,22675737	0,22675737	0,06669334	0
0,381259	0	0	0,38973142	0	0	0
0	0	0	0	0	0	0,55727554
1,23472878	0,13357224	0	0	0,27691806	0,04235217	0

1,37593162	0	0,21368635	0,34919477	0	0,16156773	0
1,59602839	0,06974931	0,09846962	0,50465679	0,49645099	0,0041029	0
1,92979392	0	0,25323088	0,62871114	0,14844569	0,34055187	0
0	0	0	0	0	0	0
0	0,07891223	0,18210514	0	0,35206993	0	0,15175428
1,3787384	0	0,23842845	0,22806199	0,29026072	0,02591614	0
0,7488205	0,11686794	0,16015236	0	0,32896161	0	0,1125395
1,66870934	0	0	0	0,18855473	0	0
0	0,3716288	0	0	0	0,26544914	0
0	0	0	11,5792263	0	0	0
2,75508066	0	0	0	0,24093861	0	0
0,5101475	0	0	0	0	0	0
0,91409577	0	0	0	0	0	0,58169731
0	0	0	0	1,76744186	0	0
0	0	0	0	0	0	0
0,51068022	0	0,05443799	0	0	0	0
0,75471698	0	0,5163853	0	0	0	0
0	0	0	0	0	0	0
0,32856908	0	0	0	0	0	0

Ligilactobacil	Limosilactob	Mediterraneil	Microvirga	Morganella	Muribaculum	Parabacteroic
4,99386913	0	0	0	0	0	0,06688218
7,98287585	0	0	0	0	0	0
0,99498183	0,68639326	0	0,47009286	0	0	0
2,97959184	0	0	0	0	0	0
0,81163613	0,37501339	0,11250402	0	0,35090539	0	0,44465874
0,91127221	0	0	0	0	0	0
3,4063692	0,55885745	0	0	0	0	0
2,28362454	0	0	0	0	0	1,25152377
3,13965341	0	0	0,67278287	0,00679579	0	0,1019368
2,19754889	0	0	1,16024434	0,21514465	0	0,8221599
2,05588441	0	0,4073579	0,31824836	0	0	0,0381898
1,85498018	0,41907608	0	0,76668183	0	0	1,13702813
1,61914314	0,15522759	0	0,92897741	0	0,09552467	0,1003009
2,31712095	0	0	0,57648178	0,01679073	0	0,35820227
1,87154198	0	0	1,92927592	1,68871783	0	0,99591051
1,5754745	0	0,23354093	1,51616251	0	0,56346382	0
2,3074037	0	0	0,8004002	0	0	0,9192096
0	0	0	0	0	0	0
1,6894896	0,27120754	0,19784812	1,05815401	0	0	0,92921928
2,42792109	0,29438543	0,33687405	0,72837633	0,47040971	0,02427921	1,14112291
2,60271026	0,23661002	0	2,66007027	0,13623001	0,59511006	0
2,1185567	0,7371134	0,14948454	1,53092784	0	0,72680412	0
1,9630196	0	0,55331572	1,05658279	0	0,56999861	0,71458362
1,57800637	0,39385381	0,11141917	0,27207006	0,20470033	0	0,47417926
2,74710831	0	0	2,97712934	0,01314406	0	0
2,01150851	0,51341832	0	0,4216479	0	0	0,14385634
0	0,75923037	0,21840874	1,72646906	0,15600624	0	0
1,7854959	0,13860014	0,23032082	0,55847703	0	0,17528841	0,75210958
1,50539627	0,17601556	0	0,3242392	0	0,38908704	0,89397378
2,00150838	0	0	0,53953704	0	0	0,88762546
5,02910737	0,98641656	0	0	0	0	0
1,53673689	0,17749545	0	1,05563081	0,08874772	0	1,53206595
1,16085695	0	0	0,49238595	0	0	0,21195422
3,00396356	0,42417078	0	0,32682011	0	0	0
1,66482478	0,2424156	0,22815586	0,78428577	0	0	0,82350005
2,40044754	0	0,23394192	0,60011189	0,32039872	0	0,65605452
5,9091649	0	0	0	0	0	0
1,7195572	0,16236162	0	1,17712177	0	0,1402214	0,16605166
1,38095771	0,21274213	0	0	0	0,39935804	0,26126227
4,26400915	0	0	0	0	0	0
3,30930253	0,29531834	0	0,80778251	0	0,2345175	0
1,93940785	0	0	0,37296305	0	0	0
1,84518252	0,14672536	0	0,60468632	0	0,30678938	1,19603397
2,49089215	0	0	0	0	0	0,33889689
6,84210526	0	0	0,51083591	0	0	0
1,73643916	0,35184884	0	0,48216322	0,48867894	0	0,30298094

1,99614322	0	0	0	0,12508469	0,09902538	0
1,90374595	0,35284946	0	0,44721618	0	0	0,81647725
3,44044708	0	0	0,0261963	0	0	0
2,840537	0	0	0,25376555	0,01637197	0	0
2,47055967	0	0	0,42491198	0	0	1,23224475
1,94889338	0	0	0,70491888	0	0	0,14513036
1,95212743	0	0	1,04748301	0	0	0,05626975
3,3939851	0,91449043	0	0	0,33939851	0	0
0,04247186	0,7114037	0	0	0	0	0
0	1,48383678	0	0	0	0	0
3,0902996	0,58663314	0	0	0,16760947	0	1,64466792
2,43983587	0	0	0	0	0	0
2,87732419	0	0	0	0	0,27007375	1,45424327
0,09302326	0	0	0	0,04651163	0	0
0	0	0	2,89179104	0	0	0
0,87100788	0	0	0	0,12183741	0	0
0,07944389	0	0	0	0	0	0
5,64247243	0	0	0	0	0	0
3,63068835	0	0	0	0	0	0,52571053

Paramuribaci	Parasutterella	Peptacetobac	Peptoniphilus	Phocaeicola	Porphyromor	Prevotellama
0	0	0,39014603	0	0	0	0
0,8562075	0	0	0	0	0	0
0	0	0	0	0,45567284	0	0
0,47619048	0	0	0,95238095	0,72789116	1,45578231	0,91156463
0	0	0	0,31072538	0	0	0
0	0	0,42372881	0,30120482	0	0	0
0	0	0	1,4193205	0,51450368	0	0
0	0	0	0,31694433	0	0,32507111	0
0,42813456	0,56405029	0	0	0	0	0
0,64927581	0	0,53786162	0,5148104	1,11414192	0,74916439	0
0,43281777	0	0	0,31824836	1,52122717	0	0
0,37034631	0	0,51653564	0,15593529	0	0	0,15268664
0,45851841	0,31284329	0,51822133	0,28896212	0,80479534	0,27702154	0
0	0	0	0,58207869	0	0,17910114	0
0	0,29348088	0,47149387	0,91412076	1,31825836	0	0,21169112
0,20759193	0	0,48932384	0,48561684	1,04908066	0,56717082	0,21871293
0,2813907	0	0	0,76288144	0,86918459	0	0
0	0	0	0,72933549	0	0	0
0,13338076	0	0,40903432	0	0,41348035	0,44460253	0,29121465
0,46433991	0	0,53414264	0	0,95902883	0,4370258	0
0	0,20793002	0	1,15437012	0	0,33699003	0
0,21134021	0	0	0,24742268	0	0	0,3814433
0	0	0,48102322	0,66453496	0,83136383	0,36146253	0
0	0	0	0,48713497	0,72552017	0,25911435	0,27466121
0	0	0	0	0	0	0
0,28275212	0,28275212	0,23314649	0,19346198	0,7887296	0,448931	0,12649437
0	0	0,96723869	0,4576183	1,80967239	0,4576183	0
0	0	0,39949452	0,26904733	0,28331499	0,27720028	0,18140312
0,03705591	0	0	0	0,99587753	0,21770346	0,09263977
0	0,57434588	0,21465452	0	0,03480884	0	0
0	0	0	0	2,23156533	0	0
0,48110608	0	0,5698538	0,28025597	0	0	0,30828156
0,39456093	0,46303845	0,42716927	0	0,74673101	0,07173835	0
0,17384048	0,02781448	0,3963563	0,23642306	0	0	0
0,1818117	0,26380521	0	0,29232469	0,72011693	0	0
0,02034278	0	0	0,40176982	0	0	0
0	0	0	0	0	0	0
0	0	0,43542435	0,27675277	0,90774908	0	0
0,46280745	0,14556041	0	0,69794349	0,86589781	0,13436345	0,11196954
0,68616239	0	0	0	0	0	0
0	0	0,72092417	0,62538001	0	0	0
0,21230204	0	0	0,27541887	0,94675235	0	0
0,44906852	0,24454226	0,15117158	0	0,16451025	0,19118759	0
0,27111751	0	0,75404558	0,46598322	1,4064221	0,20333813	0
0	0	0	1,08359133	0	0	0
0,18895586	0	0,14986154	0,29972308	0,51474181	0	0

0,12508469	0	0	0,77135561	0,5107625	0	0
0,13949863	0,27899725	0,40208427	0,45952488	1,13240061	0	0
0,28815927	0	0,41040866	0	1,25742228	0	0
0,6794368	0	0	0	0	0	0
0	0	0,38242078	0	0,58273643	0	0
0,48722335	0	0,54423884	0,2487949	0,80858342	0,21251231	0
0	0,51075618	0	0,09522573	0,7488205	0,26403497	0
1,2444612	0,14141605	0	0,65994155	0	0	0
0	0	0,59460607	0	0	0	0
0	0	0	1,45733969	0	0	0
0	0	0,80662057	0	1,47705845	0	0
0	0	0	0	0	0	0
0	0	0	0	0	0,31162356	0
0	0	0	0	0	0,10852713	0
0	0	0	0	0	0	0
0	0	0	0,09072999	0,20738283	0	0
0	0	0	0,2979146	0	0	0
0	0	0	0	0	0	0
0	0	0	0	0	0	0

Raoultella	Romboutsia	[Ruminococc	Salmonella	Sangeribacte	Segatella	Staphylococc
0	0	0	0	0	1,17043808	0
0	0	0,15109544	0	0	0	0
0	0	0	0	0	0,57680106	0
0	0	0	0,55782313	1,04761905	0,7414966	0,47619048
0	0	0	0	0	0,1875067	0,27590271
0	0	0	0	0	0,2578109	0,20675924
0,79836778	0	0	0	0	0	0,80723853
0	0	0	0	0	0	0
0	0	0	0	0,36017669	0	0
0,46486611	0	0,06531177	0,08067924	0,4533405	0,45718237	0
0,08910954	0	0,12729934	0	0	0,28005856	0
0,1884218	0,29887597	0	0,09096225	0,07471899	0	0,47755182
0,08836032	0	0,08836032	0	0,42747289	0,20537804	0,32717199
0	0,5876756	0,59886942	0,1063413	0	0	0,374993
0,61101756	0,58215059	0,0384893	0	0	0	0
0,44483986	0,34475089	0,011121	0	0,48932384	0,11120996	0
0,55652826	0	0	0,20635318	0	0	0,26263132
0,84278768	0	0	0	0	0	0
0	0	0	0	0,30899876	0,24230838	0,11559666
0,07587253	0	0	0	0	0	0,16388467
0,19359002	0,78870008	0	0,19359002	0	0	0
0,18041237	0	0,06185567	0,06185567	0	0	0
0,45877937	0	0,07229251	0	0,23634089	0	0,39482831
0,12178374	0	0	0	0,22542948	0,01554686	0,28243464
0,39432177	0,06572029	0,08543638	0	0	0	0
0,18106057	0	0	0,06944789	0,02976338	0	0,33235776
0,21840874	0	0	0	0	0	0
0,25274143	0,30369736	0,04076475	0	0,30165913	0,26904733	0,35057682
0	0,5651026	0,06484784	0	0	0	0
1,21250798	0	0,05221326	0,07541916	0,20885305	0	0
0,4851229	0	0	0,11319534	0	0	0
0	0	0	0	0	0,14946985	0,20085011
0,02282584	0	0	0	0	0	0,89672938
0,75099089	0,70926917	0,0347681	0	0	0	0
0,23528573	0	0,04990909	0	0	0	0
0,24411331	0,13731374	0,21359915	0	0,04068555	0	0,87473936
0,35813121	0	0	0	0	0	0
0,1402214	0	0	0,04797048	0	0,20664207	0,44649446
0,2873885	0	0	0	0	0	0
0	0	0	0	0	0,60447639	0
0,38217667	0,81646834	0	0	0	0	0
0,02868947	0	0	0	0	0	0
0	0	0	0	0,22231115	0,19118759	0
0,42362111	0	0	0,27958993	0	0	0
0	0	0	0	0	0	0
0,26062877	0,39420101	0,0162893	0,11728295	0	0	0,24433947

0,32834732	0,27101683	0	0	0,31792359	0,27101683	0
0	0,22155664	0	0	0	0	0,32823206
0,13971359	0	0	0	0	0	0,23576668
0,72855272	0	0	0	0,33562541	0	0
0,17603496	0	0	0,22459633	0,18210514	0	0,30350856
0,12439745	0	0	0	0	0,1917794	0
0,10388261	0,63195256	0	0	0	0,1125395	0,41120201
0	0	0	0	0,5185255	0	0,82021307
0	0	0	0	0	0	0
0	0	0	0	0	0	0
1,07898596	0	0	0	0	0	0
0	0	0	0	0	0	0
0	0	0	0	0,44666043	0	0
0	0	0	0	0	0	0
0	0	0	0	0	0	0
0	0	0	0	0	0,11146827	0
0	0	0	0	0	0,93346574	0
0	0	0	0	0,35906643	0	0
0	0	0	0	0	0	0

Trueperella	Turicimonas	Zhenhengia
0	0	0,81373314
0	0	0
0	0,63736517	0,5998731
0	0,89115646	1,06122449
0	0,63216543	0,52769742
0,29865224	0	0,29865224
0	0,70966025	1,15319791
0	0	0
0	0,03397893	1,29119946
0	1,11414192	0,81447616
0,28642352	0,09547451	0,84017567
0	0,35410305	0,63673575
0,22687109	0,67822515	0,62329847
0	0	0,89550568
0	0,60139524	0,91893192
0	0,36328588	0,65243179
0	0,9192096	1,11930965
0	0	0
0,35345901	0,31789081	0,68468789
0	0	0,72837633
0	0,04302	1,31211013
1,11340206	0,6185567	1,12886598
0	0,35868205	0,63116919
0,28243464	0,23579406	0,5545047
1,04495268	0,38117771	0,92008412
0	0,1761	0,43156903
0	0	1,39365575
0	0,40560923	0,47694754
0	0	0,6438464
0	0	0,95724314
0	0,63065977	1,2774903
0	0	1,20510066
0	0,32608341	0,30651841
0	0,50066059	1,15430081
0,3636234	0	0,40640262
0,0813711	0,39159843	0,98153893
0	0	0
0,46125461	0,17712177	0,81549815
0	0,3694995	0,68674654
0	0	1,78075478
0	0	1,73716668
0,41886619	0,17787468	0,63690613
0	0,58690143	0,74696545
0	0	0,94891129
0	0	0
0,23456589	0,64179834	0,42677961

0	0,44300829	0,7036014
0,21745374	0,43080458	0,79596275
0	0,50646175	0,74222843
0	0	0
0	0,64950832	0,99550807
0	0	0,85004924
0	0,90464442	0,72284985
0	0	0
0	0	2,29348057
0	0	4,02755697
0	0,34569453	0
0	0	0
0	0	0,34278591
0	0	0
0	0	0
0	0	0
0	0,83416087	0
0	0	0
0	0	0,64070971

Source Data Figure 3E

Matches	ANOSIM	ANOSIM P value	PERMANOVA	PERMANOVA P value
**('CP', 'DONOR')**	0,079213383	0,087491251	5,664725975	0,00029997
**('CP', 'IPMN')**	0,111981784	0,0079992	5,468267315	0,0019998
**('DONOR', 'IPMN')**	-0,092957317	0,893610639	1,282698894	0,254274573

Source Data Figure 3F

Donor	EU/g	CP	EU/g	IPMN	EU/g
HD804	15,24	HD4914	51,5	HD4973	20,8
HD2024	8,20	HD4917	35,6	HD5045	20,1
HD2084	19,01	HD5217	47	HD5948	44,9
HD2105	0,00	HD5502	82	HD6106	70,5
HD2106	32,66	HD5646	67	HD6163	23,1
HD2150	30,92	HD5703	87,7	HD6392	33,4
HD3051	18,88	HD5820	68,1	HD6457	58,4
HD5602	0,19	HD5960	23,3	HD6715	35,5
HD5731	0,00	HD6278	19,8	HD6956	53,2
HD6168	41,16	HD6373	88	HD7089	18,1
		HD6389	34,4	HD7269	45,6
		HD6493	49,2	HD7319	35
		HD6800	21,6	HD7711	65,2
		HD7129	40,6	HD8023	0
		HD7621	32,3	HD8066	34,8
		HD7671	5	HD8092	37,6
		HD7733	46	HD8198	3,6
		HD8044	13	HD8207	86,7
		HD8068	17,4	HD8210	13,6
		HD8069	63,8	HD8267	37,7
		HD8073	25,8	HD8276	12,2
		HD6086	38,8	HD8439	21,8
		HD8108	0,5	HD8494	55,8
		HD8279	55,6	HD8503	33,3
		HD8295	32,3		
		HD8390	13,5		
		HD8393	37,3		
		HD8405	88		
		HD8409	11,3		
		HD8469	39		
		HD8513	14,6		
		HD8221	0,2		
		HD8560	0		

Source Data Figure 4A, 4B, 4C

sample	Group	Richness	Shannon	inverse_Simpson
2024	Donor	14	3,03281281	0,8196841
2084	Donor	9	2,4007041	0,75549804
2105	Donor	21	3,87887731	0,886234588
2150	Donor	25	4,00802917	0,903969372
3051	Donor	23	3,98501282	0,914863825
5602	Donor	22	3,81056796	0,884724046
5731	Donor	23	3,96130596	0,908403687
6168	Donor	13	3,1434776	0,845762725
7927	Adjuvant	26	3,53106935	0,864465999
7963	Adjuvant	46	4,42994045	0,908355533
7977	Adjuvant	45	4,56828837	0,919765452
8001	Adjuvant	31	4,07841519	0,906792242
8030	Adjuvant	44	4,4586529	0,922846037
8035	Adjuvant	27	3,98149849	0,906644344
8048	Adjuvant	33	4,25984214	0,915007979
8099	Adjuvant	47	4,60753947	0,93109154
8118	Adjuvant	40	4,35382875	0,914032358
8145	Adjuvant	49	4,71045655	0,933039856
8197	Adjuvant	48	4,57906884	0,921092623
8209	Adjuvant	20	3,69298277	0,887812131
8214	Adjuvant	45	4,53138531	0,930764547
8215	Adjuvant	50	4,46565837	0,913369696
8216	Adjuvant	51	4,52366027	0,904252349
8219	Adjuvant	6	2,10105594	0,700737598
8225	Adjuvant	28	4,25445993	0,918762659
8231	Adjuvant	48	4,61729153	0,925188735
8247	Adjuvant	53	4,71144085	0,93355972
8257	Adjuvant	55	4,73181416	0,921844301
8268	Adjuvant	48	4,64342018	0,926813735
8272	Adjuvant	53	4,51275978	0,911377908
8293	Adjuvant	49	4,58813464	0,920860072
8366	Adjuvant	30	3,92946913	0,886389031
8376	Adjuvant	54	4,82666079	0,93336347
8401	Adjuvant	57	4,69347168	0,920660261
8417	Adjuvant	49	4,62495034	0,925141866
8453	Adjuvant	52	4,64888812	0,924622326
8455	Adjuvant	50	4,72631116	0,930776076
8465	Adjuvant	48	4,51888522	0,923794351
8571	Adjuvant	44	4,52576501	0,930715444
8640	Adjuvant	31	4,07067843	0,91037187
7968	Neoadjuvant	24	3,91419876	0,899533657
8029	Neoadjuvant	50	4,58005559	0,921659994
8046	Neoadjuvant	44	4,30799723	0,910587515
8059	Neoadjuvant	54	4,70497223	0,926429277
8061	Neoadjuvant	47	4,4338652	0,923776873
8085	Neoadjuvant	51	4,71234152	0,928726374

8217	Neoadjuvant	57	4,82731282	0,934788063
8222	Neoadjuvant	48	4,71684674	0,938021564
8260	Neoadjuvant	32	3,89329006	0,889303043
8273	Neoadjuvant	47	4,55684029	0,918264813
8359	Neoadjuvant	42	4,51124811	0,922977534
8381	Neoadjuvant	57	4,88228853	0,935454987
8575	Neoadjuvant	41	4,42335202	0,921178696
8644	Neoadjuvant	36	4,25530674	0,912339785

**Mean Adjuvant**	42,40625	4,3593045	0,910134753
**Mean DONOR**	18,75	3,52759846	0,864892548
**Mean Neoadjuvant**	45	4,47999399	0,920217298
**SEM Adjuvant**	2,09879928	0,09124085	0,007266022
**SEM DONOR**	2,07665597	0,21102915	0,019430751
**SEM Neoadjuvant**	2,52895322	0,08153118	0,00365991
**KW**	0,00017208	0,00072444	0,001388981
**P_MW Adjuvant vs DO**	0,00010655	0,00041022	0,001102718
**P_MW Adjuvant vs Ne**	0,69325008	0,64155992	0,383559884
**P_MW DONOR vs Neo**	0,00019694	0,00072885	0,001186993

Source Data Figure 4D

HD ID	Group	Akkermansia	Anaerobiospi	Anaerostipes	Bacteroides	Bariatricus
2024	Donor	1,05896779	0	0	1,1035559	0
2084	Donor	3,19818685	0	0	1,10803324	0
2105	Donor	0,72388533	0	0,3778047	1,40162658	0
2150	Donor	1,68027211	0	0	0,62585034	0
3051	Donor	0,70716811	0	0	0	0
5602	Donor	0,61006739	0	0	0,59985706	0
5731	Donor	1,80963364	0	0	1,47254502	0
6168	Donor	2,47054043	0	0	2,67370987	0
7927	Adjuvant	2,41028388	0	0	2,0085699	0
7963	Adjuvant	1,17620851	0	0,13588773	2,39251504	0,03341501
7977	Adjuvant	0,970453	0,09987186	0	1,89944976	0
8001	Adjuvant	1,13554061	0	0	2,96689084	0
8030	Adjuvant	2,0750137	0,36019106	0	3,21039856	0
8035	Adjuvant	1,47212362	0	0	2,33869284	0
8048	Adjuvant	1,74271763	0,3746422	0,19784476	2,84138744	0
8099	Adjuvant	1,51557589	0	0	2,28425606	0
8118	Adjuvant	1,51016184	0	0	2,46989085	0,07056831
8145	Adjuvant	1,35890517	0	0	2,66640966	0,16383963
8197	Adjuvant	0,46898336	0,14174142	0	0,50798604	0,0684925
8209	Adjuvant	0,0401714	0	0	3,08650241	0
8214	Adjuvant	1,86801534	0	0,02447182	2,50020393	0
8215	Adjuvant	1,6888434	0	0,26375876	2,73206834	0,1850248
8216	Adjuvant	1,10179886	0	0,19215045	2,92109567	0,05519215
8219	Adjuvant	0,06357455	0	0	0,05942838	0
8225	Adjuvant	0,71307448	0,17392061	0	0,8326449	0
8231	Adjuvant	1,41124476	0	0,30384451	2,09426713	0
8247	Adjuvant	1,26928501	0,09188427	0,24787384	2,2372751	0,07051583
8257	Adjuvant	1,09111406	0,0946169	0	1,94870556	0,22144381
8268	Adjuvant	0,89296636	0	0,26736566	1,9781564	0
8272	Adjuvant	0,77036024	0	0,07039846	2,11195366	0,22929783
8293	Adjuvant	0,81405084	0	0,04275103	1,80088708	0,06590784
8366	Adjuvant	1,61733657	0	0,16731068	0,0637374	0
8376	Adjuvant	0,92049701	0,12911262	0	1,63137588	0,05316402
8401	Adjuvant	0,77491137	0,13592478	0,00560515	1,04255707	0,18076594
8417	Adjuvant	0,51942343	0,09139076	0,09370445	0,66865644	0
8453	Adjuvant	0,88394639	0	0,12915346	2,16541707	0
8455	Adjuvant	1,02337233	0,32513392	0,34645417	2,03075447	0
8465	Adjuvant	1,31311371	0,07553309	0,11039452	1,6210563	0,25565046
8571	Adjuvant	0,57385416	0	0,09584983	0,99459756	0
8640	Adjuvant	1,63030448	0,26372573	0	1,79333493	0
7968	Neoadjuvant	1,90967088	0	0,23237146	1,67729942	0
8029	Neoadjuvant	1,41951488	0	0,29768467	2,42557883	0,08820287
8046	Neoadjuvant	1,55605105	0,14232174	0,28464348	2,98875658	0
8059	Neoadjuvant	1,27821002	0,29720195	0,16672305	2,38486445	0
8061	Neoadjuvant	1,57783393	0	0	2,84127637	0
8085	Neoadjuvant	1,21832142	0,23745062	0,25520394	1,58670277	0

8217	Neoadjuvant	1,34030729	0,1004063	0,39462009	1,92406482	0
8222	Neoadjuvant	1,61988035	0,27611597	0,21782482	3,04034361	0
8260	Neoadjuvant	1,57900631	0	0,35462317	1,27291052	0
8273	Neoadjuvant	1,17337165	0,25034831	0,24599443	1,97884013	0
8359	Neoadjuvant	1,26965494	0,49426956	0	2,97797411	0
8381	Neoadjuvant	0,90505293	0	0	1,4594396	0
8575	Neoadjuvant	1,00647977	0,15587076	0	1,00425305	0,22044579
8644	Neoadjuvant	1,66297935	0,23230088	0	2,37463127	0

Blautia	Citrobacter	Clostridium	Corynebacter	Cutibacteriun	Duncaniella	Enterobacter
0	0	0	0,67996879	1,27076134	0	0
0	0	0	0	0	0	0
0	0	0,582569	0,56814905	0,74695737	0	0
0	0,12244898	0,897959	0,53741497	1,42857143	0	0
0	0	0,455373	0,39376406	1,09557484	0,30536805	0
0	0	0,546253	0,89595671	0,66111905	0	0
0	0	0,691919	3,05153908	1,52576954	0,45240841	0,48789142
0	0	0	0,61763511	0	0	0
0	0	0,117836	0	0,06963042	0	0,21960364
0	0,04678102	0,76409	0	0,96012475	0	0,07796837
0	0	0,776362	0,10740936	1,10235924	0,22800935	0,26381247
0	0	0,589757	0	0,02845318	0	0,19399897
0	0,0195756	0,070472	0	1,2215175	0,21141649	0,61075875
0	0,02088119	1,174567	0	0,93443308	0,08352474	0,3027772
0	0,06735141	1,41438	0	0	0,17258798	0
0	0,01867239	0,936732	0	1,12034357	0,1618274	0,31743068
1,24670681	0	0,870342	0,16936394	0	0,26815958	0,45163718
0	0,00963763	0,648933	0	0,69712156	0	0,44333076
0	0,01522056	0,019026	0,14174142	0,43759097	0,16837739	0,19025694
1,56668452	0	1,111409	0	0	0	0
0	0,02039318	0,342605	0	1,1746472	0,40378497	0,43641406
0,08660735	0,04330368	0,88182	0	0,755846	0,60625148	0,20470829
0	0,00613246	0,570319	0,24938675	0,92804579	0	0,59893704
0	0	0	0	0	0	0
0	0	0,341319	0,55002391	0,65872429	0,28044698	0
0,00502222	0,01506667	0,667956	0,2787334	0,98686689	0,46455566	0,73324461
0	0,0149579	0,386769	0,0982948	0,71370571	0,02136843	0,63036882
0	0,01811813	0,549584	0,23754882	0,79317148	0,41269074	0,4247695
0	0,02096986	0,712975	0,01572739	0,72870249	0,40192224	0,32328528
0	0,0643643	0,56721	0,13476276	0,78242855	0,16694491	0,13878553
0	0	0,388322	0,18525446	0,63770284	0	0,10509628
0,10357328	0	1,083536	0	0,11950763	0,386408	0,1593435
0,01822766	0	0,381263	0	0,6015129	0	0,21417505
0,00560515	0,00420386	0,539496	0	0,57172484	0,10649776	0,16254922
0	0,00231369	0,490502	0	0,26723121	0,02892113	0,05668541
0	0	0,028514	0	0,33210889	0,35055939	0,27675741
0,01066013	0	0,812835	0,34645417	0,11193135	0,4477254	0,24251792
0	0,01452559	0	0,15106618	0,60716983	0	0,26727093
0,28754948	0,00248961	0,043568	0	0,40954017	0,07344337	0,07842259
0	0,01918005	1,318629	0,16303045	1,1268281	0	0,17262047
0,92103595	0	1,390004	0	0	0,26617094	0,36334446
0	0,01653804	0,441014	0	0,66703418	0,10198456	0,1323043
0	0,03795246	0,839698	0	0,28938754	0	0,16129797
0	0,01449766	0,654811	0,05799062	0,83119896	0,08940221	0,47117383
0	0,04407357	0,48481	0	0,70811541	0,00293824	0,10283834
0,00443833	0,00887666	0,630242	0,05991745	0,79002264	0,29071058	0,53481869

0	0,04203054	0,354925	0,30822398	0,73319946	0,18213235	0,33857937
0	0,02147569	0,696426	0,36201871	1,42046326	1,0615125	0,12885412
0	0	0,496472	0	0	0	0,30236291
0	0	0,254702	0	0,64219784	0,3853187	0,19810171
0	0,02162429	0,657996	0	0,99162831	0,16372679	0,25640234
0	0,01669839	0,769796	0,11521892	0,77981498	0,4074408	0,3773837
0	0	0,015587	0	0,7882607	0,06902848	0,25161994
0	0	0,955015	0,41666667	1,08775811	0,17330383	0,10693215

Enterocloster	Enterococcus	Escherichia	Extibacter	Faecalibacteri	Flavonifractoı	Flintibacter
0	1,24846728	8,1373314	0	0	0,92520343	0
0	2,71971796	10,2241249	0	0	0	0
0	0,82194151	4,80763685	0	0,44125281	0	0,53354098
0	2,15646259	6,56462585	0	0	0	0
0,02678667	1,29915354	2,40812172	0	0	0	0
0	0,86022054	3,35664693	0	0,28333674	0	0
0	2,89186552	6,83935066	0	0,53224519	0	0
0	2,62494921	6,99715563	0	0	0,84518488	0
0,0374933	2,55490091	6,64167113	0	0	0,87305838	0
0,02227668	1,06928046	5,388728	0	0	0,28068612	0,38984184
0,05841562	1,50749981	5,06143062	0,26758122	0,06783749	0,45601869	0,57661868
0,01551992	1,57268495	4,48784273	0,37506467	0	0,2250388	0,53026384
0,0587268	1,49557591	5,55947068	0	0	0	0,03132096
0,10962623	1,72791815	4,87053665	0	0	0	0
0,05051355	1,99107594	5,0345176	0	0,56827749	0,43778414	0,63141943
0,04356892	1,48134317	4,68677061	0	0,12759468	0,51971493	0,30187035
0,25404592	2,08882198	6,29469326	0	0,18818216	0,5833647	0
0,06746338	1,500257	4,93767669	0	0,4690311	0,29555384	0,44654331
0,21499034	0,55459899	2,07570324	0,15696198	0	0,18264666	0,18454923
0	2,50401714	5,18880557	0	0	0,6293519	0,54231387
0,09380863	2,10049759	5,37564239	0,11828045	0,33444816	0,79941268	0,87690676
0,09841745	1,6770333	6,90496811	0	0,22832848	0,23620187	0,44091016
0,053148	1,33483238	7,19746525	0,26369583	0,38021259	0,84832379	0,46606705
0	0	0,20316214	0	0	0	0
0	0,90873516	2,65011522	0	0	0	0,22174877
0,02008889	1,79544484	5,02975667	0,24106672	0,23855561	0,43693343	0,53235567
0,01923159	1,52570623	3,4766443	0,16026326	0,12821061	0,21795803	0,6047267
0,02013126	1,17566534	4,89793453	0,19124693	0,20131256	0,20936506	0,17111567
0,03494976	1,30362604	3,79903888	0	0,32328528	0,31804281	0,09261686
0	1,46831064	4,96610817	0	0,19510429	0,40831104	0,3097532
0,01603164	1,3715955	3,9687205	0	0	0,28144427	0,66264094
0,02788511	1,88423694	4,87192766	0	0,10357328	0,56566944	0
0,03949327	0,58784215	3,32502962	0	0,10480907	0,28404776	0,7853085
0,0210193	1,07899051	3,12486863	0,09388619	0,09809005	0,20318653	0,18356852
0,01619583	0,74038083	2,2905532	0,04858749	0,18278152	0,23599639	0,33664191
0,03522367	1,11541623	4,05910867	0	0,04696489	0,51158188	0,39752428
0,12259148	1,45777257	4,66913626	0,18388722	0,60762732	0,51168617	0,30647869
0,02324095	1,78374296	4,09331242	0	0	0,55197258	0,38057057
0	0,55642692	1,62695745	0,05477133	0,03734409	0,20788209	0,00622401
0	1,61591944	4,70390794	0,4363462	0,1438504	1,13162311	0
0	1,62237526	5,40791753	0	0	0,77738814	0,51544214
0,03307607	1,25413451	4,53142227	0	0,20948181	0,49062845	0,18467475
0	1,93557569	5,22795199	0,25617914	0	0	0,03320841
0,01691393	1,47876093	4,86879621	0,12806263	0,27545547	0	0,12806263
0,0381971	1,34571311	4,46906035	0	0,05582653	0,63465946	0,52006817
0,07101327	0,88766588	4,50712352	0	0,2640806	0,39279215	0

0,07939102	1,66954654	4,91056835	0,11208145	0,11208145	0,16111708	0,45533087
0,10124252	1,54011351	5,12041724	0	0,18714527	0,78846449	0
0,08958901	1,58273918	4,46451902	0,45167793	0	0,69431483	0,76150659
0,06095437	1,27133403	4,57810867	0	0,23728666	0,51811216	0,13061651
0	1,80099472	5,18056285	0	0,2224213	0,39541565	0,85879336
0,02003807	1,29579534	3,71038306	0	0,1653141	0,34732659	0,16698394
0	0,86619609	2,78563317	0	0,20263199	0,21153889	0,11356298
0,04056047	1,2979351	4,80088496	0	0,18436578	0,41666667	0

Fusobacteriur	Klebsiella	Kluyvera	Lactobacillus	Lawsonella	Leclercia	Leptotrichia
2,2071118	0	0	0	0	0	0
1,10803324	0	0	0	0	0,4784689	0
0,69504528	0	0	0,44990483	0,22495241	0	0
1,46938776	0	0	0	0	0	0
0,77413479	0	0	0,07500268	0,36965606	0	0
0,89085154	0,06381458	0	0,24760057	0	0	0
1,81850439	0	0	0	0	0	0
1,9341731	0	0	0	0,16253555	0	0
0,73915372	0,24638457	0,10176754	0	0	0,21424746	0
0,89775006	0	0,02895968	0,32301181	0	0	0
0,5671968	0,05653124	0	0,14132811	0,03957187	0	0,27134997
0	0	0,39834454	0,29746508	0,10346611	0,02327988	0
1,82836113	0,0587268	0,19967113	0,18401065	0	0,0195756	0,19184089
1,56086866	0	0	0,22969305	0	0	0
0	0,2946624	0	0	0,1810069	0,05893248	0
1,52802415	0	0,40456851	0,15560327	0	0,00622413	0,10581023
1,30316146	0	0	0,37636432	0,45634174	0	0
1,51631971	0,00642508	0	0,10280134	0,16062709	0,03533796	0,44011822
0,46137308	0	0,0808592	0,0313924	0,01902569	0,00380514	0,08466434
0	0	0	0,20755222	0	0	0
1,45607309	0,00815727	0,07341545	0,15498817	0,24063953	0,31405498	0
1,43295803	0,05511377	0	0,24801197	0,16927801	0,04724037	0
0,75429272	0	0,17988553	0,20645953	0	0,09811938	0
0	0	0	0	0	0	0
0,68698639	0	0	0	0	0	0,27392495
1,42631143	0	0	0,29882229	0,2159556	0	0,17326671
1,03209539	0	0	0	0	0,12821061	0
0,63212143	0,02214438	0,00402625	0,28989008	0,35028385	0,12481379	0
0,84578419	0,1013543	0	0,12232416	0	0	0,23765837
0,80656516	0,01609108	0	0	0,20918398	0,026148	0,11263753
0,8496767	0	0,09618981	0,17991058	0	0,00356259	0
0,65330837	0	0	0,16731068	0,15137633	0	0
0,87492785	0,01822766	0,09873318	0,23999757	0,22480785	0,03949327	0,09417626
0,99911719	0,05324888	0,06866303	0,23121225	0,05745274	0,01121029	0,02522315
0,54603086	0	0,07403808	0	0	0,02082321	0
1,45087975	0,03857831	0,10231638	0,14760395	0	0	0,38410574
0,94342137	0,01066013	0	0,23452283	0,0746209	0,0079951	0
1,05455813	0,01452559	0,00871536	0,30213236	0,25274534	0,06391261	0,20335832
0,47924913	0	0,01867204	0,03983369	0	0	0
1,26108847	0	0,03356509	0,25893071	0	0	0
1,5674511	0	0	0	0,13942287	0	0
1,2954796	0	0,00551268	0,1323043	0,20948181	0,0137817	0
1,30461597	0,00948812	0,04744058	0,18976232	0	0,2372029	0,14232174
0,78045716	0,24646016	0,07007201	0,23679505	0	0,10631615	0
1,31926897	0,02056767	0,07051772	0,27913263	0	0,06464124	0
1,10958235	0,08654742	0,188629	0,34618969	0,17309485	0,08654742	0,12427322

1,66020642	0,00934012	0,14477187	0,09106617	0,24284313	0,18680241	0,15411199
0,91425065	0,05522319	0	0,28225188	0,22396073	0,06749501	0,09203866
0,54873269	0,05972601	0,1978424	0	0	0,03732875	0
0,8794845	0,19810171	0,11537792	0,10449321	0,28517938	0,01088471	0
1,18933613	0	0,14519168	0,29347255	0	0,08340799	0
0,88167518	0,11688876	0,12189827	0,24379655	0,21039976	0,00667936	0,10019036
0,87064954	0,02449398	0,12915006	0,19817854	0	0,01336035	0
1,19469027	0	0,22123894	0,32079646	0	0	0,2359882

Ligilactobacill	Limosilactoba	Mediterraneil	Microvirga	Morganella	Muribaculum	Parabacteroic
4,99386913	0	0	0	0	0	0,06688218
7,98287585	0	0	0	0	0	0
0,99498183	0,68639326	0	0,47009286	0	0	0
2,97959184	0	0	0	0	0	0
0,81163613	0,37501339	0,11250402	0	0,35090539	0	0,44465874
0,91127221	0	0	0	0	0	0
3,4063692	0,55885745	0	0	0	0	0
2,28362454	0	0	0	0	0	1,25152377
2,69416176	0,32672737	0	2,61381896	0	0,27852169	0
1,74203609	0,11806638	0,21831143	0,75295166	0,05791936	0	0,89552239
1,73362478	0,31845934	0,11306249	1,42270295	0	0,49559056	0,80839677
1,65804449	0	0	0,92602173	0	0	0
1,97322058	0,50896563	0,13702921	1,85576697	0	0,097878	0,77127868
1,62873251	0	0	0,66297766	0,13572771	0	0
2,05421788	0	0	1,01027109	0,36201381	0	0
1,51557589	0,14004295	0	1,41287773	0,11825849	0,31743068	1,06743846
2,22054949	0,11761385	0	0,03763643	0	0	1,07263831
1,97892573	0	0	1,48098175	0,18311488	0	1,17579029
0,89230506	0,08846948	0,10083618	1,65143026	0	0	0,15981583
2,28976968	0	0	0,6293519	0	0	0,38162828
2,08826169	0	0	1,00742312	0	0	1,01965903
2,22423431	0,29918904	0	1,26368003	0	0,20864499	1,26368003
2,34464432	0	0,14309076	1,80907604	0,25347506	0,37612428	0,8217498
0,23633147	0	0	1,65846647	0	0	0
1,03265359	0,20870473	0	0	0	0	0,34131919
1,62468925	0,26868895	0,08286669	2,00386711	0	0	0
1,42313774	0,05128424	0	1,28424292	0	0	0,41027394
1,49172605	0,12884004	0	1,5823167	0	0,22748319	0,88980151
1,21275666	0,16251638	0	0,74792486	0	0	0,61861075
1,42406018	0,22125229	0	0,30170767	0,16091075	0,0905123	0,69392763
1,14002743	0,25116229	0,07125171	0,88708385	0,05878266	0,21197385	0,72854878
1,44205872	0,50591563	0,21909732	2,44990639	0	0	0
0,96302822	0,13366953	0,10329009	0,5741714	0	0,09873318	0,44202084
1,03555064	0	0,07426818	1,28497961	0,06866303	0	0,21860068
0,83986951	0	0	1,27021587	0	0,05321487	0,39564101
1,37707778	0	0,11238028	1,26302018	0,10902565	0	0,75479294
1,5190683	0,56765184	0	1,73760094	0	0,2078725	0,02398529
1,45546453	0,20626344	0,24402998	0,93544826	0,09877404	0,00581024	0,22369415
0,57634376	0,04481291	0,10331864	1,25974058	0	0,04854731	0,41700899
1,71661472	0	0	0,68568689	0	0	0
1,58435084	0,34221978	0	1,82094723	0	0	0
1,41124587	0,08820287	0,15435502	0,3693495	0	0,1185226	0,84068357
2,1158499	0,05218464	0,16129797	0,10911334	0	0,11860145	0
1,6841444	0,27303919	0,24887643	0,50258542	0,18605325	0,16189049	0,96167786
1,7982018	0,18804725	0,32614444	0,94905095	0,02644414	0,17629429	0
1,39363544	0,14424571	0	0,98974746	0,1131774	0,3018064	0,82109094

1,88903937	0,25685331	0,16812217	1,22355578	0	0,32223416	0,44832578
1,50329805	0,62893082	0,07363092	1,46648259	0,09203866	0,5890474	0,46019328
1,70592407	0,22397253	0	0,35089029	0	0	0,38448617
1,44113549	0,31565657	0	0,94914664	0,17415535	0	0
1,49825461	0,34598869	0	0,71978005	0,27493744	0	0,44175342
1,08539558	0,05677454	0,23711719	1,50786494	0	0,26550446	0,75810707
1,0621479	0,19149836	0,08461556	1,71457837	0	0	0
1,65560472	0,44985251	0	1,03244838	0,25073746	0,11061947	0,2359882

Paramuribacu	Parasutterella	Peptacetobac	Peptoniphilus	Phocaeicola	Porphyromon	Prevotellamas
0	0	0,39014603	0	0	0	0
0,8562075	0	0	0	0	0	0
0	0	0	0	0,45567284	0	0
0,47619048	0	0	0,95238095	0,72789116	1,45578231	0,91156463
0	0	0	0,31072538	0	0	0
0	0	0,42372881	0,30120482	0	0	0
0	0	0	1,4193205	0,51450368	0	0
0	0	0	0,31694433	0	0,32507111	0
0	0	0,05891805	0,57311194	0,01071237	0	0
0,11806638	0	0	0,45889953	0,49899755	0,49231455	0,16039207
0	0,19974373	0,34860933	0,50689681	0,66518429	0,50689681	0,16582498
0	0	0,71391619	0,81738231	0	0,12415934	0,4009312
0,12528385	0,28580377	0	0,52854123	0,7360426	0,24665257	0
0,26623512	0	0	0,71518062	0,72562122	0	0
0	0	0,25677724	0,37043273	0	0,3746422	0
0,31120655	0,57262005	0,22406871	0,19917219	0,85893007	0,2707497	0
0,28227324	0,14584117	0,20700038	0	1,34550245	0,1740685	0
0,39514264	0,56540735	0,06103829	0,5846826	0,62644564	0,62965819	0,26985351
0,07134635	0,11700802	0,15981583	0	0,21308778	0,06183351	0,16837739
0	0,49544724	0	0	0	0	0
0	0,59548087	0	0,77086222	1,31332083	0,40786361	0,06525818
0,21651838	0	0	0,3818597	1,16919928	0,5629478	0,26769546
0,14717907	0	0	0,57645135	0,56623058	0,4946852	0,23303352
0	0	0	0	0	0	0
0,15000652	0,29349102	0	0	0,26740293	0	0
0	0	0,43693343	0	0,83368907	0,31640007	0
0,11538955	0,48292662	0,33121074	0,38249498	0,42950553	0,22650541	0,13248429
0	0,26371945	0,19124693	0,23956194	0,58783267	0,57978017	0,20332568
0,31629533	0,08038445	0	0,32153779	0,52075142	0,20795107	0
0,31981013	0,07442122	0,17297906	0,52698272	0,72812117	0,34595812	0,17901321
0	0,20484868	0,38654055	0,38654055	0,48451166	0	0,09618981
0	0	0,64135761	0,38242441	0	0,386408	0,19917938
0,19746635	0,11240393	0,2491114	0,25214935	0,5255643	0,51037458	0
0,1541415	0	0,02242058	0,29006628	0,33070359	0,18076594	0,11910934
0,10874343	0,17121307	0,10874343	0,23136901	0,3516809	0	0,06709701
0	0,10063906	0,36733256	0,2952079	0,69944145	0,47971284	0,05367416
0,52501133	0	0,17589212	0,47171068	0,66092796	0,32246889	0
0,31665795	0,26146069	0	0,12201499	0,76985649	0,36313985	0,13363547
0,07966739	0	0	0,16306919	0,26016382	0,26265342	0
0,34524095	0,08151522	0	0,39798609	0,03356509	0,31167586	0
0	0	0,60416579	0,36334446	0,06759897	0	0
0,15711136	0,12127894	0,47684675	0,2646086	0,44101433	0,42723264	0
0,25143508	0	0,26566725	0,74007306	0,83969828	0,77802552	0,13283363
0,14739284	0	0,16189049	0,31169961	0,76837578	0,42284831	0
0,26737968	0	0,25562673	0,52006817	0,95198919	0,54651231	0
0,33065554	0	0,44383294	0,34618969	0,74342018	0	0,23523146

0,88497642	0,48568627	0,50670154	0,42030542	0,65847849	0,1938075	0,37593985
0,2945237	0	0,47553306	0,60131922	1,04310477	0,57370762	0,3558828
0,01866438	0	0,07092463	0,29863003	0	0	0,08958901
0,22640195	0	0,37225705	0,55294323	0,46804249	0,18068617	0
0,14828087	0	0,23786723	0,18535109	0,73522597	0	0
0,32895835	0,18201249	0,22876799	0,43081856	0,47590422	0,55438667	0,26383462
0,24271304	0	0,17591129	0,23603287	0,59453562	0,12246988	0,14251041
0	0,14749263	0	0,44985251	0,09955752	0,39454277	0

Raoultella	Romboutsia	[Ruminococcu	Salmonella	Sangeribacter	Segatella	Staphylococcu
0	0	0	0	0	1,17043808	0
0	0	0,15109544	0	0	0	0
0	0	0	0	0	0,57680106	0
0	0	0	0,55782313	1,04761905	0,7414966	0,47619048
0	0	0	0	0	0,1875067	0,27590271
0	0	0	0	0	0,2578109	0,20675924
0,79836778	0	0	0	0	0	0,80723853
0	0	0	0	0	0	0
0,41778254	0	0,06427424	0	0	0	0
0,21831143	0,33860548	0,04009802	0	0,21831143	0	0,10024504
0,13190623	0	0	0	0,30526871	0	0,26758122
0,29487843	0,27935851	0	0,06983963	0	0	0
0,3092945	0,49330514	0,01566048	0	0,53637147	0	0
0,21925245	0	0	0,22447275	0,37064105	0	0
0,13049335	0,6693046	0	0,13470281	0	0	0
0,28631002	0	0	0	0,06224131	0,37033579	0
0	0	0,39988709	0	0,29168235	0	0,38577343
0,28912876	0	0	0,077101	0,32767926	0	0,23451555
0,0313924	0,0494668	0	0,0561258	0	0,09607976	0,07610278
0,39501875	0,42849491	0,47536154	0	0	0	0
0	0	0	0,02855045	0	0	0,28958316
0,10629084	0	0,28344225	0,03149358	0,49602394	0	0
0,26573998	0	0,00613246	0	0,13082584	0	0,31888798
0	0,1271491	0	0	0	0	0
0,33044915	0	0	0	0	0,23696682	0,4587156
0,30635562	0	0	0,0853778	0	0	0
0,18376854	0,19872644	0,03632634	0	0	0,1132527	0
0,30196884	0	0	0,06240689	0,23352257	0,16910255	0,28183758
0,29357798	0,19571865	0,02271734	0	0,10659677	0	0,15727392
0	0,15688799	0	0,01005692	0,1287286	0,09855784	0
0	0,22622419	0	0,05165749	0,09262723	0	0,14428472
0	0,22308091	0,06772099	0	0	0	0
0,05316402	0,10784701	0	0	0,18531458	0,11696084	0,21417505
0,03643345	0,14012864	0,00700643	0	0,02942701	0,18917366	0,12751706
0,18625205	0,21980056	0	0,01850952	0	0,08907707	0,07403808
0,13250809	0	0,04528758	0	0,27843473	0	0,16605445
0,21320257	0	0,03731045	0	0,40508488	0	0,12792154
0,03486143	0	0	0,14235082	0,20916855	0,37185521	0,37476033
0,05477133	0	0,08962581	0,00995842	0,07095377	0,0435681	0
0,20618557	0	0	0	0,30208583	0	0
0	0	0	0	0	0	0
0,29768467	0	0	0	0,12679162	0,23153252	0
0,34631624	0	0	0,04269652	0	0	0,43170928
0,09906732	0,2416276	0	0,02416276	0,2319625	0,09423477	0,25129271
0,02056767	0	0,01175295	0,04113534	0,37903273	0	0
0	0	0,00887666	0	0,17531401	0	0

0,21248774	0,61644795	0	0,0700509	0	0,05137066	0,14944193
0,13499003	0,54916398	0,03374751	0,06442706	0	0	0,18100936
0,11571914	0	0	0	0	0,29489716	0
0,22640195	0,48981191	0,01306165	0	0,34613375	0,18504006	0
0,12974576	0,32127522	0	0,13901331	0	0	0
0,08349197	0,12857763	0,08015229	0	0,17032361	0,07681261	0,10353004
0,11578971	0,43643813	0	0	0,09797591	0	0,05121468
0,23967552	0	0,02949853	0	0	0	0

Trueperella	Turicimonas	Zhenhengia
0	0	0,81373314
0	0	0
0	0,63736517	0,5998731
0	0,89115646	1,06122449
0	0,63216543	0,52769742
0,29865224	0	0,29865224
0	0,70966025	1,15319791
0	0	0
0,05891805	0	1,17836101
0	0,3029628	0,6059256
0	0,34860933	0,38441245
0	0,66994309	0,66994309
0,37976666	0,45415394	0,716467
0,48026728	0,34453957	0,88745041
0	0,44620306	0,95554807
0,03734479	0,31431861	0,75000778
0,48927362	0	0,8185924
0,20560267	0,51400668	0,52043177
0,01522056	0,17789024	0,23972375
0	0	0,92394215
0,33444816	0,79941268	0,93400767
0,03936698	0,98417447	1,00385796
0,31275552	0,21054783	0,64390842
0	0	0
0	0	0,2587069
0,18582226	0,45451121	0,36411119
0	0	0,51711612
0	0,43886138	0,50126827
0,01747488	0	0,36871997
0	0,35400366	0,46060704
0,20128609	0,27075652	0,33310177
0,40632594	0,28283472	0,84452058
0,26126318	0,30683234	0,4010086
0	0	0,34471645
0,13419403	0,20823211	0,28111335
0	0,10063906	0,65583119
0,34112411	0,47437572	0,46105056
0	0,10167916	0,6333159
0,00746882	0,20539249	0,28132547
0	0	0,97338768
0,1901221	0,33799485	1,1407326
0	0,38864388	0,53472988
0,02846435	0,09488116	0,62147161
0,38177161	0,5944039	0,57024114
0	0,56414174	0,82564494
0,27739559	0,42607962	0,56144867

0,17979732	0,4576659	0,60710783
0,75471698	0,22702869	0,62893082
0	0,04852738	0,76897234
0	0,1197318	0,50069662
0,19770782	0,87423929	0,66726391
0,20205056	0,30224092	0,37571386
0	0,09352246	0,26943374
0,02581121	0,31342183	0,60471976

Source Data Figure 4E

Matches	ANOSIM	ANOSIM Pvalue	PERMANOVA	PERMANOVA Pvalue
('Adjuvant', 'DONOR')	0,3252475	9,999E-05	10,20136795	9,999E-05
('Adjuvant', 'Neoadjuvant')	-0,00754441	0,541145885	1,572957669	0,159584042
('DONOR', 'Neoadjuvant')	0,66326531	0,00019998	13,03349779	9,999E-05

Source Data Figure 4F

Donor	EU/g protein	PDAC Adjuvant	EU/g protein	PDAC Neoadjuvant	EU/g protein
HD804	15,24	HD7823	3,56	HD7916	82,14
HD2024	8,20	HD7927	20,82	HD7968	15,92
HD2084	19,01	HD7963	20,90	HD8029	95,98
HD2105	0,00	HD7977	30,78	HD8046	14,45
HD2106	32,66	HD8001	17,63	HD8059	73,01
HD2150	30,92	HD8030	19,36	HD8061	22,10
HD3051	18,88	HD8035	2,62	HD8085	57,13
HD5602	0,19	HD8048	14,12	HD8217	67,43
HD5731	0,00	HD8099	68,20	HD8222	12,79
HD6168	41,16	HD8118	5,86	HD8260	16,87
		HD8145	31,55	HD8273	29,31
		HD8197	69,96	HD8359	31,80
		HD8214	47,17	HD8381	19,70
		HD8215	26,65	HD8575	57,30
		HD8216	57,91	HD8644	53,01
		HD8219	28,80		
		HD8225	48,03		
		HD8231	58,61		
		HD8247	79,37		
		HD8257	72,84		
		HD8268	35,15		
		HD8269	13,00		
		HD8271	26,82		
		HD8293	29,12		
		HD8366	25,11		
		HD8376	6,82		
		HD8401	125,33		
		HD8408	32,23		
		HD8417	19,10		
		HD8453	33,86		
		HD8455	54,55		
		HD8465	38,33		
		HD8476	40,65		
		HD8571	107,33		
		HD8640	50,09		

Source Data Figure 5B						
Tissue	HD ID	months	stat	Area (mm²)	total cells #	LPS cells counts
Donor	HD1527	N/A	N/A	14,16	186162	0
Donor	HD1590	N/A	N/A	18,51	192990	5
Donor	HD1611	N/A	N/A	39,10	344782	9
Donor	HD1663	N/A	N/A	22,28	303274	6
Donor	HD1715	N/A	N/A	61,94	558703	172
Donor	HD2024	N/A	N/A	41,64	351035	6
Donor	HD2105	N/A	N/A	45,36	355794	158
Donor	HD2106	N/A	N/A	37,10	309516	76
Donor	HD2150	N/A	N/A	9,66	71430	1
Adjuvant	HD3866	19,67	1	70,92	240013	813
Adjuvant	HD3868	18,10	1	7,53	27247	295
Adjuvant	HD3948	77,17	1	99,08	450376	1495
Adjuvant	HD4112	16,82	0	46,98	197456	36
Adjuvant	HD4127	22,47	1	38,66	237975	290
Adjuvant	HD4132	25,79	1	88,12	498651	789
Adjuvant	HD4337	13,86	1	82,76	283701	319
Adjuvant	HD4432	5,32	1	35,52	319550	1543
Adjuvant	HD4828	16,10	1	40,48	161109	467
Adjuvant	HD4952	34,43	1	30,13	175894	3318
Adjuvant	HD4954	6,64	1	27,91	178295	121
Adjuvant	HD5008	84,90	0	45,38	293300	487
Adjuvant	HD5024	20,14	1	43,28	142567	3968
Adjuvant	HD5031	14,75	1	44,67	254723	808
Adjuvant	HD5163	50,10	1	13,83	159730	17042
Adjuvant	HD5311	43,37	1	34,47	211042	815
Adjuvant	HD5321	24,77	0	46,65	186291	574
Adjuvant	HD5353	49,71	1	14,54	99580	266
Adjuvant	HD5362	18,30	1	79,13	339367	5009
Adjuvant	HD5398	33,08	1	50,92	227697	506
Adjuvant	HD5402	44,85	1	39,67	356253	149
Adjuvant	HD5457	88,18	1	15,12	134401	35
Adjuvant	HD5517	9,69	0	61,94	289928	242
Adjuvant	HD5521	38,87	1	53,83	174356	225
Adjuvant	HD5530	15,18	1	79,05	399618	52
Adjuvant	HD5534	16,89	1	24,36	97222	81
Adjuvant	HD5737	12,29	1	39,03	247583	282
Adjuvant	HD5743	40,34	1	49,02	289617	5981
Adjuvant	HD5795	38,64	0	33,35	288662	257
Adjuvant	HD5919	32,36	1	75,71	610238	423
Adjuvant	HD5950	15,61	1	33,09	225630	21512
Adjuvant	HD6190	15,54	1	22,92	216292	455
Adjuvant	HD6202	5,13	0	27,16	132760	422
Adjuvant	HD6221	46,52	1	20,09	111625	115
Adjuvant	HD6251	11,79	1	27,64	67065	618
Adjuvant	HD6253	17,91	0	20,93	56443	825
Adjuvant	HD6260	23,80	0	20,53	82638	124
djuvant/Blasto	HD6268	19,22	1	30,51	249910	2922

Adjuvant	HD6296	55,20	0	32,19	108906	75
Adjuvant	HD6351	11,53	0	33,27	240272	164
Adjuvant	HD6359	16,13	1	56,90	275212	884
Adjuvant	HD6391	53,85	0	30,88	192345	443
Adjuvant	HD6508	25,13	1	51,95	383689	258
Adjuvant	HD6574	7,69	1	38,15	148537	1114
Adjuvant	HD6726	19,61	0	101,32	521541	98
Adjuvant	HD6748	11,89	0	65,64	233849	376
Adjuvant	HD6771	5,95	1	46,09	209332	534
Adjuvant	HD6795	41,86	0	51,80	238701	1374
Adjuvant	HD6821	25,95	1	98,90	451196	764
Adjuvant	HD6987	17,48	1	38,11	171196	378
Adjuvant	HD7057	14,39	1	55,34	156375	404
Adjuvant	HD7059	52,24	1	29,35	129798	640
uvant/Cholan	HD7155	6,24	1	32,41	94652	413
Adjuvant	HD7289	11,53	1	65,28	374288	268
Adjuvant	HD7372	55,16	0	117,44	746077	405
Adjuvant	HD7399	55,75	0	33,59	182919	3920
Adjuvant	HD7451	37,36	1	33,69	134847	518
Adjuvant	HD7515	16,62	1	50,64	208721	59
Adjuvant	HD7559	7,03	1	85,57	307099	34
Adjuvant	HD7573	40,57	0	55,14	314221	619
Adjuvant	HD7603	43,14	1	54,07	269585	335
Adjuvant	HD7643	17,61	1	109,91	736059	832
Neoadjuvant	HD3530	3,88	1	82,94	346170	623
Neoadjuvant	HD3622	0,82	0	43,95	112481	626
Neoadjuvant	HD3739	14,46	1	25,19	205609	2130
Neoadjuvant	HD3855	24,11	1	58,18	149940	569
Neoadjuvant	HD3907	21,62	1	29,52	191066	2449
Neoadjuvant	HD4055	2,17	1	56,64	304873	1711
Neoadjuvant	HD4129	43,63	1	53,83	322162	12651
Neoadjuvant	HD4378	81,48	1	48,47	130240	129
Neoadjuvant	HD4593	1,05	1	66,94	190640	147
Neoadjuvant	HD4639	38,77	1	25,52	134404	97
Neoadjuvant	HD4686	20,96	1	17,30	110613	383
Neoadjuvant	HD4715	44,52	1	28,17	134272	322
Neoadjuvant	HD4761	6,70	1	30,64	133229	384
Neoadjuvant	HD4880	10,83	0	50,36	300696	215
Neoadjuvant	HD4961	10,78	1	17,75	61190	944
Neoadjuvant	HD5153	42,84	1	39,42	199528	126
Neoadjuvant	HD5179	12,75	1	40,62	234901	632
Neoadjuvant	HD5276	13,86	1	58,83	253588	310
Neoadjuvant	HD5308	36,50	1	8,62	27927	411
Neoadjuvant	HD5471	28,98	1	33,29	173525	1306
Neoadjuvant	HD5593	25,13	1	22,78	116354	334
Neoadjuvant	HD5603	91,47	0	8,99	35101	145
Neoadjuvant	HD5648	41,63	0	25,34	81180	500
Neoadjuvant	HD5764	20,01	1	41,25	157076	308
Neoadjuvant	HD5788	12,81	1	28,83	153077	111
Neoadjuvant	HD5794	5,26	1	29,70	98204	374

Neoadjuvant	HD5811	19,19	1	35,99	142752	974
Neoadjuvant	HD5850	7,79	1	62,81	219191	3198
Neoadjuvant	HD5870	6,60	1	57,39	211630	209
Neoadjuvant	HD5889	6,24	1	66,32	378708	5414
Neoadjuvant	HD6035	21,16	1	22,49	108041	13436
Neoadjuvant	HD6056	28,42	1	59,17	349015	3936
Neoadjuvant	HD6126	7,82	1	17,55	98259	4499
Neoadjuvant	HD6157	18,27	1	27,85	184658	2927
Neoadjuvant	HD6196	19,38	1	29,36	262483	8394
Neoadjuvant	HD6240	21,85	0	46,31	311871	908
Neoadjuvant	HD6292	36,24	0	45,13	213259	216
Neoadjuvant	HD6300	12,94	1	35,74	133323	986
Neoadjuvant	HD6321	17,58	1	37,74	214550	588
Neoadjuvant	HD6346	11,56	1	63,60	298301	1575
Neoadjuvant	HD6395	32,23	1	56,44	281773	494
Neoadjuvant	HD6399	18,00	1	6,32	9619	81
Neoadjuvant	HD6436	37,75	1	24,86	49827	18
Neoadjuvant	HD6448	5,91	1	26,24	151097	1222
Neoadjuvant	HD6474	69,19	1	45,04	229951	509
Neoadjuvant	HD6483	4,80	1	53,96	364007	420
Neoadjuvant	HD6520	10,22	1	60,26	403412	681
Neoadjuvant	HD6933	65,61	0	23,10	135416	186
Neoadjuvant	HD7809	20,27	1	13,62	19222	13

**LPS cells/mm2**	**LPS %**	**LPS dots**	**LPS dots /mm2**
0,00	0,00	1011	71,4
0,27	0,00	3204	173,1
0,23	0,00	7227	184,8
0,27	0,00	2317	104,0
2,78	0,03	13343	215,4
0,14	0,00	5467	131,3
3,48	0,04	6119	134,9
2,05	0,02	6488	174,9
0,10	0,00	1112	115,1
11,46	0,34	74068	1044,3
39,19	1,08	9817	1304,2
15,09	0,33	77839	785,6
0,77	0,02	20581	438,0
7,50	0,12	21062	544,9
8,95	0,16	60596	687,7
3,85	0,11	48364	584,4
43,45	0,48	52903	1489,6
11,54	0,29	59221	1463,0
110,12	1,89	52266	1734,6
4,34	0,07	11555	414,0
10,73	0,17	13611	300,0
91,69	2,78	54213	1252,7
18,09	0,32	28069	628,4
1232,56	10,67	115405	8346,6
23,64	0,39	22865	663,3
12,31	0,31	30953	663,6
18,29	0,27	27301	1877,7
63,30	1,48	55271	698,5
9,94	0,22	24994	490,9
3,76	0,04	20670	521,0
2,31	0,03	10459	691,7
3,91	0,08	21889	353,4
4,18	0,13	34487	640,6
0,66	0,01	84583	1070,1
3,32	0,08	14260	585,4
7,23	0,11	29851	764,8
122,00	2,07	85617	1746,4
7,71	0,09	30393	911,4
5,59	0,07	36652	484,1
650,03	9,53	231188	6985,8
19,85	0,21	30949	1350,4
15,54	0,32	47018	1731,1
5,72	0,10	31686	1577,2
22,36	0,92	30919	1118,8
39,41	1,46	32601	1557,4
6,04	0,15	18639	907,8
95,78	1,17	48609	1593,4

2,33	0,07	35376	1098,8
4,93	0,07	32564	978,7
15,54	0,32	125246	2201,0
14,35	0,23	34354	1112,6
4,97	0,07	174930	3367,4
29,20	0,75	39461	1034,3
0,97	0,02	13964	137,8
5,73	0,16	61414	935,7
11,59	0,26	65442	1419,8
26,52	0,58	136774	2640,3
7,73	0,17	53549	541,5
9,92	0,22	46473	1219,4
7,30	0,26	57397	1037,1
21,81	0,49	33550	1143,3
12,74	0,44	54006	1666,2
4,11	0,07	40221	616,1
3,45	0,05	56255	479,0
116,70	2,14	127430	3793,7
15,38	0,38	64388	1911,3
1,17	0,03	62260	1229,4
0,40	0,01	46734	546,1
11,23	0,20	82384	1494,2
6,20	0,12	56184	1039,1
7,57	0,11	85481	777,7
7,51	0,18	73081	881,2
14,24	0,56	42715	971,9
84,54	1,04	51613	2048,6
9,78	0,38	53489	919,4
82,97	1,28	45810	1552,0
30,21	0,56	105590	1864,2
235,02	3,93	160254	2977,1
2,66	0,10	26700	550,9
2,20	0,08	43096	643,8
3,80	0,07	15784	618,6
22,14	0,35	22814	1319,0
11,43	0,24	43551	1545,9
12,53	0,29	60580	1977,5
4,27	0,07	19219	381,6
53,19	1,54	45409	2558,6
3,20	0,06	69209	1755,6
15,56	0,27	38830	955,8
5,27	0,12	62478	1061,9
47,67	1,47	35932	4168,0
39,23	0,75	69940	2100,7
14,66	0,29	59274	2602,3
16,13	0,41	15520	1726,4
19,73	0,62	36743	1450,2
7,47	0,20	63505	1539,7
3,85	0,07	29618	1027,4
12,59	0,38	53404	1797,9

27,06	0,68	96203	2673,2
50,91	1,46	159424	2538,0
3,64	0,10	88660	1544,9
81,63	1,43	188937	2848,8
597,48	12,44	162362	7220,0
66,51	1,13	98901	1671,3
256,31	4,58	81469	4641,3
105,09	1,59	95363	3423,9
285,86	3,20	123864	4218,2
19,61	0,29	75261	1625,3
4,79	0,10	85092	1885,5
27,58	0,74	96970	2712,8
15,58	0,27	59384	1573,7
24,76	0,53	146000	2295,6
8,75	0,18	109698	1943,5
12,81	0,84	16589	2623,9
0,72	0,04	58235	2342,5
46,56	0,81	71906	2740,0
11,30	0,22	130733	2902,6
7,78	0,12	187612	3476,9
11,30	0,17	159152	2641,0
8,05	0,14	36676	1587,8
0,95	0,07	29843	2190,9

Source Data Figure 5D					
Sample ID	Sample type	months	Status	Total Area (mm²)	DAPI-Total cell #
HD1527	Donor			76,46	344774
HD1590	Donor			17,53	85207
HD1611	Donor			35,53	207752
HD1663	Donor			27,89	140435
HD1715	Donor			66,42	299502
HD2024	Donor			56,53	236475
HD2105	Donor			39,27	112556
HD2106	Donor			47,40	197753
HD2150	Donor			65,16	315733
HD3866	Adjuvant	19,68	1	82,55	121249,00
HD3868	Adjuvant	18,10	1	25,76	27043,00
HD3948	Adjuvant	77,17	1	129,44	450059,00
HD4112	Adjuvant	16,82	0	73,58	131515,00
HD4127	Adjuvant	22,47	1	54,62	255824,00
HD4132	Adjuvant	25,79	1	121,36	450043,00
HD4337	Adjuvant	13,86	1	99,89	193274,00
HD4378	Adjuvant	82,70	1	61,19	101277,00
HD4432	Adjuvant	5,32	1	44,86	183422,00
HD4585	Adjuvant	2,20	0	26,30	90886,00
HD4639	Adjuvant	20,96	1	42,98	173960,00
HD4828	Adjuvant	16,10	1	52,02	119208,00
HD4952	Adjuvant	34,43	1	59,27	176086,00
HD4954	Adjuvant	6,64	1	39,25	127141,00
HD5008	Adjuvant	84,90	0	60,72	219697,00
HD5024	Adjuvant	20,14	1	45,07	54007,00
HD5031	Adjuvant	14,75	1	55,91	141166,00
HD5153	Adjuvant	43,47	1	44,31	148750,00
HD5163	Adjuvant	50,10	1	20,60	92232,00
HD5311	Adjuvant	43,37	1	44,40	171850,00
HD5321	Adjuvant	24,77	0	61,43	132487,00
HD5353	Adjuvant	49,71	1	19,34	79703,00
HD5362	Adjuvant	18,30	1	80,49	128368,00
HD5398	Adjuvant	33,08	1	56,86	156064,00
HD5402	Adjuvant	44,85	1	51,60	203423,00
HD5457	Adjuvant	88,18	1	21,47	93954,00
HD5517	Adjuvant	9,69	0	76,30	71954,00
HD5521	Adjuvant	38,87	1	88,79	125073,00
HD5530	Adjuvant	15,18	1	105,07	393521,00
HD5534	Adjuvant	16,89	1	38,75	79208,00
HD5737	Adjuvant	12,29	1	44,51	103954,00
HD5743	Adjuvant	40,34	1	47,15	89669,00
HD5795	Adjuvant	38,64	0	39,83	179468,00
HD5919	Adjuvant	32,36	1	111,35	528387,00
HD5950	Adjuvant	15,61	1	54,64	178264,00
HD6190	Adjuvant	15,54	1	24,58	156216,00
HD6202	Adjuvant	5,13	0	34,22	72319,00
HD6221	Adjuvant	46,52	1	32,90	102775,00

HD6251	Adjuvant	11,79	1	38,87	25269,00
HD6253	Adjuvant	17,91	0	36,18	29259,00
HD6260	Adjuvant	23,80	0	35,94	65403,00
HD6268	djuvant/Blasto	19,22	1	51,92	256109,00
HD6296	Adjuvant	55,20	0	44,79	72051,00
HD6351	Adjuvant	11,53	0	50,31	169207,00
HD6359	Adjuvant	16,13	1	67,98	187153,00
HD6391	Adjuvant	53,85	0	37,29	123262,00
HD6508	Adjuvant	25,13	1	61,00	258827,00
HD6574	Adjuvant	7,69	1	34,36	72768,00
HD6726	Adjuvant	19,61	0	94,31	326981,00
HD6748	Adjuvant	11,89	0	81,75	143651,00
HD6771	Adjuvant	5,95	1	54,00	114344,00
HD6795	Adjuvant	41,86	0	81,71	166908,00
HD6821	Adjuvant	25,95	1	130,81	335498,00
HD6987	Adjuvant	17,48	1	59,67	134107,00
HD7057	Adjuvant	14,39	1	65,58	72557,00
HD7059	Adjuvant	52,24	1	56,91	95089,00
HD7155	juvant/Cholan	6,24	1	40,50	24426,00
HD7289	Adjuvant	11,53	1	71,87	201694,00
HD7372	Adjuvant	55,16	0	145,29	579167,00
HD7399	Adjuvant	55,75	0	46,43	72465,00
HD7451	Adjuvant	37,36	1	36,34	62503,00
HD7515	Adjuvant	16,62	1	58,53	143731,00
HD7559	Adjuvant	7,03	1	115,88	196372,00
HD7573	Adjuvant	40,57	0	76,65	232636,00
HD7603	Adjuvant	43,14	1	61,90	209521,00
HD7643	Adjuvant	17,61	1	136,88	583409,00
HD3530	Neoadjuvant	3,877	1	120,82	311046,00
HD3622	Neoadjuvant	0,821	0	100,62	156483,00
HD3739	Neoadjuvant	14,456	1	25,08	101526,00
HD3855	Neoadjuvant	24,115	1	92,13	84935,00
HD3907	Neoadjuvant	21,618	1	58,96	262509,00
HD4055	Neoadjuvant	2,168	1	76,10	237736,00
HD4129	Neoadjuvant	43,630	1	86,23	284194,00
HD4378	Neoadjuvant	81,478	1	61,19	101277,00
HD4593	Neoadjuvant	1,051	1	93,69	127063,00
HD4686	Neoadjuvant	20,96	1	25,19	95976,00
HD4715	Neoadjuvant	44,517	1	36,58	104119,00
HD4761	Neoadjuvant	6,702	1	47,44	135549,00
HD4880	Neoadjuvant	10,83	0	85,53	364363,00
HD4961	Neoadjuvant	10,776	1	53,98	76778,00
HD5153	Neoadjuvant	42,842	1	44,31	148750,00
HD5179	Neoadjuvant	12,747	1	53,76	207872,00
HD5276	Neoadjuvant	13,864	1	69,73	165422,50
HD5308	Neoadjuvant	36,501	1	17,12	22034,00
HD5593	Neoadjuvant	25,133	1	24,05	41944,00
HD5603	Neoadjuvant	91,466	0	12,57	28587,00
HD5648	Neoadjuvant	41,626	0	40,55	64587,00
HD5764	Neoadjuvant	20,008	1	51,46	70363,00

HD5788	Neoadjuvant	12,813	1	43,43	146832,00
HD5794	Neoadjuvant	5,257	1	33,05	41425,00
HD5811	Neoadjuvant	19,187	1	41,55	98238,00
HD5850	Neoadjuvant	7,786	1	64,72	84249,00
HD5870	Neoadjuvant	6,604	1	73,48	150982,00
HD5889	Neoadjuvant	6,242	1	52,62	283342,50
HD6035	Neoadjuvant	21,158	1	37,32	85782,00
HD6056	Neoadjuvant	28,419	1	83,93	234582,00
HD6126	Neoadjuvant	7,819	1	31,73	111804,00
HD6157	Neoadjuvant	18,267	1	30,91	129450,00
HD6196	Neoadjuvant	19,384	1	40,34	134952,00
HD6240	Neoadjuvant	21,848	0	52,81	144838,00
HD6292	Neoadjuvant	36,238	0	55,40	139776,00
HD6300	Neoadjuvant	12,945	1	47,44	108681,00
HD6321	Neoadjuvant	17,577	1	60,11	191840,00
HD6346	Neoadjuvant	11,565	1	88,43	183314,00
HD6395	Neoadjuvant	32,230	1	97,21	235344,00
HD6399	Neoadjuvant	18,004	1	35,81	18310,50
HD6436	Neoadjuvant	37,749	1	32,17	23445,00
HD6448	Neoadjuvant	5,914	1	38,81	75981,00
HD6474	Neoadjuvant	69,191	1	71,04	154229,00
HD6483	Neoadjuvant	4,797	1	66,39	210103,00
HD6520	Neoadjuvant	10,218	1	89,51	310566,00
HD6933	Neoadjuvant	65,610	0	53,82	225682,00

**cells/mm2**	**16s-positive cell #**	**16s-positive cell %**	**16s positive cell/mm2**
4509	43,00	0,01	0,562
4860	10,00	0,01	1,737
5847	320,00	0,15	9,006
5035	51,00	0,04	1,829
4509	228,00	0,07	3,432
4183	4,00	0,00	0,071
2866	113,00	0,10	2,878
4172	98,00	0,05	2,068
4846	113,00	0,04	1,734
1469	147,00	0,12	1,781
1050	214,00	0,79	8,306
3477	1735,00	0,38	13,404
1787	393,00	0,30	5,341
4684	1134,00	0,44	20,762
3708	355,00	0,08	2,925
1935	457,00	0,24	4,575
1655	1581,00	1,56	25,837
4089	18565,00	10,09	413,848
3456	122,00	0,13	4,640
4048	8606,00	4,94	200,244
2292	1810,00	1,52	34,798
2971	278,00	0,16	4,690
3240	441,00	0,35	11,237
3618	784,00	0,36	12,912
1198	55,00	0,10	1,220
2525	119,00	0,08	2,128
3357	8714,00	5,85	196,654
4476	197,00	0,21	9,561
3871	487,00	0,28	10,969
2157	586,00	0,44	9,540
4121	443,00	0,56	22,903
1595	49,00	0,04	0,609
2745	2261,00	1,45	39,763
3942	182,00	0,09	3,527
4375	635,00	0,67	29,569
943	325,00	0,45	4,259
1409	56,00	0,04	0,631
3745	3005,00	0,76	28,599
2044	64,00	0,08	1,651
2335	62,00	0,06	1,393
1902	120,00	0,13	2,545
4506	1133,00	0,63	28,448
4745	9318,00	1,76	83,682
3263	2532,00	1,42	46,339
6355	1054,00	0,67	42,879
2114	5,00	0,01	0,146
3124	38,00	0,04	1,155

650	23,00	0,09	0,592
809	10,00	0,03	0,276
1820	22,00	0,03	0,612
4933	36,00	0,01	0,693
1609	54,00	0,07	1,206
3363	466,00	0,27	9,263
2753	893,00	0,48	13,137
3306	2838,00	2,29	76,107
4243	1279,00	0,49	20,968
2118	192,00	0,26	5,588
3467	4366,00	1,32	46,292
1757	440,00	0,30	5,382
2118	241,00	0,21	4,463
2043	705,00	0,42	8,628
2565	1745,00	0,52	13,340
2247	1697,00	1,26	28,439
1106	126,00	0,17	1,921
1671	325,00	0,34	5,711
603	248,00	1,00	6,123
2806	661,00	0,32	9,197
3986	17451,00	3,01	120,109
1561	526,00	0,71	11,330
1720	395,00	0,63	10,870
2456	503,00	0,35	8,593
1695	767,00	0,39	6,6188
3035	509,00	0,22	6,640
3385	1778,00	0,85	28,723
4262	3178,00	0,54	23,217
2574	602,00	0,19	4,98
1555	165,00	0,11	1,64
4049	1441,00	1,42	57,47
922	196,00	0,23	2,13
4452	389,00	0,15	6,60
3124	366,00	0,15	4,81
3296	1569,00	0,55	18,19
1655	1581,00	1,56	25,837
1356	375,00	0,29	4,002
3810	2646,00	2,75	105,03
2846	1053,00	1,01	28,78
2857	1093,00	0,80	23,04
4260	2204,00	0,60	25,77
1422	220,00	0,29	4,08
3357	8714,00	5,85	196,654
3866	4024,00	1,93	74,85
2222	324,00	0,17	3,91
1287	130,00	0,59	7,60
1744	166,00	0,40	6,90
2274	1261,00	4,40	100,31
1593	684,00	1,06	16,87
1367	22,00	0,03	0,43

3381	717,00	0,49	16,51
1253	12,00	0,03	0,36
2365	171,50	0,17	4,14
1302	44,00	0,05	0,68
2055	157,00	0,10	2,14
6011	32099,00	10,69	454,51
2299	1405,00	1,63	37,65
2839	1425,50	0,61	16,47
3523	344,00	0,31	10,84
4188	1069,00	0,82	34,59
3346	46774,00	34,62	1159,61
2743	3275,00	2,26	62,02
2523	629,00	0,45	11,35
2291	1143,00	1,05	24,09
3191	847,00	0,44	14,09
2073	267,00	0,14	3,02
2421	458,00	0,19	4,71
1508	430,00	1,91	42,45
729	12,00	0,05	0,37
1958	20,00	0,03	0,52
2171	733,00	0,47	10,32
3164	222,00	0,11	3,34
3469	2194,00	0,71	24,51
4193	2494,00	1,10	46,34

Source Data Figure 5E				
Treatment	HD ID	SURV. Months	FISH cells/mm2 < 8.6	FISH cells/mm2 > 8.6
Adjuvant	6202	5,13	0	
Adjuvant	6253	17,91	0	
Adjuvant	6251	11,79	1	
Adjuvant	5362	18,30	1	
Adjuvant	6260	23,80	0	
Adjuvant	5521	38,87	1	
Adjuvant/Blastoma	6268	19,22	1	
Adjuvant	6221	46,52	1	
Adjuvant	6296	55,20	0	
Adjuvant	5024	20,14	1	
Adjuvant	5737	12,29	1	
Adjuvant	5534	16,89	1	
Adjuvant	3866	19,68	1	
Adjuvant	7057	14,39	1	
Adjuvant	5031	14,75	1	
Adjuvant	5743	40,34	1	
Adjuvant	4132	25,79	1	
Adjuvant	5402	44,85	1	
Adjuvant	5517	9,69	0	
Adjuvant	6771	5,95	1	
Adjuvant	4337	13,86	1	
Adjuvant	4585	2,20	0	
Adjuvant	4952	34,43	1	
Adjuvant	4112	16,82	0	
Adjuvant	6748	11,89	0	
Adjuvant	6574	7,69	1	
Adjuvant/Cholangio	7155	6,24	1	
Adjuvant	7559	7,03	1	
Adjuvant	7573	40,57	0	
Adjuvant	3868	18,10	1	
Adjuvant	7515	16,62	1	
Adjuvant	6795	41,86		0
Adjuvant	7289	11,53		1
Adjuvant	6351	11,53		0
Adjuvant	5321	24,77		0
Adjuvant	5163	50,10		1
Adjuvant	7451	37,36		1
Adjuvant	5311	43,37		1
Adjuvant	4954	6,64		1
Adjuvant	7399	55,75		0
Adjuvant	5008	84,90		0
Adjuvant	6359	16,13		1
Adjuvant	6821	25,95		1
Adjuvant	3948	77,17		1
Adjuvant	4127	22,47		1
Adjuvant	6508	25,13		1

Adjuvant	5353	49,71		1
Adjuvant	7643	17,61		1
Adjuvant	6987	17,48		1
Adjuvant	5795	38,64		0
Adjuvant	5530	15,18		1
Adjuvant	7603	43,14		1
Adjuvant	5457	88,18		1
Adjuvant	4828	16,10		1
Adjuvant	5398	33,08		1
Adjuvant	6190	15,54		1
Adjuvant	6726	19,61		0
Adjuvant	5950	15,61		1
Adjuvant	6391	53,85		0
Adjuvant	5919	32,36		1
Adjuvant	7372	55,16		0
Adjuvant	4639	20,96		1
Adjuvant	4432	5,32		1

Source Data Figure 5F				
Treatment	HD ID	SURV. Months	FISH cells/mm2 < 12.72	FISH cells/mm2 > 12.72
Neoadjuvant	5794	5,3	1	
Neoadjuvant	6436	37,7	1	
Neoadjuvant	5764	20,0	1	
Neoadjuvant	6448	5,9	1	
Neoadjuvant	5850	7,8	1	
Neoadjuvant	7809	20,3	1	
Neoadjuvant	3622	0,8	0	
Neoadjuvant	3855	24,1	1	
Neoadjuvant	5870	6,6	1	
Neoadjuvant	6346	11,6	1	
Neoadjuvant	6483	4,8	1	
Neoadjuvant	4593	1,1	1	
Neoadjuvant	4961	10,8	1	
Neoadjuvant	6395	32,2	1	
Neoadjuvant	4055	2,2	1	
Neoadjuvant	3530	3,9	1	
Neoadjuvant	5276	13,9	1	
Neoadjuvant	3907	21,6	1	
Neoadjuvant	5593	25,1	1	
Neoadjuvant	5308	36,5	1	
Neoadjuvant	5811	19,2	1	
Neoadjuvant	6474	69,2	1	
Neoadjuvant	6126	7,8	1	
Neoadjuvant	6292	36,2	0	
Neoadjuvant	6321	17,6		1
Neoadjuvant	6056	28,4		1
Neoadjuvant	5788	12,8		1
Neoadjuvant	5648	41,6		0
Neoadjuvant	4129	43,6		1
Neoadjuvant	4761	6,7		1
Neoadjuvant	5471	29,0		1
Neoadjuvant	6300	12,9		1
Neoadjuvant	6520	10,2		1
Neoadjuvant	4880	12,8		0
Neoadjuvant	4378	81,5		1
Neoadjuvant	4715	44,5		1
Neoadjuvant	6157	18,3		1
Neoadjuvant	6035	21,2		1
Neoadjuvant	6933	65,6		0
Neoadjuvant	3739	14,5		1
Neoadjuvant	6240	21,8		0
Neoadjuvant	5179	12,7		1
Neoadjuvant	6399	18,0		1
Neoadjuvant	5603	91,5		0
Neoadjuvant	4686	39,3		1
Neoadjuvant	5153	42,8		1

Neoadjuvant	5889	6,2		1
Neoadjuvant	6196	19,4		1

Source Data Figure 5G					
			FISH cells/mm2	FISH cells/mm2	
**Sample type**	**Sample ID**	**SURV. Months**	**< 9,54**	**> 9,54**	**FISH cells/mm2**
Adjuvant	HD6202	5,13	0		0,146
Adjuvant	HD6253	17,91	0		0,276
Neoadjuvant	HD5794	5,3	1		0,36
Neoadjuvant	HD6436	37,7	1		0,37
Neoadjuvant	HD5764	20,0	1		0,43
Neoadjuvant	HD6448	5,9	1		0,52
Adjuvant	HD6251	11,79	1		0,592
Adjuvant	HD5362	18,30	1		0,609
Adjuvant	HD6260	23,80	0		0,612
Adjuvant	HD5521	38,87	1		0,631
Neoadjuvant	HD5850	7,8	1		0,68
Adjuvant/Blastom	HD6268	19,22	1		0,693
Neoadjuvant	HD7809	20,3	1		0,81
Adjuvant	HD6221	46,52	1		1,155
Adjuvant	HD6296	55,20	0		1,206
Adjuvant	HD5024	20,14	1		1,220
Adjuvant	HD5737	12,29	1		1,393
Neoadjuvant	HD3622	0,8	0		1,64
Adjuvant	HD5534	16,89	1		1,651
Adjuvant	HD3866	19,68	1		1,781
Adjuvant	HD7057	14,39	1		1,921
Neoadjuvant	HD3855	24,1	1		2,13
Adjuvant	HD5031	14,75	1		2,128
Neoadjuvant	HD5870	6,6	1		2,14
Adjuvant	HD5743	40,34	1		2,545
Adjuvant	HD4132	25,79	1		2,925
Neoadjuvant	HD6346	11,6	1		3,02
Neoadjuvant	HD6483	4,8	1		3,34
Adjuvant	HD5402	44,85	1		3,527
Neoadjuvant	HD5276	13,9	1		3,91
Neoadjuvant	HD4593	1,1	1		4,002
Neoadjuvant	HD4961	10,8	1		4,08
Neoadjuvant	HD5811	19,2	1		4,14
Adjuvant	HD5517	9,69	0		4,259
Adjuvant	HD6771	5,95	1		4,463
Adjuvant	HD4337	13,86	1		4,575
Adjuvant	HD4585	2,20	0		4,640
Adjuvant	HD4952	34,43	1		4,690
Neoadjuvant	HD6395	32,2	1		4,71
Neoadjuvant	HD4055	2,2	1		4,81
Neoadjuvant	HD3530	3,9	1		4,98
Adjuvant	HD4112	16,82	0		5,341
Adjuvant	HD6748	11,89	0		5,382
Adjuvant	HD6574	7,69	1		5,588
Adjuvant/Cholangic	HD7155	6,24	1		6,123
Neoadjuvant	HD3907	21,6	1		6,60
Adjuvant	HD7559	7,03	1		6,6188

Adjuvant	HD7573	40,57	0		6,640
Neoadjuvant	HD5593	25,1	1		6,90
Neoadjuvant	HD5308	36,5	1		7,60
Adjuvant	HD3868	18,10	1		8,306
Adjuvant	HD7515	16,62	1		8,593
Adjuvant	HD6795	41,86	0		8,628
Adjuvant	HD7289	11,53	1		9,197
Adjuvant	HD6351	11,53	0		9,263
Adjuvant	HD5321	24,77	0		9,540
Adjuvant	HD5163	50,10		1	9,561
Neoadjuvant	HD6474	69,2		1	10,32
Neoadjuvant	HD6126	7,8		1	10,84
Adjuvant	HD7451	37,36		1	10,870
Adjuvant	HD5311	43,37		1	10,969
Adjuvant	HD4954	6,64		1	11,237
Adjuvant	HD7399	55,75		0	11,330
Neoadjuvant	HD6292	36,2		0	11,35
Adjuvant	HD5008	84,90		0	12,912
Adjuvant	HD6359	16,13		1	13,137
Adjuvant	HD6821	25,95		1	13,340
Adjuvant	HD3948	77,17		1	13,404
Neoadjuvant	HD6321	17,6		1	14,09
Neoadjuvant	HD6056	28,4		1	16,47
Neoadjuvant	HD5788	12,8		1	16,51
Neoadjuvant	HD5648	41,6		0	16,87
Neoadjuvant	HD4129	43,6		1	18,19
Adjuvant	HD4127	22,47		1	20,762
Adjuvant	HD6508	25,13		1	20,968
Adjuvant	HD5353	49,71		1	22,903
Neoadjuvant	HD4761	6,7		1	23,04
Adjuvant	HD7643	17,61		1	23,217
Neoadjuvant	HD5471	29,0		1	23,23
Neoadjuvant	HD6300	12,9		1	24,09
Neoadjuvant	HD6520	10,2		1	24,51
Neoadjuvant	HD4880	12,8		0	25,77
Neoadjuvant	HD4378	81,5		1	25,837
Adjuvant	HD6987	17,48		1	28,439
Adjuvant	HD5795	38,64		0	28,448
Adjuvant	HD5530	15,18		1	28,599
Adjuvant	HD7603	43,14		1	28,723
Neoadjuvant	HD4715	44,5		1	28,78
Adjuvant	HD5457	88,18		1	29,569
Neoadjuvant	HD6157	18,3		1	34,59
Adjuvant	HD4828	16,10		1	34,798
Neoadjuvant	HD6035	21,2		1	37,65
Adjuvant	HD5398	33,08		1	39,763
Neoadjuvant	HD6399	18,0		1	42,45
Adjuvant	HD6190	15,54		1	42,879
Adjuvant	HD6726	19,61		0	46,292
Adjuvant	HD5950	15,61		1	46,339

Neoadjuvant	HD6933	65,6		0	46,34
Neoadjuvant	HD3739	14,5		1	57,47
Neoadjuvant	HD6240	21,8		0	62,02
Neoadjuvant	HD5179	12,7		1	74,85
Adjuvant	HD6391	53,85		0	76,107
Adjuvant	HD5919	32,36		1	83,682
Neoadjuvant	HD5603	91,5		0	100,31
Neoadjuvant	HD4686	39,3		1	105,03
Adjuvant	HD7372	55,16		0	120,109
Neoadjuvant	HD5153	42,8		1	196,654
Adjuvant	HD4639	20,96		1	200,244
Adjuvant	HD4432	5,32		1	413,848
Neoadjuvant	HD5889	6,2		1	454,51
Neoadjuvant	HD6196	19,4		1	1159,61

Source Data Figure 6B, C, D						
**Tissue**	**HD ID**	**sample**	**months**	**stat**	**Area (mm²)**	**total cells #**
Adjuvant	HD3866	Adjuvant	19,67	1	70,92	240013
Adjuvant	HD3868	Adjuvant	18,10	1	7,53	27247
Adjuvant	HD3948	Adjuvant	77,17	1	99,08	450376
Adjuvant	HD4112	Adjuvant	16,82	0	46,98	197456
Adjuvant	HD4127	Adjuvant	22,47	1	38,66	237975
Adjuvant	HD4132	Adjuvant	25,79	1	88,12	498651
Adjuvant	HD4337	Adjuvant	13,86	1	82,76	283701
Adjuvant	HD4432	Adjuvant	5,32	1	35,52	319550
Adjuvant	HD4828	Adjuvant	16,10	1	40,48	161109
Adjuvant	HD4952	Adjuvant	34,43	1	30,13	175894
Adjuvant	HD4954	Adjuvant	6,64	1	27,91	178295
Adjuvant	HD5008	Adjuvant	84,90	0	45,38	293300
Adjuvant	HD5024	Adjuvant	20,14	1	43,28	142567
Adjuvant	HD5031	Adjuvant	14,75	1	44,67	254723
Adjuvant	HD5163	Adjuvant	50,10	1	13,83	159730
Adjuvant	HD5311	Adjuvant	43,37	1	34,47	211042
Adjuvant	HD5321	Adjuvant	24,77	0	46,65	186291
Adjuvant	HD5353	Adjuvant	49,71	1	14,54	99580
Adjuvant	HD5362	Adjuvant	18,30	1	79,13	339367
Adjuvant	HD5398	Adjuvant	33,08	1	50,92	227697
Adjuvant	HD5402	Adjuvant	44,85	1	39,67	356253
Adjuvant	HD5457	Adjuvant	88,18	1	15,12	134401
Adjuvant	HD5517	Adjuvant	9,69	0	61,94	289928
Adjuvant	HD5521	Adjuvant	38,87	1	53,83	174356
Adjuvant	HD5530	Adjuvant	15,18	1	79,05	399618
Adjuvant	HD5534	Adjuvant	16,89	1	24,36	97222
Adjuvant	HD5737	Adjuvant	12,29	1	39,03	247583
Adjuvant	HD5743	Adjuvant	40,34	1	49,02	289617
Adjuvant	HD5795	Adjuvant	38,64	0	33,35	288662
Adjuvant	HD5919	Adjuvant	32,36	1	75,71	610238
Adjuvant	HD5950	Adjuvant	15,61	1	33,09	225630
Adjuvant	HD6190	Adjuvant	15,54	1	22,92	216292
Adjuvant	HD6202	Adjuvant	5,13	0	27,16	132760
Adjuvant	HD6221	Adjuvant	46,52	1	20,09	111625
Adjuvant	HD6251	Adjuvant	11,79	1	27,64	67065
Adjuvant	HD6253	Adjuvant	17,91	0	20,93	56443
Adjuvant	HD6260	Adjuvant	23,80	0	20,53	82638
djuvant/Blasto	HD6268	Adjuvant	19,22	1	30,51	249910
Adjuvant	HD6296	Adjuvant	55,20	0	32,19	108906
Adjuvant	HD6351	Adjuvant	11,53	0	33,27	240272
Adjuvant	HD6359	Adjuvant	16,13	1	56,90	275212
Adjuvant	HD6391	Adjuvant	53,85	0	30,88	192345
Adjuvant	HD6508	Adjuvant	25,13	1	51,95	383689
Adjuvant	HD6574	Adjuvant	7,69	1	38,15	148537
Adjuvant	HD6726	Adjuvant	19,61	0	101,32	521541
Adjuvant	HD6748	Adjuvant	11,89	0	65,64	233849
Adjuvant	HD6771	Adjuvant	5,95	1	46,09	209332

Adjuvant	HD6795	Adjuvant	41,86	0	51,80	238701
Adjuvant	HD6821	Adjuvant	25,95	1	98,90	451196
Adjuvant	HD6987	Adjuvant	17,48	1	38,11	171196
Adjuvant	HD7057	Adjuvant	14,39	1	55,34	156375
Adjuvant	HD7059	Adjuvant	52,24	1	29,35	129798
uvant/Cholan	HD7155	Adjuvant	6,24	1	32,41	94652
Adjuvant	HD7289	Adjuvant	11,53	1	65,28	374288
Adjuvant	HD7372	Adjuvant	55,16	0	117,44	746077
Adjuvant	HD7399	Adjuvant	55,75	0	33,59	182919
Adjuvant	HD7451	Adjuvant	37,36	1	33,69	134847
Adjuvant	HD7515	Adjuvant	16,62	1	50,64	208721
Adjuvant	HD7559	Adjuvant	7,03	1	85,57	307099
Adjuvant	HD7573	Adjuvant	40,57	0	55,14	314221
Adjuvant	HD7603	Adjuvant	43,14	1	54,07	269585
Adjuvant	HD7643	Adjuvant	17,61	1	109,91	736059
Neoadjuvant	HD3530	Neoadjuvant	3,88	1	82,94	346170
Neoadjuvant	HD3622	Neoadjuvant	0,82	0	43,95	112481
Neoadjuvant	HD3739	Neoadjuvant	14,46	1	25,19	205609
Neoadjuvant	HD3855	Neoadjuvant	24,11	1	58,18	149940
Neoadjuvant	HD3907	Neoadjuvant	21,62	1	29,52	191066
Neoadjuvant	HD4055	Neoadjuvant	2,17	1	56,64	304873
Neoadjuvant	HD4129	Neoadjuvant	43,63	1	53,83	322162
Neoadjuvant	HD4378	Neoadjuvant	81,48	1	48,47	130240
Neoadjuvant	HD4593	Neoadjuvant	1,05	1	66,94	190640
Neoadjuvant	HD4639	Neoadjuvant	38,77	1	25,52	134404
Neoadjuvant	HD4686	Neoadjuvant	20,96	1	17,30	110613
Neoadjuvant	HD4715	Neoadjuvant	44,52	1	28,17	134272
Neoadjuvant	HD4761	Neoadjuvant	6,70	1	30,64	133229
Neoadjuvant	HD4880	Neoadjuvant	10,83	0	50,36	300696
Neoadjuvant	HD4961	Neoadjuvant	10,78	1	17,75	61190
Neoadjuvant	HD5153	Neoadjuvant	42,84	1	39,42	199528
Neoadjuvant	HD5179	Neoadjuvant	12,75	1	40,62	234901
Neoadjuvant	HD5276	Neoadjuvant	13,86	1	58,83	253588
Neoadjuvant	HD5308	Neoadjuvant	36,50	1	8,62	27927
Neoadjuvant	HD5471	Neoadjuvant	28,98	1	33,29	173525
Neoadjuvant	HD5593	Neoadjuvant	25,13	1	22,78	116354
Neoadjuvant	HD5603	Neoadjuvant	91,47	0	8,99	35101
Neoadjuvant	HD5648	Neoadjuvant	41,63	0	25,34	81180
Neoadjuvant	HD5764	Neoadjuvant	20,01	1	41,25	157076
Neoadjuvant	HD5788	Neoadjuvant	12,81	1	28,83	153077
Neoadjuvant	HD5794	Neoadjuvant	5,26	1	29,70	98204
Neoadjuvant	HD5811	Neoadjuvant	19,19	1	35,99	142752
Neoadjuvant	HD5850	Neoadjuvant	7,79	1	62,81	219191
Neoadjuvant	HD5870	Neoadjuvant	6,60	1	57,39	211630
Neoadjuvant	HD5889	Neoadjuvant	6,24	1	66,32	378708
Neoadjuvant	HD6035	Neoadjuvant	21,16	1	22,49	108041
Neoadjuvant	HD6056	Neoadjuvant	28,42	1	59,17	349015
Neoadjuvant	HD6126	Neoadjuvant	7,82	1	17,55	98259
Neoadjuvant	HD6157	Neoadjuvant	18,27	1	27,85	184658
Neoadjuvant	HD6196	Neoadjuvant	19,38	1	29,36	262483

Neoadjuvant	HD6240	Neoadjuvant	21,85	0	46,31	311871
Neoadjuvant	HD6292	Neoadjuvant	36,24	0	45,13	213259
Neoadjuvant	HD6300	Neoadjuvant	12,94	1	35,74	133323
Neoadjuvant	HD6321	Neoadjuvant	17,58	1	37,74	214550
Neoadjuvant	HD6346	Neoadjuvant	11,56	1	63,60	298301
Neoadjuvant	HD6395	Neoadjuvant	32,23	1	56,44	281773
Neoadjuvant	HD6399	Neoadjuvant	18,00	1	6,32	9619
Neoadjuvant	HD6436	Neoadjuvant	37,75	1	24,86	49827
Neoadjuvant	HD6448	Neoadjuvant	5,91	1	26,24	151097
Neoadjuvant	HD6474	Neoadjuvant	69,19	1	45,04	229951
Neoadjuvant	HD6483	Neoadjuvant	4,80	1	53,96	364007
Neoadjuvant	HD6520	Neoadjuvant	10,22	1	60,26	403412
Neoadjuvant	HD6933	Neoadjuvant	65,61	0	23,10	135416
Neoadjuvant	HD7809	Neoadjuvant	20,27	1	13,62	19222

MP2 cells cou	C3B Cells coun	MP2 cells/mr	C3B cells/mm	2_LC3B cells/	LAMP2 %	LC3B %
31756	35032	447,74	493,93	423,82	13,23	14,60
7794	8144	1035,44	1081,94	954,40	28,60	29,89
48919	46255	493,75	466,86	394,65	10,86	10,27
45563	47029	969,74	1000,94	902,23	23,08	23,82
65308	65693	1689,47	1699,43	1479,62	27,44	27,61
14238	14711	161,58	166,94	110,88	2,86	2,95
34735	17103	419,70	206,66	197,69	12,24	6,03
284718	239277	8016,72	6737,26	6735,82	89,10	74,88
73102	65467	1805,88	1617,27	1594,32	45,37	40,64
103586	70037	3437,88	2324,44	2308,37	58,89	39,82
13987	5681	501,20	203,57	200,88	7,84	3,19
21550	4020	474,92	88,59	88,00	7,35	1,37
18807	8787	434,58	203,05	195,37	13,19	6,16
8061	3659	180,47	81,92	79,10	3,16	1,44
105992	80834	7665,84	5846,30	5837,98	66,36	50,61
55948	34914	1623,09	1012,88	1002,70	26,51	16,54
26461	14896	567,28	319,35	314,76	14,20	8,00
66433	9643	4569,01	663,21	662,93	66,71	9,68
14486	6736	183,07	85,13	81,44	4,27	1,98
3049	1526	59,88	29,97	25,67	1,34	0,67
31393	20514	791,30	517,08	497,09	8,81	5,76
18968	16426	1254,37	1086,27	884,17	14,11	12,22
15764	9255	254,52	149,43	136,61	5,44	3,19
14371	7997	266,96	148,56	148,20	8,24	4,59
38524	24020	487,36	303,88	293,93	9,64	6,01
3939	2895	161,69	118,84	106,56	4,05	2,98
14339	11038	367,37	282,80	252,24	5,79	4,46
16517	8197	336,91	167,20	160,31	5,70	2,83
24037	5129	720,80	153,80	148,92	8,33	1,78
7800	4585	103,03	60,56	55,87	1,28	0,75
133719	93382	4040,59	2821,73	2818,83	59,26	41,39
65226	42223	2846,06	1842,35	1834,32	30,16	19,52
18058	19933	664,85	733,88	597,36	13,60	15,01
19960	19429	993,55	967,11	870,05	17,88	17,41
7497	7403	271,28	267,88	226,66	11,18	11,04
2316	2244	110,64	107,20	76,96	4,10	3,98
9119	9150	444,13	445,64	387,73	11,03	11,07
44741	40801	1466,57	1337,42	1140,87	17,90	16,33
22158	24805	688,27	770,49	645,40	20,35	22,78
18286	16471	549,56	495,01	423,03	7,61	6,86
54029	48605	949,48	854,16	824,75	19,63	17,66
33855	33581	1096,43	1087,55	961,44	17,60	17,46
142592	121350	2744,93	2336,02	2282,62	37,16	31,63
29003	27701	760,17	726,04	670,90	19,53	18,65
958	2653	9,46	26,19	8,45	0,18	0,51
6468	11669	98,54	177,78	94,98	2,77	4,99
16914	25490	366,97	553,03	354,88	8,08	12,18

37929	44361	732,20	856,37	691,25	15,89	18,58
16963	32004	171,52	323,60	159,42	3,76	7,09
6947	11328	182,28	297,23	175,59	4,06	6,62
5436	10168	98,23	183,73	94,97	3,48	6,50
13060	21168	445,04	721,34	441,13	10,06	16,31
4391	7636	135,47	235,59	127,30	4,64	8,07
1342	1822	20,56	27,91	14,51	0,36	0,49
7868	5190	67,00	44,19	25,78	1,05	0,70
47285	45348	1407,72	1350,05	1217,28	25,85	24,79
31249	27811	927,58	825,53	753,49	23,17	20,62
9431	13581	186,23	268,18	176,57	4,52	6,51
649	1401	7,58	16,37	7,15	0,21	0,46
45783	57364	830,37	1040,42	789,42	14,57	18,26
22209	27340	410,74	505,64	366,41	8,24	10,14
17155	21273	156,08	193,54	119,26	2,33	2,89
99581	75461	1200,71	909,88	907,84	28,77	21,80
28092	12954	639,18	294,74	291,17	24,97	11,52
116246	28415	4614,01	1127,84	1127,84	56,54	13,82
47712	25330	820,13	435,40	433,58	31,82	16,89
139913	106660	4740,05	3613,49	3613,35	73,23	55,82
87614	23215	1546,81	409,86	406,68	28,74	7,61
119176	80188	2213,98	1489,69	1460,13	36,99	24,89
29244	24799	603,36	511,65	497,52	22,45	19,04
3950	3055	59,00	45,64	34,31	2,07	1,60
58942	55468	2309,95	2173,80	2061,68	43,85	41,27
54601	17746	3156,76	1025,99	1025,75	49,36	16,04
86749	53201	3079,17	1888,38	1887,64	64,61	39,62
36198	22674	1181,58	740,13	740,07	27,17	17,02
58419	40920	1160,03	812,55	777,82	19,43	13,61
10335	4693	582,34	264,43	264,10	16,89	7,67
59422	44263	1507,34	1122,81	1083,03	29,78	22,18
98769	52149	2431,30	1283,70	1281,58	42,05	22,20
38052	23921	646,77	406,58	376,91	15,01	9,43
6156	0	714,07	0,00	0,00	22,04	0,00
64153	38629	1926,85	1160,23	1159,57	36,97	22,26
65489	38851	2875,21	1705,70	1703,29	56,28	33,39
13246	7177	1473,45	798,35	798,35	37,74	20,45
20480	13501	808,30	532,86	521,88	25,23	16,63
36122	28447	875,78	689,70	676,63	23,00	18,11
49924	14417	1731,73	500,09	496,69	32,61	9,42
15938	8499	536,56	286,13	282,86	16,23	8,65
64493	36918	1792,06	1025,83	1025,78	45,18	25,86
57291	15440	912,06	245,80	245,80	26,14	7,04
19508	9937	339,93	173,15	168,26	9,22	4,70
49015	12241	739,05	184,57	184,56	12,94	3,23
30121	10152	1339,44	451,45	450,60	27,88	9,40
45073	16229	761,69	274,26	273,56	12,91	4,65
65468	26105	3729,73	1487,21	1487,21	66,63	26,57
143758	60363	5161,50	2167,28	2167,28	77,85	32,69
106679	39918	3632,99	1359,42	1359,08	40,64	15,21

61500	33122	1328,10	715,27	710,78	19,72	10,62
23265	9716	515,52	215,29	214,98	10,91	4,56
40823	24827	1142,07	694,56	694,09	30,62	18,62
31039	12978	822,52	343,91	327,83	14,47	6,05
38796	20950	609,99	329,40	323,25	13,01	7,02
78901	46398	1397,85	822,01	818,46	28,00	16,47
3252	1687	514,37	266,84	266,84	33,81	17,54
4900	4343	197,10	174,70	159,37	9,83	8,72
38349	24620	1461,30	938,15	934,65	25,38	16,29
24734	13973	549,15	310,23	303,13	10,76	6,08
31829	15562	589,87	288,40	282,70	8,74	4,28
28975	13601	480,82	225,70	202,77	7,18	3,37
98363	77987	4258,46	3376,31	3370,81	72,64	57,59
4973	3947	365,08	289,76	284,55	25,87	20,53

Lamp2_LC3B %	AMP2_LC3B ce	LAMP2 dots	LC3B dots	LPS_LC3B dots	AMP2_LC3B do	P2_LC3B_LPS
12,52	30059	722890	750507	14731	639097	13317
26,37	7184	169338	154717	3576	149267	3449
8,68	39101	593503	465923	16399	413140	15769
21,47	42391	681144	687524	7205	617316	6619
24,03	57196	584397	549717	8577	512544	8054
1,96	9771	572459	435225	7648	390096	6722
5,77	16361	660113	488223	13831	453894	13405
74,86	239226	1868815	1585696	37817	1584961	37804
40,06	64538	1560482	1268591	24240	1257556	24047
39,54	69553	1080094	764898	30968	763779	30938
3,14	5606	562314	273537	1347	270411	1335
1,36	3993	560081	204326	1064	202872	1063
5,93	8455	430907	332362	26119	325957	26082
1,39	3533	415758	240445	2609	233318	2569
50,53	80719	493351	415452	104041	414553	104015
16,38	34563	835006	637533	8835	626270	8789
7,88	14682	658623	456194	6714	447757	6650
9,68	9639	790795	253698	6707	253503	6703
1,90	6444	313179	206012	18524	193270	18385
0,57	1307	169243	108360	4046	98964	3951
5,54	19721	801491	558844	4056	543344	3956
9,95	13370	333435	112434	688	110386	681
2,92	8461	369998	291891	3871	267566	3744
4,58	7978	375604	231028	2956	226925	2902
5,81	23234	2200625	1288689	12353	1259784	11923
2,67	2596	284990	232120	1891	216101	1815
3,98	9845	506140	397159	5803	368595	5603
2,71	7859	518826	347884	31479	326507	31328
1,72	4966	808831	331456	4194	325698	4158
0,69	4230	641665	334694	2686	309918	2632
41,34	93286	1484843	1203930	207697	1202154	207666
19,44	42039	1087156	847414	8878	841433	8830
12,22	16225	915294	822119	19050	785964	18085
15,66	17479	583957	478018	8509	456184	7975
9,34	6264	399779	358778	11323	327940	10920
2,85	1611	211547	164385	5437	143874	5075
9,63	7961	199917	174092	5840	163834	5442
13,93	34805	790353	677159	20709	631569	20071
19,08	20778	581412	515058	10172	468926	8994
5,86	14076	679927	484592	5613	458234	5116
17,05	46931	1431339	1082383	31053	1041752	30190
15,43	29687	689634	587173	15169	560150	14429
30,90	118576	2936242	2425438	87204	2390834	85863
17,23	25597	477168	396186	11928	383517	11632
0,16	856	103108	102998	564	61970	452
2,67	6234	581372	757447	12105	530688	9485
7,81	16357	894912	933294	16853	799205	14290

15,00	35808	1506601	1537772	58859	1419798	53918
3,49	15766	597322	772087	21702	557177	18149
3,91	6692	501897	633625	16811	473563	13907
3,36	5256	317264	435541	15495	297660	11501
9,97	12945	408265	493954	13978	389010	11852
4,36	4126	237897	289620	19225	225865	15887
0,25	947	327746	281609	2297	212711	1974
0,41	3028	597956	482857	4315	378427	3675
22,35	40888	777044	702762	80224	663796	78099
18,82	25384	851282	758953	28123	721933	27060
4,28	8942	515085	599530	11950	465729	9007
0,20	612	144819	155249	1428	105339	1132
13,85	43525	850866	934474	26573	781491	21993
7,35	19812	622805	595442	11864	520658	9981
1,78	13108	809051	767659	13048	645355	11878
21,75	75292	2125530	1395575	24994	1391275	24953
11,38	12797	489219	202036	7365	201424	7362
13,82	28415	1424357	578823	23711	578737	23710
16,82	25224	1556278	953270	19318	947049	19260
55,82	106656	1505963	1182815	30038	1181930	30015
7,56	23035	1959723	1048309	36512	1045798	36489
24,40	78597	1560871	1135125	69869	1126428	69745
18,52	24114	387978	349864	4699	313845	4423
1,20	2297	405932	303178	3026	261040	2831
39,14	52607	629659	555128	7157	550148	6980
16,04	17742	901147	487181	8728	486808	8723
39,61	53180	1389358	1013523	23237	1012603	23210
17,02	22672	901552	553560	16055	553205	16044
13,03	39171	968408	428114	2445	422205	2423
7,66	4687	286382	195977	16119	194098	16054
21,40	42695	1528108	1025479	17073	1009216	16697
22,16	52063	1863076	1071971	16922	1068440	16901
8,74	22175	1287112	827551	17608	800291	17133
0,00	0	388793	0	0	0	0
22,25	38607	1292500	851412	33783	848699	33732
33,34	38796	1130799	633119	21853	632600	21811
20,45	7177	304806	181494	6909	181319	6908
16,29	13223	711726	498384	11298	495595	11235
17,77	27908	1204709	882053	24398	869674	24019
9,35	14319	976832	402216	5641	401392	5633
8,56	8402	458090	304842	16039	301163	15896
25,86	36916	1486755	967036	49442	966941	49431
7,04	15440	2127362	1022419	82887	1021247	82863
4,56	9656	1002265	603043	14108	575331	13789
3,23	12240	990294	427012	29503	426824	29490
9,38	10133	862244	435575	71755	435009	71743
4,64	16188	502666	272444	24944	271699	24928
26,57	26105	598622	387305	56176	387279	56176
32,69	60363	1926643	1073914	43622	1073909	43622
15,20	39908	785839	416906	64442	416692	64439

10,55	32914	936419	535477	23365	533319	23334
4,55	9702	917653	495124	14896	494177	14866
18,61	24810	979246	726582	56619	725879	56594
5,77	12371	739912	396711	8895	391802	8846
6,89	20559	890938	544819	21540	530665	21288
16,40	46198	1646347	881138	22752	875978	22547
17,54	1687	112736	73972	6281	73917	6275
7,95	3962	273730	246581	10943	239008	10678
16,23	24528	607659	403924	21754	402375	21703
5,94	13653	808586	602816	21752	597530	21559
4,19	15254	521856	321681	17556	316112	17331
3,03	12219	938409	540320	20809	521272	20242
57,50	77860	967505	833249	21732	832906	21700
20,16	3876	223003	179802	5560	177150	5456

**_LC3B_LPS do**	**P2_LC3B dots/mm2**
187,76	9010,9
458,20	19830,3
159,16	4169,9
140,88	13138,6
208,35	13259,1
76,28	4426,9
161,97	5484,4
1064,44	44627,3
594,05	31066,2
1026,79	25348,8
47,84	9689,6
23,43	4470,9
602,69	7532,1
57,51	5223,5
7522,85	29982,4
254,97	18168,5
142,57	9599,2
461,01	17435,0
232,34	2442,4
77,59	1943,5
99,72	13695,6
45,04	7299,9
60,45	4320,0
53,91	4215,5
150,84	15937,4
74,50	8870,8
143,55	9443,6
639,03	6660,1
124,69	9766,7
34,77	4093,7
6275,05	36325,5
385,29	36714,9
665,84	28937,1
396,97	22707,4
395,14	11866,4
242,43	6872,9
265,05	7979,3
657,91	20702,2
279,37	14565,7
153,75	13771,5
530,55	18307,3
467,30	18141,0
1652,88	46024,2
304,87	10052,0
4,46	611,6
144,51	8085,4
310,04	17339,6

1040,86	27408,5
183,51	5633,8
364,89	12425,5
207,82	5378,6
403,88	13256,3
490,15	6968,5
30,24	3258,3
31,29	3222,3
2325,08	19761,9
803,24	21429,6
177,86	9196,5
13,23	1231,0
398,89	14174,0
184,59	9629,3
108,07	5871,4
300,87	16775,5
167,51	4583,0
941,09	22971,1
331,06	16278,9
1016,87	40042,1
644,21	18463,4
1295,68	20926,1
91,26	6475,2
42,29	3899,4
273,55	21560,4
504,32	28144,8
823,84	35942,6
523,71	18057,9
48,11	8383,8
904,59	10936,8
423,55	25600,5
416,04	26300,7
291,21	13602,5
0,00	0,0
1013,15	25490,8
957,58	27773,5
768,43	20169,4
443,42	19560,1
582,34	21085,3
195,39	13923,2
535,15	10138,9
1373,53	26868,2
1319,16	16258,1
240,27	10025,1
444,65	6435,7
3190,31	19344,2
421,26	4591,5
3200,36	22063,4
1566,21	38557,7
2194,49	14190,6

503,90	11517,1
329,41	10950,3
1583,28	20307,3
234,42	10382,6
334,71	8343,6
399,45	15519,2
992,53	11691,6
429,52	9614,1
827,00	15332,6
478,66	13266,6
321,19	5858,4
335,90	8650,2
939,46	36059,2
400,54	13005,2

Source Data Figure 6E

Treatment	HD ID	SURV. Months	LAMP2_LC3B cells/mm2 < 360	LAMP2_LC3B cells/mm2 > 360
Adjuvant	7559	7,03	1	
Adjuvant	6726	19,61	0	
Adjuvant	7289	11,53	1	
Adjuvant	5398	33,08	1	
Adjuvant	7372	55,16	0	
Adjuvant	5919	32,36	1	
Adjuvant	6253	17,91	0	
Adjuvant	5031	14,75	1	
Adjuvant	5362	18,3	1	
Adjuvant	5008	84,9	0	
Adjuvant	7057	14,39	1	
Adjuvant	6748	11,89	0	
Adjuvant	5534	16,89	1	
Adjuvant	4132	25,79	1	
Adjuvant	7643	17,61	1	
Adjuvant	7155	6,24	1	
Adjuvant	5517	9,69	0	
Adjuvant	5521	38,87	1	
Adjuvant	5795	38,64	0	
Adjuvant	6821	25,95	1	
Adjuvant	5743	40,34	1	
Adjuvant	6987	17,48	1	
Adjuvant	7515	16,62	1	
Adjuvant	5024	20,14	1	
Adjuvant	4337	13,86	1	
Adjuvant	4954	6,64	1	
Adjuvant	6251	11,79	1	
Adjuvant	5737	12,29	1	
Adjuvant	5530	15,18	1	
Adjuvant	5321	24,77	0	
Adjuvant	6771	5,95	1	
Adjuvant	7603	43,14		1
Adjuvant	6260	23,8		0
Adjuvant	3948	77,17		1
Adjuvant	6351	11,53		0
Adjuvant	3866	19,67		1
Adjuvant	7059	52,24		1
Adjuvant	5402	44,85		1
Adjuvant	6202	5,13		0
Adjuvant	6296	55,2		0
Adjuvant	5353	49,71		1
Adjuvant	6574	7,69		1
Adjuvant	6795	41,86		0
Adjuvant	7451	37,36		1
Adjuvant	7573	40,57		0

Adjuvant	6359	16,13		1
Adjuvant	6221	46,52		1
Adjuvant	5457	88,18		1
Adjuvant	4112	16,82		0
Adjuvant	3868	18,1		1
Adjuvant	6391	53,85		0
Adjuvant	5311	43,37		1
Adjuvant	6268	19,22		1
Adjuvant	7399	55,75		0
Adjuvant	4127	22,47		1
Adjuvant	4828	16,1		1
Adjuvant	6190	15,54		1
Adjuvant	6508	25,13		1
Adjuvant	4952	34,43		1
Adjuvant	5950	15,61		1
Adjuvant	5163	50,1		1
Adjuvant	4432	5,32		1

Source Data Figure 6F

Treatment	HD ID	SURV. Months	LAMP2_LC3B cells/mm2 < 490	LAMP2_LC3B cells/mm2 > 490
Neoadjuvant	HD4593	1,05	1	
Neoadjuvant	HD6436	37,75	1	
Neoadjuvant	HD5870	6,6	1	
Neoadjuvant	HD5889	6,24	1	
Neoadjuvant	HD6520	10,22	1	
Neoadjuvant	HD6292	36,24	0	
Neoadjuvant	HD5850	7,79	1	
Neoadjuvant	HD4961	10,78	1	
Neoadjuvant	HD6399	18	1	
Neoadjuvant	HD6056	28,42	1	
Neoadjuvant	HD6483	4,8	1	
Neoadjuvant	HD5794	5,26	1	
Neoadjuvant	HD7809	20,27	1	
Neoadjuvant	HD3622	0,82	0	
Neoadjuvant	HD6474	69,19	1	
Neoadjuvant	HD6346	11,56	1	
Neoadjuvant	HD6321	17,58	1	
Neoadjuvant	HD5276	13,86	1	
Neoadjuvant	HD4055	2,17	1	
Neoadjuvant	HD3855	24,11	1	
Neoadjuvant	HD6035	21,16	1	
Neoadjuvant	HD5788	12,81	1	
Neoadjuvant	HD4378	81,48		1
Neoadjuvant	HD5648	41,63		0
Neoadjuvant	HD5764	20,01		1
Neoadjuvant	HD6300	12,94		1
Neoadjuvant	HD6240	21,85		0
Neoadjuvant	HD4761	6,7		1
Neoadjuvant	HD4880	10,83		0
Neoadjuvant	HD5603	91,47		0
Neoadjuvant	HD6395	32,23		1
Neoadjuvant	HD3530	3,88		1
Neoadjuvant	HD6448	5,91		1
Neoadjuvant	HD4686	20,96		1
Neoadjuvant	HD5811	19,19		1
Neoadjuvant	HD5153	42,84		1
Neoadjuvant	HD3739	14,46		1
Neoadjuvant	HD5471	28,98		1
Neoadjuvant	HD5179	12,75		1
Neoadjuvant	HD6196	19,38		1
Neoadjuvant	HD4129	43,63		1
Neoadjuvant	HD6126	7,82		1
Neoadjuvant	HD5593	25,13		1
Neoadjuvant	HD4715	44,52		1
Neoadjuvant	HD4639	38,77		1

Neoadjuvant	HD6157	18,27		1

Source Data Figure 6G					
			LAMP2_LC3B	LAMP2_LC3B	
sample	ID	months	cells/mm2 < 497,6	cells/mm2 > 497,6	cells/mm2
Adjuvant	HD7559	7,03	1		16,37
Adjuvant	HD6726	19,61	0		26,19
Adjuvant	HD7289	11,53	1		27,91
Adjuvant	HD5398	33,08	1		29,97
Adjuvant	HD7372	55,16	0		44,19
Neoadjuvant	HD4593	1,05	1		45,64
Adjuvant	HD5919	32,36	1		60,56
Adjuvant	HD5031	14,75	1		81,92
Adjuvant	HD5362	18,30	1		85,13
Adjuvant	HD5008	84,90	0		88,59
Adjuvant	HD6253	17,91	0		107,20
Adjuvant	HD5534	16,89	1		118,84
Adjuvant	HD5521	38,87	1		148,56
Adjuvant	HD5517	9,69	0		149,43
Adjuvant	HD5795	38,64	0		153,80
Adjuvant	HD4132	25,79	1		166,94
Adjuvant	HD5743	40,34	1		167,20
Neoadjuvant	HD5870	6,60	1		173,15
Neoadjuvant	HD6436	37,75	1		174,70
Adjuvant	HD6748	11,89	0		177,78
Adjuvant	HD7057	14,39	1		183,73
Neoadjuvant	HD5889	6,24	1		184,57
Adjuvant	HD7643	17,61	1		193,54
Adjuvant	HD5024	20,14	1		203,05
Adjuvant	HD4954	6,64	1		203,57
Adjuvant	HD4337	13,86	1		206,66
Neoadjuvant	HD6292	36,24	0		215,29
Neoadjuvant	HD6520	10,22	1		225,70
Adjuvant	HD7155	6,24	1		235,59
Neoadjuvant	HD5850	7,79	1		245,80
Neoadjuvant	HD4961	10,78	1		264,43
Neoadjuvant	HD6399	18,00	1		266,84
Adjuvant	HD6251	11,79	1		267,88
Adjuvant	HD7515	16,62	1		268,18
Neoadjuvant	HD6056	28,42	1		274,26
Adjuvant	HD5737	12,29	1		282,80
Neoadjuvant	HD5794	5,26	1		286,13
Neoadjuvant	HD6483	4,80	1		288,40
Neoadjuvant	HD7809	20,27	1		289,76
Neoadjuvant	HD3622	0,82	0		294,74
Adjuvant	HD6987	17,48	1		297,23
Adjuvant	HD5530	15,18	1		303,88
Neoadjuvant	HD6474	69,19	1		310,23
Adjuvant	HD5321	24,77	0		319,35
Adjuvant	HD6821	25,95	1		323,60
Neoadjuvant	HD6346	11,56	1		329,40

Neoadjuvant	HD6321	17,58	1		343,91
Neoadjuvant	HD5276	13,86	1		406,58
Neoadjuvant	HD4055	2,17	1		409,86
Neoadjuvant	HD3855	24,11	1		435,40
Adjuvant	HD6260	23,80	0		445,64
Neoadjuvant	HD6035	21,16	1		451,45
Adjuvant	HD3948	77,17	1		466,86
Adjuvant	HD3866	19,67	1		493,93
Adjuvant	HD6351	11,53	0		495,01
Neoadjuvant	HD5788	12,81		1	500,09
Adjuvant	HD7603	43,14		1	505,64
Neoadjuvant	HD4378	81,48		1	511,65
Adjuvant	HD5402	44,85		1	517,08
Neoadjuvant	HD5648	41,63		0	532,86
Adjuvant	HD6771	5,95		1	553,03
Adjuvant	HD5353	49,71		1	663,21
Neoadjuvant	HD5764	20,01		1	689,70
Neoadjuvant	HD6300	12,94		1	694,56
Neoadjuvant	HD6240	21,85		0	715,27
Adjuvant	HD7059	52,24		1	721,34
Adjuvant	HD6574	7,69		1	726,04
Adjuvant	HD6202	5,13		0	733,88
Neoadjuvant	HD4761	6,70		1	740,13
Adjuvant	HD6296	55,20		0	770,49
Neoadjuvant	HD5603	91,47		0	798,35
Neoadjuvant	HD4880	10,83		0	812,55
Neoadjuvant	HD6395	32,23		1	822,01
Adjuvant	HD7451	37,36		1	825,53
Adjuvant	HD6359	16,13		1	854,16
Adjuvant	HD6795	41,86		0	856,37
Neoadjuvant	HD3530	3,88		1	909,88
Neoadjuvant	HD6448	5,91		1	938,15
Adjuvant	HD6221	46,52		1	967,11
Adjuvant	HD4112	16,82		0	1000,94
Adjuvant	HD5311	43,37		1	1012,88
Neoadjuvant	HD5811	19,19		1	1025,83
Neoadjuvant	HD4686	20,96		1	1025,99
Adjuvant	HD7573	40,57		0	1040,42
Adjuvant	HD3868	18,10		1	1081,94
Adjuvant	HD5457	88,18		1	1086,27
Adjuvant	HD6391	53,85		0	1087,55
Neoadjuvant	HD5153	42,84		1	1122,81
Neoadjuvant	HD3739	14,46		1	1127,84
Neoadjuvant	HD5471	28,98		1	1160,23
Neoadjuvant	HD5179	12,75		1	1283,70
Adjuvant	HD6268	19,22		1	1337,42
Adjuvant	HD7399	55,75		0	1350,05
Neoadjuvant	HD6196	19,38		1	1359,42
Neoadjuvant	HD6126	7,82		1	1487,21
Neoadjuvant	HD4129	43,63		1	1489,69

Adjuvant	HD4828	16,10		1	1617,27
Adjuvant	HD4127	22,47		1	1699,43
Neoadjuvant	HD5593	25,13		1	1705,70
Adjuvant	HD6190	15,54		1	1842,35
Neoadjuvant	HD4715	44,52		1	1888,38
Neoadjuvant	HD6157	18,27		1	2167,28
Neoadjuvant	HD4639	38,77		1	2173,80
Adjuvant	HD4952	34,43		1	2324,44
Adjuvant	HD6508	25,13		1	2336,02
Adjuvant	HD5950	15,61		1	2821,73
Neoadjuvant	HD6933	65,61		0	3376,31
Neoadjuvant	HD3907	21,62		1	3613,49
Adjuvant	HD5163	50,10		1	5846,30
Adjuvant	HD4432	5,32		1	6737,26

Source Data Figure 6H

LC3B mRNA

sample_id	month	LC3B mRNA_low	LC3B mRNA_high
GSM1844112	7	1	
GSM1844113	11	1	
GSM1844114	47	0	
GSM1844115	10	1	
GSM1844116	1	1	
GSM1844117	6	1	
GSM1844118	2	1	
GSM1844119	2	0	
GSM1844120	17	0	
GSM1844125	19	1	
GSM1844128	18	1	
GSM1844130	43	0	
GSM1844133	20	1	
GSM1844136	21	1	
GSM1844137	30	0	
GSM1844139	31	1	
GSM1844144	4	1	
GSM1844145	22	0	
GSM1844146	6	1	
GSM1844147	10	1	
GSM1844152	21	0	
GSM1844154	15	1	
GSM1844158	1	1	
GSM1844159	10	1	
GSM1844162	41	1	
GSM1844163	23	1	
GSM1844164	7	1	
GSM1844165	1	0	
GSM1844166	18	0	
GSM1844168	8	1	
GSM1844171	1	1	
GSM1844172	4	1	
GSM1844174	33	1	
GSM1844175	7	1	
GSM1844177	52	0	
GSM1844179	16	1	
GSM1844180	18	1	
GSM1844181	9	1	
GSM1844182	12	1	
GSM1844185	32	1	
GSM1844190	3	1	
GSM1844193	13	1	
GSM1844196	4	1	
GSM1844199	13	0	
GSM1844205	8	0	

GSM1844206	8	0	
GSM1844208	2	1	
GSM1844209	14	1	
GSM1844218	7	1	
GSM1844219	13	1	
GSM1844223	7	1	
GSM1844227	25	1	
GSM1844228	30	0	
GSM1844229	22	1	
GSM1844230	6	1	
GSM1844231	14	1	
GSM1844232	1	0	
GSM1844233	6	0	
GSM1844236	3	1	
GSM1844237	19	1	
GSM1844238	17	1	
GSM1844241	2	1	
GSM1844121	49		1
GSM1844122	52		0
GSM1844123	55		0
GSM1844124	8		1
GSM1844126	52		0
GSM1844129	29		1
GSM1844131	6		1
GSM1844132	42		0
GSM1844134	35		1
GSM1844135	13		1
GSM1844138	33		0
GSM1844140	35		0
GSM1844141	13		1
GSM1844142	8		1
GSM1844143	18		0
GSM1844148	25		0
GSM1844149	18		1
GSM1844150	13		1
GSM1844151	19		0
GSM1844153	23		0
GSM1844155	11		1
GSM1844156	4		1
GSM1844157	13		0
GSM1844160	59		0
GSM1844161	21		1
GSM1844167	15		1
GSM1844169	28		1
GSM1844170	1		0
GSM1844173	10		1
GSM1844178	45		1
GSM1844186	0		1
GSM1844187	0		0
GSM1844188	7		1

GSM1844189	3	1
GSM1844191	15	1
GSM1844192	26	1
GSM1844194	33	0
GSM1844198	15	0
GSM1844200	4	0
GSM1844201	10	0
GSM1844202	2	0
GSM1844203	8	0
GSM1844204	7	0
GSM1844207	17	1
GSM1844210	4	1
GSM1844211	1	0
GSM1844212	29	0
GSM1844213	1	0
GSM1844214	8	1
GSM1844215	54	1
GSM1844216	6	1
GSM1844217	7	1
GSM1844220	16	1
GSM1844221	15	1
GSM1844222	6	1
GSM1844224	18	1
GSM1844225	7	1
GSM1844226	22	1
GSM1844234	7	1
GSM1844235	19	1
GSM1844239	1	0
GSM1844240	20	1
GSM1844249	24	1
