# Supplementary material for: Autophagy and intratumoral bacteria abundance influence the prognosis of patients with pancreatic carcinoma
Source: Cell Death Dis. 2026 Oct 27;17(1):916. doi: 10.1038/s41419-026-09256-1 (PMC13720179; doi:10.1038/s41419-026-09256-1)
Supplement: Supplementary file 3 [file 41419_2026_9256_MOESM4_ESM.pdf]

Supple. Fig. 1B-F

	LAMP2	GAPDH	LAMP2	GAPDH	LAMP2	GAPDH
	**Kras/B6**		**KC**		**Kras/B6**	
**MP2/GAPDH**	0,00889468	0,010504854	0,18290745	0,16039191	0,25257386	0,413887808
	0,02176227	0,034050293	0,28294017	0,23533343	0,15415832	0,271149976
	0,04385604	0,060113725	0,56539532	0,46636709	0,365382	0,546184856
	0,16439548	0,262401434	0,11340131	0,09026810	0,22231833	0,335345978
	0,15162367	0,244075977	0,21408984	0,17076929	0,24139702	0,333669992
	0,26350634	0,443998325	0,25227040	0,20436317	0,38044598	0,621704337

	LAMP2	GAPDH	LAMP2	GAPDH	LAMP2	GAPDH
	**Kras/B6**		**KC**		**Kras/B6**	
**ATG5/GAPDH**	0,2271025	0,3081204	0,66007548	0,63864162	0,1288936	0,1886001
	0,1900043	0,3022654	1,05954605	0,96910778	0,0450753	0,1249797
	0,2552433	0,3716208	0,98795781	0,90643557	0,1272932	0,2296165
	0,1205900	0,2341462	0,66022439	0,61249748	0,0783206	0,1363867
	0,3060968	0,3466921	0,38095341	0,36107941	0,1539642	0,2118623
	0,1249409	0,2385702	1,05190077	0,97941652	0,0458709	0,1134107

	BECLIN1	GAPDH	BECLIN1	GAPDH	BECLIN1	GAPDH
	**Kras/B6**		**KC**		**Kras/B6**	
**CLIN1/GAPDH**	1042637,5	26881097	4087204,5	12406796,5	17935844,5	30924809
	1788391	23719677	4823800,5	14004589,5	11889919,5	32922151,5
	1716708,5	21339056	6425508	12764861,5	9662049,5	27611580,5
	1819658,5	24478691,5	8744918	12832200,5	22990854,5	41543890,5
	3079094	27894030,5	8161129,5	14332114,5	16891687,5	30745884
	2436376,5	27133248	10109528,5	13880397	13604762,5	35185651,5

	SQSTM1/p62	GAPDH	SQSTM1/p62	GAPDH	SQSTM1/p62	GAPDH
	**Kras/B6**		**KC**		**Kras/B6**	
**/p62/GAPDH**	54468653	26881097	6631372	12406796,5	14968333,5	30924809
	19194915	23719677	3621997,5	14004589,5	10899580,5	32922151,5
	24452289,5	21339056	2030694,5	12764861,5	20302915	27611580,5
	23752668,5	24478691,5	12840913,5	12832200,5	3815503,5	41543890,5
	46439563	27894030,5	19360348,5	14332114,5	17920915	30745884
	26729946,5	27133248	6438988,5	13880397	6752827,5	35185651,5

	**LC3-I**	**LC3-II**	**LC3-I**	**LC3-II**	**LC3-I**	**LC3-II**
**LC3-II/LC3-I**	**Kras/B6**		**KC**		**Kras/B6**	
	2.836.326	1.331.113	18.798.823	5.549.004	15.455.459	1.461.062
	1.462.234	1.229.234	13.970.510	1.938.790	17.170.066	1.928.255
	2.565.912	997.284	12.107.146	2.173.548	22.634.380	2.377.205
	1.916.134	1.689.891	15.335.974	3.087.569	3.976.418	2.461.861
	3.196.882	2.123.912	17.418.388	3.671.154	14.414.409	3.609.811
	2.768.347	986.627	16.743.953	1.743.891	4.559.660	1.877.134

LAMP2	GAPDH
KC	
0,45951059	0,35682807
1,05153438	0,75281647
0,64119532	0,51428156
0,37064925	0,28221852
0,39970489	0,31612997
0,68712630	0,54428565

LAMP2/GAPDH			
Kras/B6 -	KC -	Kras/B6 LPS	KC LPS
0,00969977	0,17164968	0,33323083	0,40816933
0,02790628	0,2591368	0,21265415	0,90217543
0,05198488	0,5158812	0,45578343	0,57773844
0,21339845	0,1018347	0,27883215	0,32643388
0,19784983	0,19242956	0,2875335	0,35791743
0,35375233	0,22831678	0,50107516	0,61570597

LAMP2	GAPDH
KC	
1,21710946	1,17021269
1,11845407	1,04449049
0,96772472	0,89919331
0,91469689	0,83410594
0,99359965	0,90617235
1,46181466	1,45415012

ATG5/GAPDH			
Kras/B6 -	KC -	Kras/B6 LPS	KC LPS
0,26761142	0,64935855	0,15874687	1,19366108
0,24613483	1,01432692	0,08502749	1,08147228
0,31343203	0,94719669	0,17845485	0,93345902
0,17736809	0,63636093	0,10735361	0,87440141
0,32639446	0,37101641	0,18291322	0,949886
0,18175557	1,01565864	0,0796408	1,45798239

BECLIN1	GAPDH
KC	
8260535	16508250,5
16762549,5	14649554
16990792	13195018
13320599,5	14377811
11146382,5	12323189,5
12898539	13606968

BECLIN1/GAPDH			
Kras/B6 -	KC -	Kras/B6 LPS	KC LPS
0,03878701	0,3294327	0,57998239	0,50038828
0,07539694	0,34444426	0,36115257	1,14423617
0,08044913	0,50337467	0,34992743	1,28766721
0,07433643	0,68148234	0,5534112	0,92646923
0,11038541	0,56942955	0,54939671	0,90450467
0,08979303	0,72833137	0,38665655	0,94793631

SQSTM1/p62	GAPDH
KC	
2574815,5	16508250,5
2223437	14649554
2583547,5	13195018
2227416	14377811
1299598	12323189,5
1769987,5	13606968

SQSTM1/p62			
Kras/B6 -	KC -	Kras/B6 LPS	KC LPS
2,02628088	0,53449511	0,48402347	0,15597143
0,80924015	0,25862932	0,33107133	0,15177506
1,14589368	0,15908473	0,73530434	0,19579719
0,97034061	1,000679	0,09184271	0,15492038
1,66485668	1,35083686	0,582872	0,10545955
0,98513626	0,4638908	0,19191992	0,13007949

LC3-I	LC3-II
KC	
15.403.681	2.320.962
14.286.681	10.059.246
14.178.146	9.670.953
16.869.681	5.476.054
13.265.610	2.575.719
7.721.794	7.721.501

Ratio LC3-II/LC3-I			
Kras/B6 -	KC -	Kras/B6 LPS	KC LPS
0,46930889	0,29517827	0,09453372	0,1506758
0,84065478	0,13877732	0,1123033	0,70409957
0,38866649	0,17952604	0,10502629	0,68210279
0,88192736	0,20132852	0,61911524	0,32460922
0,66436985	0,21076313	0,25043073	0,19416514
0,35639571	0,1041505	0,41168289	0,99996206

Supple. Fig. 2 GSE15471

Controls TLR1	PDAC TLR1	Controls TLR2	PDAC TLR2	Controls TLR3	PDAC TLR3	Controls TLR3
8,344197	7,81262	8,869876	9,920274	5,542414	5,212022	8,024005
8,047195	7,914408	8,983277	10,33843	5,590323	5,182105	7,883575
4,827544	6,789961	6,401563	8,599485	4,801791	5,49727	4,069444
4,234092	6,503404	6,01501	8,517359	4,905068	5,470976	4,101506
4,742425	6,093636	6,313007	8,28302	4,946741	5,642636	4,110841
4,408655	6,185091	6,087641	8,344199	4,784183	5,578837	4,16133
4,976242	5,53295	6,253892	8,102222	4,894419	4,507916	4,03289
7,069351	6,36905	8,441729	9,051476	3,888409	5,243393	7,206167
4,582688	6,822696	6,173516	8,861942	4,973418	5,219476	3,89836
7,291849	7,596438	9,400349	8,673136	4,692474	4,427156	7,717112
5,10875	7,094151	6,847612	8,256311	5,662031	4,960735	5,360116
6,218473	6,384109	7,356924	7,763714	6,06453	5,516212	5,395642
5,217665	6,953383	6,583107	8,828513	6,32615	5,367164	4,888442
5,062517	6,40446	6,624739	8,406732	5,503721	5,15842	4,880601
5,018885	6,280595	6,895051	8,488246	5,116041	5,661374	4,938299
6,831137	6,124559	8,714542	7,791426	5,708098	5,689256	6,42027
4,912119	7,165514	6,482945	8,983355	5,387362	6,072596	4,255222
5,451578	6,491074	6,759487	8,457176	5,122636	6,142815	4,564127
4,405237	7,530907	6,494012	8,884642	5,071205	6,349044	4,239965
4,923654	7,049013	6,616487	8,546302	4,721414	6,003672	4,077811
6,509244	6,290995	7,971593	7,614539	5,628148	5,800251	5,650178
6,802734	7,45386	9,16194	9,753605	4,93866	5,430636	6,879251
5,156632	7,724289	6,363143	10,54377	5,330334	5,059118	4,360322
6,248751	6,699295	7,307182	7,793008	5,566237	5,169004	5,076685
5,22068	6,921072	6,43861	8,369195	5,201671	6,294498	4,284508
3,717104	5,384952	6,387047	7,864898	4,80691	5,75673	4,30121
5,989595	7,044591	6,554599	8,287069	5,339906	5,271876	4,323168
5,38529	6,856572	6,340231	8,41696	5,024922	6,180856	4,126497
6,962098	6,296192	7,743701	8,30437	4,302615	5,67838	7,178605
7,260155	5,875766	9,084989	7,798319	5,76502	5,611166	6,579513
5,051371	5,997192	6,532764	7,460077	6,079817	5,456502	4,721768
5,096753	5,925653	6,45351	7,747007	6,63407	5,301083	4,748635
4,704284	7,022909	6,777761	8,718617	5,12401	5,700168	4,574997
4,166374	5,508555	6,476289	7,551146	5,017489	5,693461	4,380107
4,811212	6,646619	6,337694	8,620215	5,149917	4,837843	3,952386
3,645141	5,644255	7,131227	7,802078	4,488947	5,438266	4,991143
4,46891	6,753638	6,455476	8,978005	5,058389	5,152667	3,94274
4,479806	7,410659	6,786837	8,51939	5,17651	5,521337	4,291408
7,285593	5,630992	7,76455	7,607833	3,949734	5,11501	7,159482

PDAC	Controls	PDAC	Controls	PDAC	Controls	PDAC
TLR4	TLR4	TLR5	TLR5	TLR6	TLR6	TLR7
7,188143	6,520891	6,561492	5,386324	5,211278	6,248325	5,424355
7,272512	6,64762	6,647471	5,607346	5,28642	6,107966	5,652957
6,170016	6,294937	7,304219	4,477106	4,548304	4,708344	5,893735
6,21977	6,320534	7,309866	5,026035	4,560865	4,567286	5,691586
5,727781	5,882636	6,834706	4,50215	4,230224	4,574121	6,118961
5,783712	5,778061	7,034137	4,714957	4,351336	4,249211	6,016088
5,501859	6,161776	7,245404	4,643505	4,304898	4,486619	5,892087
6,538427	6,348182	6,962555	5,018554	4,276171	5,417812	5,429644
6,458978	6,249257	6,866258	4,444716	4,586549	4,406772	5,792421
7,077332	7,258984	6,517419	5,384054	5,690527	5,561892	5,782313
6,885421	5,774212	6,896195	4,509956	4,908613	4,930471	6,189834
5,642388	5,645647	6,155589	4,374661	4,372445	5,207672	5,101304
5,751816	6,142158	7,09894	4,630457	4,465002	5,241108	6,026024
6,477476	6,4537	7,254187	4,581737	4,877959	5,403464	6,256093
5,451113	5,873396	7,193489	4,766048	4,413133	4,212766	5,204756
6,025438	6,653241	7,281749	4,595035	4,256035	6,265347	6,105729
5,962361	6,337303	7,712358	4,358462	4,682947	4,714441	6,74504
5,390263	6,705455	7,976366	4,196883	4,478476	4,969374	6,310829
5,875704	5,657774	7,63821	4,698339	4,700075	4,407524	6,92416
5,578121	6,545914	8,078759	4,456503	4,582511	4,201961	6,404532
5,372119	7,278132	7,347907	4,317661	4,322238	5,427983	5,421922
6,403419	6,704877	7,503509	4,483047	5,113409	5,839624	7,001658
6,282762	6,395765	6,832493	4,537395	4,839162	4,967252	5,160589
5,202638	7,241539	8,273474	4,344719	4,432778	5,770507	5,244771
5,813063	6,122712	7,070593	4,922868	4,700434	4,464742	6,631613
5,357931	6,003032	7,45949	4,300213	4,471932	4,465943	5,391789
5,997682	6,602675	6,812496	4,363586	4,856885	4,578218	6,302579
5,332112	6,576644	7,000411	4,524506	4,522856	4,758245	5,930566
5,977444	6,702218	7,156227	5,626067	4,596282	6,06761	5,301731
5,423903	7,557765	7,028797	4,74667	4,209055	7,365523	5,428437
5,166293	5,829921	7,043049	4,597283	4,480873	5,200193	5,485263
5,323953	5,525543	6,927472	4,371684	4,023471	5,593571	5,785848
5,837627	6,376884	7,62057	4,634986	4,652731	4,446256	6,60611
4,795897	5,65204	7,454461	4,984888	4,369788	4,763001	5,117411
6,739317	6,267499	7,088097	4,431912	4,880568	4,969177	7,346369
5,432517	7,078658	7,531107	4,906274	4,34551	5,20666	6,028419
6,448457	6,395765	7,838979	4,397996	4,896334	4,634053	5,788751
5,940498	6,364111	7,097114	4,596182	4,736629	4,260812	6,495867
5,044998	6,941018	6,845522	5,179568	4,332931	5,962571	5,145679

Controls	PDAC	Controls	PDAC	Controls	PDAC	Controls
TLR7	TLR8	TLR9	TLR9	TLR10	TLR10	CD14
7,830663	7,020634	5,661211	5,211179	7,810822	5,926564	9,825105
7,73887	7,364384	5,572588	5,191474	7,567987	6,275492	9,875826
4,308477	5,362202	5,500706	5,140142	4,761703	4,798315	6,880435
4,29919	5,303208	5,99124	5,335688	4,482671	4,579624	6,800088
4,586138	5,774926	5,633781	5,26101	4,527617	4,558633	6,423577
4,014085	5,871176	6,138189	5,345217	4,394224	4,67211	6,110048
4,343751	5,400134	6,076231	5,199256	4,486877	4,233026	6,702188
6,275153	5,817935	5,701294	5,105387	7,098548	4,6756	9,765453
4,203448	6,557302	5,9351	5,393613	4,478791	4,492931	6,479028
7,170508	6,925669	5,414192	5,702897	5,949276	7,559562	11,09707
5,077749	6,134652	5,328262	5,572776	4,334398	6,230064	9,433372
5,577824	5,231272	5,553133	5,256724	4,754781	4,874372	8,432879
4,839644	5,977067	5,479758	5,442463	4,61144	4,423122	8,518451
4,9794	6,123015	5,672977	5,336482	4,988012	4,875302	8,711594
4,644054	5,026109	5,492106	5,431946	4,091468	4,276183	7,947204
6,272346	5,565078	5,385936	5,236143	4,711592	5,505771	11,30795
4,507424	6,419274	5,967652	5,405246	4,520004	5,144599	7,72663
4,346756	5,469987	5,659747	5,700699	4,340758	4,881044	7,892483
4,544447	5,58579	5,633079	5,238718	4,354898	4,792994	6,762583
4,562676	5,858586	5,97866	5,512559	4,777796	4,725946	7,434511
5,161304	4,736284	5,320972	4,994472	4,815934	5,300809	9,799765
6,717421	7,003078	5,345645	5,660548	4,946217	5,128304	10,73187
4,01854	6,582445	5,374778	5,087504	3,997482	4,348506	7,407964
4,727015	4,622367	5,708675	5,390454	4,483373	4,506631	8,908279
4,245704	5,558124	5,748867	5,14661	4,572603	4,768328	7,151241
4,223224	5,144385	6,18483	5,550557	4,361908	4,337977	6,468007
4,390118	6,272964	5,709281	5,208867	4,555755	5,277552	7,906941
4,541682	5,231337	5,427062	5,127167	4,454776	4,459827	7,117928
6,513176	6,57427	5,447245	4,794712	7,717733	4,498457	10,30946
6,823837	5,004658	5,563281	5,245889	4,954563	4,133697	10,61285
4,82954	5,219799	5,337513	5,590346	4,469456	4,770025	7,318462
4,594845	4,554988	5,299966	5,188342	4,629909	4,527466	7,498231
4,625696	6,500566	6,010942	5,242536	4,176436	5,305413	7,333691
4,405973	4,876156	5,958049	5,510359	4,500085	4,540206	7,367631
4,306947	5,762166	5,957315	5,396104	4,75843	5,121712	6,632727
5,126067	5,140791	6,647742	5,08802	4,732579	4,856784	7,117825
4,312398	6,62049	6,064057	5,647236	4,224241	5,641095	6,786778
4,518162	6,002883	6,170952	5,361378	4,314622	5,827161	7,161746
6,083782	5,273297	5,815786	5,52125	7,704579	5,119384	9,990063

PDAC	Controls	PDAC	Controls	PDAC	Controls	PDAC
CD14	LBP	LBP	MyD88	MYD88	MD2	MD2
11,0067	4,516191	4,581478	10,0198	9,745788	10,09111	9,970914
11,01485	4,417954	4,356647	9,805203	9,615481	10,04388	9,958494
9,843904	5,474848	4,492528	7,634762	9,581798	5,086916	8,966137
9,627554	5,041196	4,361776	7,552259	9,523269	4,751342	8,735068
9,625518	5,069719	4,623454	7,625688	9,832289	4,045627	8,966382
9,459453	5,105162	4,509696	7,699189	9,915296	4,033463	8,89578
8,917298	5,145698	4,463114	7,61519	9,772651	4,698379	8,931372
9,716422	4,708967	4,956959	9,496619	9,06314	10,02423	9,289288
10,6673	4,941958	5,676878	7,688093	9,395518	4,142053	9,157781
10,64886	6,003307	6,052842	10,13736	10,17505	9,807955	10,24887
11,01077	4,677853	5,245062	9,315229	9,938743	7,650853	9,744894
9,519026	4,574075	4,464308	10,16098	9,623098	7,878772	8,429812
10,19064	4,660823	4,794851	9,732506	9,454543	7,855949	9,087577
10,78531	4,672224	4,846297	9,411208	9,166413	8,049383	9,816341
9,340656	4,908286	4,351136	8,018517	9,083337	6,038103	8,502849
9,990549	5,927217	4,588173	10,02588	9,197116	9,977058	9,553132
10,34506	4,709719	4,628076	8,106933	9,585871	5,354613	9,279219
9,674463	4,911019	4,626952	7,857157	9,205763	6,002904	8,441935
9,866359	4,887037	4,203327	8,046488	9,438864	3,710644	9,353016
9,962736	5,041878	4,81851	7,834042	9,108949	5,194539	8,757507
8,767459	4,642644	4,451751	9,179219	9,039977	8,843633	8,621935
10,98004	5,231699	5,125594	9,47705	9,689016	9,932288	9,747196
11,86707	4,879958	4,428527	8,081835	9,866212	5,0207	9,097554
9,109979	4,835029	4,509179	8,300917	9,509435	8,016143	8,602227
9,831961	4,877416	4,257121	8,046955	9,517202	5,931973	9,455897
8,619678	4,966448	4,530129	7,645793	9,20227	4,262592	7,779605
10,34576	4,560163	4,880505	7,938449	9,841636	5,698415	9,30128
10,05474	4,889635	4,471155	7,905507	9,628859	5,385582	8,72854
9,565249	5,546633	4,413695	9,590631	10,01497	9,964248	8,873833
9,452424	4,678882	4,414935	9,371019	9,393786	9,641136	8,919301
8,93111	4,72147	4,538382	9,735785	8,981536	6,343057	8,27222
9,170408	4,768369	4,394896	9,862158	9,02744	7,362381	8,097809
9,967041	4,853328	4,422323	7,89451	9,593976	4,372068	8,692709
8,841484	4,900875	4,667871	8,12414	9,321504	5,265861	7,689415
10,91028	5,267254	6,29194	7,922545	9,550013	4,976483	9,950334
9,198228	6,311351	4,238863	7,006027	9,826193	4,574732	9,000027
10,90126	4,945692	5,295787	7,672452	9,592122	4,447504	9,623901
10,36062	4,915629	5,961039	8,156204	9,547285	4,805868	9,417989
9,477488	4,583657	5,294045	9,622464	9,095011	9,660571	8,451852

Controls NOD1	PDAC NOD1	Controls NOD2	PDAC NOD2
7,612339	7,4608	5,097115	6,058438
7,417991	7,389744	5,330989	6,216332
6,605207	7,060465	4,219859	4,962244
7,011698	7,041723	4,342968	5,146538
6,608365	6,785679	3,676908	5,518725
6,639895	6,942171	3,872111	5,142495
6,752067	6,960145	4,172807	6,483586
7,159745	6,903029	4,133285	4,475266
6,870186	6,712833	4,098879	4,664929
7,440694	7,250825	6,035527	5,609848
6,478643	7,168469	4,25102	5,331558
6,386194	6,93616	4,807981	5,252805
6,368862	7,19775	4,326859	4,821179
6,098638	7,210807	4,490058	4,550684
6,318679	6,974946	3,466335	4,899336
8,037094	7,438489	5,373909	5,295688
6,574417	7,196465	3,74439	5,212954
6,585057	6,883088	3,578563	4,677746
6,52484	7,186568	3,542764	5,148641
6,618024	6,699552	4,042785	4,564056
7,01749	7,140986	4,52307	4,400529
7,177752	7,493422	5,555639	6,126389
6,259053	7,063216	3,711263	7,68993
6,674522	6,614041	3,902836	5,858895
6,755443	7,051143	3,915431	5,44296
6,704697	6,747581	4,171397	4,271037
6,387232	7,349111	3,627482	5,714372
6,537028	7,078144	3,897323	5,119537
7,173906	6,913837	4,561245	6,242
6,878385	7,285715	5,353666	5,015081
6,51325	6,781363	4,432305	4,26529
6,410885	6,872986	4,248719	4,593117
6,659788	6,894284	3,840955	5,629041
6,621303	6,76935	3,628302	4,101044
6,707271	7,323833	4,119447	4,61999
6,913047	7,235579	4,61999	5,229924
6,462074	7,650588	3,850198	5,048515
6,819444	7,22122	4,193751	5,325996
7,453055	7,153292	4,722389	4,542622

Supple. Fig. 2 GSE56560

Controls TLR1	PDAC TLR1	Controls TLR2	PDAC TLR2	Controls TLR3	PDAC TLR3	Controls TLR3
4,119398	5,175157	4,468874	5,163148	4,919634	5,226735	4,752587
4,122526	4,822791	4,564985	5,670445	5,076452	5,369354	4,607524
4,17653	5,027415	4,82273	5,677066	5,01502	5,507009	5,26854
3,581159	5,405498	4,131756	5,921056	4,713936	5,844125	4,598255
3,643561	5,030699	4,181021	6,027484	4,443144	4,327182	4,766994
3,571867	3,971749	4,360724	4,964189	4,813922	4,540386	4,611043
	4,855736		5,992647		5,285097	
	4,305616		4,821063		5,03191	
	4,730235		5,059533		4,413641	
	4,093751		5,105929		4,704826	
	4,33023		5,146065		4,828612	
	4,704292		5,337866		5,390523	
	4,980477		5,922544		5,617641	
	5,339626		5,306294		5,770525	
	4,247864		5,300887		5,027083	
	5,11064		5,5709		5,008927	
	4,605835		5,156365		4,899763	
	4,977756		5,46789		5,415569	
	4,255337		5,638709		4,842352	
	4,645606		5,949176		4,900082	
	4,125993		5,513619		4,615604	
	3,831161		4,58523		4,921096	
	4,895896		5,548362		5,089405	
	3,936104		4,563692		4,962107	
	4,389035		5,249597		4,783875	
	4,961165		5,290382		5,262069	
	4,923682		5,781078		4,554525	
	4,003115		4,935868		5,002816	

PDAC	Controls	PDAC	Controls	PDAC	Controls	PDAC
TLR4	TLR4	TLR5	TLR5	TLR6	TLR6	TLR7
6,083075	5,10854	4,99397	3,524686	4,780782	3,53435	4,555993
5,454199	5,059167	5,402391	2,711548	4,629934	3,40203	3,836521
6,283339	5,111038	4,825269	3,851997	5,10768	4,479104	3,961351
6,165801	4,895579	5,429311	3,524619	4,40344	3,499874	5,009463
6,518684	5,01683	4,591555	3,704243	4,887923	3,338927	4,267132
5,525284	4,62076	4,744941	3,17427	4,100537	3,437498	3,540304
5,86237		5,21655		4,766186		4,665548
5,906599		5,011315		4,696529		3,960031
5,646139		4,664121		4,911758		4,584831
5,631997		4,595213		3,898245		4,081279
5,712273		5,110117		4,248766		3,862364
5,782641		5,194738		4,359764		4,835749
6,699296		5,091668		4,901314		4,411715
5,949399		5,228657		4,353728		4,866963
5,88735		5,064518		4,028571		3,976455
6,029238		5,010835		4,689613		4,76269
6,360755		4,694944		4,065102		4,360158
5,668376		5,112801		4,734609		4,210075
5,987307		4,762234		4,488456		4,255095
6,099765		5,041434		4,761756		4,186358
5,971952		4,844916		4,352214		3,97328
5,75283		5,036461		3,975201		3,923047
5,926008		4,995376		5,247982		5,195537
5,253482		4,590368		3,464035		3,429936
5,725567		4,929439		4,235868		4,425942
6,025976		5,499803		4,602488		4,138216
6,302891		4,914588		4,830459		4,695205
5,283026		4,983055		4,019204		3,861266

Controls	PDAC	Controls	PDAC	Controls	PDAC	Controls
TLR7	TLR8	TLR9	TLR9	TLR10	TLR10	CD14
3,778065	3,734051	5,211782	4,402266	3,22018	3,065681	6,126774
3,383071	3,571547	4,885002	4,373292	3,129269	3,593232	6,187609
4,074792	4,155792	4,62046	4,489202	3,235095	4,754077	7,418577
3,688467	4,073999	5,217921	4,496948	2,964922	3,581822	6,451114
3,585735	5,151669	4,931078	4,430274	3,308389	3,943684	6,735248
3,74326	3,821268	4,965074	4,434695	3,388665	2,935382	6,066708
	4,106013		4,514996		4,284057	
	3,75951		4,477892		3,688484	
	4,690978		4,808053		4,740384	
	3,82888		4,55171		3,102206	
	3,68975		4,340249		2,995426	
	4,158969		4,420385		3,395663	
	4,352138		4,441361		3,749482	
	4,140626		4,097493		3,515321	
	4,046949		4,940188		3,312841	
	3,881078		4,578569		4,372827	
	4,10712		4,742534		3,663665	
	3,925779		4,054527		3,482212	
	4,097937		4,702183		3,195234	
	4,670785		4,549592		3,849574	
	4,022649		4,762591		3,387267	
	4,015536		4,264414		3,163954	
	4,595968		4,782047		4,092058	
	3,642821		4,800927		3,301571	
	4,719544		4,806462		3,635634	
	3,82693		4,564997		3,770699	
	4,735659		4,442359		3,599213	
	3,776611		4,718903		3,102652	

PDAC	Controls	PDAC	Controls	PDAC	Controls	PDAC
CD14	LBP	LBP	MyD88	MYD88	MD2	MD2
7,411158	4,768621	3,930996	5,244142	6,012375	3,089145	4,426691
7,105674	4,326516	3,308653	5,01099	5,826202	3,253785	3,720513
7,622515	4,474098	4,015225	5,766451	5,993933	3,739119	4,041294
7,775951	4,459126	3,605918	5,327277	6,201734	2,997724	3,909749
7,864391	3,933622	4,478791	5,400144	5,957686	3,364212	4,06219
7,418318	4,360933	3,784771	5,051102	6,408921	3,078627	3,672756
7,389328		3,98253		5,975119		3,865938
6,870295		3,842173		6,319695		3,117383
7,527371		3,94958		6,091384		3,920795
7,3072		3,759269		5,99898		3,362839
7,325485		3,810177		6,217789		3,820753
7,662705		3,993553		6,362919		3,808615
7,793637		3,756188		6,303382		4,453857
7,562018		3,825822		5,946613		3,981956
6,720255		4,376659		5,807721		3,233913
7,501342		3,831061		5,911575		4,008514
7,655286		4,194317		5,773163		3,561602
6,552698		3,671024		5,803252		3,453579
7,719828		4,741307		6,088317		3,855133
7,797323		4,083908		6,025039		4,406162
6,596259		4,556084		6,115745		3,543269
6,713635		3,6112		6,251938		3,473772
7,71636		4,351568		6,193921		4,156789
6,214175		4,158845		6,246131		2,922992
7,369702		4,195732		6,264453		4,234486
7,079927		4,648173		5,967428		3,751363
7,726051		4,178758		5,990135		3,908313
7,206337		4,026662		6,171036		3,644734

Controls NOD1	PDAC NOD1	Controls NOD2	PDAC NOD2
4,435311	4,601913	4,198715	4,146518
4,511475	4,898682	3,882535	4,110964
4,701992	4,72826	4,254352	4,260923
4,508012	4,77917	4,344091	3,954744
4,712237	4,755298	4,014575	4,22019
4,365862	4,82131	4,31201	4,434962
	4,747842		4,267175
	4,872318		4,477682
	4,85155		4,760114
	4,760636		4,193004
	4,934493		4,079032
	4,847215		4,237907
	5,146313		4,565224
	4,510356		3,979262
	4,620988		4,223244
	4,89665		4,212643
	5,153626		4,199144
	4,880543		4,192039
	4,96297		4,434438
	4,913033		4,477301
	4,775722		4,00494
	4,797144		4,401441
	4,974321		4,410871
	4,713618		3,952706
	4,789991		4,345111
	4,952986		4,247303
	4,901612		4,830851
	5,139077		4,139444

Supple. Fig. 2 GSE62165

Controls	PDAC	Controls	PDAC	Controls	PDAC	Controls
TLR1	TLR1	TLR2	TLR2	TLR3	TLR3	TLR3
6,322353	6,407509	5,637414	5,858788	6,133836	6,110347	5,148751
5,708595	6,679232	5,368259	6,716261	5,784797	4,418828	4,487721
7,854062	6,947789	6,396998	6,17823	6,679302	6,205608	5,544959
6,642626	7,228802	5,511284	6,139105	5,773076	5,54633	4,83983
5,784935	6,700243	5,096363	5,618329	5,495275	5,865997	4,872518
5,364732	6,806209	5,314107	6,857571	5,929949	6,065025	4,629847
5,891809	7,115031	5,345551	5,962217	5,055287	6,281875	5,247726
6,769815	7,116844	5,766805	7,067078	6,142431	5,4893	5,249651
6,847271	5,958912	6,077795	5,658422	4,153475	5,870603	5,408358
6,050654	6,663855	4,947615	7,122333	5,735619	4,712515	4,149758
5,609566	7,059344	5,644684	6,114946	5,456557	6,613456	4,894787
5,057255	6,49726	5,423141	6,649512	5,736996	6,105859	4,331183
6,808163	7,223136	6,336596	7,14524	6,301406	6,247664	5,035389
	6,873137		6,253395		5,99707	
	7,124985		6,068213		6,042301	
	6,377992		5,789226		5,76024	
	6,842835		6,607683		5,131165	
	7,53315		6,609691		6,045042	
	6,522104		5,797773		6,446687	
	5,960505		6,101651		5,439841	
	5,983778		5,519668		6,472426	
	5,917555		6,230371		4,437531	
	7,369962		6,282644		6,145361	
	7,078822		6,507334		4,688356	
	7,725167		6,409138		4,459674	
	6,816876		6,534864		5,959006	
	8,07966		6,446522		7,356484	
	7,386538		5,941295		5,095123	
	6,978784		6,464351		6,782476	
	7,219014		6,933858		5,16205	
	6,819778		5,92969		6,269675	
	7,050223		6,827271		5,914032	
	6,426351		5,83703		6,35302	
	6,624677		6,082217		6,176824	
	6,098421		5,924883		5,965803	
	6,704431		6,08683		5,562836	
	5,829556		5,925254		5,687908	
	7,583451		6,474935		6,305303	
	6,655932		6,09939		6,143496	
	5,778701		5,587364		5,828121	
	6,952603		6,741451		6,38251	
	6,225549		5,781426		5,821896	
	7,260913		6,568993		6,341664	
	6,472059		6,25437		4,233055	
	7,272946		6,246727		5,40245	
	7,290311		6,608453		5,740862	
	5,776137		6,004527		5,846091	

7,53554	6,864587	6,134706
7,102392	6,5231	5,632081
7,706232	6,225904	6,193325
7,373982	7,089335	5,263384
6,495735	6,824367	6,12578
7,227775	6,59464	5,582145
7,438186	6,644337	6,875244
5,954717	6,325774	5,700998
6,935267	6,121597	5,567751
7,332688	6,330057	5,556932
6,621395	6,275413	6,016873
6,635514	5,680145	5,040999
6,798231	6,360364	6,617025
7,042843	6,445528	5,836485
6,448885	6,293285	5,433642
6,874957	6,100118	5,862105
6,473356	6,182887	5,706465
6,38885	6,444484	5,684864
7,695993	6,269584	6,419344
7,534692	6,217794	6,743262
6,992315	6,483802	5,533152
6,928923	6,492398	5,355444
7,220401	6,755664	5,839171
6,525656	6,125272	6,204453
7,628903	6,349148	6,950714
7,027699	6,078232	5,972232
8,838953	6,802423	5,582299
5,873798	5,520843	5,371886
7,184918	5,939206	5,703093
6,246412	5,672525	5,684791
6,708201	6,002002	6,056235
6,622309	5,789974	5,736346
6,794488	6,396318	5,838915
5,639297	5,654439	3,838539
5,894469	6,29221	5,404583
7,617683	6,657859	6,874658
5,212987	6,152989	4,41539
7,173643	5,942829	4,977977
6,380072	5,762722	6,648828
7,4201	6,015159	5,632829
6,12434	5,89308	4,917567
7,78611	6,696729	6,491385
6,503795	6,294915	5,55344
6,933008	6,261765	5,007695
6,852883	6,139507	5,997692
6,650248	5,857208	6,238922
6,031034	6,453206	5,088008
7,474538	6,56901	6,016863
7,414376	6,145327	6,665677
6,756897	6,311437	5,601991

7,580756	6,966493	6,686544
6,639577	6,30365	5,450648
7,127923	6,941213	6,167423
7,250756	5,627761	5,816266
6,8754	6,645385	6,490539
6,823663	6,810012	5,539494
6,956636	6,115938	5,887582
7,609622	6,637725	5,705522
6,271999	5,979975	4,180543
6,73421	6,073865	5,934857
7,664308	7,26899	6,266126
6,458073	5,85784	6,037565
6,50488	6,174545	5,629077
7,12206	6,433986	6,166909
6,073876	5,264078	5,504927
8,073712	8,140113	5,889637
6,685786	6,410899	5,520123
6,608666	6,152896	6,273913
6,118851	6,278839	5,937015
6,709828	5,882598	6,042819
7,221172	5,775096	6,341025

PDAC	Controls	PDAC	Controls	PDAC	Controls	PDAC
TLR4	TLR4	TLR5	TLR5	TLR6	TLR6	TLR7
5,409501	7,1025	6,535269	3,544049	3,339335	3,421935	4,282608
6,366157	6,628824	6,130412	3,258018	3,3707	3,267267	3,902597
5,725738	6,415324	6,419463	3,536573	3,433028	4,281996	4,439305
5,899048	6,397347	6,594044	3,729748	3,452336	3,62586	5,722237
5,622545	5,102291	7,048276	3,562511	3,324568	3,495375	5,587554
5,403909	6,271091	6,552946	3,334015	3,368764	3,366451	4,887165
5,73766	5,378895	7,334508	3,561822	3,705567	3,16266	4,554607
6,525518	7,01672	6,670219	3,332735	3,540535	4,815923	4,140026
4,78731	5,977493	5,561405	3,502741	3,457661	4,96533	3,573814
6,050712	6,03873	6,932616	3,962286	3,501051	3,452811	4,279023
5,372356	6,071849	7,22	3,317376	3,353525	3,559486	4,546946
5,495619	5,050322	5,093009	3,842247	3,731837	3,419554	3,952496
7,037363	7,107133	6,721693	3,598511	3,863529	4,543314	4,807369
6,016788		6,496439		3,832007		5,474461
5,25188		8,003223		3,431703		5,194198
5,97637		5,219396		3,692714		4,690152
6,081453		6,423358		3,438908		5,453959
6,195728		6,672589		3,942031		5,842386
5,474676		7,607559		3,523102		4,972173
5,532431		6,765443		3,352983		4,602001
5,990474		6,352096		3,35252		4,437862
5,545323		5,763521		3,403077		3,735523
5,449834		8,146866		3,651792		5,096376
6,560907		6,264446		3,97629		5,845056
5,847364		4,501914		3,894871		5,111019
6,061688		6,05312		3,486515		4,116111
6,262484		6,795197		3,47842		4,747161
5,216519		4,659144		3,705587		3,797148
6,0549		5,309585		3,610173		4,852934
6,940202		7,41719		3,593288		5,888171
5,708222		7,122496		3,531023		4,0314
6,482228		7,17382		3,479551		5,921241
5,098833		6,976781		3,405414		4,282738
5,348423		6,332258		3,321363		4,713699
5,265643		7,002774		3,379749		3,995778
6,013607		5,814285		3,467382		4,961721
5,052565		6,443847		3,263134		3,850836
6,08808		7,498261		3,785854		6,358941
5,899756		6,187698		3,748035		4,752055
5,892848		6,266438		3,35992		4,012149
5,091769		6,898247		3,486901		4,997422
5,460059		6,144815		3,302363		4,408463
6,579365		7,140902		3,623354		5,849299
5,78499		6,142922		3,290666		4,555871
6,157715		6,670283		3,387727		5,590236
5,767693		5,568874		3,365896		3,902085
5,902598		5,582965		3,310939		4,24145

7,145546	7,284533	3,838761	4,5195
6,288408	7,387278	3,596876	5,503536
6,928323	6,647996	3,778567	5,017023
6,477061	7,77665	3,476257	5,047843
6,008245	6,481699	3,475941	4,053647
6,658125	7,283798	3,483468	6,272558
5,9446	6,639468	3,637925	6,068595
5,419099	7,100483	3,352973	4,227802
6,108769	6,296109	3,437916	4,85447
6,232025	7,007196	3,525097	5,669007
6,287224	6,122038	3,260599	4,677537
5,445802	6,147612	3,44536	3,767459
5,616944	6,900676	3,368286	5,524738
6,116475	6,44049	3,673542	4,668658
6,230589	6,761808	3,444288	5,358512
6,286102	6,806949	3,82087	5,806019
5,439404	6,880525	3,379135	3,578756
5,976842	6,2986	3,467774	4,568395
6,479796	5,387287	4,005998	4,108072
5,811089	8,373522	3,570716	5,330238
5,561616	6,453365	3,557465	4,608196
5,292464	6,720459	3,522328	3,932422
5,969799	7,196239	3,448282	5,305198
5,684184	6,252298	3,56442	4,753426
5,827905	7,50413	3,429809	5,291101
5,440255	4,882385	3,679971	3,519671
6,014286	8,861173	4,429236	5,333593
4,845496	6,834785	3,340318	4,013815
5,920504	5,462305	4,138061	5,52056
4,775069	6,666982	3,303072	4,008472
6,366382	7,137429	3,543623	4,91967
5,196693	6,771225	3,401967	4,211062
6,137075	6,032424	3,449355	5,121235
4,971908	6,522775	3,423013	3,094498
5,324361	5,794069	3,279597	3,954714
5,6917	7,67574	3,452549	5,055763
4,628362	7,405341	3,489168	3,823007
6,168116	5,70214	3,582676	4,328731
5,918131	6,59833	3,538772	3,893377
6,135888	7,006434	3,73515	5,341345
5,649068	6,054747	3,552456	4,418639
6,226174	6,710209	3,659813	5,790408
6,391941	6,853505	3,691921	5,962606
5,973954	6,22505	3,520558	6,075768
5,916157	6,769047	3,42439	5,795953
5,166709	6,807775	3,487273	4,868422
6,28545	6,91094	3,257772	4,191522
5,878519	7,411699	3,947297	4,968733
5,759247	7,032935	3,40256	5,408924
6,4818	6,903506	3,69274	4,782673

6,665837	6,508634	3,61231	4,216453
5,958821	6,058732	3,603392	4,31493
6,56622	7,328744	3,427874	5,770753
5,968906	6,916791	3,451414	4,686163
5,672027	7,221453	3,432287	4,727171
6,269706	6,105791	3,383917	4,582802
5,743521	6,480929	3,484395	4,748162
6,472618	7,01657	3,593176	6,191855
5,58493	6,595954	3,321354	4,338555
5,503443	6,480941	3,504429	4,66673
7,193872	6,760198	3,874353	5,861335
5,911843	5,797016	3,349741	5,231051
4,91926	7,1338	3,563262	4,289121
6,085739	6,784697	3,503462	5,288971
5,596244	5,20812	3,465568	5,307246
6,922484	6,606278	4,045213	6,495183
5,983003	6,645769	3,603304	4,894824
5,714788	6,862816	3,396934	5,844717
5,750284	6,269805	3,522971	3,654446
4,801291	6,660155	3,46521	4,414115
6,855098	5,68909	3,370596	4,606415

Controls	PDAC	Controls	PDAC	Controls	PDAC	Controls
TLR7	TLR8	TLR9	TLR9	TLR10	TLR10	CD14
3,922255	4,613689	3,322731	3,105887	3,247083	3,141047	6,748646
3,695171	4,542565	3,050104	3,113603	2,898883	3,114105	6,847068
4,974648	4,565414	2,953656	3,143489	3,190588	3,179668	8,30383
3,665613	4,633952	2,93974	3,098307	2,979496	5,016386	7,147579
3,705662	4,881898	3,211464	3,160995	2,92262	4,26189	6,055993
4,217088	4,839554	3,433862	3,090299	2,86971	3,411352	7,73978
3,737721	4,698808	3,338206	3,005094	3,064674	3,04921	7,125162
3,826104	5,370974	3,552497	2,882422	3,200471	3,357695	7,820776
4,283746	3,756344	3,66457	3,059237	5,399433	3,043694	8,176546
3,79989	4,687117	3,308381	2,987209	3,009698	3,202575	5,066224
3,893433	4,41317	3,207344	2,870717	2,890814	3,528082	7,543831
3,673963	4,455126	3,176602	3,182589	2,762745	3,162365	5,407565
4,707836	6,811019	3,128655	3,098239	3,041021	3,799972	8,255067
	4,90448		2,981962		3,987356	
	4,702303		3,264899		3,411276	
	3,780107		3,015046		2,891199	
	4,882072		2,880951		3,537346	
	5,225752		3,014236		3,619133	
	4,426633		3,114126		3,252262	
	4,800609		3,141867		3,319557	
	4,054965		2,98503		3,248686	
	3,806773		3,188339		2,840034	
	4,627531		2,961727		4,603927	
	4,748441		3,533247		5,567807	
	4,904213		3,233511		4,137521	
	4,675866		2,935026		3,199404	
	5,247969		3,088202		3,278358	
	4,194367		2,999405		2,849473	
	5,894429		3,069928		2,980689	
	6,537596		2,802388		4,18051	
	4,329787		3,055522		2,973803	
	5,593507		2,815275		4,160213	
	4,205363		3,346331		3,81121	
	4,443206		2,965125		2,97876	
	4,067639		3,088966		3,481671	
	4,972036		3,053371		3,444936	
	4,018395		2,950301		3,101308	
	5,235862		3,1501		3,472491	
	4,774604		3,103231		4,935627	
	4,129788		2,963095		3,076179	
	4,636682		2,891924		3,246446	
	4,655658		2,93256		3,121393	
	6,302352		3,020533		3,701031	
	4,829304		2,874192		3,15966	
	5,46427		3,150512		3,195599	
	4,406421		3,334759		3,228338	
	3,775527		3,025699		3,114118	

6,169341	2,935502	3,281309
4,881288	3,218805	4,102694
5,539507	2,933163	3,622429
5,959077	3,192974	3,279611
4,481245	3,038261	3,06207
5,698308	3,04456	4,039399
5,204754	2,956001	3,441881
4,41213	2,956699	3,113921
5,895063	3,000894	3,550592
5,514709	2,915256	4,570694
4,559189	3,06261	2,972524
3,971894	3,156162	3,049503
4,881306	3,144371	3,710717
4,877155	3,032001	2,94186
4,970983	3,035691	3,972034
5,600783	3,108117	5,692883
4,293313	3,090299	3,033049
4,381478	2,840421	3,031632
4,801731	3,274303	3,326082
4,308555	3,04759	3,748922
4,513105	3,164112	3,873775
4,29721	2,888605	3,01908
5,413172	2,874698	4,068945
5,027832	3,146165	4,410241
5,275544	2,850044	3,653448
4,378419	3,078479	2,978483
4,791658	3,082552	3,898063
3,694774	3,106234	2,927459
4,914518	3,370083	4,803005
3,886841	3,065751	3,17507
4,301277	3,306357	2,874135
4,289309	2,983549	3,147593
4,789866	3,278472	3,162187
3,684048	3,094109	2,790395
4,014106	2,652011	3,011761
5,195062	3,180053	3,23737
3,542152	3,158334	2,964999
4,166538	2,910901	2,901192
3,877394	3,149353	3,000055
4,995303	3,240965	5,469797
3,964224	3,239504	2,852197
5,918131	3,015799	4,273918
5,036692	3,035698	3,708062
4,947612	3,15558	2,954349
4,736962	3,147372	3,486599
4,289691	3,494006	3,010218
4,149282	2,874161	2,974912
5,491268	2,88112	3,473077
4,496733	3,253281	3,154382
5,865373	2,935123	4,549224

4,794748	3,075373	2,795187
5,304137	3,02686	4,38268
5,685444	2,912185	3,59627
4,30529	3,231709	3,206606
4,806603	3,090299	2,911735
5,092293	3,125618	3,485671
4,336556	3,25348	3,538478
5,442379	3,131781	3,210699
4,446195	3,159957	2,917311
4,389699	3,781781	3,582343
6,410392	3,169887	3,578385
4,229549	3,148158	2,784092
4,254911	3,159282	2,94664
5,3059	2,990206	3,108603
3,775189	3,26959	2,800444
7,396903	3,218956	3,652131
5,051559	3,233914	3,266
4,531116	3,189604	3,129071
4,037281	3,042625	2,938927
4,048944	3,110128	2,979954
4,740829	2,966919	3,247272

PDAC	Controls	PDAC	Controls	PDAC	Controls	PDAC
CD14	LBP	LBP	MyD88	MYD88	MD2	MD2
8,577549	3,149464	2,861446	4,405459	4,39507	6,950484	7,453391
8,931213	2,978063	2,816217	4,157295	4,572243	4,688875	8,507237
9,165388	3,263192	2,740372	4,549395	4,784275	7,902075	8,058581
9,859573	3,014621	2,749505	4,426599	4,456378	6,633943	8,193029
9,045088	3,085965	9,788319	4,420578	4,258589	5,437009	7,667635
8,947513	2,800013	2,822813	4,271569	4,396645	6,432661	7,698813
9,266022	3,647192	3,760283	4,28388	4,428467	5,228977	8,477758
9,958633	2,69096	2,876122	4,487001	4,720939	6,696238	8,719729
8,152517	2,819323	2,71868	4,453914	4,266022	8,086269	7,833147
10,08427	2,96199	2,714997	4,194078	4,238047	4,585989	8,314512
8,816276	2,890866	2,849248	4,708703	4,69203	4,845104	7,693801
9,346849	4,048356	2,665439	4,546564	5,103255	4,014998	7,564592
9,940877	3,054469	3,088645	4,625056	4,990301	6,522374	8,609263
9,582141		3,472367		4,640945		8,429792
8,664301		2,949811		4,742112		7,46547
8,339876		2,967936		4,847315		7,215026
9,231548		3,047577		4,507123		8,453606
9,758793		2,993897		4,536215		8,855533
9,000167		2,707652		4,597015		7,846719
8,468526		2,816528		4,305038		8,481308
7,999549		2,718135		5,136014		7,508299
9,137361		2,720542		4,293688		8,305793
8,871347		2,87838		4,555729		8,430115
10,20246		6,458259		4,68915		8,878924
8,908271		3,591644		4,619717		7,775245
9,49431		2,715777		4,801059		8,23401
9,418769		2,967112		4,809128		8,46933
8,405378		2,814587		4,549346		7,498522
9,629976		2,722646		4,285927		8,728716
9,822826		2,643763		4,826282		8,934952
8,589713		2,649465		4,434704		8,242683
10,22092		2,565981		4,311591		8,929468
7,608882		2,977504		4,452774		7,801883
9,21823		3,127474		4,432105		8,074047
8,136478		2,970338		4,505389		7,746137
9,252453		3,276402		4,611728		8,353024
7,929016		2,696259		4,492729		6,832103
9,271611		2,813654		4,409645		8,624839
8,65463		3,473419		5,130888		8,152828
8,300914		2,831469		4,661339		7,473587
9,266759		2,944545		4,402801		7,759522
8,161076		2,906945		4,730431		7,900072
9,090791		3,10554		4,61633		9,13057
9,866969		2,853971		4,417281		8,94619
9,521463		2,797248		4,429028		8,054539
8,99124		2,896106		4,589411		8,231947
8,440452		2,723121		5,027392		7,477688

9,361269	2,877217	4,616247	9,241431
9,381923	2,944737	4,515731	8,399708
9,136547	2,571059	4,554768	8,791814
9,481876	3,154875	4,760593	8,290865
9,21008	2,853241	4,21636	8,450845
9,862703	2,802399	4,406675	9,843537
8,907071	2,956921	4,323465	8,667578
8,03906	2,899603	4,396216	8,136293
9,273737	2,900874	4,897564	8,078382
9,253537	3,13357	4,749235	8,600639
9,303208	2,88738	4,703232	8,28269
8,410091	2,664157	4,333879	7,46346
8,656119	2,711998	4,637736	8,591622
8,450628	2,808882	4,224831	7,917039
9,132328	2,845933	4,546364	8,715772
9,023784	3,309819	4,46219	8,996601
8,759289	3,016954	4,829549	7,846871
9,169993	2,679449	4,331257	8,15549
8,866157	2,854839	4,653029	8,352208
8,618919	2,955898	4,413347	8,618632
9,084063	3,04136	4,643287	8,071474
7,729534	2,743405	5,013293	7,276799
9,542173	2,898309	4,545781	8,64549
8,913176	2,730541	4,416196	9,034078
8,664474	2,898809	4,39098	8,870074
8,73718	3,032957	4,346294	7,53981
9,063278	3,828693	4,946847	8,66813
7,518883	2,728079	4,473598	7,211286
9,044486	3,272646	5,037996	7,77734
8,460496	2,852786	4,387012	7,597149
8,58067	3,063894	4,575	8,935012
8,435054	2,713782	4,702045	7,417663
9,407412	2,746932	4,716949	8,216102
7,655844	2,713399	4,375886	7,948515
9,108976	3,055431	4,17584	7,31477
8,811662	2,906988	4,381177	8,178836
7,722278	2,547247	4,373354	7,603087
9,199266	2,791276	4,889751	8,681719
8,209442	3,130683	4,729462	7,314951
9,49997	3,213119	4,439526	8,486228
9,220006	2,702882	4,426714	8,373362
9,497456	4,207156	4,517135	8,302857
9,050897	2,852784	4,486089	8,87782
9,704008	2,62707	4,143424	7,946736
8,656618	2,799813	4,483685	9,252682
8,074374	2,895057	4,712999	7,45646
8,168561	2,958132	4,161264	7,722348
9,186536	2,563883	4,400122	8,787382
8,632149	2,919265	4,450151	8,404381
9,075313	2,674365	4,611295	8,329665

8,934452	2,765935	4,542115	8,720199
9,153941	3,488547	4,558868	8,207645
8,859633	3,139453	4,317092	9,121882
8,185445	2,673705	4,237296	7,938018
9,443234	2,660937	4,366221	7,966163
8,292511	3,174135	4,850733	8,262055
9,192549	2,607518	4,833755	7,705468
8,982043	2,786216	4,53103	8,970534
8,766281	2,534765	4,821015	7,973434
9,491037	3,157711	4,756851	7,862501
10,18051	2,745155	4,603085	9,047286
8,586604	2,725342	4,817454	8,103932
8,758032	2,643642	4,534318	7,580803
9,008811	2,891992	5,060011	8,401607
7,867568	2,865669	4,184313	7,951928
10,71953	2,752996	4,62812	9,411279
8,665737	2,713328	4,427167	8,490048
9,243857	2,656647	4,298276	8,773521
8,617506	3,059957	4,624933	8,338835
8,194274	3,01922	4,617471	7,083207
8,172002	2,825897	5,040128	7,299709

Controls NOD1	PDAC NOD1	Controls NOD2	PDAC NOD2
4,459077	5,079203	3,504683	3,722819
4,935435	5,478846	3,638208	3,769031
4,609631	4,820906	3,209866	4,294378
5,161307	5,031377	3,366665	4,206876
4,880109	4,544192	3,268463	3,51581
4,610942	5,172661	3,468076	4,261082
5,012722	4,651714	3,206167	4,302952
4,922092	4,941204	3,615981	4,195231
4,857062	4,56167	3,78213	3,633557
4,978715	5,808675	3,208364	4,083413
4,552783	4,765106	3,566337	3,658425
4,574923	5,30073	3,120094	4,63984
4,668651	5,426868	3,45846	4,7782
	5,02421		3,707847
	4,579375		3,729595
	4,521072		3,649023
	5,495281		3,857238
	5,052799		4,199697
	4,421733		3,690313
	5,132054		4,21094
	4,705839		3,809606
	5,517447		4,479028
	4,746594		4,128848
	5,834514		3,908168
	4,592655		4,276375
	5,556735		4,297675
	4,826774		3,925059
	4,929914		3,881581
	4,832191		3,871938
	5,800623		5,078044
	5,062885		4,078935
	5,087275		4,456849
	4,666037		3,678946
	5,250845		4,225182
	4,847081		3,490632
	4,739784		4,34965
	5,384593		4,166027
	4,696313		4,066152
	4,807545		3,958899
	4,604632		3,69194
	5,005717		4,022953
	4,752835		4,138537
	5,177439		4,636755
	5,513461		4,56948
	4,730275		4,632013
	4,953388		4,605683
	5,059521		4,281224

5,069113	4,440883
4,944985	4,018641
4,889414	3,990948
5,67983	4,99505
5,296785	4,941041
5,125709	4,177898
4,84228	3,929877
5,507232	4,058404
4,760784	4,443988
4,779739	4,230471
4,997977	4,159284
5,264516	3,943207
4,989344	3,860351
4,998961	4,496486
5,77685	4,176516
5,088174	4,34852
4,761037	4,479981
5,845948	4,480338
5,025776	4,61948
4,795116	3,866194
5,887886	4,366471
4,687206	4,118078
5,489961	4,074687
4,952084	4,108398
5,035161	3,853565
5,421427	3,782009
5,066456	4,165002
5,306419	3,379651
4,918255	4,408891
5,699931	4,068914
5,424309	4,011636
5,054785	4,753638
5,027726	4,115652
5,270625	4,561705
5,395799	4,083587
4,834775	3,656279
4,88444	4,534899
4,803825	4,07213
5,166325	3,799535
4,919665	3,911865
5,60747	4,021848
4,907404	4,737111
5,074557	4,064806
5,152798	3,828629
5,046188	3,941306
4,658158	3,83485
5,840115	4,618475
5,157277	4,213038
4,819646	3,83377
5,883648	4,623745

4,976538	4,753656
4,910791	4,015309
5,123003	3,701037
4,766802	3,950044
5,346979	4,679533
5,046691	3,795036
5,42547	4,17401
4,989185	4,224468
4,771486	3,509387
5,699801	4,390045
5,162979	4,218001
4,745749	3,937178
5,491799	3,869191
4,807701	4,583158
5,031992	3,681461
5,932955	4,874322
4,761907	3,952975
4,911569	4,038611
4,933062	4,779411
5,153793	3,408892
4,616043	3,865238

Supple. Fig. 2 GSE62452

Controls	PDAC	Controls	PDAC	Controls	PDAC	Controls
TLR5	TLR6	TLR6	TLR7	TLR7	TLR8	TLR9
3,544049	3,339335	3,421935	4,282608	3,922255	4,613689	3,322731
3,258018	3,3707	3,267267	3,902597	3,695171	4,542565	3,050104
3,536573	3,433028	4,281996	4,439305	4,974648	4,565414	2,953656
3,729748	3,452336	3,62586	5,722237	3,665613	4,633952	2,93974
3,562511	3,324568	3,495375	5,587554	3,705662	4,881898	3,211464
3,334015	3,368764	3,366451	4,887165	4,217088	4,839554	3,433862
3,561822	3,705567	3,16266	4,554607	3,737721	4,698808	3,338206
3,332735	3,540535	4,815923	4,140026	3,826104	5,370974	3,552497
3,502741	3,457661	4,96533	3,573814	4,283746	3,756344	3,66457
3,962286	3,501051	3,452811	4,279023	3,79989	4,687117	3,308381
3,317376	3,353525	3,559486	4,546946	3,893433	4,41317	3,207344
3,842247	3,731837	3,419554	3,952496	3,673963	4,455126	3,176602
3,598511	3,863529	4,543314	4,807369	4,707836	6,811019	3,128655
	3,832007		5,474461		4,90448	
	3,431703		5,194198		4,702303	
	3,692714		4,690152		3,780107	
	3,438908		5,453959		4,882072	
	3,942031		5,842386		5,225752	
	3,523102		4,972173		4,426633	
	3,352983		4,602001		4,800609	
	3,35252		4,437862		4,054965	
	3,403077		3,735523		3,806773	
	3,651792		5,096376		4,627531	
	3,97629		5,845056		4,748441	
	3,894871		5,111019		4,904213	
	3,486515		4,116111		4,675866	
	3,47842		4,747161		5,247969	
	3,705587		3,797148		4,194367	
	3,610173		4,852934		5,894429	
	3,593288		5,888171		6,537596	
	3,531023		4,0314		4,329787	
	3,479551		5,921241		5,593507	
	3,405414		4,282738		4,205363	
	3,321363		4,713699		4,443206	
	3,379749		3,995778		4,067639	
	3,467382		4,961721		4,972036	
	3,263134		3,850836		4,018395	
	3,785854		6,358941		5,235862	
	3,748035		4,752055		4,774604	
	3,35992		4,012149		4,129788	
	3,486901		4,997422		4,636682	
	3,302363		4,408463		4,655658	
	3,623354		5,849299		6,302352	
	3,290666		4,555871		4,829304	
	3,387727		5,590236		5,46427	
	3,365896		3,902085		4,406421	
	3,310939		4,24145		3,775527	

3,838761	4,5195	6,169341
3,596876	5,503536	4,881288
3,778567	5,017023	5,539507
3,476257	5,047843	5,959077
3,475941	4,053647	4,481245
3,483468	6,272558	5,698308
3,637925	6,068595	5,204754
3,352973	4,227802	4,41213
3,437916	4,85447	5,895063
3,525097	5,669007	5,514709
3,260599	4,677537	4,559189
3,44536	3,767459	3,971894
3,368286	5,524738	4,881306
3,673542	4,668658	4,877155
3,444288	5,358512	4,970983
3,82087	5,806019	5,600783
3,379135	3,578756	4,293313
3,467774	4,568395	4,381478
4,005998	4,108072	4,801731
3,570716	5,330238	4,308555
3,557465	4,608196	4,513105
3,522328	3,932422	4,29721
3,448282	5,305198	5,413172
3,56442	4,753426	5,027832
3,429809	5,291101	5,275544
3,679971	3,519671	4,378419
4,429236	5,333593	4,791658
3,340318	4,013815	3,694774
4,138061	5,52056	4,914518
3,303072	4,008472	3,886841
3,543623	4,91967	4,301277
3,401967	4,211062	4,289309
3,449355	5,121235	4,789866
3,423013	3,094498	3,684048
3,279597	3,954714	4,014106
3,452549	5,055763	5,195062
3,489168	3,823007	3,542152
3,582676	4,328731	4,166538
3,538772	3,893377	3,877394
3,73515	5,341345	4,995303
3,552456	4,418639	3,964224
3,659813	5,790408	5,918131
3,691921	5,962606	5,036692
3,520558	6,075768	4,947612
3,42439	5,795953	4,736962
3,487273	4,868422	4,289691
3,257772	4,191522	4,149282
3,947297	4,968733	5,491268
3,40256	5,408924	4,496733
3,69274	4,782673	5,865373

3,61231	4,216453	4,794748
3,603392	4,31493	5,304137
3,427874	5,770753	5,685444
3,451414	4,686163	4,30529
3,432287	4,727171	4,806603
3,383917	4,582802	5,092293
3,484395	4,748162	4,336556
3,593176	6,191855	5,442379
3,321354	4,338555	4,446195
3,504429	4,66673	4,389699
3,874353	5,861335	6,410392
3,349741	5,231051	4,229549
3,563262	4,289121	4,254911
3,503462	5,288971	5,3059
3,465568	5,307246	3,775189
4,045213	6,495183	7,396903
3,603304	4,894824	5,051559
3,396934	5,844717	4,531116
3,522971	3,654446	4,037281
3,46521	4,414115	4,048944
3,370596	4,606415	4,740829

PDAC	Controls	PDAC	Controls	PDAC	Controls	PDAC
TLR9	TLR10	TLR10	CD14	CD14	LBP	LBP
3,105887	3,247083	3,141047	6,748646	8,577549	3,149464	2,861446
3,113603	2,898883	3,114105	6,847068	8,931213	2,978063	2,816217
3,143489	3,190588	3,179668	8,30383	9,165388	3,263192	2,740372
3,098307	2,979496	5,016386	7,147579	9,859573	3,014621	2,749505
3,160995	2,92262	4,26189	6,055993	9,045088	3,085965	9,788319
3,090299	2,86971	3,411352	7,73978	8,947513	2,800013	2,822813
3,005094	3,064674	3,04921	7,125162	9,266022	3,647192	3,760283
2,882422	3,200471	3,357695	7,820776	9,958633	2,69096	2,876122
3,059237	5,399433	3,043694	8,176546	8,152517	2,819323	2,71868
2,987209	3,009698	3,202575	5,066224	10,08427	2,96199	2,714997
2,870717	2,890814	3,528082	7,543831	8,816276	2,890866	2,849248
3,182589	2,762745	3,162365	5,407565	9,346849	4,048356	2,665439
3,098239	3,041021	3,799972	8,255067	9,940877	3,054469	3,088645
2,981962		3,987356		9,582141		3,472367
3,264899		3,411276		8,664301		2,949811
3,015046		2,891199		8,339876		2,967936
2,880951		3,537346		9,231548		3,047577
3,014236		3,619133		9,758793		2,993897
3,114126		3,252262		9,000167		2,707652
3,141867		3,319557		8,468526		2,816528
2,98503		3,248686		7,999549		2,718135
3,188339		2,840034		9,137361		2,720542
2,961727		4,603927		8,871347		2,87838
3,533247		5,567807		10,20246		6,458259
3,233511		4,137521		8,908271		3,591644
2,935026		3,199404		9,49431		2,715777
3,088202		3,278358		9,418769		2,967112
2,999405		2,849473		8,405378		2,814587
3,069928		2,980689		9,629976		2,722646
2,802388		4,18051		9,822826		2,643763
3,055522		2,973803		8,589713		2,649465
2,815275		4,160213		10,22092		2,565981
3,346331		3,81121		7,608882		2,977504
2,965125		2,97876		9,21823		3,127474
3,088966		3,481671		8,136478		2,970338
3,053371		3,444936		9,252453		3,276402
2,950301		3,101308		7,929016		2,696259
3,1501		3,472491		9,271611		2,813654
3,103231		4,935627		8,65463		3,473419
2,963095		3,076179		8,300914		2,831469
2,891924		3,246446		9,266759		2,944545
2,93256		3,121393		8,161076		2,906945
3,020533		3,701031		9,090791		3,10554
2,874192		3,15966		9,866969		2,853971
3,150512		3,195599		9,521463		2,797248
3,334759		3,228338		8,99124		2,896106
3,025699		3,114118		8,440452		2,723121

2,935502	3,281309	9,361269	2,877217
3,218805	4,102694	9,381923	2,944737
2,933163	3,622429	9,136547	2,571059
3,192974	3,279611	9,481876	3,154875
3,038261	3,06207	9,21008	2,853241
3,04456	4,039399	9,862703	2,802399
2,956001	3,441881	8,907071	2,956921
2,956699	3,113921	8,03906	2,899603
3,000894	3,550592	9,273737	2,900874
2,915256	4,570694	9,253537	3,13357
3,06261	2,972524	9,303208	2,88738
3,156162	3,049503	8,410091	2,664157
3,144371	3,710717	8,656119	2,711998
3,032001	2,94186	8,450628	2,808882
3,035691	3,972034	9,132328	2,845933
3,108117	5,692883	9,023784	3,309819
3,090299	3,033049	8,759289	3,016954
2,840421	3,031632	9,169993	2,679449
3,274303	3,326082	8,866157	2,854839
3,04759	3,748922	8,618919	2,955898
3,164112	3,873775	9,084063	3,04136
2,888605	3,01908	7,729534	2,743405
2,874698	4,068945	9,542173	2,898309
3,146165	4,410241	8,913176	2,730541
2,850044	3,653448	8,664474	2,898809
3,078479	2,978483	8,73718	3,032957
3,082552	3,898063	9,063278	3,828693
3,106234	2,927459	7,518883	2,728079
3,370083	4,803005	9,044486	3,272646
3,065751	3,17507	8,460496	2,852786
3,306357	2,874135	8,58067	3,063894
2,983549	3,147593	8,435054	2,713782
3,278472	3,162187	9,407412	2,746932
3,094109	2,790395	7,655844	2,713399
2,652011	3,011761	9,108976	3,055431
3,180053	3,23737	8,811662	2,906988
3,158334	2,964999	7,722278	2,547247
2,910901	2,901192	9,199266	2,791276
3,149353	3,000055	8,209442	3,130683
3,240965	5,469797	9,49997	3,213119
3,239504	2,852197	9,220006	2,702882
3,015799	4,273918	9,497456	4,207156
3,035698	3,708062	9,050897	2,852784
3,15558	2,954349	9,704008	2,62707
3,147372	3,486599	8,656618	2,799813
3,494006	3,010218	8,074374	2,895057
2,874161	2,974912	8,168561	2,958132
2,88112	3,473077	9,186536	2,563883
3,253281	3,154382	8,632149	2,919265
2,935123	4,549224	9,075313	2,674365

3,075373	2,795187	8,934452	2,765935
3,02686	4,38268	9,153941	3,488547
2,912185	3,59627	8,859633	3,139453
3,231709	3,206606	8,185445	2,673705
3,090299	2,911735	9,443234	2,660937
3,125618	3,485671	8,292511	3,174135
3,25348	3,538478	9,192549	2,607518
3,131781	3,210699	8,982043	2,786216
3,159957	2,917311	8,766281	2,534765
3,781781	3,582343	9,491037	3,157711
3,169887	3,578385	10,18051	2,745155
3,148158	2,784092	8,586604	2,725342
3,159282	2,94664	8,758032	2,643642
2,990206	3,108603	9,008811	2,891992
3,26959	2,800444	7,867568	2,865669
3,218956	3,652131	10,71953	2,752996
3,233914	3,266	8,665737	2,713328
3,189604	3,129071	9,243857	2,656647
3,042625	2,938927	8,617506	3,059957
3,110128	2,979954	8,194274	3,01922
2,966919	3,247272	8,172002	2,825897

Controls	PDAC	Controls	PDAC	Controls	PDAC	Controls
MyD88	MYD88	MD2	MD2	NOD1	NOD1	NOD2
4,405459	4,39507	6,950484	7,453391	4,459077	5,079203	3,504683
4,157295	4,572243	4,688875	8,507237	4,935435	5,478846	3,638208
4,549395	4,784275	7,902075	8,058581	4,609631	4,820906	3,209866
4,426599	4,456378	6,633943	8,193029	5,161307	5,031377	3,366665
4,420578	4,258589	5,437009	7,667635	4,880109	4,544192	3,268463
4,271569	4,396645	6,432661	7,698813	4,610942	5,172661	3,468076
4,28388	4,428467	5,228977	8,477758	5,012722	4,651714	3,206167
4,487001	4,720939	6,696238	8,719729	4,922092	4,941204	3,615981
4,453914	4,266022	8,086269	7,833147	4,857062	4,56167	3,78213
4,194078	4,238047	4,585989	8,314512	4,978715	5,808675	3,208364
4,708703	4,69203	4,845104	7,693801	4,552783	4,765106	3,566337
4,546564	5,103255	4,014998	7,564592	4,574923	5,30073	3,120094
4,625056	4,990301	6,522374	8,609263	4,668651	5,426868	3,45846
	4,640945		8,429792		5,02421	
	4,742112		7,46547		4,579375	
	4,847315		7,215026		4,521072	
	4,507123		8,453606		5,495281	
	4,536215		8,855533		5,052799	
	4,597015		7,846719		4,421733	
	4,305038		8,481308		5,132054	
	5,136014		7,508299		4,705839	
	4,293688		8,305793		5,517447	
	4,555729		8,430115		4,746594	
	4,68915		8,878924		5,834514	
	4,619717		7,775245		4,592655	
	4,801059		8,23401		5,556735	
	4,809128		8,46933		4,826774	
	4,549346		7,498522		4,929914	
	4,285927		8,728716		4,832191	
	4,826282		8,934952		5,800623	
	4,434704		8,242683		5,062885	
	4,311591		8,929468		5,087275	
	4,452774		7,801883		4,666037	
	4,432105		8,074047		5,250845	
	4,505389		7,746137		4,847081	
	4,611728		8,353024		4,739784	
	4,492729		6,832103		5,384593	
	4,409645		8,624839		4,696313	
	5,130888		8,152828		4,807545	
	4,661339		7,473587		4,604632	
	4,402801		7,759522		5,005717	
	4,730431		7,900072		4,752835	
	4,61633		9,13057		5,177439	
	4,417281		8,94619		5,513461	
	4,429028		8,054539		4,730275	
	4,589411		8,231947		4,953388	
	5,027392		7,477688		5,059521	

4,616247	9,241431	5,069113
4,515731	8,399708	4,944985
4,554768	8,791814	4,889414
4,760593	8,290865	5,67983
4,21636	8,450845	5,296785
4,406675	9,843537	5,125709
4,323465	8,667578	4,84228
4,396216	8,136293	5,507232
4,897564	8,078382	4,760784
4,749235	8,600639	4,779739
4,703232	8,28269	4,997977
4,333879	7,46346	5,264516
4,637736	8,591622	4,989344
4,224831	7,917039	4,998961
4,546364	8,715772	5,77685
4,46219	8,996601	5,088174
4,829549	7,846871	4,761037
4,331257	8,15549	5,845948
4,653029	8,352208	5,025776
4,413347	8,618632	4,795116
4,643287	8,071474	5,887886
5,013293	7,276799	4,687206
4,545781	8,64549	5,489961
4,416196	9,034078	4,952084
4,39098	8,870074	5,035161
4,346294	7,53981	5,421427
4,946847	8,66813	5,066456
4,473598	7,211286	5,306419
5,037996	7,77734	4,918255
4,387012	7,597149	5,699931
4,575	8,935012	5,424309
4,702045	7,417663	5,054785
4,716949	8,216102	5,027726
4,375886	7,948515	5,270625
4,17584	7,31477	5,395799
4,381177	8,178836	4,834775
4,373354	7,603087	4,88444
4,889751	8,681719	4,803825
4,729462	7,314951	5,166325
4,439526	8,486228	4,919665
4,426714	8,373362	5,60747
4,517135	8,302857	4,907404
4,486089	8,87782	5,074557
4,143424	7,946736	5,152798
4,483685	9,252682	5,046188
4,712999	7,45646	4,658158
4,161264	7,722348	5,840115
4,400122	8,787382	5,157277
4,450151	8,404381	4,819646
4,611295	8,329665	5,883648

4,542115	8,720199	4,976538
4,558868	8,207645	4,910791
4,317092	9,121882	5,123003
4,237296	7,938018	4,766802
4,366221	7,966163	5,346979
4,850733	8,262055	5,046691
4,833755	7,705468	5,42547
4,53103	8,970534	4,989185
4,821015	7,973434	4,771486
4,756851	7,862501	5,699801
4,603085	9,047286	5,162979
4,817454	8,103932	4,745749
4,534318	7,580803	5,491799
5,060011	8,401607	4,807701
4,184313	7,951928	5,031992
4,62812	9,411279	5,932955
4,427167	8,490048	4,761907
4,298276	8,773521	4,911569
4,624933	8,338835	4,933062
4,617471	7,083207	5,153793
5,040128	7,299709	4,616043

PDAC
NOD2
3,722819
3,769031
4,294378
4,206876
3,51581
4,261082
4,302952
4,195231
3,633557
4,083413
3,658425
4,63984
4,7782
3,707847
3,729595
3,649023
3,857238
4,199697
3,690313
4,21094
3,809606
4,479028
4,128848
3,908168
4,276375
4,297675
3,925059
3,881581
3,871938
5,078044
4,078935
4,456849
3,678946
4,225182
3,490632
4,34965
4,166027
4,066152
3,958899
3,69194
4,022953
4,138537
4,636755
4,56948
4,632013
4,605683
4,281224

4,440883
4,018641
3,990948
4,99505
4,941041
4,177898
3,929877
4,058404
4,443988
4,230471
4,159284
3,943207
3,860351
4,496486
4,176516
4,34852
4,479981
4,480338
4,61948
3,866194
4,366471
4,118078
4,074687
4,108398
3,853565
3,782009
4,165002
3,379651
4,408891
4,068914
4,011636
4,753638
4,115652
4,561705
4,083587
3,656279
4,534899
4,07213
3,799535
3,911865
4,021848
4,737111
4,064806
3,828629
3,941306
3,83485
4,618475
4,213038
3,83377
4,623745

4,753656
4,015309
3,701037
3,950044
4,679533
3,795036
4,17401
4,224468
3,509387
4,390045
4,218001
3,937178
3,869191
4,583158
3,681461
4,874322
3,952975
4,038611
4,779411
3,408892
3,865238

Supple. Fig. 2B

GSE62165			P-Wert	Fold changes	GSE15471		
	P-wert	Fold changes	-LOG10	LOG2		P-wert	Fold changes
**TLR1**	0,00068405	1,55896439	3,1649133	0,64058798	**TLR1**	6,35E-06	2,16845736
**TLR2**	9,33E-07	1,60200122	6,03018247	0,67987524	**TLR2**	8,55E-09	2,63265168
**TLR3**	0,66475498	1,06342703	0,1773384	0,08872104	**TLR3**	0,02092989	1,21778657
**TLR4**	8,36E-09	1,9610436	8,07801314	0,97162161	**TLR4**	0,00102701	1,73871812
**TLR5**	0,07529207	1,32048152	1,12325079	0,40106411	**TLR5**	1,33E-09	1,75454134
**TLR6**	0,90379514	0,9940866	0,04393	-0,00855656	**TLR6**	0,36038323	0,94685334
**TLR7**	8,55E-06	2,02131526	5,06814475	1,01529435	**TLR7**	1,24E-06	1,76457166
**TLR8**	0,00026956	1,70988248	3,56934032	0,77389718	**TLR8**	0,0015875	1,62108456
**TLR9**	0,00091666	0,88795924	3,03779309	-0,17143464	**TLR9**	8,78E-08	0,76466608
**TLR10**	0,17299301	1,20181934	0,76197146	0,26522004	**TLR10**	0,95189245	1,00993521
**CD14**	8,67E-16	3,61091991	15,0617405	1,85236642	**CD14**	3,73E-08	3,4586272
**LBP**	0,63149494	0,92283552	0,19963013	-0,11585455	**LBP**	0,05162701	0,85915369
**MD2**	5,81E-20	4,62460094	19,2356358	2,20932888	**MD2**	5,17E-08	5,26556863
**MYD88**	0,04719523	1,10293539	1,32610186	0,14134828	**MYD88**	1,07E-06	1,87419797
**NOD1**	0,00576598	1,22923169	2,23912713	0,29775687	**NOD1**	0,00032621	1,23344711
**NOD2**	3,69E-10	1,65025463	9,43261281	0,72268865	**NOD2**	2,52E-07	1,88905075

P-Wert -LOG10	Fold changes LOG2	GSE56560	P-wert	Fold changes	P-Wert -LOG10	Fold changes LOG2
5,19695092	1,11666908	**TLR1**	0,000523141	1,696243333	3,28138137	0,76234315
8,06781602	1,39651665	**TLR2**	7,65E-06	1,94434693	5,11651211	0,95928566
1,67923298	0,28426131	**TLR3**	0,24040417	1,15706047	0,61905801	0,21046427
2,98842471	0,79802406	**TLR4**	3,97E-08	2,20850056	7,40138131	1,1430672
8,87586032	0,81109394	**TLR5**	0,89740632	1,01141049	0,04701088	0,01636865
0,44323543	-0,07878712	**TLR6**	3,51E-06	2,09713639	5,45424087	1,06842069
5,90709968	0,81931802	**TLR7**	0,00216371	1,58494772	2,66480167	0,66443525
2,7992871	0,69695935	**TLR8**	0,0239913	1,32774539	1,61994627	0,40897852
7,05651977	-0,38709822	**TLR9**	0,00017733	0,73889405	3,7512251	-0,43656058
0,02141212	0,01426274	**TLR10**	0,05787206	1,3279363	1,23753105	0,40918595
7,42776304	1,79019952	**CD14**	0,00033221	1,77902423	3,47858785	0,83108616
1,28712301	-0,21901186	**LBP**	0,02335893	0,77740508	1,631547	-0,36326156
7,28617472	2,39658934	**MD2**	0,00276922	1,4573752	2,55764281	0,54337235
5,96968662	0,90627335	**MYD88**	8,79E-09	1,71653949	8,05624381	0,77950305
3,48649936	0,30269586	**NOD1**	0,00031029	1,23821985	3,5082381	0,30826749
6,59872419	0,91766146	**NOD2**	0,31210184	1,07878006	0,50570367	0,10940076

GSE62452	P-wert	Fold changes	**P-Wert -LOG10**	**Fold changes LOG2**
**TLR1**	0,38795156	1,05320888	0,4112225	0,07479159
**TLR2**	0,02079997	1,22372801	1,68193724	0,29128293
**TLR3**	0,65611	1,04118802	0,18302334	0,05823061
**TLR4**	0,10605742	1,14651899	0,97445893	0,19726025
**TLR5**	0,24311197	1,07508826	0,61419366	0,1044551
**TLR6**	0,0006977	1,30194002	3,15633182	0,38066299
**TLR7**	0,99222903	0,99904047	0,00338807	-0,00138498
**TLR8**	0,57590309	1,03386765	0,23965059	0,04805151
**TLR9**	0,01251693	0,91729969	1,90250235	-0,12453495
**TLR10**	0,27816687	0,92830727	0,55569459	-0,10732568
**CD14**	0,42302061	1,07457469	0,37363847	0,10376576
**LBP**	0,04468005	0,82421567	1,34988632	-0,27890621
**MD2**	0,1338156	1,07566218	0,87349327	0,10522505
**MYD88**	6,65E-06	1,23361496	5,17739989	0,30289216
**NOD1**	1,39E-06	1,32072178	5,85547669	0,40132658
**NOD2**	6,58E-05	1,19995623	4,18184569	0,26298178

Supple. Fig. 2C

TLR4 Donor	TLR4 PDAC	CD14 Donor	CD14 PDAC	LBP Donor	LBP PDAC	MyD88 Donor
8,024005	7,188143	9,825105	11,0067	4,516191	4,581478	10,0198
7,883575	7,272512	9,875826	11,01485	4,417954	4,356647	9,805203
4,069444	6,170016	6,880435	9,843904	5,474848	4,492528	7,634762
4,101506	6,21977	6,800088	9,627554	5,041196	4,361776	7,552259
4,110841	5,727781	6,423577	9,625518	5,069719	4,623454	7,625688
4,16133	5,783712	6,110048	9,459453	5,105162	4,509696	7,699189
4,03289	5,501859	6,702188	8,917298	5,145698	4,463114	7,61519
7,206167	6,538427	9,765453	9,716422	4,708967	4,956959	9,496619
3,89836	6,458978	6,479028	10,6673	4,941958	5,676878	7,688093
7,717112	7,077332	11,09707	10,64886	6,003307	6,052842	10,13736
5,360116	6,885421	9,433372	11,01077	4,677853	5,245062	9,315229
5,395642	5,642388	8,432879	9,519026	4,574075	4,464308	10,16098
4,888442	5,751816	8,518451	10,19064	4,660823	4,794851	9,732506
4,880601	6,477476	8,711594	10,78531	4,672224	4,846297	9,411208
4,938299	5,451113	7,947204	9,340656	4,908286	4,351136	8,018517
6,42027	6,025438	11,30795	9,990549	5,927217	4,588173	10,02588
4,255222	5,962361	7,72663	10,34506	4,709719	4,628076	8,106933
4,564127	5,390263	7,892483	9,674463	4,911019	4,626952	7,857157
4,239965	5,875704	6,762583	9,866359	4,887037	4,203327	8,046488
4,077811	5,578121	7,434511	9,962736	5,041878	4,81851	7,834042
5,650178	5,372119	9,799765	8,767459	4,642644	4,451751	9,179219
6,879251	6,403419	10,73187	10,98004	5,231699	5,125594	9,47705
4,360322	6,282762	7,407964	11,86707	4,879958	4,428527	8,081835
5,076685	5,202638	8,908279	9,109979	4,835029	4,509179	8,300917
4,284508	5,813063	7,151241	9,831961	4,877416	4,257121	8,046955
4,30121	5,357931	6,468007	8,619678	4,966448	4,530129	7,645793
4,323168	5,997682	7,906941	10,34576	4,560163	4,880505	7,938449
4,126497	5,332112	7,117928	10,05474	4,889635	4,471155	7,905507
7,178605	5,977444	10,30946	9,565249	5,546633	4,413695	9,590631
6,579513	5,423903	10,61285	9,452424	4,678882	4,414935	9,371019
4,721768	5,166293	7,318462	8,93111	4,72147	4,538382	9,735785
4,748635	5,323953	7,498231	9,170408	4,768369	4,394896	9,862158
4,574997	5,837627	7,333691	9,967041	4,853328	4,422323	7,89451
4,380107	4,795897	7,367631	8,841484	4,900875	4,667871	8,12414
3,952386	6,739317	6,632727	10,91028	5,267254	6,29194	7,922545
4,991143	5,432517	7,117825	9,198228	6,311351	4,238863	7,006027
3,94274	6,448457	6,786778	10,90126	4,945692	5,295787	7,672452
4,291408	5,940498	7,161746	10,36062	4,915629	5,961039	8,156204
7,159482	5,044998	9,990063	9,477488	4,583657	5,294045	9,622464

MyD88 PDAC	MD2 Donor	MD2 PDAC
9,745788	10,09111	9,970914
9,615481	10,04388	9,958494
9,581798	5,086916	8,966137
9,523269	4,751342	8,735068
9,832289	4,045627	8,966382
9,915296	4,033463	8,89578
9,772651	4,698379	8,931372
9,06314	10,02423	9,289288
9,395518	4,142053	9,157781
10,17505	9,807955	10,24887
9,938743	7,650853	9,744894
9,623098	7,878772	8,429812
9,454543	7,855949	9,087577
9,166413	8,049383	9,816341
9,083337	6,038103	8,502849
9,197116	9,977058	9,553132
9,585871	5,354613	9,279219
9,205763	6,002904	8,441935
9,438864	3,710644	9,353016
9,108949	5,194539	8,757507
9,039977	8,843633	8,621935
9,689016	9,932288	9,747196
9,866212	5,0207	9,097554
9,509435	8,016143	8,602227
9,517202	5,931973	9,455897
9,20227	4,262592	7,779605
9,841636	5,698415	9,30128
9,628859	5,385582	8,72854
10,01497	9,964248	8,873833
9,393786	9,641136	8,919301
8,981536	6,343057	8,27222
9,02744	7,362381	8,097809
9,593976	4,372068	8,692709
9,321504	5,265861	7,689415
9,550013	4,976483	9,950334
9,826193	4,574732	9,000027
9,592122	4,447504	9,623901
9,547285	4,805868	9,417989
9,095011	9,660571	8,451852

Supple. Fig. 3A

Months	low LAMP2/LC3	high LAMP2/LC3
36,5	1	
7,03	1	
19,61	0	
11,53	1	
33,08	1	
55,16	0	
1,05	1	
32,36	1	
17,91	0	
14,75	1	
18,3	1	
84,9	0	
14,39	1	
11,89	0	
16,89	1	
25,79	1	
17,61	1	
6,24	1	
9,69	0	
38,87	1	
38,64	0	
37,75	1	
25,95	1	
40,34	1	
6,6	1	
17,48	1	
16,62	1	
6,24	1	
20,14	1	
13,86	1	
6,64	1	
10,22	1	
36,24	0	
11,79	1	
7,79	1	
12,29	1	
10,78	1	
18	1	
28,42	1	
4,8	1	
5,26	1	
20,27	1	
0,82	0	
15,18	1	
69,19	1	
24,77	0	
11,56	1	

Supple. Fig. 3B

LMAP2_LC3B_LPS cells/mm2		
**Donor**	**Adjuvant**	**Neoadjuvant**
0	2,93	2,21
0,22	0,27	3,62
0,23	7,75	37,15
0,27	0,26	5,04
2,49	5,43	36,15
0,14	0,23	4,78
3	0,95	57,5
1,16	33,73	1,36
0,1	4,86	0,9
	8,15	2,04
	66,28	7,92
	0,75	2,19
	0	0,52
	13,06	7,78
	0,83	1,32
	5,18	5,44
	1124,29	1,07
	15,98	0
	6,84	24,63
	4,88	8,12
	13,18	9,01
	1,59	3,32
	1,16	2,89
	0,6	1,21
	0,52	7,88
	0,48	13,37
	0,11	16,57
	1,31	1,5
	3,23	6,91
	17,26	67,73
	3	16,66
	0,58	135.93
	524,17	20,72
	2,53	164,21
	4,53	11,73
	4,03	2,42
	9,7	11,97
	3,15	3,92
	2,58	4,54
	22,42	4,75
	1,93	9,81
	1,08	0,08
	4,2	12,8
	4,66	1,58
	2,64	3,39
	6	2,47

17,58	1	
5,95	1	
43,14	1	
13,86	1	
23,8	0	
77,17	1	
2,17	1	
11,53	0	
19,67		1
24,11		1
52,24		1
21,16		1
12,81		1
44,85		1
81,48		1
41,63		0
5,13		0
55,2		0
49,71		1
7,69		1
20,01		1
41,86		0
12,94		1
21,85		0
6,7		1
37,36		1
10,83		0
40,57		0
91,47		0
32,23		1
16,13		1
46,52		1
88,18		1
16,82		0
3,88		1
5,91		1
18,1		1
53,85		0
43,37		1
20,96		1
19,19		1
42,84		1
14,46		1
19,22		1
28,98		1
55,75		0
12,75		1
19,38		1
43,63		1
22,47		1

0,18	6,23
2,7	0,37
3,51	
4,44	
5,25	
3,62	
2,91	
6,44	
3,02	
1,01	
0,31	
73,45	
6,5	
0,61	
0,16	
4,21	
4,09	
1,38	

7,82	1
16,1	1
25,13	1
15,54	1
44,52	1
38,77	1
18,27	1
25,13	1
34,43	1
15,61	1
65,61	0
21,62	1
50,1	1
5,32	1

Supple. Fig. 3C

LMAP2_LC3B_LPS dots/mm2		
**Donor**	**Adjuvant**	**Neoadjuvant**
11,22955	187,76	300,87
100,2294	458,2	167,51
137,6076	159,16	941,09
72,98334	140,88	331,06
182,8335	208,35	1016,87
104,123	76,28	644,21
99,07637	161,97	1295,68
134,4632	1064,44	91,26
73,05538	504,32	42,29
	594,05	273,55
	1026,79	823,84
	47,84	523,71
	23,43	48,11
	602,69	904,59
	57,51	423,55
	216,36	416,04
	7522,85	291,21
	254,97	0
	142,57	1013,15
	461,01	957,58
	232,34	768,43
	77,59	443,42
	99,72	582,34
	45,04	195,39
	60,45	535,15
	53,91	1373,53
	150,84	1319,16
	74,5	240,27
	143,55	444,65
	639,03	3190,31
	124,69	421,26
	34,77	3200,36
	6275,05	1566,21
	385,29	2194,49
	665,84	503,9
	396,97	329,41
	395,14	1583,28
	242,43	234,42
	265,05	334,71
	657,91	399,45
	279,37	992,53
	153,75	429,52
	530,55	827
	467,3	478,66
	1652,88	321,19
	304,87	335,9

4,46	939,46
144,51	400,54
310,04	
1040,86	
183,51	
364,89	
207,82	
403,88	
490,15	
30,24	
31,29	
2325,08	
803,24	
177,86	
13,23	
398,89	
184,59	
108,07	

Supple. Fig. 3D

Type	Sample_ids	months	stat	16s positive cell/mm2	CA19-9 preop
Adjuvant	3866	19,67	1	1,7808	300,7
Adjuvant	3868	18,10	1	8,3060	6656,8
Adjuvant	3948	77,17	1	13,4043	2036,3
Adjuvant	3954	101,46	0		151
Adjuvant	4112	16,82	0	5,3411	1249,8
Adjuvant	4127	22,47	1	20,7623	57,3
Adjuvant	4132	25,79	1	2,9253	484,2
Adjuvant	4337	13,86	1	4,5750	4,2
Adjuvant	4432	5,32	1	413,8476	815,2
Adjuvant	4585	2,20	0	4,6396	408,6
Adjuvant	4828	16,10	1	34,7976	485,5
Adjuvant	4952	34,43	1	4,6903	2604,4
Adjuvant	4954	6,64	1	11,2366	508,6
Adjuvant	5008	84,90	0	12,9119	80,4
Adjuvant	5024	20,14	1	1,2204	5,1
Adjuvant	5031	14,75	1	2,1283	148
Adjuvant	5163	50,10	1	9,5610	24,8
Adjuvant	5311	43,37	1	10,9690	410,6
Adjuvant	5321	24,77	0	9,5399	691,5
Adjuvant	5353	49,71	1	22,9034	282
Adjuvant	5362	18,30	1	0,6088	1,1
Adjuvant	5398	33,08	1	39,7632	228,8
Adjuvant	5402	44,85	1	3,5272	667,3
Adjuvant	5410	35,63	1		288,8
Adjuvant	5457	88,18	1	29,5694	3305,9
Adjuvant	5517	9,69	0	4,2593	67
Adjuvant	5521	38,87	1	0,6307	100,1
Adjuvant	5530	15,18	1	28,5993	913,7
Adjuvant	5534	16,89	1	1,6515	1938,9
Adjuvant	5629	46,63	1		1044,4
Adjuvant	5737	12,29	1	1,3928	8,8
Adjuvant	5743	40,34	1	2,5450	100,2
Adjuvant	5795	38,64	0	28,4479	1907,8
Adjuvant	5919	32,36	1	83,6823	5,1
Adjuvant	5950	15,61	1	46,3394	21236,3
Adjuvant	5982	19,93	1		28,6
Adjuvant	6190	15,54	1	42,8787	14,3
Adjuvant	6202	5,13	0	0,1461	2447,3
Adjuvant	6209	52,03	0		286,5
Adjuvant	6210	41,43	1		204,8
Adjuvant	6221	46,52	1	1,1550	3902,3
Adjuvant	6251	11,79	1	0,5917	67,8
Adjuvant	6253	17,91	0	0,2764	407,9
Adjuvant	6260	23,80	0	0,6121	152,8
Adjuvant	6268	19,22	1	0,6934	8,4
Adjuvant	6296	55,20	0	1,2056	25,5
Adjuvant	6351	11,53	0	9,2630	760,4
Adjuvant	6359	16,13	1	13,1371	1123

Adjuvant	6391	53,85	0	76,1068	72,7
Adjuvant	6508	25,13	1	20,9680	31,7
Adjuvant	6574	7,69	1	5,5880	2406,3
Adjuvant	6726	19,61	0	46,2924	75,7
Adjuvant	6748	11,89	0	5,3822	166,4
Adjuvant	6771	5,95	1	4,4630	195,8
Adjuvant	6795	41,86	0	8,6276	3444,1
Adjuvant	6821	25,95	1	13,3398	100,2
Adjuvant	6987	17,48	1	28,4393	147,7
Adjuvant	7057	14,39	1	1,9213	20,3
Adjuvant	7059	52,24	1	5,7108	103,6
Adjuvant	7155	6,24	1	6,1228	2065,2
Adjuvant	7289	11,53	1	9,1971	142,2
Adjuvant	7372	55,16	0	120,1094	4,1
Adjuvant	7399	55,75	0	11,3301	122,5
Adjuvant	7451	37,36	1	10,8698	2477,6
Adjuvant	7515	16,62	1	8,5934	
Adjuvant	7559	7,03	1	6,6188	1355
Adjuvant	7573	40,57	0	6,6405	3517,2
Adjuvant	7603	43,14	1	28,7230	583
Adjuvant	7643	17,61	1	23,2172	407,3
Neoadjuvant	3530	3,88	1	4,9826	1
Neoadjuvant	3622	0,82	0	1,6398	
Neoadjuvant	3739	14,46	1	57,4673	10,2
Neoadjuvant	3855	24,11	1	2,1274	92,1
Neoadjuvant	3907	21,62	1	6,5972	36,3
Neoadjuvant	4055	2,17	1	4,8092	5309,8
Neoadjuvant	4129	43,63	1	18,1946	29,9
Neoadjuvant	4378	81,48	1	25,8374	88
Neoadjuvant	4593	1,05	1	4,0024	34,4
Neoadjuvant	4639	38,77	1	4,0024	279,1
Neoadjuvant	4686	20,96	1	105,0295	
Neoadjuvant	4715	44,52	1	28,7831	3,4
Neoadjuvant	4761	6,70	1	23,0381	8,3
Neoadjuvant	4880	10,83	0	25,7684	484,6
Neoadjuvant	4961	10,78	1	4,0753	356,2
Neoadjuvant	5153	42,84	1	196,6535	116,3
Neoadjuvant	5179	12,75	1	74,8471	398,4
Neoadjuvant	5276	13,86	1	3,9101	42
Neoadjuvant	5308	36,50	1	7,5954	42,6
Neoadjuvant	5471	28,98	1	23,2261	212,2
Neoadjuvant	5593	25,13	1	6,9017	5,8
Neoadjuvant	5603	91,47	0	100,3074	12,7
Neoadjuvant	5648	41,63	0	16,8666	
Neoadjuvant	5764	20,01	1	0,4275	1221,4
Neoadjuvant	5788	12,81	1	16,5092	270,7
Neoadjuvant	5794	5,26	1	0,3631	1
Neoadjuvant	5811	19,19	1	4,1442	3,3
Neoadjuvant	5850	7,79	1	0,6798	88,4
Neoadjuvant	5870	6,60	1	2,1366	12946,9

Neoadjuvant	5889	6,24	1	454,5105	3,9
Neoadjuvant	6035	21,16	1	37,6509	82
Neoadjuvant	6056	28,42	1	16,4692	503,6
Neoadjuvant	6126	7,82	1	10,8403	387,4
Neoadjuvant	6157	18,27	1	34,5872	55,6
Neoadjuvant	6196	19,38	1	1159,6131	74,4
Neoadjuvant	6240	21,85	0	62,0166	2,7
Neoadjuvant	6292	36,24	0	11,3528	117,1
Neoadjuvant	6300	12,94	1	24,0948	798,2
Neoadjuvant	6321	17,58	1	14,0897	86,6
Neoadjuvant	6346	11,56	1	3,0192	4,4
Neoadjuvant	6395	32,23	1	4,7115	453
Neoadjuvant	6399	18,00	1	42,4462	7,3
Neoadjuvant	6436	37,75	1	0,3731	23,9
Neoadjuvant	6448	5,91	1	0,5153	508,8
Neoadjuvant	6474	69,19	1	10,3181	310,9
Neoadjuvant	6483	4,80	1	3,3436	71,7
Neoadjuvant	6520	10,22	1	24,5099	9,4
Neoadjuvant	6933	65,61	0	46,3405	22,6
Neoadjuvant	7494	6,70	1		20
Neoadjuvant	7809	20,27	1	0,8112	219,6
Neoadjuvant	8005	5,73	0		1590

stage	therapy type
II	FFX
II	GEM
II	GEM
II	GEM
II	GEM
II	GEM
II	FFX
II	GEM
IV	GEM
II	GEM
II	GEM
II	GEM
II	GEM
II	GEM
II	GEM
IV	FFX
II	GEM
II	GEM
II	GEM
I	GEM
III	GEM
III	GEM
II	GEM
III	GEM
III	GEM
II	GEM
II	GEM
II	GEM
II	GEM
II	GEM
II	GEM
II	GEM
IV	FFX
II	GEM
II	GEM
II	GEM
II	GEM
III	FFX
IV	FFX
II	GEM
IV	GEM
II	GEM
II	GEM
II	GEM
II	
II	GEM
II	GEM
II	GEM

II	GEM
IV	FFX
II	GEM
II	GEM
II	GEM
II	FFX
II	GEM
II	GEM
III	GEM
I	FFX
IV	FFX
II	GEM
III	GEM
III	FFX
III	FFX
II	FFX
II	GEM
III	
II	FFX
I	FFX
I	GEM
I	GEM
II	GEM
I	GEM
III	GEM
II	GEM
II	GEM
IV	GEM
II	
II	GEM
II	FFX
III	GEM
III	GEM
II	FFX
I	GEM
II	GEM
II	GEM
I	FFX
IV	FFX
IV	GEM
I	FFX
II	FFX
III	FFX
II	GEM
II	FFX
II	FFX
IV	FFX

II	FFX
II	FFX
II	FFX
II	GEM
II	FFX
II	FFX
II	FFX
II	FFX
II	FFX
III	FFX
II	FFX
II	FFX
IV	GEM
II	FFX
III	FFX
I	FFX
I	FFX
IV	GEM
I	GEM
III	GEM
III	GEM
II	GEM

Supple. Fig. 3E

Type	Sample_ids	months	stat	Lamp2_LC3 cells/mm2	CA19-9 preop
Adjuvant	3866	19,67	1	423,82	300,7
Adjuvant	3868	18,10	1	954,40	6656,8
Adjuvant	3948	77,17	1	394,65	2036,3
Adjuvant	3954	101,46	0		151
Adjuvant	4112	16,82	0	902,23	1249,8
Adjuvant	4127	22,47	1	1479,62	57,3
Adjuvant	4132	25,79	1	110,88	484,2
Adjuvant	4337	13,86	1	197,69	4,2
Adjuvant	4432	5,32	1	6735,82	815,2
Adjuvant	4585	2,20	0		408,6
Adjuvant	4828	16,10	1	1594,32	485,5
Adjuvant	4952	34,43	1	2308,37	2604,4
Adjuvant	4954	6,64	1	200,88	508,6
Adjuvant	5008	84,90	0	88,00	80,4
Adjuvant	5024	20,14	1	195,37	5,1
Adjuvant	5031	14,75	1	79,10	148
Adjuvant	5163	50,10	1	5837,98	24,8
Adjuvant	5311	43,37	1	1002,70	410,6
Adjuvant	5321	24,77	0	314,76	691,5
Adjuvant	5353	49,71	1	662,93	282
Adjuvant	5362	18,30	1	81,44	1,1
Adjuvant	5398	33,08	1	25,67	228,8
Adjuvant	5402	44,85	1	497,09	667,3
Adjuvant	5410	35,63	1		288,8
Adjuvant	5457	88,18	1	884,17	3305,9
Adjuvant	5517	9,69	0	136,61	67
Adjuvant	5521	38,87	1	148,20	100,1
Adjuvant	5530	15,18	1	293,93	913,7
Adjuvant	5534	16,89	1	106,56	1938,9
Adjuvant	5629	46,63	1		1044,4
Adjuvant	5737	12,29	1	252,24	8,8
Adjuvant	5743	40,34	1	160,31	100,2
Adjuvant	5795	38,64	0	148,92	1907,8
Adjuvant	5919	32,36	1	55,87	5,1
Adjuvant	5950	15,61	1	2818,83	21236,3
Adjuvant	5982	19,93	1		28,6
Adjuvant	6190	15,54	1	1834,32	14,3
Adjuvant	6202	5,13	0	597,36	2447,3
Adjuvant	6209	52,03	0		286,5
Adjuvant	6210	41,43	1		204,8
Adjuvant	6221	46,52	1	870,05	3902,3
Adjuvant	6251	11,79	1	226,66	67,8
Adjuvant	6253	17,91	0	76,96	407,9
Adjuvant	6260	23,80	0	387,73	152,8
Adjuvant	6268	19,22	1	1140,87	8,4
Adjuvant	6296	55,20	0	645,40	25,5
Adjuvant	6351	11,53	0	423,03	760,4
Adjuvant	6359	16,13	1	824,75	1123

Adjuvant	6391	53,85	0	961,44	72,7
Adjuvant	6508	25,13	1	2282,62	31,7
Adjuvant	6574	7,69	1	670,90	2406,3
Adjuvant	6726	19,61	0	8,45	75,7
Adjuvant	6748	11,89	0	94,98	166,4
Adjuvant	6771	5,95	1	354,88	195,8
Adjuvant	6795	41,86	0	691,25	3444,1
Adjuvant	6821	25,95	1	159,42	100,2
Adjuvant	6987	17,48	1	175,59	147,7
Adjuvant	7057	14,39	1	94,97	20,3
Adjuvant	7059	52,24	1	441,13	103,6
Adjuvant	7155	6,24	1	127,30	2065,2
Adjuvant	7289	11,53	1	14,51	142,2
Adjuvant	7372	55,16	0	25,78	4,1
Adjuvant	7399	55,75	0	1217,28	122,5
Adjuvant	7451	37,36	1	753,49	2477,6
Adjuvant	7515	16,62	1	176,57	
Adjuvant	7559	7,03	1	7,15	1355
Adjuvant	7573	40,57	0	789,42	3517,2
Adjuvant	7603	43,14	1	366,41	583
Adjuvant	7643	17,61	1	119,26	407,3
Neoadjuvant	3530	3,88	1	907,84	1
Neoadjuvant	3622	0,82	0	291,17	
Neoadjuvant	3739	14,46	1	1127,84	10,2
Neoadjuvant	3855	24,11	1	433,58	92,1
Neoadjuvant	3907	21,62	1	3613,35	36,3
Neoadjuvant	4055	2,17	1	406,68	5309,8
Neoadjuvant	4129	43,63	1	1460,13	29,9
Neoadjuvant	4378	81,48	1	497,52	88
Neoadjuvant	4593	1,05	1	34,31	34,4
Neoadjuvant	4639	38,77	1	2061,68	279,1
Neoadjuvant	4686	20,96	1	1025,75	
Neoadjuvant	4715	44,52	1	1887,64	3,4
Neoadjuvant	4761	6,70	1	740,07	8,3
Neoadjuvant	4880	10,83	0	777,82	484,6
Neoadjuvant	4961	10,78	1	264,10	356,2
Neoadjuvant	5153	42,84	1	1083,03	116,3
Neoadjuvant	5179	12,75	1	1281,58	398,4
Neoadjuvant	5276	13,86	1	376,91	42
Neoadjuvant	5308	36,50	1	0,00	42,6
Neoadjuvant	5471	28,98	1	1159,57	212,2
Neoadjuvant	5593	25,13	1	1703,29	5,8
Neoadjuvant	5603	91,47	0	798,35	12,7
Neoadjuvant	5648	41,63	0	521,88	
Neoadjuvant	5764	20,01	1	676,63	1221,4
Neoadjuvant	5788	12,81	1	496,69	270,7
Neoadjuvant	5794	5,26	1	282,86	1
Neoadjuvant	5811	19,19	1	1025,78	3,3
Neoadjuvant	5850	7,79	1	245,80	88,4
Neoadjuvant	5870	6,60	1	168,26	12946,9

Neoadjuvant	5889	6,24	1	184,56	3,9
Neoadjuvant	6035	21,16	1	450,60	82
Neoadjuvant	6056	28,42	1	273,56	503,6
Neoadjuvant	6126	7,82	1	1487,21	387,4
Neoadjuvant	6157	18,27	1	2167,28	55,6
Neoadjuvant	6196	19,38	1	1359,08	74,4
Neoadjuvant	6240	21,85	0	710,78	2,7
Neoadjuvant	6292	36,24	0	214,98	117,1
Neoadjuvant	6300	12,94	1	694,09	798,2
Neoadjuvant	6321	17,58	1	327,83	86,6
Neoadjuvant	6346	11,56	1	323,25	4,4
Neoadjuvant	6395	32,23	1	818,46	453
Neoadjuvant	6399	18,00	1	266,84	7,3
Neoadjuvant	6436	37,75	1	159,37	23,9
Neoadjuvant	6448	5,91	1	934,65	508,8
Neoadjuvant	6474	69,19	1	303,13	310,9
Neoadjuvant	6483	4,80	1	282,70	71,7
Neoadjuvant	6520	10,22	1	202,77	9,4
Neoadjuvant	6933	65,61	0	3370,81	22,6
Neoadjuvant	7494	6,70	1		20
Neoadjuvant	7809	20,27	1	284,55	219,6
Neoadjuvant	8005	5,73	0		1590

stage	therapy type
II	FFX
II	GEM
II	GEM
II	GEM
II	GEM
II	GEM
II	FFX
II	GEM
IV	GEM
II	GEM
II	GEM
II	GEM
II	GEM
II	GEM
II	GEM
IV	FFX
II	GEM
II	GEM
II	GEM
I	GEM
III	GEM
III	GEM
II	GEM
III	GEM
III	GEM
II	GEM
II	GEM
II	GEM
II	GEM
II	GEM
II	GEM
II	GEM
IV	FFX
II	GEM
II	GEM
II	GEM
II	GEM
III	FFX
IV	FFX
II	GEM
IV	GEM
II	GEM
II	GEM
II	GEM
II	
II	GEM
II	GEM
II	GEM

II	GEM
IV	FFX
II	GEM
II	GEM
II	GEM
II	FFX
II	GEM
II	GEM
III	GEM
I	FFX
IV	FFX
II	GEM
III	GEM
III	FFX
III	FFX
II	FFX
II	GEM
III	
II	FFX
I	FFX
I	GEM
I	GEM
II	GEM
I	GEM
III	GEM
II	GEM
II	GEM
IV	GEM
II	
II	GEM
II	FFX
III	GEM
III	GEM
II	FFX
I	GEM
II	GEM
II	GEM
I	FFX
IV	FFX
IV	GEM
I	FFX
II	FFX
III	FFX
II	GEM
II	FFX
II	FFX
IV	FFX

II	FFX
II	FFX
II	FFX
II	GEM
II	FFX
II	FFX
II	FFX
II	FFX
II	FFX
III	FFX
II	FFX
II	FFX
IV	GEM
II	FFX
III	FFX
I	FFX
I	FFX
IV	GEM
I	GEM
III	GEM
III	GEM
II	GEM

Supple. Fig. 4A, 4B, 4C

HD ID	Group	Richness	Shannon	inverse_Simpson
4914	CP	26	3,1290194	0,77323454
4917	CP	41	4,46765272	0,920593208
5217	CP	27	3,96370062	0,905432985
5502	CP	49	4,69408293	0,931260783
5646	CP	49	4,64671987	0,918764986
5703	CP	29	3,8828009	0,88647339
5820	CP	39	4,52831543	0,936832861
5960	CP	43	4,64627171	0,932495453
6086	CP	31	4,18260852	0,91561778
6278	CP	11	2,2915482	0,697829801
6373	CP	50	4,74500432	0,929521554
6389	CP	48	4,66352081	0,932169607
6493	CP	23	3,6238419	0,878448143
6800	CP	36	4,1817919	0,90649998
7129	CP	47	4,68106098	0,927070998
7671	CP	48	4,48682086	0,913883867
7733	CP	21	3,52211895	0,87261361
8044	CP	50	4,65749612	0,920908856
8068	CP	23	3,37345998	0,809732621
8069	CP	42	4,50977687	0,923874701
8073	CP	39	4,43244426	0,924194368
8108	CP	33	4,20213442	0,915084015
8221	CP	17	3,40330696	0,861752383
8279	CP	31	4,10721054	0,906811097
8295	CP	33	4,49150132	0,942823914
8390	CP	29	3,92544386	0,897104006
8393	CP	43	4,33028014	0,910944273
8405	CP	40	4,25553864	0,908618957
8409	CP	10	2,31003226	0,711830225
8469	CP	46	4,57648998	0,929906616
8513	CP	39	4,34392216	0,912214228
8560	CP	12	3,1313209	0,854486401
4973	IPMN	28	3,91979513	0,902012931
5045	IPMN	30	4,04702528	0,90789401
5948	IPMN	46	4,55980093	0,928094068
6106	IPMN	27	3,72223438	0,86891889
6163	IPMN	10	2,42300432	0,738084173
6392	IPMN	40	4,46437094	0,925235001
6457	IPMN	32	4,12406122	0,902760749
6715	IPMN	43	4,34523733	0,910022595
6956	IPMN	27	3,81733515	0,881026857
7089	IPMN	13	3,12208614	0,861957701
7269	IPMN	37	4,25256725	0,910845339
7319	IPMN	34	4,1360434	0,903602246
7711	IPMN	38	4,31577215	0,916877627
8023	IPMN	22	3,88419891	0,900652851
8066	IPMN	19	3,51290201	0,870197791
8092	IPMN	8	2,56037333	0,792040955

8198	IPMN	20	3,7058566	0,886816398
8207	IPMN	11	2,69404288	0,79535179
8210	IPMN	19	3,399053	0,845533553
8265	IPMN	10	2,25999058	0,724201653
8267	IPMN	2	0,40430567	0,148214858
8276	IPMN	22	3,62504004	0,860990727
8439	IPMN	14	2,70330591	0,725483565
8494	IPMN	7	2,26570081	0,741610821
8503	IPMN	9	2,70750729	0,806950149
7927	Adjuvant	26	3,53106935	0,864465999
7963	Adjuvant	46	4,42994045	0,908355533
7977	Adjuvant	45	4,56828837	0,919765452
8001	Adjuvant	31	4,07841519	0,906792242
8030	Adjuvant	44	4,4586529	0,922846037
8035	Adjuvant	27	3,98149849	0,906644344
8048	Adjuvant	33	4,25984214	0,915007979
8099	Adjuvant	47	4,60753947	0,93109154
8118	Adjuvant	40	4,35382875	0,914032358
8145	Adjuvant	49	4,71045655	0,933039856
8197	Adjuvant	48	4,57906884	0,921092623
8209	Adjuvant	20	3,69298277	0,887812131
8214	Adjuvant	45	4,53138531	0,930764547
8215	Adjuvant	50	4,46565837	0,913369696
8216	Adjuvant	51	4,52366027	0,904252349
8219	Adjuvant	6	2,10105594	0,700737598
8225	Adjuvant	28	4,25445993	0,918762659
8231	Adjuvant	48	4,61729153	0,925188735
8247	Adjuvant	53	4,71144085	0,93355972
8257	Adjuvant	55	4,73181416	0,921844301
8268	Adjuvant	48	4,64342018	0,926813735
8272	Adjuvant	53	4,51275978	0,911377908
8293	Adjuvant	49	4,58813464	0,920860072
8366	Adjuvant	30	3,92946913	0,886389031
8376	Adjuvant	54	4,82666079	0,93336347
8401	Adjuvant	57	4,69347168	0,920660261
8417	Adjuvant	49	4,62495034	0,925141866
8453	Adjuvant	52	4,64888812	0,924622326
8455	Adjuvant	50	4,72631116	0,930776076
8465	Adjuvant	48	4,51888522	0,923794351
8571	Adjuvant	44	4,52576501	0,930715444
8640	Adjuvant	31	4,07067843	0,91037187
7968	Neoadjuvant	24	3,91419876	0,899533657
8029	Neoadjuvant	50	4,58005559	0,921659994
8046	Neoadjuvant	44	4,30799723	0,910587515
8059	Neoadjuvant	54	4,70497223	0,926429277
8061	Neoadjuvant	47	4,4338652	0,923776873
8085	Neoadjuvant	51	4,71234152	0,928726374
8217	Neoadjuvant	57	4,82731282	0,934788063
8222	Neoadjuvant	48	4,71684674	0,938021564
8260	Neoadjuvant	32	3,89329006	0,889303043

8273	Neoadjuvant	47	4,55684029	0,918264813
8359	Neoadjuvant	42	4,51124811	0,922977534
8381	Neoadjuvant	57	4,88228853	0,935454987
8575	Neoadjuvant	41	4,42335202	0,921178696
8644	Neoadjuvant	36	4,25530674	0,912339785

	Richness	Shannon	inverse_Simpson
**P_MW CP vs I**	0,0007	0,0003	< 0,0001
**P_MW CP vs I**	0,0003	0,0191	0,2074
**P_MW IPMN**	< 0,0001	< 0,0001	< 0,0001

Supple. Fig. 4D

HD ID	Group	Akkermansia	Anaerobiospi	Anaerostipes	Bacteroides	Bariatricus
4914	CP	1,84165817	0	0	1,70574244	0
4917	CP	2,18602328	0	0	3,33474202	0
5217	CP	1,37483292	0	0	1,4957673	0
5502	CP	1,31245533	0,40283282	0,15593529	2,69313235	0,27938406
5646	CP	1,16540096	0,27463342	0,47523523	2,34990686	0,08119597
5703	CP	1,72384844	0	0	1,28169251	0
5820	CP	2,16983401	0	0	1,50589367	0,07216743
5960	CP	1,79048043	0	0	3,09905101	0
6086	CP	2,32616308	0,48149075	0,27513757	1,71335668	0
6278	CP	0	0	0	5,39708266	0
6373	CP	1,3916059	0,09336653	0	2,4831051	0
6389	CP	1,85128983	0,12139605	0,13353566	3,15326252	0,16084977
6493	CP	2,54535025	0	0	1,26909013	0
6800	CP	2,22680412	0,26804124	0	3,02061856	0
7129	CP	1,64604477	0,12234117	0,45043793	2,03809259	0
7671	CP	1,51581893	0,04923173	0,18656233	1,7464307	0
7733	CP	2,24106204	0	0	1,39984227	0
8044	CP	1,35423384	0,30507466	0	2,85480431	0
8068	CP	0	0,43681747	0	4,06656266	0
8069	CP	1,24536301	0	0	1,86091068	0
8073	CP	1,4498124	0	0	2,76992913	0
8108	CP	2,72669258	0	0,11602947	2,09433196	0
8221	CP	1,5847348	0	0	1,30983182	0
8279	CP	1,46200196	0	0	1,77495446	0
8295	CP	0,78912186	0,31630091	0	1,31085532	0
8390	CP	2,94138099	0,38940268	0	2,9831027	0
8393	CP	1,42240918	0	0	2,37424691	0,05703896
8405	CP	2,27330519	0,60011189	0	2,89884555	0
8409	CP	0	0,45580335	0	4,65570568	0
8469	CP	1,59778598	0	0,29520295	2,82656827	0,03690037
8513	CP	1,53398276	0	0	1,84749748	0,17915127
8560	CP	1,74808038	0,53912759	0	0	0
4973	IPMN	3,63067836	0,052115	0	4,42977504	0
5045	IPMN	1,83038788	0,35574937	0	2,6394308	0,26394308
5948	IPMN	1,29385087	0,19563381	0	2,53434707	0
6106	IPMN	2,0926883	0	0	0,71168347	0
6163	IPMN	0	0	0	1,09907121	0
6392	IPMN	1,43345822	0	0	2,1762502	0
6457	IPMN	1,60004169	0	0	2,5225413	0
6715	IPMN	1,91195175	0,14770443	0,38977557	0,48003939	0,25437985
6956	IPMN	1,93852602	0	0	1,22249389	0
7089	IPMN	3,32351015	0	0	3,0124427	0
7269	IPMN	1,87568289	0	0,30957873	2,94403302	0,16996479
7319	IPMN	1,52905199	0	0,08293163	2,25470378	0
7711	IPMN	1,58420984	0	0,24239276	2,15123577	0
8023	IPMN	1,4235882	0	0	2,05524654	0
8066	IPMN	1,81567212	0	0	4,00297303	0

8092	IPMN	4,05405405	0	0	3,04716481	0
8198	IPMN	0,95327886	0	0	1,08946155	0
8207	IPMN	1,21991793	0	0	0,16635245	0
8210	IPMN	0,48821024	0	0	1,53734289	0
8265	IPMN	5,1627907	0	0	0	0
8267	IPMN	0	0	0	0	0
8276	IPMN	0,42254251	0	0	0,96951472	0,18664455
8439	IPMN	1,17179742	0	0	0,63555114	0
8494	IPMN	1,51320852	0	0	1,69274173	0
8503	IPMN	1,28141942	0	0	0,54213898	0
7927	Adjuvant	2,41028388	0	0	2,0085699	0
7963	Adjuvant	1,17620851	0	0,13588773	2,39251504	0,03341501
7977	Adjuvant	0,970453	0,09987186	0	1,89944976	0
8001	Adjuvant	1,13554061	0	0	2,96689084	0
8030	Adjuvant	2,0750137	0,36019106	0	3,21039856	0
8035	Adjuvant	1,47212362	0	0	2,33869284	0
8048	Adjuvant	1,74271763	0,3746422	0,19784476	2,84138744	0
8099	Adjuvant	1,51557589	0	0	2,28425606	0
8118	Adjuvant	1,51016184	0	0	2,46989085	0,07056831
8145	Adjuvant	1,35890517	0	0	2,66640966	0,16383963
8197	Adjuvant	0,46898336	0,14174142	0	0,50798604	0,0684925
8209	Adjuvant	0,0401714	0	0	3,08650241	0
8214	Adjuvant	1,86801534	0	0,02447182	2,50020393	0
8215	Adjuvant	1,6888434	0	0,26375876	2,73206834	0,1850248
8216	Adjuvant	1,10179886	0	0,19215045	2,92109567	0,05519215
8219	Adjuvant	0,06357455	0	0	0,05942838	0
8225	Adjuvant	0,71307448	0,17392061	0	0,8326449	0
8231	Adjuvant	1,41124476	0	0,30384451	2,09426713	0
8247	Adjuvant	1,26928501	0,09188427	0,24787384	2,2372751	0,07051583
8257	Adjuvant	1,09111406	0,0946169	0	1,94870556	0,22144381
8268	Adjuvant	0,89296636	0	0,26736566	1,9781564	0
8272	Adjuvant	0,77036024	0	0,07039846	2,11195366	0,22929783
8293	Adjuvant	0,81405084	0	0,04275103	1,80088708	0,06590784
8366	Adjuvant	1,61733657	0	0,16731068	0,0637374	0
8376	Adjuvant	0,92049701	0,12911262	0	1,63137588	0,05316402
8401	Adjuvant	0,77491137	0,13592478	0,00560515	1,04255707	0,18076594
8417	Adjuvant	0,51942343	0,09139076	0,09370445	0,66865644	0
8453	Adjuvant	0,88394639	0	0,12915346	2,16541707	0
8455	Adjuvant	1,02337233	0,32513392	0,34645417	2,03075447	0
8465	Adjuvant	1,31311371	0,07553309	0,11039452	1,6210563	0,25565046
8571	Adjuvant	0,57385416	0	0,09584983	0,99459756	0
8640	Adjuvant	1,63030448	0,26372573	0	1,79333493	0
7968	Neoadjuvant	1,90967088	0	0,23237146	1,67729942	0
8029	Neoadjuvant	1,41951488	0	0,29768467	2,42557883	0,08820287
8046	Neoadjuvant	1,55605105	0,14232174	0,28464348	2,98875658	0
8059	Neoadjuvant	1,27821002	0,29720195	0,16672305	2,38486445	0
8061	Neoadjuvant	1,57783393	0	0	2,84127637	0
8085	Neoadjuvant	1,21832142	0,23745062	0,25520394	1,58670277	0
8217	Neoadjuvant	1,34030729	0,1004063	0,39462009	1,92406482	0
8222	Neoadjuvant	1,61988035	0,27611597	0,21782482	3,04034361	0

8260	Neoadjuvant	1,57900631	0	0,35462317	1,27291052	0
8273	Neoadjuvant	1,17337165	0,25034831	0,24599443	1,97884013	0
8359	Neoadjuvant	1,26965494	0,49426956	0	2,97797411	0
8381	Neoadjuvant	0,90505293	0	0	1,4594396	0
8575	Neoadjuvant	1,00647977	0,15587076	0	1,00425305	0,22044579
8644	Neoadjuvant	1,66297935	0,23230088	0	2,37463127	0

Blautia	Citrobacter	Clostridium	Corynebacter	Cutibacteriun	Duncaniella	Enterobacter
0	0,01359157	0,652396	0,53686714	1,94359497	0	0,3058104
0	0,09604672	0,603173	0,39187061	0	0,19977717	0,1959353
1,13932913	0	0	0,45827764	1,164789	0	0
0	0,00974596	1,104541	0,40608148	0,91936846	0,17217855	0,63998441
0	0,02149305	0,599417	0,16716817	0,49434016	0,21015427	0,5707599
0,87871495	0,01679073	1,108188	0	0	0,3190239	0,29663626
0	0,71205196	0	0,52441665	1,06326678	0	0,55809478
0	0,05189798	1,256673	0	1,33081257	0	0,16310795
0,93171586	0,04377189	1,738369	0,43146573	0	0	0,71910955
0	0,66450567	0	0	0	0	0,76175041
0,06002134	0	1,469412	0,36012805	0,81806865	0,175618	0,58465232
0	0	0,61912	0,55235205	0,73444613	0,90136571	0,16084977
1,39098014	0,00717	0	0,05019001	0	0	1,0109701
0	0,01030928	0,804124	1,08247423	2,1443299	0	0,69072165
0	0,01946337	0,728486	0,31419436	0,84526623	0,51994995	0,43097456
0,05700516	0,018138	0,342031	0	0,71774674	0,27466121	0,40421838
0	0	0,308885	0,50604627	0	0	0,82150368
0	0,02976338	0,935066	0,35716057	0,6523141	0,73168312	0,30011409
0	0	0,083203	1,32085283	0	0	0,29121165
0,00815295	0	0,03465	0,27108557	0,65019771	0	0
0	0	0,727222	0,44930289	1,10704525	0,2825513	0,33350317
0	0	0,690375	0,20885305	1,57800081	0,77739746	0
0,66300129	0	0,565977	0	1,45536869	0	0
0	0	0,85011	0,26157224	0	0	0,8547807
0,02608667	0	0,453256	0,27391007	0,63912349	0	0,29999674
0	0	1,008275	0,36158821	0,05562895	0,41026354	1,17516167
0	0,00712987	0,30302	0,12477274	0,96253253	0,1212078	0,35292859
1,0934242	0	0	0,21359915	0,3356558	0,28479886	0,82896811
0,3744099	0	0	0	0	0,87904932	0
0	0,04797048	0,380074	0,12546125	1,36900369	0,80442804	0,15498155
0,01492927	0	0,291121	0	0,97413504	0	0,26499459
0	0	0	0,91488319	1,47034798	0	0
0	0	0,138973	0	0,12160167	0,32137584	0,98149917
0	0	0,120496	0,25820519	1,4000459	0	0,23525361
0	0,03556978	0,578009	0,53799297	1,2982971	0,3690365	0,41794496
0	0,05083453	1,059053	0,51681776	1,08447005	0,14403118	0,16097602
0,20123839	0	1,362229	0,68111455	0	0	0
0	0,01954716	0,026063	0,43981104	0,62225118	0,19221372	0,47238964
0	0	1,334237	0,40652525	0	0	0,45864387
0	0	0,484142	0,11898412	1,15701801	0,05744061	0,67697862
0,76842473	0	0,61998	0	1,03038771	0	0,0523926
0	0	0	0,32743942	2,99607073	0	0,66306483
1,06227996	0,09105257	0,613087	0	1,16547287	0,12140342	0,2306665
0	0,00518323	1,218058	0	1,28544032	0	0,47167366
0	0	1,034498	0	1,27689045	0,68822231	0,17313769
0,25454888	0	0	0,94277364	0,98048459	0	0,55623645
0,80696539	0	0	2,09173922	0	0	0,89190911

0	0	0	0	0	0	1,11287758
0	0	0	1,01613241	1,53991201	0	0
0,07763114	0,12199179	0,443607	0,38815571	1,57480315	0	0
0,94525813	0	0	0	0,85177106	0	0,21813649
0	0	0	3,79844961	0	0	0,79069767
0	0	0	0	0	0	0
0,30848196	0	0,209975	0,13479884	0,39921195	0,13479884	0
0	0,21847071	0	0,19860973	0	0	0,45680238
0	0	0,74378	0	0	0	0
0	0	0	0	1,19927715	0	0
0	0	0,117836	0	0,06963042	0	0,21960364
0	0,04678102	0,76409	0	0,96012475	0	0,07796837
0	0	0,776362	0,10740936	1,10235924	0,22800935	0,26381247
0	0	0,589757	0	0,02845318	0	0,19399897
0	0,0195756	0,070472	0	1,2215175	0,21141649	0,61075875
0	0,02088119	1,174567	0	0,93443308	0,08352474	0,3027772
0	0,06735141	1,41438	0	0	0,17258798	0
0	0,01867239	0,936732	0	1,12034357	0,1618274	0,31743068
1,24670681	0	0,870342	0,16936394	0	0,26815958	0,45163718
0	0,00963763	0,648933	0	0,69712156	0	0,44333076
0	0,01522056	0,019026	0,14174142	0,43759097	0,16837739	0,19025694
1,56668452	0	1,111409	0	0	0	0
0	0,02039318	0,342605	0	1,1746472	0,40378497	0,43641406
0,08660735	0,04330368	0,88182	0	0,755846	0,60625148	0,20470829
0	0,00613246	0,570319	0,24938675	0,92804579	0	0,59893704
0	0	0	0	0	0	0
0	0	0,341319	0,55002391	0,65872429	0,28044698	0
0,00502222	0,01506667	0,667956	0,2787334	0,98686689	0,46455566	0,73324461
0	0,0149579	0,386769	0,0982948	0,71370571	0,02136843	0,63036882
0	0,01811813	0,549584	0,23754882	0,79317148	0,41269074	0,4247695
0	0,02096986	0,712975	0,01572739	0,72870249	0,40192224	0,32328528
0	0,0643643	0,56721	0,13476276	0,78242855	0,16694491	0,13878553
0	0	0,388322	0,18525446	0,63770284	0	0,10509628
0,10357328	0	1,083536	0	0,11950763	0,386408	0,1593435
0,01822766	0	0,381263	0	0,6015129	0	0,21417505
0,00560515	0,00420386	0,539496	0	0,57172484	0,10649776	0,16254922
0	0,00231369	0,490502	0	0,26723121	0,02892113	0,05668541
0	0	0,028514	0	0,33210889	0,35055939	0,27675741
0,01066013	0	0,812835	0,34645417	0,11193135	0,4477254	0,24251792
0	0,01452559	0	0,15106618	0,60716983	0	0,26727093
0,28754948	0,00248961	0,043568	0	0,40954017	0,07344337	0,07842259
0	0,01918005	1,318629	0,16303045	1,1268281	0	0,17262047
0,92103595	0	1,390004	0	0	0,26617094	0,36334446
0	0,01653804	0,441014	0	0,66703418	0,10198456	0,1323043
0	0,03795246	0,839698	0	0,28938754	0	0,16129797
0	0,01449766	0,654811	0,05799062	0,83119896	0,08940221	0,47117383
0	0,04407357	0,48481	0	0,70811541	0,00293824	0,10283834
0,00443833	0,00887666	0,630242	0,05991745	0,79002264	0,29071058	0,53481869
0	0,04203054	0,354925	0,30822398	0,73319946	0,18213235	0,33857937
0	0,02147569	0,696426	0,36201871	1,42046326	1,0615125	0,12885412

0	0	0,496472	0	0	0	0,30236291
0	0	0,254702	0	0,64219784	0,3853187	0,19810171
0	0,02162429	0,657996	0	0,99162831	0,16372679	0,25640234
0	0,01669839	0,769796	0,11521892	0,77981498	0,4074408	0,3773837
0	0	0,015587	0	0,7882607	0,06902848	0,25161994
0	0	0,955015	0,41666667	1,08775811	0,17330383	0,10693215

Enterocloster	Enterococcus	Escherichia	Extibacter	Faecalibacter	Flavonifractor	Flintibacter
0,06795787	2,47366633	13,9041794	0	0	0,53007136	0,36697248
0,16135848	1,81720389	6,7347958	0,24972146	0,16520035	1,05651389	0,59164778
0,63649672	2,12589905	5,12379861	0	0	0,42008784	0
0,03248652	1,67305568	4,96718862	0	0,15268664	0,58475733	0,0877136
0,09791279	1,0698763	5,44012991	0,10746525	0,08358409	0,78091417	0,19104934
0,34700845	2,06525998	5,64168579	0	0	0,4813343	0
0,08660091	3,47365889	5,75414963	0,22612461	0	1,43372624	1,38080346
0,66355279	2,12411032	5,52342823	0,15198695	0	0,27431791	0,35587189
0,4002001	2,31365683	6,39694847	0,16258129	0	1,85092546	0,6128064
0	0	10,5510535	0	0	0	0
0,07780544	1,70505068	5,23741775	0	0,40681131	0,62688956	0,44237951
0,03338392	2,07283763	5,5660091	0,15174507	0,31562974	1,00151745	0
0	2,05062021	8,0375708	0	0	2,31591023	0
0,07731959	2,46391753	7,60824742	0,61340206	0	0,48453608	0
0	1,77394689	5,80564438	0,13624357	0	0,57834005	0,3364382
0,05700516	1,52877465	5,17192237	0,16583318	0,30057264	0,52341098	0,09846345
0	3,10856993	6,01340694	0,446898	0	0	0
0,49109579	1,6865916	5,6972072	0	0,23314649	0,52085917	0,38444367
0,1976079	0,07280291	9,79719189	0	0	0,54082163	0
0,07133831	1,21886592	4,08462761	0	0,12840895	0,58701235	0,56866822
0	1,83889944	4,3957571	0	0,26402335	0,26402335	0,23159943
0	3,10958984	6,24238557	0	0	0,89342693	0,5047282
0	2,60349288	7,55174644	0	0	0	0
0,09808959	1,9524499	4,77369331	0,22420477	0,35499089	0,45308048	0,16815358
0,28043173	1,72498125	2,11954218	0	0	0,36521342	0
0,07648981	2,94833461	7,00924831	0	0	0	1,5437035
0,04634416	1,96784428	4,95169513	0	0,10694806	0,38501301	0,39214288
0	2,8530743	6,43848853	0,45771245	0,19325637	0,64079744	0,10679957
0	0	13,1857399	0	0	1,95344294	0
0,08856089	1,82287823	4,53874539	0,46125461	0,29889299	0,38376384	0,35793358
0	1,23166499	5,00877095	0,328444	0,26872691	0,56358004	0
0	3,08773076	6,51854272	0	0,58813919	1,89511518	0
0,052115	3,25718753	8,25154174	0,64275167	0	2,63180752	2,16277252
0	2,10006885	4,99196695	0,21803994	0	0	0,32132201
0,56467031	2,44097639	5,02867814	0,14227913	0,37348273	0,30678938	0
0	2,87215115	7,78615606	0	0,64390409	0,17792087	0
0	4,55108359	14,4891641	0	0	2,2755418	0
0,02606288	1,60612478	4,16354455	0	0	0,68740837	0,18895586
0,0416949	1,73555011	5,84249752	0,2345338	0	0,29707615	0,50555063
0,03282321	1,68629221	6,0107496	0	0,06564641	0,65236122	0,11077832
0,27069508	2,29654209	7,69297939	0	0	0,76842473	0,41914076
0	2,07924034	5,24721676	0	0	0	0
0,14568411	2,14277043	5,94269758	0,15782445	0,12747359	0,32171907	0,17603496
0,04146582	1,69491525	5,76374851	0,26952781	0	0,20214586	0,66345307
0	2,0213825	5,3239839	0	0	0,74449206	0,32896161
0	2,45121146	5,92061846	0	0	0,69765249	0
0	2,39966023	7,42195795	0	0	0	1,6351667

0	0	9,88341282	0	0	0	0
0	0,86947413	6,7043788	0	0,30379216	0	0,83804735
0	0	3,92591771	0	0	0,44360652	0
0	1,080295	6,24285863	0	0,50898515	0	0,15581178
0	0,27906977	11,4883721	2,10852713	0	0	0
0	0	17,0242537	0	0	0	1,49253731
0	0,64547905	2,57413936	0	0	0	0
0	1,6285998	8,14299901	0	0	0,67527309	0
0	1,15414209	6,18107207	0	0	0	0
0	1,0021357	3,63068835	0	0	0	0
0,0374933	2,55490091	6,64167113	0	0	0,87305838	0
0,02227668	1,06928046	5,388728	0	0	0,28068612	0,38984184
0,05841562	1,50749981	5,06143062	0,26758122	0,06783749	0,45601869	0,57661868
0,01551992	1,57268495	4,48784273	0,37506467	0	0,2250388	0,53026384
0,0587268	1,49557591	5,55947068	0	0	0	0,03132096
0,10962623	1,72791815	4,87053665	0	0	0	0
0,05051355	1,99107594	5,0345176	0	0,56827749	0,43778414	0,63141943
0,04356892	1,48134317	4,68677061	0	0,12759468	0,51971493	0,30187035
0,25404592	2,08882198	6,29469326	0	0,18818216	0,5833647	0
0,06746338	1,500257	4,93767669	0	0,4690311	0,29555384	0,44654331
0,21499034	0,55459899	2,07570324	0,15696198	0	0,18264666	0,18454923
0	2,50401714	5,18880557	0	0	0,6293519	0,54231387
0,09380863	2,10049759	5,37564239	0,11828045	0,33444816	0,79941268	0,87690676
0,09841745	1,6770333	6,90496811	0	0,22832848	0,23620187	0,44091016
0,053148	1,33483238	7,19746525	0,26369583	0,38021259	0,84832379	0,46606705
0	0	0,20316214	0	0	0	0
0	0,90873516	2,65011522	0	0	0	0,22174877
0,02008889	1,79544484	5,02975667	0,24106672	0,23855561	0,43693343	0,53235567
0,01923159	1,52570623	3,4766443	0,16026326	0,12821061	0,21795803	0,6047267
0,02013126	1,17566534	4,89793453	0,19124693	0,20131256	0,20936506	0,17111567
0,03494976	1,30362604	3,79903888	0	0,32328528	0,31804281	0,09261686
0	1,46831064	4,96610817	0	0,19510429	0,40831104	0,3097532
0,01603164	1,3715955	3,9687205	0	0	0,28144427	0,66264094
0,02788511	1,88423694	4,87192766	0	0,10357328	0,56566944	0
0,03949327	0,58784215	3,32502962	0	0,10480907	0,28404776	0,7853085
0,0210193	1,07899051	3,12486863	0,09388619	0,09809005	0,20318653	0,18356852
0,01619583	0,74038083	2,2905532	0,04858749	0,18278152	0,23599639	0,33664191
0,03522367	1,11541623	4,05910867	0	0,04696489	0,51158188	0,39752428
0,12259148	1,45777257	4,66913626	0,18388722	0,60762732	0,51168617	0,30647869
0,02324095	1,78374296	4,09331242	0	0	0,55197258	0,38057057
0	0,55642692	1,62695745	0,05477133	0,03734409	0,20788209	0,00622401
0	1,61591944	4,70390794	0,4363462	0,1438504	1,13162311	0
0	1,62237526	5,40791753	0	0	0,77738814	0,51544214
0,03307607	1,25413451	4,53142227	0	0,20948181	0,49062845	0,18467475
0	1,93557569	5,22795199	0,25617914	0	0	0,03320841
0,01691393	1,47876093	4,86879621	0,12806263	0,27545547	0	0,12806263
0,0381971	1,34571311	4,46906035	0	0,05582653	0,63465946	0,52006817
0,07101327	0,88766588	4,50712352	0	0,2640806	0,39279215	0
0,07939102	1,66954654	4,91056835	0,11208145	0,11208145	0,16111708	0,45533087
0,10124252	1,54011351	5,12041724	0	0,18714527	0,78846449	0

0,08958901	1,58273918	4,46451902	0,45167793	0	0,69431483	0,76150659
0,06095437	1,27133403	4,57810867	0	0,23728666	0,51811216	0,13061651
0	1,80099472	5,18056285	0	0,2224213	0,39541565	0,85879336
0,02003807	1,29579534	3,71038306	0	0,1653141	0,34732659	0,16698394
0	0,86619609	2,78563317	0	0,20263199	0,21153889	0,11356298
0,04056047	1,2979351	4,80088496	0	0,18436578	0,41666667	0

Fusobacteriu	Klebsiella	Kluyvera	Lactobacillus	Lawsonella	Leclercia	Leptotrichia
0,39415562	0	0	0	0,24464832	0,06795787	0
1,74420838	0,05378616	0	0	0,1344654	0	0
1,55941697	0	0,08274457	0	0	0,21640889	0,08274457
0,89337925	0,05522708	0,10395686	0	0,22415697	0,06822169	0,14943798
0,5994173	0	0,143287	0,09552467	0,07880785	0,07641974	0
0	0	0,10074439	0,30223317	0	0,12313203	0
1,76569642	0,02405581	0,08660091	0	0,47630503	0,13471253	0
1,75711744	0	0	0,57829181	0,29655991	0	0,20759193
0,05002501	0	0,00625313	0	0,53151576	0	0
1,76661264	1,13452188	0	0	0	0,19448947	0
0,95367242	0,10670461	0,12004268	0,23786235	0,38235817	0,04446025	0
1,93323217	0,13050076	0,11229135	0,50075873	0,09408194	0,09104704	0
1,87137019	0	0	0,48756005	0	0	0
0,02061856	0	0,32474227	0	0,42268041	0,07731959	0,65979381
1,30682608	0	0,24468233	0,10565828	0,0583901	0,01112192	0,10009732
0,73588475	0	0,00518229	0,77216075	0,44308553	0,06218744	0
0,88065195	0	0	0,4008938	0	0	0,40746583
1,14340989	0	0,18354085	0,2033831	0,08432958	0	0
0	0	0	0,5200208	0	0,44721789	0
0,82752436	0	0,02242061	0	0,3057356	0,05095593	0,24051201
0,41687897	0,0787438	0,15285562	0,26402335	0	0,18064755	0
2,03051575	0	0,20885305	0,28427221	0,48732378	0,19144863	0
0,59831824	0	0	0	0	0	0
0,04670933	0,02335466	0,25223037	0	0	0	0
1,91410963	0,04565168	0	0,1434767	0,41738677	0	0
0	0	0,26423754	0,44503164	0	0	0
0,89123382	0,36005846	0,09981819	0,12833767	0	0,00712987	0
0,04068555	0,0864568	0	0,21359915	0	0,07628541	0,54416925
0	0	0,24418037	0	0	0	0
0,80811808	0	0,08487085	0	0,10701107	0,02214022	0
0,97040272	0	0,23513604	0,4329489	0,26499459	0,08584332	0
0	0	0	0	0	0	0
0	0	0,50377834	0,40823417	0	0	0
1,73858159	0	0	0,3327978	0,07459261	0	0,36148726
1,7251345	0,04446223	0,05335468	0,22675737	0,22675737	0,06669334	0
0,381259	0	0	0,38973142	0	0	0
0	0	0	0	0	0	0,55727554
1,23472878	0,13357224	0	0	0,27691806	0,04235217	0
1,37593162	0	0,21368635	0,34919477	0	0,16156773	0
1,59602839	0,06974931	0,09846962	0,50465679	0,49645099	0,0041029	0
1,92979392	0	0,25323088	0,62871114	0,14844569	0,34055187	0
0	0	0	0	0	0	0
0	0,07891223	0,18210514	0	0,35206993	0	0,15175428
1,3787384	0	0,23842845	0,22806199	0,29026072	0,02591614	0
0,7488205	0,11686794	0,16015236	0	0,32896161	0	0,1125395
1,66870934	0	0	0	0,18855473	0	0
0	0,3716288	0	0	0	0,26544914	0

0	0	0	11,5792263	0	0	0
2,75508066	0	0	0	0,24093861	0	0
0,5101475	0	0	0	0	0	0
0,91409577	0	0	0	0	0	0,58169731
0	0	0	0	1,76744186	0	0
0	0	0	0	0	0	0
0,51068022	0	0,05443799	0	0	0	0
0,75471698	0	0,5163853	0	0	0	0
0	0	0	0	0	0	0
0,32856908	0	0	0	0	0	0
0,73915372	0,24638457	0,10176754	0	0	0,21424746	0
0,89775006	0	0,02895968	0,32301181	0	0	0
0,5671968	0,05653124	0	0,14132811	0,03957187	0	0,27134997
0	0	0,39834454	0,29746508	0,10346611	0,02327988	0
1,82836113	0,0587268	0,19967113	0,18401065	0	0,0195756	0,19184089
1,56086866	0	0	0,22969305	0	0	0
0	0,2946624	0	0	0,1810069	0,05893248	0
1,52802415	0	0,40456851	0,15560327	0	0,00622413	0,10581023
1,30316146	0	0	0,37636432	0,45634174	0	0
1,51631971	0,00642508	0	0,10280134	0,16062709	0,03533796	0,44011822
0,46137308	0	0,0808592	0,0313924	0,01902569	0,00380514	0,08466434
0	0	0	0,20755222	0	0	0
1,45607309	0,00815727	0,07341545	0,15498817	0,24063953	0,31405498	0
1,43295803	0,05511377	0	0,24801197	0,16927801	0,04724037	0
0,75429272	0	0,17988553	0,20645953	0	0,09811938	0
0	0	0	0	0	0	0
0,68698639	0	0	0	0	0	0,27392495
1,42631143	0	0	0,29882229	0,2159556	0	0,17326671
1,03209539	0	0	0	0	0,12821061	0
0,63212143	0,02214438	0,00402625	0,28989008	0,35028385	0,12481379	0
0,84578419	0,1013543	0	0,12232416	0	0	0,23765837
0,80656516	0,01609108	0	0	0,20918398	0,026148	0,11263753
0,8496767	0	0,09618981	0,17991058	0	0,00356259	0
0,65330837	0	0	0,16731068	0,15137633	0	0
0,87492785	0,01822766	0,09873318	0,23999757	0,22480785	0,03949327	0,09417626
0,99911719	0,05324888	0,06866303	0,23121225	0,05745274	0,01121029	0,02522315
0,54603086	0	0,07403808	0	0	0,02082321	0
1,45087975	0,03857831	0,10231638	0,14760395	0	0	0,38410574
0,94342137	0,01066013	0	0,23452283	0,0746209	0,0079951	0
1,05455813	0,01452559	0,00871536	0,30213236	0,25274534	0,06391261	0,20335832
0,47924913	0	0,01867204	0,03983369	0	0	0
1,26108847	0	0,03356509	0,25893071	0	0	0
1,5674511	0	0	0	0,13942287	0	0
1,2954796	0	0,00551268	0,1323043	0,20948181	0,0137817	0
1,30461597	0,00948812	0,04744058	0,18976232	0	0,2372029	0,14232174
0,78045716	0,24646016	0,07007201	0,23679505	0	0,10631615	0
1,31926897	0,02056767	0,07051772	0,27913263	0	0,06464124	0
1,10958235	0,08654742	0,188629	0,34618969	0,17309485	0,08654742	0,12427322
1,66020642	0,00934012	0,14477187	0,09106617	0,24284313	0,18680241	0,15411199
0,91425065	0,05522319	0	0,28225188	0,22396073	0,06749501	0,09203866

0,54873269	0,05972601	0,1978424	0	0	0,03732875	0
0,8794845	0,19810171	0,11537792	0,10449321	0,28517938	0,01088471	0
1,18933613	0	0,14519168	0,29347255	0	0,08340799	0
0,88167518	0,11688876	0,12189827	0,24379655	0,21039976	0,00667936	0,10019036
0,87064954	0,02449398	0,12915006	0,19817854	0	0,01336035	0
1,19469027	0	0,22123894	0,32079646	0	0	0,2359882

Ligilactobacil	Limosilactob	Mediterraneil	Microvirga	Morganella	Muribaculum	Parabacteroi
3,13965341	0	0	0,67278287	0,00679579	0	0,1019368
2,19754889	0	0	1,16024434	0,21514465	0	0,8221599
2,05588441	0	0,4073579	0,31824836	0	0	0,0381898
1,85498018	0,41907608	0	0,76668183	0	0	1,13702813
1,61914314	0,15522759	0	0,92897741	0	0,09552467	0,1003009
2,31712095	0	0	0,57648178	0,01679073	0	0,35820227
1,87154198	0	0	1,92927592	1,68871783	0	0,99591051
1,5754745	0	0,23354093	1,51616251	0	0,56346382	0
2,3074037	0	0	0,8004002	0	0	0,9192096
0	0	0	0	0	0	0
1,6894896	0,27120754	0,19784812	1,05815401	0	0	0,92921928
2,42792109	0,29438543	0,33687405	0,72837633	0,47040971	0,02427921	1,14112291
2,60271026	0,23661002	0	2,66007027	0,13623001	0,59511006	0
2,1185567	0,7371134	0,14948454	1,53092784	0	0,72680412	0
1,9630196	0	0,55331572	1,05658279	0	0,56999861	0,71458362
1,57800637	0,39385381	0,11141917	0,27207006	0,20470033	0	0,47417926
2,74710831	0	0	2,97712934	0,01314406	0	0
2,01150851	0,51341832	0	0,4216479	0	0	0,14385634
0	0,75923037	0,21840874	1,72646906	0,15600624	0	0
1,7854959	0,13860014	0,23032082	0,55847703	0	0,17528841	0,75210958
1,50539627	0,17601556	0	0,3242392	0	0,38908704	0,89397378
2,00150838	0	0	0,53953704	0	0	0,88762546
5,02910737	0,98641656	0	0	0	0	0
1,53673689	0,17749545	0	1,05563081	0,08874772	0	1,53206595
1,16085695	0	0	0,49238595	0	0	0,21195422
3,00396356	0,42417078	0	0,32682011	0	0	0
1,66482478	0,2424156	0,22815586	0,78428577	0	0	0,82350005
2,40044754	0	0,23394192	0,60011189	0,32039872	0	0,65605452
5,9091649	0	0	0	0	0	0
1,7195572	0,16236162	0	1,17712177	0	0,1402214	0,16605166
1,38095771	0,21274213	0	0	0	0,39935804	0,26126227
4,26400915	0	0	0	0	0	0
3,30930253	0,29531834	0	0,80778251	0	0,2345175	0
1,93940785	0	0	0,37296305	0	0	0
1,84518252	0,14672536	0	0,60468632	0	0,30678938	1,19603397
2,49089215	0	0	0	0	0	0,33889689
6,84210526	0	0	0,51083591	0	0	0
1,73643916	0,35184884	0	0,48216322	0,48867894	0	0,30298094
1,99614322	0	0	0	0,12508469	0,09902538	0
1,90374595	0,35284946	0	0,44721618	0	0	0,81647725
3,44044708	0	0	0,0261963	0	0	0
2,840537	0	0	0,25376555	0,01637197	0	0
2,47055967	0	0	0,42491198	0	0	1,23224475
1,94889338	0	0	0,70491888	0	0	0,14513036
1,95212743	0	0	1,04748301	0	0	0,05626975
3,3939851	0,91449043	0	0	0,33939851	0	0
0,04247186	0,7114037	0	0	0	0	0

0	1,48383678	0	0	0	0	0
3,0902996	0,58663314	0	0	0,16760947	0	1,64466792
2,43983587	0	0	0	0	0	0
2,87732419	0	0	0	0	0,27007375	1,45424327
0,09302326	0	0	0	0,04651163	0	0
0	0	0	2,89179104	0	0	0
0,87100788	0	0	0	0,12183741	0	0
0,07944389	0	0	0	0	0	0
5,64247243	0	0	0	0	0	0
3,63068835	0	0	0	0	0	0,52571053
2,69416176	0,32672737	0	2,61381896	0	0,27852169	0
1,74203609	0,11806638	0,21831143	0,75295166	0,05791936	0	0,89552239
1,73362478	0,31845934	0,11306249	1,42270295	0	0,49559056	0,80839677
1,65804449	0	0	0,92602173	0	0	0
1,97322058	0,50896563	0,13702921	1,85576697	0	0,097878	0,77127868
1,62873251	0	0	0,66297766	0,13572771	0	0
2,05421788	0	0	1,01027109	0,36201381	0	0
1,51557589	0,14004295	0	1,41287773	0,11825849	0,31743068	1,06743846
2,22054949	0,11761385	0	0,03763643	0	0	1,07263831
1,97892573	0	0	1,48098175	0,18311488	0	1,17579029
0,89230506	0,08846948	0,10083618	1,65143026	0	0	0,15981583
2,28976968	0	0	0,6293519	0	0	0,38162828
2,08826169	0	0	1,00742312	0	0	1,01965903
2,22423431	0,29918904	0	1,26368003	0	0,20864499	1,26368003
2,34464432	0	0,14309076	1,80907604	0,25347506	0,37612428	0,8217498
0,23633147	0	0	1,65846647	0	0	0
1,03265359	0,20870473	0	0	0	0	0,34131919
1,62468925	0,26868895	0,08286669	2,00386711	0	0	0
1,42313774	0,05128424	0	1,28424292	0	0	0,41027394
1,49172605	0,12884004	0	1,5823167	0	0,22748319	0,88980151
1,21275666	0,16251638	0	0,74792486	0	0	0,61861075
1,42406018	0,22125229	0	0,30170767	0,16091075	0,0905123	0,69392763
1,14002743	0,25116229	0,07125171	0,88708385	0,05878266	0,21197385	0,72854878
1,44205872	0,50591563	0,21909732	2,44990639	0	0	0
0,96302822	0,13366953	0,10329009	0,5741714	0	0,09873318	0,44202084
1,03555064	0	0,07426818	1,28497961	0,06866303	0	0,21860068
0,83986951	0	0	1,27021587	0	0,05321487	0,39564101
1,37707778	0	0,11238028	1,26302018	0,10902565	0	0,75479294
1,5190683	0,56765184	0	1,73760094	0	0,2078725	0,02398529
1,45546453	0,20626344	0,24402998	0,93544826	0,09877404	0,00581024	0,22369415
0,57634376	0,04481291	0,10331864	1,25974058	0	0,04854731	0,41700899
1,71661472	0	0	0,68568689	0	0	0
1,58435084	0,34221978	0	1,82094723	0	0	0
1,41124587	0,08820287	0,15435502	0,3693495	0	0,1185226	0,84068357
2,1158499	0,05218464	0,16129797	0,10911334	0	0,11860145	0
1,6841444	0,27303919	0,24887643	0,50258542	0,18605325	0,16189049	0,96167786
1,7982018	0,18804725	0,32614444	0,94905095	0,02644414	0,17629429	0
1,39363544	0,14424571	0	0,98974746	0,1131774	0,3018064	0,82109094
1,88903937	0,25685331	0,16812217	1,22355578	0	0,32223416	0,44832578
1,50329805	0,62893082	0,07363092	1,46648259	0,09203866	0,5890474	0,46019328

1,70592407	0,22397253	0	0,35089029	0	0	0,38448617
1,44113549	0,31565657	0	0,94914664	0,17415535	0	0
1,49825461	0,34598869	0	0,71978005	0,27493744	0	0,44175342
1,08539558	0,05677454	0,23711719	1,50786494	0	0,26550446	0,75810707
1,0621479	0,19149836	0,08461556	1,71457837	0	0	0
1,65560472	0,44985251	0	1,03244838	0,25073746	0,11061947	0,2359882

Paramuribac	Parasutterell	Peptacetobac	Peptoniphilu	Phocaeicola	Porphyromor	Prevotellama
0,42813456	0,56405029	0	0	0	0	0
0,64927581	0	0,53786162	0,5148104	1,11414192	0,74916439	0
0,43281777	0	0	0,31824836	1,52122717	0	0
0,37034631	0	0,51653564	0,15593529	0	0	0,15268664
0,45851841	0,31284329	0,51822133	0,28896212	0,80479534	0,27702154	0
0	0	0	0,58207869	0	0,17910114	0
0	0,29348088	0,47149387	0,91412076	1,31825836	0	0,21169112
0,20759193	0	0,48932384	0,48561684	1,04908066	0,56717082	0,21871293
0,2813907	0	0	0,76288144	0,86918459	0	0
0	0	0	0,72933549	0	0	0
0,13338076	0	0,40903432	0	0,41348035	0,44460253	0,29121465
0,46433991	0	0,53414264	0	0,95902883	0,4370258	0
0	0,20793002	0	1,15437012	0	0,33699003	0
0,21134021	0	0	0,24742268	0	0	0,3814433
0	0	0,48102322	0,66453496	0,83136383	0,36146253	0
0	0	0	0,48713497	0,72552017	0,25911435	0,27466121
0	0	0	0	0	0	0
0,28275212	0,28275212	0,23314649	0,19346198	0,7887296	0,448931	0,12649437
0	0	0,96723869	0,4576183	1,80967239	0,4576183	0
0	0	0,39949452	0,26904733	0,28331499	0,27720028	0,18140312
0,03705591	0	0	0	0,99587753	0,21770346	0,09263977
0	0,57434588	0,21465452	0	0,03480884	0	0
0	0	0	0	2,23156533	0	0
0,48110608	0	0,5698538	0,28025597	0	0	0,30828156
0,39456093	0,46303845	0,42716927	0	0,74673101	0,07173835	0
0,17384048	0,02781448	0,3963563	0,23642306	0	0	0
0,1818117	0,26380521	0	0,29232469	0,72011693	0	0
0,02034278	0	0	0,40176982	0	0	0
0	0	0	0	0	0	0
0	0	0,43542435	0,27675277	0,90774908	0	0
0,46280745	0,14556041	0	0,69794349	0,86589781	0,13436345	0,11196954
0,68616239	0	0	0	0	0	0
0	0	0,72092417	0,62538001	0	0	0
0,21230204	0	0	0,27541887	0,94675235	0	0
0,44906852	0,24454226	0,15117158	0	0,16451025	0,19118759	0
0,27111751	0	0,75404558	0,46598322	1,4064221	0,20333813	0
0	0	0	1,08359133	0	0	0
0,18895586	0	0,14986154	0,29972308	0,51474181	0	0
0,12508469	0	0	0,77135561	0,5107625	0	0
0,13949863	0,27899725	0,40208427	0,45952488	1,13240061	0	0
0,28815927	0	0,41040866	0	1,25742228	0	0
0,6794368	0	0	0	0	0	0
0	0	0,38242078	0	0,58273643	0	0
0,48722335	0	0,54423884	0,2487949	0,80858342	0,21251231	0
0	0,51075618	0	0,09522573	0,7488205	0,26403497	0
1,2444612	0,14141605	0	0,65994155	0	0	0
0	0	0,59460607	0	0	0	0

0	0	0	1,45733969	0	0	0
0	0	0,80662057	0	1,47705845	0	0
0	0	0	0	0	0	0
0	0	0	0	0	0,31162356	0
0	0	0	0	0	0,10852713	0
0	0	0	0	0	0	0
0	0	0	0,09072999	0,20738283	0	0
0	0	0	0,2979146	0	0	0
0	0	0	0	0	0	0
0	0	0	0	0	0	0
0	0	0,05891805	0,57311194	0,01071237	0	0
0,11806638	0	0	0,45889953	0,49899755	0,49231455	0,16039207
0	0,19974373	0,34860933	0,50689681	0,66518429	0,50689681	0,16582498
0	0	0,71391619	0,81738231	0	0,12415934	0,4009312
0,12528385	0,28580377	0	0,52854123	0,7360426	0,24665257	0
0,26623512	0	0	0,71518062	0,72562122	0	0
0	0	0,25677724	0,37043273	0	0,3746422	0
0,31120655	0,57262005	0,22406871	0,19917219	0,85893007	0,2707497	0
0,28227324	0,14584117	0,20700038	0	1,34550245	0,1740685	0
0,39514264	0,56540735	0,06103829	0,5846826	0,62644564	0,62965819	0,26985351
0,07134635	0,11700802	0,15981583	0	0,21308778	0,06183351	0,16837739
0	0,49544724	0	0	0	0	0
0	0,59548087	0	0,77086222	1,31332083	0,40786361	0,06525818
0,21651838	0	0	0,3818597	1,16919928	0,5629478	0,26769546
0,14717907	0	0	0,57645135	0,56623058	0,4946852	0,23303352
0	0	0	0	0	0	0
0,15000652	0,29349102	0	0	0,26740293	0	0
0	0	0,43693343	0	0,83368907	0,31640007	0
0,11538955	0,48292662	0,33121074	0,38249498	0,42950553	0,22650541	0,13248429
0	0,26371945	0,19124693	0,23956194	0,58783267	0,57978017	0,20332568
0,31629533	0,08038445	0	0,32153779	0,52075142	0,20795107	0
0,31981013	0,07442122	0,17297906	0,52698272	0,72812117	0,34595812	0,17901321
0	0,20484868	0,38654055	0,38654055	0,48451166	0	0,09618981
0	0	0,64135761	0,38242441	0	0,386408	0,19917938
0,19746635	0,11240393	0,2491114	0,25214935	0,5255643	0,51037458	0
0,1541415	0	0,02242058	0,29006628	0,33070359	0,18076594	0,11910934
0,10874343	0,17121307	0,10874343	0,23136901	0,3516809	0	0,06709701
0	0,10063906	0,36733256	0,2952079	0,69944145	0,47971284	0,05367416
0,52501133	0	0,17589212	0,47171068	0,66092796	0,32246889	0
0,31665795	0,26146069	0	0,12201499	0,76985649	0,36313985	0,13363547
0,07966739	0	0	0,16306919	0,26016382	0,26265342	0
0,34524095	0,08151522	0	0,39798609	0,03356509	0,31167586	0
0	0	0,60416579	0,36334446	0,06759897	0	0
0,15711136	0,12127894	0,47684675	0,2646086	0,44101433	0,42723264	0
0,25143508	0	0,26566725	0,74007306	0,83969828	0,77802552	0,13283363
0,14739284	0	0,16189049	0,31169961	0,76837578	0,42284831	0
0,26737968	0	0,25562673	0,52006817	0,95198919	0,54651231	0
0,33065554	0	0,44383294	0,34618969	0,74342018	0	0,23523146
0,88497642	0,48568627	0,50670154	0,42030542	0,65847849	0,1938075	0,37593985
0,2945237	0	0,47553306	0,60131922	1,04310477	0,57370762	0,3558828

0,01866438	0	0,07092463	0,29863003	0	0	0,08958901
0,22640195	0	0,37225705	0,55294323	0,46804249	0,18068617	0
0,14828087	0	0,23786723	0,18535109	0,73522597	0	0
0,32895835	0,18201249	0,22876799	0,43081856	0,47590422	0,55438667	0,26383462
0,24271304	0	0,17591129	0,23603287	0,59453562	0,12246988	0,14251041
0	0,14749263	0	0,44985251	0,09955752	0,39454277	0

Raoultella	Romboutsia	[Ruminococc	Salmonella	Sangeribacte	Segatella	Staphylococc
0	0	0	0	0,36017669	0	0
0,46486611	0	0,06531177	0,08067924	0,4533405	0,45718237	0
0,08910954	0	0,12729934	0	0	0,28005856	0
0,1884218	0,29887597	0	0,09096225	0,07471899	0	0,47755182
0,08836032	0	0,08836032	0	0,42747289	0,20537804	0,32717199
0	0,5876756	0,59886942	0,1063413	0	0	0,374993
0,61101756	0,58215059	0,0384893	0	0	0	0
0,44483986	0,34475089	0,011121	0	0,48932384	0,11120996	0
0,55652826	0	0	0,20635318	0	0	0,26263132
0,84278768	0	0	0	0	0	0
0	0	0	0	0,30899876	0,24230838	0,11559666
0,07587253	0	0	0	0	0	0,16388467
0,19359002	0,78870008	0	0,19359002	0	0	0
0,18041237	0	0,06185567	0,06185567	0	0	0
0,45877937	0	0,07229251	0	0,23634089	0	0,39482831
0,12178374	0	0	0	0,22542948	0,01554686	0,28243464
0,39432177	0,06572029	0,08543638	0	0	0	0
0,18106057	0	0	0,06944789	0,02976338	0	0,33235776
0,21840874	0	0	0	0	0	0
0,25274143	0,30369736	0,04076475	0	0,30165913	0,26904733	0,35057682
0	0,5651026	0,06484784	0	0	0	0
1,21250798	0	0,05221326	0,07541916	0,20885305	0	0
0,4851229	0	0	0,11319534	0	0	0
0	0	0	0	0	0,14946985	0,20085011
0,02282584	0	0	0	0	0	0,89672938
0,75099089	0,70926917	0,0347681	0	0	0	0
0,23528573	0	0,04990909	0	0	0	0
0,24411331	0,13731374	0,21359915	0	0,04068555	0	0,87473936
0,35813121	0	0	0	0	0	0
0,1402214	0	0	0,04797048	0	0,20664207	0,44649446
0,2873885	0	0	0	0	0	0
0	0	0	0	0	0,60447639	0
0,38217667	0,81646834	0	0	0	0	0
0,02868947	0	0	0	0	0	0
0	0	0	0	0,22231115	0,19118759	0
0,42362111	0	0	0,27958993	0	0	0
0	0	0	0	0	0	0
0,26062877	0,39420101	0,0162893	0,11728295	0	0	0,24433947
0,32834732	0,27101683	0	0	0,31792359	0,27101683	0
0	0,22155664	0	0	0	0	0,32823206
0,13971359	0	0	0	0	0	0,23576668
0,72855272	0	0	0	0,33562541	0	0
0,17603496	0	0	0,22459633	0,18210514	0	0,30350856
0,12439745	0	0	0	0	0,1917794	0
0,10388261	0,63195256	0	0	0	0,1125395	0,41120201
0	0	0	0	0,5185255	0	0,82021307
0	0	0	0	0	0	0

0	0	0	0	0	0	0
1,07898596	0	0	0	0	0	0
0	0	0	0	0	0	0
0	0	0	0	0,44666043	0	0
0	0	0	0	0	0	0
0	0	0	0	0	0	0
0	0	0	0	0	0,11146827	0
0	0	0	0	0	0,93346574	0
0	0	0	0	0,35906643	0	0
0	0	0	0	0	0	0
0,41778254	0	0,06427424	0	0	0	0
0,21831143	0,33860548	0,04009802	0	0,21831143	0	0,10024504
0,13190623	0	0	0	0,30526871	0	0,26758122
0,29487843	0,27935851	0	0,06983963	0	0	0
0,3092945	0,49330514	0,01566048	0	0,53637147	0	0
0,21925245	0	0	0,22447275	0,37064105	0	0
0,13049335	0,6693046	0	0,13470281	0	0	0
0,28631002	0	0	0	0,06224131	0,37033579	0
0	0	0,39988709	0	0,29168235	0	0,38577343
0,28912876	0	0	0,077101	0,32767926	0	0,23451555
0,0313924	0,0494668	0	0,0561258	0	0,09607976	0,07610278
0,39501875	0,42849491	0,47536154	0	0	0	0
0	0	0	0,02855045	0	0	0,28958316
0,10629084	0	0,28344225	0,03149358	0,49602394	0	0
0,26573998	0	0,00613246	0	0,13082584	0	0,31888798
0	0,1271491	0	0	0	0	0
0,33044915	0	0	0	0	0,23696682	0,4587156
0,30635562	0	0	0,0853778	0	0	0
0,18376854	0,19872644	0,03632634	0	0	0,1132527	0
0,30196884	0	0	0,06240689	0,23352257	0,16910255	0,28183758
0,29357798	0,19571865	0,02271734	0	0,10659677	0	0,15727392
0	0,15688799	0	0,01005692	0,1287286	0,09855784	0
0	0,22622419	0	0,05165749	0,09262723	0	0,14428472
0	0,22308091	0,06772099	0	0	0	0
0,05316402	0,10784701	0	0	0,18531458	0,11696084	0,21417505
0,03643345	0,14012864	0,00700643	0	0,02942701	0,18917366	0,12751706
0,18625205	0,21980056	0	0,01850952	0	0,08907707	0,07403808
0,13250809	0	0,04528758	0	0,27843473	0	0,16605445
0,21320257	0	0,03731045	0	0,40508488	0	0,12792154
0,03486143	0	0	0,14235082	0,20916855	0,37185521	0,37476033
0,05477133	0	0,08962581	0,00995842	0,07095377	0,0435681	0
0,20618557	0	0	0	0,30208583	0	0
0	0	0	0	0	0	0
0,29768467	0	0	0	0,12679162	0,23153252	0
0,34631624	0	0	0,04269652	0	0	0,43170928
0,09906732	0,2416276	0	0,02416276	0,2319625	0,09423477	0,25129271
0,02056767	0	0,01175295	0,04113534	0,37903273	0	0
0	0	0,00887666	0	0,17531401	0	0
0,21248774	0,61644795	0	0,0700509	0	0,05137066	0,14944193
0,13499003	0,54916398	0,03374751	0,06442706	0	0	0,18100936

0,11571914	0	0	0	0	0,29489716	0
0,22640195	0,48981191	0,01306165	0	0,34613375	0,18504006	0
0,12974576	0,32127522	0	0,13901331	0	0	0
0,08349197	0,12857763	0,08015229	0	0,17032361	0,07681261	0,10353004
0,11578971	0,43643813	0	0	0,09797591	0	0,05121468
0,23967552	0	0,02949853	0	0	0	0

Trueperella	Turicimonas	Zhenhengia
0	0,03397893	1,29119946
0	1,11414192	0,81447616
0,28642352	0,09547451	0,84017567
0	0,35410305	0,63673575
0,22687109	0,67822515	0,62329847
0	0	0,89550568
0	0,60139524	0,91893192
0	0,36328588	0,65243179
0	0,9192096	1,11930965
0	0	0
0,35345901	0,31789081	0,68468789
0	0	0,72837633
0	0,04302	1,31211013
1,11340206	0,6185567	1,12886598
0	0,35868205	0,63116919
0,28243464	0,23579406	0,5545047
1,04495268	0,38117771	0,92008412
0	0,1761	0,43156903
0	0	1,39365575
0	0,40560923	0,47694754
0	0	0,6438464
0	0	0,95724314
0	0,63065977	1,2774903
0	0	1,20510066
0	0,32608341	0,30651841
0	0,50066059	1,15430081
0,3636234	0	0,40640262
0,0813711	0,39159843	0,98153893
0	0	0
0,46125461	0,17712177	0,81549815
0	0,3694995	0,68674654
0	0	1,78075478
0	0	1,73716668
0,41886619	0,17787468	0,63690613
0	0,58690143	0,74696545
0	0	0,94891129
0	0	0
0,23456589	0,64179834	0,42677961
0	0,44300829	0,7036014
0,21745374	0,43080458	0,79596275
0	0,50646175	0,74222843
0	0	0
0	0,64950832	0,99550807
0	0	0,85004924
0	0,90464442	0,72284985
0	0	0
0	0	2,29348057

0	0	4,02755697
0	0,34569453	0
0	0	0
0	0	0,34278591
0	0	0
0	0	0
0	0	0
0	0,83416087	0
0	0	0
0	0	0,64070971
0,05891805	0	1,17836101
0	0,3029628	0,6059256
0	0,34860933	0,38441245
0	0,66994309	0,66994309
0,37976666	0,45415394	0,716467
0,48026728	0,34453957	0,88745041
0	0,44620306	0,95554807
0,03734479	0,31431861	0,75000778
0,48927362	0	0,8185924
0,20560267	0,51400668	0,52043177
0,01522056	0,17789024	0,23972375
0	0	0,92394215
0,33444816	0,79941268	0,93400767
0,03936698	0,98417447	1,00385796
0,31275552	0,21054783	0,64390842
0	0	0
0	0	0,2587069
0,18582226	0,45451121	0,36411119
0	0	0,51711612
0	0,43886138	0,50126827
0,01747488	0	0,36871997
0	0,35400366	0,46060704
0,20128609	0,27075652	0,33310177
0,40632594	0,28283472	0,84452058
0,26126318	0,30683234	0,4010086
0	0	0,34471645
0,13419403	0,20823211	0,28111335
0	0,10063906	0,65583119
0,34112411	0,47437572	0,46105056
0	0,10167916	0,6333159
0,00746882	0,20539249	0,28132547
0	0	0,97338768
0,1901221	0,33799485	1,1407326
0	0,38864388	0,53472988
0,02846435	0,09488116	0,62147161
0,38177161	0,5944039	0,57024114
0	0,56414174	0,82564494
0,27739559	0,42607962	0,56144867
0,17979732	0,4576659	0,60710783
0,75471698	0,22702869	0,62893082

0	0,04852738	0,76897234
0	0,1197318	0,50069662
0,19770782	0,87423929	0,66726391
0,20205056	0,30224092	0,37571386
0	0,09352246	0,26943374
0,02581121	0,31342183	0,60471976

Supple. Fig. 4E

Sample	PCoA1	PCoA2	Group
4914	-0,22670184	-0,05198125	CP
4917	-0,07928393	0,07744672	CP
5960	0,00351164	0,08741834	CP
6086	0,04952216	0,0804196	CP
5217	0,06679203	0,03744967	CP
5502	-0,05591808	0,04829776	CP
5820	-0,0605881	0,05606186	CP
5646	0,00525426	0,0774544	CP
5703	-0,122166	0,0810017	CP
6278	-0,31209334	-0,16231429	CP
6373	0,05905105	0,07060285	CP
6389	-0,02388726	0,11425856	CP
6493	-0,19547611	-0,0408733	CP
6800	-0,1523288	0,02608042	CP
7129	0,01891445	0,07823394	CP
7671	0,07270743	0,06901964	CP
7733	-0,11024021	-0,02294832	CP
8044	0,0176165	0,08676627	CP
8068	-0,2219061	-0,158014	CP
8069	0,13608311	0,02804597	CP
8073	0,07306997	0,08510688	CP
8108	-0,08527144	0,10121115	CP
8221	-0,17812813	0,03792987	CP
8279	0,0275266	0,03509446	CP
8295	0,22004726	0,01734269	CP
8390	-0,19721366	0,05957293	CP
8393	0,0699209	0,05375777	CP
8405	-0,12472217	0,06464756	CP
8409	-0,3914462	-0,18253201	CP
8469	0,07470614	0,05920913	CP
8513	0,06580712	0,07941607	CP
8560	-0,22037498	0,03236054	CP
4973	-0,27190385	-0,00568334	IPMN
5045	0,00661403	0,09752703	IPMN
5948	0,03283054	0,11023492	IPMN
6106	-0,12896833	0,04756424	IPMN
6163	-0,35298308	-0,16780326	IPMN
6392	0,09252279	0,0585401	IPMN
6457	-0,04290721	0,09541538	IPMN
6715	0,01121903	0,08214018	IPMN
6956	-0,13071336	0,07010137	IPMN
7089	-0,13577216	0,07147263	IPMN
7269	-0,05323789	0,1112648	IPMN
7319	0,00338341	0,07893311	IPMN
8023	0,0322168	0,07144198	IPMN
7711	-0,11118977	0,08478705	IPMN
8066	-0,27471236	-0,00831084	IPMN
8092	-0,35412484	-0,13930599	IPMN

8198	-0,07940457	0,02354304	IPMN
8207	0,0656895	-0,06706461	IPMN
8210	-0,03285993	0,00207339	IPMN
8267	-0,38442299	-0,26728248	IPMN
8276	-0,33931021	-0,45447409	IPMN
8439	0,23686638	-0,24274639	IPMN
8494	-0,14494776	-0,10797755	IPMN
8503	-0,15063678	-0,01231083	IPMN
8265	0,06927361	-0,04132161	IPMN
7927	-0,132217	-0,02139904	PDAC
7963	0,07672387	0,03906992	PDAC
7977	0,10159211	0,02302198	PDAC
8001	0,03797965	0,01743566	PDAC
8030	0,0016825	0,0513471	PDAC
8035	0,04500261	0,0866816	PDAC
8048	-0,02212297	0,06261044	PDAC
8099	0,09294918	0,06046192	PDAC
8118	-0,06030837	0,12569591	PDAC
8145	0,07214031	0,06644357	PDAC
8197	0,31701866	-0,29628981	PDAC
8214	-0,07067464	0,05225532	PDAC
8215	-0,00011162	0,10656549	PDAC
8216	-0,03077305	0,07137379	PDAC
8219	-0,02860473	0,00529519	PDAC
8225	0,14345036	-0,63598158	PDAC
8231	0,24898228	-0,1094842	PDAC
8247	0,06046465	0,03255042	PDAC
8257	0,16094171	-0,01536933	PDAC
8272	0,12568015	-0,00091435	PDAC
8268	0,17871425	0,00026252	PDAC
8293	0,11657689	0,04591656	PDAC
8366	0,18343617	-0,02948816	PDAC
8376	0,06399784	-0,03146362	PDAC
8401	0,23301343	-0,04382336	PDAC
8417	0,25543877	-0,12958555	PDAC
8453	0,31657913	-0,21599465	PDAC
8455	0,15242591	-0,01030699	PDAC
8465	0,09774803	0,0106572	PDAC
8571	0,13513153	0,02525184	PDAC
8640	0,31999444	-0,27065456	PDAC
8209	0,03882673	0,06575623	PDAC
7968	-0,00786238	0,01137854	PDAC
8029	0,11684547	0,06536752	PDAC
8046	-0,00074632	0,10949956	PDAC
8059	0,09661664	0,07874237	PDAC
8061	0,07137291	0,06750117	PDAC
8085	0,13903259	0,02946478	PDAC
8217	0,08741715	0,05877233	PDAC
8222	0,05196234	0,06978025	PDAC
8260	0,06189861	0,01533504	PDAC

8273	0,11866472	0,00631565	PDAC
8359	0,05185182	0,07215423	PDAC
8381	0,20458064	-0,03087647	PDAC
8575	0,25800997	-0,15332379	PDAC
8644	0,06922481	0,04121233	PDAC

Supple. Fig. 4F

Genus	log2FC	p	Abundance	p.adj	-LOG10p.adj	Direction
Akkermansia	-0,44442368	0,00146744	1,46914229	0,00733719	2,13447001	IPMN_CP
Anaerobiospir	0,00600893	0,34645265	0,09855486	0,46409397	0,33339407	NS
Anaerostipes	1,03415567	0,00110585	0,08016761	0,00733719	2,13447001	PDAC
Bacteroides	-0,0672732	0,90232463	2,04921893	0,90232463	0,04463719	NS
Bariatricus	0,48115592	0,17091634	0,03595677	0,29468334	0,53064442	NS
Blautia	-1,06874656	0,1411509	0,14872534	0,27877106	0,55475231	NS
Citrobacter	-1,48233793	0,16219828	0,02893588	0,28963978	0,5381418	NS
Clostridium	0,28826078	0,07397673	0,53287356	0,19467562	0,71068844	NS
Corynebacteri	-2,13122457	5,827E-05	0,25894134	0,00072837	3,13764796	IPMN_CP
Cutibacterium	-0,29223745	0,34520402	0,69919263	0,46409397	0,33339407	NS
Duncaniella	0,23550887	0,04457322	0,17215266	0,1485774	0,82804726	NS
Enterobacter	-0,5749131	0,10403637	0,33219944	0,22942093	0,63936696	NS
Enterocloster	-0,99937397	0,53340585	0,07174108	0,60815209	0,21598779	NS
Enterococcus	-0,35971237	0,00115065	1,64400668	0,00733719	2,13447001	IPMN_CP
Escherichia	-0,57940511	9,4584E-08	5,70380686	4,7292E-06	5,32521327	IPMN_CP
Extibacter	-0,80385937	0,7043213	0,10870623	0,74927798	0,12535703	NS
Faecalibacteri	0,53727295	0,01607366	0,12403703	0,0730621	1,13630785	NS
Flavonifractor	-0,55538513	0,35271142	0,50041654	0,46409397	0,33339407	NS
Flintibacter	-0,00231079	0,07230977	0,31066992	0,19467562	0,71068844	NS
Fusobacterium	0,20316937	0,2652288	0,88907517	0,39004236	0,40888823	NS
Klebsiella	-0,36378619	0,08314344	0,04603809	0,20785859	0,68223202	NS
Kluyvera	-0,332712	0,84434758	0,08526926	0,87952873	0,05574997	NS
Lactobacillus	-1,08672105	0,22078899	0,27818273	0,34498279	0,46220257	NS
Lawsonella	-0,79577863	0,31663259	0,13433005	0,45233227	0,34454243	NS
Leclercia	-0,22750273	0,38453857	0,05142928	0,48067321	0,31815008	NS
Leptotrichia	0,03930113	0,11012205	0,07382161	0,22942093	0,63936696	NS
Ligilactobacillı	-0,5141716	0,00021136	1,91934393	0,00211359	2,67497983	IPMN_CP
Limosilactoba	-0,12864533	0,14781351	0,18805058	0,27877106	0,55475231	NS
Mediterraneil	0,44059729	0,02426663	0,05900641	0,09333318	1,02996393	NS
Microvirga	0,86331637	8,6435E-06	0,85148442	0,00021609	3,66537217	PDAC
Morganella	-0,45352569	0,48069952	0,07132803	0,58319238	0,23418816	NS
Muribaculum	0,39869967	0,02874974	0,09196505	0,10267765	0,98852407	NS
Parabacteroid	0,2535986	0,19124963	0,41476973	0,31874939	0,49655064	NS
Paramuribacu	-0,1005582	0,4898816	0,1776996	0,58319238	0,23418816	NS
Parasutterella	0,79593665	0,02172814	0,10050659	0,0905339	1,04318877	NS
Peptacetobac	-0,10541887	0,53517384	0,21269186	0,60815209	0,21598779	NS
Peptoniphilus	0,26046831	0,05486769	0,32922135	0,16137555	0,79216226	NS
Phocaeicola	0,05488889	0,37644609	0,51308812	0,48067321	0,31815008	NS
Porphyromon	1,31082906	3,0006E-05	0,18975648	0,0005001	3,30094656	PDAC
Prevotellamas	1,24932579	0,00143613	0,06662228	0,00733719	2,13447001	PDAC
Raoultella	-0,47963641	0,89073308	0,19733744	0,90232463	0,04463719	NS
Romboutsia	0,29182424	0,09281235	0,12966886	0,22098178	0,65564354	NS
[Ruminococcu	0,43412045	0,23472184	0,03290648	0,35563915	0,44899044	NS
Salmonella	0,04076458	0,11011089	0,02962499	0,22942093	0,63936696	NS
Sangeribacter	0,49700569	0,05151841	0,11502432	0,16099505	0,79318749	NS
Segatella	-0,31240411	0,5915652	0,06972423	0,65729467	0,18223989	NS
Staphylococcu	-0,34976745	0,61454687	0,12438415	0,66798573	0,17523282	NS
Trueperella	0,58275798	0,00143234	0,10903339	0,00733719	2,13447001	PDAC

Turicimonas	0,20645439	0,21883278	0,27398572	0,34498279	0,46220257	NS
Zhenhengia	-0,32688399	0,15053637	0,68417987	0,27877106	0,55475231	NS

Supple. Fig.6B			
16S rRNA FISH		Bacteroides FISH	
**Donor**	**PDAC**	**Donor**	**PDAC**
0,01958941	0,02820062	0,01322285	0,01527534
0,01030407	0,01209044	0,00449896	0,00403015
0,00388723	0,02273014	0,00251527	0,02020457
0,07422963	0,1055592	0,00437777	0,02595717
0,03102177	0,5508733	0,00221521	0,01923095
0,00276901	0,03266208	0,00084031	0,0007748
0,00471866	0,00464882		0,0216424
	0,1973494		0,0216424
	0,2527939		0,1860035
	0,2249916		0,0166752
	0,03622543		0,1655254
	0,4656127		0,02754048
	0,2187008		0,06379631
	0,03975182		0,0469239
	0,08442219		0,02343403
	0,07103159		0,001218
	0,04487368		
	0,00669902		
	0,00294544		

Supple. Fig.6C			
Probe	probe dilution	16S FISH	Bacteroides FISH
1	1	0,05	0,03
1	10	0,07	0,04
1	100	0,23	0,05
1	1000	0,77	0,135

Supple. Fig.6D

Gram-negative counts				
**Donor**	**CP**	**IPMN**	**Adjuvant**	**Neoadj.**
7	17	13	14	10
6	26	14	26	30
11	14	26	23	25
14	26	15	16	32
8	28	2	29	29
9	14	23	17	29
13	22	20	18	31
7	24	24	29	26
	15	13	19	32
	10	9	31	27
	27	19	27	26
	27	19	9	25
	13	21	28	23
	18	11	28	26
	26	11	28	26
	27	4	4	20
	9	13	16	19
	30	5	24	
	11	13	30	
	20	5	5	
	24	2	27	
	17	12	12	
	9	9	25	
	18	4	17	
	17	5	32	
	14		21	
	26		7	
	21		29	
	7		9	
	25		5	
	23		6	
	6		0	

Supple. Fig.6E

Gram-positive counts				
**Donor**	**CP**	**IPMN**	**Adjuvant**	**Neoadj.**
7	9	15	12	13
3	15	15	19	19
9	12	20	21	17
11	21	12	15	21
15	20	7	14	18
13	15	17	10	20
10	17	12	14	24
6	18	18	17	20
	15	14	21	23
	1	4	17	24
	23	16	20	22
	20	14	11	17
	10	15	16	16
	17	11	21	17
	19	8	22	15
	20	4	2	11
	11	7	11	16
	20	6	22	
	12	5	22	
	21	5	5	
	15	0	19	
	15	10	10	
	8	5	18	
	13	3	16	
	16	4	24	
	15		18	
	17		3	
	18		19	
	3		5	
	20		4	
	16		6	
	6		0	

Supple. Fig. 6F

bundance Bacteroides				
Donor	CP	IPMN	Adjuvant	Neoadj.
1,1036	1,7057	4,4298	2,0086	1,6773
1,1080	3,3347	2,6394	2,3925	2,4256
1,4016	1,4958	2,5343	1,8995	2,9888
0,6259	2,6931	0,7117	2,9669	2,3849
0,0000	2,3499	1,0991	3,2104	2,8413
0,5999	1,2817	2,1763	2,3387	1,5867
1,4725	1,5059	2,5225	2,8414	1,9241
2,6737	3,0991	0,4800	2,2843	3,0403
	1,7134	1,2225	2,4699	1,2729
	5,3971	3,0124	2,6664	1,9788
	2,4831	2,9440	0,5080	2,9780
	3,1533	2,2547	3,0865	1,4594
	1,2691	2,1512	2,5002	1,0043
	3,0206	2,0552	2,7321	2,3746
	2,0381	4,0030	2,9211	
	1,7464	3,0472	0,0594	
	1,3998	1,0895	0,8326	
	2,8548	0,1664	2,0943	
	4,0666	1,5373	2,2373	
	1,8609	0,0000	1,9487	
	2,7699	0,0000	1,9782	
	2,0943	0,9695	2,1120	
	1,3098	0,6356	1,8009	
	1,7750	1,6927	0,0637	
	1,3109	0,5421	1,6314	
	2,9831		1,0426	
	2,3742		0,6687	
	2,8988		2,1654	
	4,6557		2,0308	
	2,8266		1,6211	
	1,8475		0,9946	
	0,0000		1,7933	

Supple. Fig. 6G

Relative abundance Microvirga				
Donor	CP	IPMN	Adjuvant	Neoadj.
0,0000	0,6728	0,8078	2,6138	1,8209
0,0000	1,1602	0,3730	0,7530	0,3693
0,4701	0,3182	0,6047	1,4227	0,1091
0,0000	0,7667	0,0000	0,9260	0,5026
0,0000	0,9290	0,5108	1,8558	0,9491
0,0000	0,5765	0,4822	0,6630	0,9897
0,0000	1,9293	0,0000	1,0103	1,2236
0,0000	1,5162	0,4472	1,4129	1,4665
	0,8004	0,0262	0,0376	0,3509
	0,0000	0,2538	1,4810	0,9491
	1,0582	0,4249	1,6514	0,7198
	0,7284	0,7049	0,6294	1,5079
	2,6601	1,0475	1,0074	1,7146
	1,5309	0,0000	1,2637	1,0324
	1,0566	0,0000	1,8091	
	0,2721	0,0000	1,6585	
	2,9771	0,0000	0,0000	
	0,4216	0,0000	2,0039	
	1,7265	0,0000	1,2842	
	0,5585	0,0000	1,5823	
	0,3242	2,8918	0,7479	
	0,5395	0,0000	0,3017	
	0,0000	0,0000	0,8871	
	1,0556	0,0000	2,4499	
	0,4924	0,0000	0,5742	
	0,3268		1,2850	
	0,7843		1,2702	
	0,6001		1,2630	
	0,0000		1,7376	
	1,1771		0,9354	
	0,0000		1,2597	
	0,0000		0,6857	

**Supple. Fig. 7A GSE15471**						
Controls	PDAC	Controls	PDAC	Controls	PDAC	Controls
ATG10	**ATG10**	ATG12	**ATG12**	ATG13	**ATG13**	ATG14
4,8720436	4,62999725	7,78047936	7,63393325	7,28561144	7,46293043	7,54997524
5,05468383	4,76454689	7,81733406	7,65378855	7,30958695	7,37712019	7,61066164
5,31881609	5,09862968	7,12838458	7,91824099	7,56943249	7,51880954	6,485219
5,20708083	4,96323597	7,37454999	7,7142657	7,74913125	7,5758591	6,48524266
4,98801326	4,85476792	7,33737931	7,61903374	7,73132114	7,38042829	6,25805823
4,94711651	4,82538256	7,07731082	7,5835203	7,77148572	7,31517138	6,35372764
5,1371984	5,30555353	7,4662076	7,81290797	7,62540562	7,56532632	6,43824629
4,72395497	4,74355518	7,69103498	7,35918443	7,37947373	7,50230241	7,74770844
5,193966	5,06538954	7,38044416	7,6273486	7,70135535	7,49109498	6,47468991
4,77918511	4,71267311	7,37005804	7,60544265	7,50604073	7,29457313	7,22732184
4,61861121	4,75754581	7,45823595	7,7122595	7,44867213	7,44363469	7,42040271
4,98624119	4,88462405	7,5876658	7,41357082	7,68292787	7,49540651	6,59954453
4,82384557	4,98192989	7,35305115	7,51782901	7,54056065	7,54555948	7,3712506
4,73042207	5,11797814	7,14501213	7,25044983	7,78859252	7,37651356	7,07379623
4,96380498	4,75196812	7,20646892	7,68103699	7,49476706	7,62552773	6,65686941
4,89796446	4,90240676	7,47550969	7,39014218	7,55683604	7,44052391	7,56256829
5,18942793	5,28101778	7,0900013	7,47239717	7,61254806	7,55819309	6,73326868
5,02376203	4,87612789	7,13846258	7,47882675	7,6195671	7,38316458	6,34446417
5,34455811	5,30444267	7,03080448	7,60981135	7,72148836	7,26538037	6,48232109
5,09851994	5,05061646	6,79393465	7,55135927	7,69812262	7,42410608	6,4563012
5,01118509	4,74008655	7,35302832	7,53142991	7,46414607	7,45603231	7,36110561
5,03832541	5,05985534	7,35761369	7,3812703	7,26419336	7,50784221	7,10383891
5,35573133	5,03887351	7,27512369	8,16054128	7,76570766	7,64358326	6,84266444
4,8856379	4,87693333	7,36173961	7,52439555	7,56916893	7,52252309	6,95303553
5,06958777	4,59255182	7,64368151	8,08763505	7,54513805	7,76678894	6,89652206
4,99882441	4,85668072	6,93395565	7,50438059	7,83561007	7,55368719	6,60558793
5,1441794	4,80791194	7,48084327	7,66039953	7,66353046	7,54604989	7,06252785
5,00037324	4,77477496	7,13692515	7,62217148	7,78101689	7,58523371	7,36876458
4,60132408	4,98641903	7,70828465	7,4987761	7,39360031	7,25161311	8,05061514
5,11513306	4,59436588	7,58413971	7,55341969	7,44548367	7,47645065	7,35758038
4,89203577	4,85603156	7,55374583	7,40145281	7,62216969	7,56819279	6,81439035
4,84588527	4,85511348	7,55442895	7,59224535	7,60258804	7,53516158	7,27205365
5,11701018	5,00485536	7,21513433	7,31532753	7,59478411	7,44967792	6,30920626
5,0541881	4,85671901	7,16458412	7,32817244	7,56016425	7,54510428	6,46455377
5,15384988	4,53833144	7,02687905	7,68379653	7,844685	7,54814451	6,59810114
4,9709608	4,89211234	5,43834075	7,41801771	7,80502601	7,55905671	5,65883665
5,18107193	4,91134996	6,9905018	7,49486365	7,56622113	7,44339883	6,44106519
4,80256532	4,67241836	7,19702217	7,74751325	7,66440568	7,41781691	6,59825477
4,59287656	4,62016959	7,78362611	7,37533496	7,23602376	7,41113337	7,87035958

PDAC	Controls	PDAC	Controls	PDAC	Controls	PDAC
**ATG14**	ATG16L1	**ATG16L1**	ATG3	**ATG3**	ATG4A	**ATG4A**
7,22719412	6,53465739	6,76936883	7,22134188	6,82578038	7,22695105	7,14530301
7,41852603	6,56073597	6,90320397	7,12972808	6,87390613	7,25403583	6,98739835
7,33419951	6,15313218	6,53125172	6,30491956	6,46980649	7,83409522	7,08428524
7,3374458	5,79720588	6,55183882	6,39383192	6,47335622	8,02449324	7,00280241
6,88891707	5,62654344	6,58919136	6,33336971	6,43746171	7,94523912	7,06059103
7,18635574	5,97957363	6,7405354	6,43064428	6,50578929	8,05259743	7,10102198
7,7104459	5,63472562	6,23610751	6,43842121	6,6196289	7,8610677	7,52841972
7,82274418	7,06882162	6,5948712	6,75211414	6,27469794	6,95214459	7,13706957
6,72342496	5,97214338	6,06683282	6,30091724	6,39501896	7,77200113	7,22966938
7,36059774	6,62647777	6,922447	6,53052214	6,59275328	7,26151646	7,03734771
7,64792031	6,21357472	6,62037895	6,35928532	6,5299635	7,307882	6,81397906
7,34756293	6,6683243	6,64039481	6,89008085	6,47044493	8,0595148	7,42694229
7,24809011	6,7106663	6,30114821	6,60078145	6,39313174	7,71983273	7,13491256
7,5607574	6,13837142	6,36027323	6,36263345	6,22572333	8,0939615	7,1077802
7,09141375	6,17709208	6,44549363	6,23806864	6,39180052	7,3666695	7,040793
8,19776799	6,71410398	6,76968677	6,62724444	6,45886875	7,10044042	7,22984769
7,54440868	5,74131567	6,10640865	6,38131642	6,27378401	7,86288023	7,20653384
7,02895934	5,82613499	5,94511943	6,37675876	6,44388285	7,31253156	6,88457625
7,75659681	5,79801711	6,3257594	6,29134176	6,51058825	8,00484921	7,12129599
7,4580155	5,73469848	5,9012549	6,28737333	6,33541452	7,73532891	6,992381
7,81187055	5,80150915	6,4962322	6,41544457	6,27780757	7,14897098	6,73341255
7,41655913	6,36478926	6,40872463	6,40038116	6,51403073	7,4120301	7,15051143
8,28989974	5,66879133	6,72838138	6,5255351	7,22752612	8,02934441	7,26472142
7,64080773	5,7918183	6,63945229	6,23347673	6,69847357	7,4016113	6,92639805
7,61121403	5,92550372	6,72317764	6,21297723	6,39757937	7,83979786	7,31830785
6,93749957	6,03760736	6,22233226	6,16158076	6,27405807	7,51859691	7,19477212
7,82270353	5,6233561	6,80845608	6,49462383	6,54622572	7,81428828	7,03714681
7,62957459	5,93398324	6,62974628	6,43758239	6,54572643	7,78104384	7,10870487
7,46110304	7,14412353	7,05652663	6,71317068	6,60267624	7,11599264	6,90560391
7,42087909	6,29935456	6,49604858	6,60313092	6,59101235	7,08327857	6,90016409
6,90117185	6,37790824	5,9730427	6,54136431	6,40421924	7,80490643	7,53826465
7,42774438	6,4830399	6,54482431	6,5179741	6,57308859	8,16604962	7,00631642
7,30742503	5,89741007	6,51534934	6,35609324	6,59876	7,52996344	7,14503576
7,18809824	5,54780017	5,92681775	6,40849071	6,42397535	7,34803578	7,05036265
7,2607737	5,91127812	6,38990721	6,35945997	6,48431114	7,82114562	7,15941279
7,74495891	5,9763332	6,26384588	4,93057291	6,40836818	6,98379202	7,22573315
7,49830903	5,66824654	6,87538851	6,36550816	6,57142206	7,75717432	7,02593772
7,62550167	5,51865697	6,80931577	6,33730677	6,61573702	7,67632932	7,06780123
7,24533953	6,9383022	6,4316053	6,60509244	6,44452374	7,0306213	7,16966237

Controls	PDAC	Controls	PDAC	Controls	PDAC	Controls
ATG4B	**ATG4B**	ATG5	**ATG5**	ATG7	**ATG7**	ATG9A
7,36616126	7,4330304	7,42847283	7,41054693	6,35039115	6,57952645	6,93995683
7,16177142	7,50617617	7,48552383	7,57209195	6,57545036	6,89426033	6,83346413
7,46353626	7,32731521	6,94874867	7,76876059	5,50334693	6,11820178	7,51879154
7,22093998	7,31495264	6,84347414	7,54637907	5,52073556	6,15625995	7,65913622
7,13258161	7,29055255	6,95548869	7,51486551	5,25646485	6,06833307	6,85033339
6,9875842	7,42170386	6,92852087	7,50311501	5,52609905	6,00637813	7,04777516
7,06114104	7,56989395	7,05737966	7,39812483	5,45363407	6,41975659	7,64919206
7,1918785	7,34556972	7,18438956	7,26659656	6,12683259	6,01855518	6,76583594
7,52928662	7,43818612	6,64036379	7,02185649	5,55416412	5,90766765	7,26428392
7,27007759	7,24800869	7,17679852	7,28429692	6,47762434	6,33886375	6,84512218
7,44278944	7,32934826	7,3592237	7,30587598	5,65625017	6,27239349	7,09554286
7,53822596	7,33256294	7,91253587	7,64027548	5,89659068	5,97518631	7,17200604
7,62488763	7,44151035	7,81497012	7,00315659	5,84041197	5,89463178	7,25809462
7,38017566	7,381674	7,74196389	6,88176558	5,81651851	6,04643163	6,98130655
7,26995135	7,303364	6,92622184	7,28006965	5,56741733	6,07266633	7,17128723
7,33591597	7,40778487	7,51376264	7,44448138	6,10644562	5,9382042	7,06136403
7,26682289	7,48936111	7,1239348	7,34993291	5,46421945	6,0361776	7,18779066
7,18125981	7,43064556	7,15746487	7,19570609	5,55504136	6,04577457	7,22295748
7,29164168	7,35852096	6,77288333	7,57615134	5,60928224	6,35849214	7,58810407
7,45779787	7,32389406	6,69191693	7,19167796	5,62704261	5,90860489	7,53914006
7,41109164	7,21412074	7,35098786	7,32615659	5,82921106	6,0576633	7,24655866
7,40970101	7,59443038	7,38051848	7,38993955	6,29506069	6,46719773	6,86889333
7,45170688	7,36783635	7,09737627	7,88699557	5,55639185	6,51178333	7,12692458
7,47750261	7,60000588	6,96848821	7,44210364	5,77864114	6,08809061	7,30829634
7,36766496	7,51420156	7,11119759	7,85739478	5,33497361	6,39089047	7,25959398
7,19088755	7,48270645	6,79935579	7,37125119	5,31759895	5,66675769	7,27645502
7,35489974	7,48788871	7,27876248	7,3670398	5,66411726	6,18012052	6,71204139
7,4548211	7,4042903	6,9882234	7,2700498	5,47510082	6,07873585	7,2288077
7,29920777	7,3061354	7,40147556	7,74630821	5,98015249	6,12524239	6,81775685
7,39540728	7,6900005	7,31057385	7,68975339	6,30488578	6,15303041	6,96432622
7,73611806	7,2332047	7,7033174	7,13007458	5,57076062	5,47050009	7,31646433
7,60551871	7,51947583	7,93006221	7,58013914	5,92798095	6,2846065	7,54777097
7,21155171	7,39138125	7,1380856	7,32677376	5,58695878	6,14841814	7,28295257
7,37294392	7,44035556	6,78952031	6,96783218	5,66863307	5,64422469	7,32207677
7,45516212	7,41900803	6,83315454	7,1651568	5,61560867	6,27396324	7,59349847
8,075348	7,51879	5,33127632	7,48505525	5,98398746	6,059559	7,87736779
7,41690985	7,38963777	6,8496444	7,15717316	5,46453211	6,13383316	7,78460114
7,53807787	7,41943158	6,87511485	7,28573171	5,70967662	6,09397469	7,37311289
7,33382968	7,31020914	7,3185188	7,00560039	6,07149063	5,86569784	6,64854953

PDAC	Controls	PDAC	Controls	PDAC	Controls	PDAC
**ATG9A**	ATG9B	**ATG9B**	**BECN1**	**BECN1**	**LAMP1**	**LAMP1**
7,08187925	4,11087744	4,35000811	6,78166943	6,56843864	8,93651104	9,28866038
6,75089072	3,97623723	3,97356388	6,73118854	6,73318227	9,15714918	9,51873011
6,88819967	5,15068431	3,9320214	6,45968857	6,74848856	9,43866817	9,4255243
6,85058628	5,03162825	3,9629886	6,72019546	6,66852492	9,33590248	9,26616295
6,88693561	4,77362809	4,29106758	6,81588986	6,75385289	9,42234851	9,32251733
7,11601748	4,73416551	4,36682522	6,78000242	6,69986455	9,4608388	9,30565436
7,55745035	5,05526404	4,41683988	6,68535663	6,58739402	9,11051093	9,15724371
7,11417242	4,03384469	4,08821995	6,61274858	6,45246002	8,81234752	9,24723859
7,0168508	4,64734182	4,32794036	6,7276657	6,68136717	9,23368811	9,11694058
6,68936853	4,15314703	4,23086652	6,50282599	6,7541599	9,15513028	8,96455012
6,93801809	4,25895184	4,00920144	6,81488815	6,53673885	8,69050962	9,04155944
7,03125743	4,26397778	4,09923373	6,8231715	6,72208071	8,6160523	8,81782338
7,08846445	4,07017881	4,19665535	6,97492898	6,53076033	8,87636785	9,09102119
7,0639245	4,39194625	4,44576934	6,879846	6,50200332	8,49957272	9,25643115
7,34326742	4,6067441	4,22108798	6,64518595	6,93898026	9,66676662	9,24616537
7,12411327	3,89895195	4,09539165	6,38845709	6,31427847	8,96843629	9,02658475
6,91164047	4,51548918	3,99837533	6,70584308	6,46308245	9,29361791	9,24966065
7,16347239	4,4349973	4,34621193	6,66492673	6,68574345	9,2256243	9,51660634
6,8585344	4,47185285	3,85013713	6,80275981	6,59166868	9,41827486	8,95195002
7,08522338	4,88081088	4,46363773	6,62749675	6,50319436	9,0806943	8,93140461
6,97172701	4,13425467	3,96551985	6,50220338	6,61265004	9,1595962	9,14406925
7,05450657	3,77399074	3,94202933	6,29686106	6,65489061	9,1337891	9,44024319
7,04961048	4,39746655	4,3353956	6,5657787	6,78112026	9,58323345	8,93659686
7,21280539	4,37014988	4,14476673	6,35983748	6,72830227	9,09406074	8,77153328
6,82967227	4,5490864	3,95775253	6,72479773	6,63691469	9,28881869	9,18289684
7,30380999	4,96185347	4,50654207	6,60358353	6,65658339	9,30413654	9,08274564
6,84316276	4,35736346	4,06358496	6,72708956	6,68664024	9,43841364	9,33746825
7,01185944	4,67686057	4,59114989	6,74220597	6,7246926	9,43074281	9,21380294
6,86564452	4,17166987	4,40424218	6,7121734	6,66900683	8,95026822	9,62515219
7,57128121	3,98203497	4,12294434	6,60456466	6,70469488	9,09832919	9,0753685
7,04711266	4,47727053	4,37730382	6,80747769	6,66775547	8,96519553	9,10475907
7,10289734	4,01644394	4,05534412	6,93939352	6,68804016	9,06233095	9,30345312
7,00050313	4,91884423	4,34381757	6,66973545	6,77329618	9,31499562	9,26744457
6,99134528	4,85512123	4,37740933	6,50060771	6,69804226	9,12069247	9,00058074
7,09939481	4,7117622	4,34764805	6,89820786	6,70562012	9,36750386	8,91018238
7,40241172	5,60687561	4,14695953	5,71519045	6,54232584	8,77986895	9,06285153
7,02014801	4,76411958	4,18071539	6,5028602	6,62567448	9,4227261	9,27793401
7,16322047	5,03719964	4,00175769	6,75215082	6,67031857	9,06193249	9,11994946
7,15064892	4,18356453	4,4949535	6,59005126	6,43669092	8,79077941	8,93841607

Controls LAMP2	PDAC LAMP2	Controls MAP1LC3A	PDAC MAP1LC3A	Controls MAP1LC3B	PDAC MAP1LC3B	Controls SQSTM1
8,65519718	8,94196908	7,28823815	7,06965509	9,22327357	9,59145891	6,96672409
8,64070061	9,07327531	7,58337209	7,46298095	9,02732972	9,48908538	7,17012241
8,67254028	9,02062016	8,05396104	7,35533321	7,93232741	9,47696133	7,43654864
8,86009359	8,95156387	7,87097563	7,33819454	7,82057298	9,3550699	7,34278622
8,70195623	8,77505851	7,77755493	7,44026796	8,05362439	9,275805	7,34393144
8,61978283	8,82774722	7,92562742	7,50248267	7,93887973	9,16275275	7,34570113
8,55920199	9,16572154	7,81214257	7,68796016	7,73008838	9,00807049	7,10815604
8,02445633	8,7972054	7,73383833	7,87535366	9,187367	9,66279761	6,97906171
8,64472928	8,92306685	7,85291502	7,729245	8,34780871	9,03316565	7,41557072
8,7952307	8,55384344	7,49737193	7,72926852	9,59285523	9,25098339	7,47271344
8,44544692	8,68482605	7,93080567	7,7519121	9,11823025	9,26152466	7,23567763
8,71798402	8,82558524	7,30539011	7,59474935	8,92767538	9,26119511	7,05009132
9,02204587	9,2321274	7,7114844	7,51925944	8,87563048	9,36289618	7,07375288
8,88091581	8,81962101	6,89082002	7,77143065	8,98450303	9,17864476	7,27127304
8,49306382	8,81024046	7,54970453	7,38781207	8,53074756	8,93501792	7,56274363
8,65592755	8,63940211	7,65567703	7,821326	9,22191143	9,26238124	6,94625926
8,87970786	9,29507413	7,98796326	7,59662288	8,34869626	9,16384234	7,01280889
8,78345484	9,15782117	7,8796097	7,42043947	8,30067363	8,86856883	7,25108665
8,47043396	9,38911005	7,64012257	7,57648663	8,20307897	9,27282854	7,55246675
8,69955321	9,17975149	8,05807273	7,69625859	8,01861019	8,9636475	7,18273541
8,87137566	8,74256774	8,26769316	7,96626209	8,87084202	8,91135257	7,12928305
8,82586906	9,22178727	7,83050022	7,71920477	9,34216136	9,13105748	7,47520565
8,80631092	9,73744555	7,93950255	7,49658065	8,09387807	9,31100063	7,23838464
8,82434514	8,97870269	7,70557904	7,31259385	8,47733799	9,00811452	7,18206842
8,67773253	9,07349439	7,85268003	7,82744666	8,04870469	9,45860844	7,24154734
8,63947728	8,92721263	8,0630473	7,49478016	7,9841184	8,97783735	7,55541518
8,99147165	8,54291988	7,78424993	7,66669555	8,43091829	9,40822377	7,37311514
8,89864032	9,00642646	7,68764298	7,39226029	8,17027486	9,16480915	7,14616415
8,29430371	9,02892296	7,61750578	7,49732381	9,17500437	9,40580886	7,16503432
9,38727098	8,86559483	7,22773245	7,76814278	9,2259831	8,75272281	7,23114847
8,61183559	8,74408301	7,90124772	7,60008406	8,53524991	8,62853113	7,20390542
8,60494543	8,60778396	7,72679256	7,41335299	8,56432224	9,0364364	7,06757001
8,72450108	9,27230889	7,99992594	7,42265962	8,10908374	9,06967756	7,37091571
8,71017575	9,00004579	8,20618756	7,57862514	7,95850806	8,41966814	7,38399996
8,67440304	9,08397908	8,04196182	7,52036474	7,78600883	9,17749489	7,24259725
5,77300101	8,77153331	8,82611381	7,81397592	6,07561374	8,91613511	7,23615061
8,4052096	8,57599111	7,70434499	7,43874951	8,04480779	9,21027583	7,52588314
8,48821779	8,89312937	7,84251115	7,62783005	7,94170694	9,24267476	7,23400711
8,20306321	8,71507038	7,54982814	7,48709926	8,98654652	8,667617	7,05083407

PDAC
**SQSTM1**
7,19535787
7,34620423
7,3371226
7,35812218
7,34438708
7,20711126
7,61609662
7,13459546
7,3057546
7,32990202
7,36675156
7,3250279
7,39261973
7,30245666
7,98141327
7,07341116
7,14462247
7,44340562
7,31814794
7,18974716
7,20064679
7,63490533
7,69737977
7,43188987
7,52738431
7,30324289
7,22854883
7,39364895
7,18487579
7,44096578
7,28176302
7,76947972
7,28261573
7,21746221
7,41894827
7,14366709
7,34746743
7,05409615
7,16236454

Supple. Fig. 7A GSE56560

Controls	PDAC	Controls	PDAC	Controls	PDAC	Controls
ATG10	**ATG10**	ATG12	**ATG12**	ATG13	**ATG13**	ATG14
4,45577988	4,89154124	5,06875246	5,46182715	5,73726085	6,11713086	4,78932553
4,6010058	4,94188228	5,09805224	5,07955938	6,06511865	5,95108869	5,10512097
4,48331466	4,86777164	4,9117514	5,38220895	5,83335099	5,892775	4,78157037
4,45566649	4,3568839	4,89121735	5,445713	5,53201936	6,18248931	4,57692585
4,38486541	4,81184569	5,0188776	5,27955122	5,52844077	6,12645345	4,29835793
5,00650946	4,76253669	4,9098018	4,90917274	5,76146956	5,9056693	4,54764146
	5,39712816		5,44838915		6,11488171	
	4,38144138		4,99677699		5,97388951	
	4,76591979		4,9878218		6,10724886	
	4,4695479		4,81054027		5,80087588	
	4,76642256		5,60986889		6,13422088	
	4,8342531		5,01351313		6,07973862	
	5,06729209		5,1515603		6,13597078	
	4,57880563		5,25721177		6,35769871	
	4,4626337		5,0932898		6,09057166	
	4,95634776		5,24002864		6,1405078	
	4,60691168		5,17145151		6,05382064	
	4,9841889		4,98031618		6,01222914	
	4,51340532		5,60466082		6,0213872	
	4,73971423		5,22832757		5,76216625	
	4,38208309		5,24337241		5,75679403	
	4,66947663		4,97364523		5,94275276	
	4,75071933		4,9381276		6,02629512	
	4,58048061		5,37650627		6,20820925	
	4,24347494		5,16675112		5,63410883	
	5,00124061		5,24403749		5,81516495	
	4,83886264		5,21472278		5,8539206	
	4,52511691		4,9974838		5,95428815	

PDAC	Controls	PDAC	Controls	PDAC	Controls	PDAC
**ATG14**	ATG16L1	**ATG16L1**	ATG3	**ATG3**	ATG4A	**ATG4A**
4,77241971	4,77517998	5,69677966	5,2996552	5,83247145	4,81758056	5,04826636
5,10730157	4,93147873	5,37618406	5,69665447	5,75800895	5,09113022	4,89517405
5,4058677	4,79750581	5,45716091	5,31766789	6,01067273	4,64831482	5,24250248
5,32067564	4,87754128	5,36147533	5,39515316	5,98655981	4,8790137	5,34750759
5,29736599	5,14733815	5,60195102	5,20447222	6,05570345	4,51122909	5,20148069
5,05344408	4,75824197	5,42583873	5,20559368	5,70250786	5,02593623	4,9547048
5,02883456		5,26021727		5,9311389		5,26482114
4,96692873		5,64565403		5,43034425		4,76550666
5,10869493		5,49494191		5,72811963		4,86565574
4,93579136		5,46251778		5,42142063		4,64966254
5,40298299		5,54193895		5,91032157		5,27878209
5,03218607		5,26673782		5,79665355		5,05728588
5,17063553		5,30912854		5,74486479		5,21476757
5,13111425		5,10656562		5,68400215		5,28079606
5,20301488		4,91276035		5,33911326		4,8459499
5,15402181		5,2233485		5,83343534		5,09900831
5,00181949		4,88358363		5,52758677		4,99922009
5,30740339		5,39571589		5,82293267		4,96151773
5,26291738		5,57045823		5,76634905		4,85981276
4,94927163		5,06014629		5,85049827		5,04770259
5,09081011		5,54870862		5,63927575		4,62564973
4,96608129		5,46826537		5,69243271		4,81131323
4,95029563		5,63790604		5,87878027		4,90263968
4,80866586		5,76381993		5,82753777		4,98858425
4,91400505		5,16838136		5,80024121		4,99869344
4,89954528		5,43504138		5,76607247		4,74593645
5,04924067		5,1474117		5,86652313		5,03145944
5,50262969		5,51789267		5,61474312		4,91483071

Controls	PDAC	Controls	PDAC	Controls	PDAC	Controls
ATG4B	**ATG4B**	ATG5	**ATG5**	ATG7	**ATG7**	ATG9A
5,77020324	5,54379563	4,9986072	5,89581515	5,0434473	5,7608282	5,74349299
5,70605447	5,71067037	5,21331702	5,51811285	5,35713127	5,68847311	5,59395065
5,86561285	5,43609722	5,21528773	5,63174324	5,493436	5,77641072	5,68787134
5,67133177	5,63245244	5,27836336	5,50390867	4,93744804	5,82493124	5,59515135
5,92715679	5,56945833	5,11598458	5,81586252	5,35586302	5,8749255	5,81376726
5,69344794	5,60235241	5,23298841	5,14723345	5,00971415	5,50859079	5,66542745
	5,3487967		5,70064789		5,67213111	
	5,72970392		5,36265836		5,72834398	
	5,68024427		5,39965302		6,22362397	
	5,52998924		5,64852497		5,7012667	
	5,65871734		5,56252554		5,69279161	
	5,49296832		5,41549075		5,80005793	
	5,57616553		5,56777056		5,84066934	
	5,47778576		5,46454309		5,65061139	
	5,56446019		5,1390899		5,43692069	
	5,49331707		5,26114588		5,71604815	
	5,40522493		5,07059707		5,66279262	
	5,46024607		5,64932051		5,50584078	
	5,44042038		5,33776129		5,52271623	
	5,50045718		5,50969927		5,77326756	
	5,6324921		5,47732259		5,69025851	
	5,44613842		5,20119748		5,61426118	
	5,55566061		5,44030031		5,99223406	
	5,59853283		5,66662456		5,60777109	
	5,67873788		5,48450856		5,54853482	
	5,69871023		5,48789429		5,59733257	
	5,436218		5,51187218		5,54680749	
	5,8392139		5,32462655		5,51224524	

PDAC	Controls	PDAC	Controls	PDAC	Controls	PDAC
**ATG9A**	ATG9B	**ATG9B**	**BECN1**	**BECN1**	**LAMP1**	**LAMP1**
5,63483908	5,20750766	4,70488233	5,10326396	5,44377528	6,91803194	7,03232222
5,582772	4,63939365	4,38619541	5,12846752	5,32282805	6,70965077	6,7484433
5,51036269	4,73740343	4,60959216	5,45706191	5,39690289	7,08743296	6,73047913
5,74626091	5,14125273	4,51081725	5,48180687	5,09952807	7,17791967	6,9884848
5,8472417	5,11620676	4,30283549	5,39361569	5,33759192	6,986507	6,81729253
5,80925059	5,15455234	4,86508006	5,14012373	5,5319162	7,1725768	6,9264138
5,5261196		4,40738092		5,34778269		6,86525175
5,68974108		5,11954051		5,38754503		6,95240148
5,8453164		4,53458731		5,38082096		6,90023581
5,59643034		4,47890905		5,46831449		6,889121
5,65218921		4,61882711		5,58803552		7,0239471
5,69300171		4,3804034		5,45570414		6,9732753
5,73739574		4,10477644		5,52879474		6,88544458
5,77847001		4,28964893		5,26425477		6,8306058
5,64514372		4,79136556		5,4259666		6,93515775
5,72862027		4,38147116		5,30468234		6,80427816
5,59076491		4,62427231		5,17725172		6,98312498
5,64615071		4,10534078		5,66804323		6,88546998
5,6709142		4,79550532		5,22778591		7,07341702
5,63907098		4,51617103		5,32966237		6,82427176
5,74311537		4,89327442		5,63077394		7,10329104
5,59430243		4,53136761		5,4764963		7,14294396
5,61721489		4,89606365		5,11928887		6,89088675
5,91022748		4,8136171		5,31627977		6,65890392
5,76836782		4,92791391		5,29885845		6,97340437
5,92861722		4,71037348		5,20217933		6,70530144
5,58679925		4,54376072		5,50325251		7,07600284
5,81186794		4,58915324		5,2736579		7,05391717

Controls	PDAC	Controls	PDAC	Controls	PDAC	Controls
**LAMP2**	**LAMP2**	**MAP1LC3A**	**MAP1LC3A**	**MAP1LC3B**	**MAP1LC3B**	**SQSTM1**
5,62323602	6,13471195	4,7868157	4,81242971	5,38084956	6,60472457	6,52470894
5,70765335	6,14322924	4,71578007	4,34940938	5,17304073	6,02721815	6,68211013
5,43699887	6,17683816	5,11944946	4,51267541	5,96771575	6,23618742	6,82480242
5,40980731	6,26970807	5,01219952	4,39192771	5,79023507	6,12344008	6,73395941
5,20764014	6,09016019	5,30339281	4,68097683	6,01620881	5,8013294	6,88386836
5,416306	5,69880653	4,60727018	5,03252599	5,68931224	6,21218237	6,69889522
	6,54946797		4,42075183		5,98977282	
	5,59802447		4,7990266		6,18695069	
	5,7797133		4,47580795		5,95985705	
	5,624749		4,58142092		6,04640619	
	5,90103898		4,42170176		6,47885695	
	5,9460469		4,29680209		5,84221518	
	6,21096708		4,44597144		6,2136434	
	6,19882477		4,35496271		6,11693295	
	5,83473078		4,74203015		6,00183939	
	6,17865166		4,45070918		6,09486582	
	5,95837959		4,74077017		5,98558257	
	6,18352683		4,28257487		6,05908854	
	6,13926888		4,62403231		6,48875245	
	5,85034357		4,55571541		6,10021734	
	5,45687331		4,64544906		6,27436332	
	5,83183424		4,72820363		6,13098716	
	5,68694411		4,71188587		6,03141976	
	5,53481755		4,72795763		5,96523032	
	5,74002721		4,77844251		6,23257913	
	5,95834228		4,64008063		5,804179	
	6,35209908		4,72394536		6,19422133	
	5,84342119		4,72837537		6,33402901	

PDAC
**SQSTM1**
7,24920369
6,86600945
6,96304816
6,96080776
6,70173922
6,89592965
6,8259898
6,86841217
7,14372125
6,98658711
7,44525069
6,90864825
7,07875196
6,81929248
6,73614007
6,9426299
6,61167123
6,91819178
6,95405292
6,91050747
6,8840215
6,54454825
6,71209971
6,92640595
7,07273101
6,79002823
6,56863693
7,37792003

Supple. Fig. 7A GSE62165

Controls	PDAC	Controls	PDAC	Controls	PDAC	Controls
ATG10	**ATG10**	ATG12	**ATG12**	ATG16L1	**ATG16L1**	ATG2B
4,34175885	4,53085982	6,13580267	5,98377561	4,86963632	5,33121712	6,57010737
4,20991606	4,59283208	6,13039755	5,33681529	4,69823682	5,4250693	6,45468808
4,59846327	4,19915953	5,83169503	6,02976748	4,45620145	5,03262774	5,85060251
4,16828312	4,47462556	6,44098171	5,77651125	4,87377577	5,04520214	6,88241908
4,42437021	4,43971864	6,47140044	5,94705098	4,86770202	5,13283768	6,39429185
4,39534889	4,27163597	6,4120347	6,28520647	4,63462627	5,22811369	6,65629394
4,31579393	4,45571223	6,40778099	6,15910905	4,87453041	5,07169126	6,58572878
4,03098044	4,34504606	6,31340025	6,07004679	4,68867287	5,05167738	6,58142083
4,35369477	4,59224013	6,13740521	6,20495614	4,89817337	5,1372674	5,66296575
4,49720399	4,26339112	7,22957532	5,89176685	4,81254084	5,02875399	6,66982278
4,30685891	4,74030228	6,59265555	6,19959544	4,60931385	4,81778477	7,0644405
4,43175767	5,89767354	6,91770925	6,56457614	4,77940903	5,88531184	6,49200338
4,33148001	4,78318012	6,34724758	6,06844288	4,7474055	4,89608714	6,905873
	4,610412		6,02432084		5,3616842	
	4,65484858		6,16195712		4,74740203	
	4,51296866		5,88783357		5,50300046	
	4,45182488		6,2483108		5,29720046	
	4,47915884		6,13162433		4,81675507	
	4,48964003		6,07896786		5,08618315	
	4,45469425		5,83658242		5,27008421	
	4,34355161		6,14037682		5,17016351	
	4,37611744		5,94423997		5,64254592	
	4,90572321		5,88342807		5,16363482	
	4,38208882		6,064028		5,20865856	
	4,76115834		5,85554886		5,18124225	
	4,28740573		6,07715015		5,40785931	
	4,3888159		5,93563564		4,84445996	
	4,38939707		5,70923952		5,68000794	
	4,57980352		5,87381272		5,39893131	
	4,55454841		6,16008962		5,05771245	
	4,26270045		6,10968933		5,43098061	
	4,55987206		5,96268068		5,36728793	
	4,51533035		6,28360236		5,04378746	
	4,50204564		6,00188629		5,30670306	
	4,09518348		6,18053903		5,21891447	
	4,28713662		5,87930553		5,10108287	
	4,24196312		6,16617387		5,65180803	
	4,81680808		5,88967923		5,19505668	
	3,91484561		6,08689806		5,45536069	
	4,35560628		5,70316937		5,23682594	
	4,21884432		5,96696994		5,01555796	
	4,17401167		5,70998836		5,1669714	
	4,26081856		6,182725		5,23076693	
	4,2844317		5,9508677		5,32164237	
	4,65807744		5,64216524		4,97411184	
	4,90117041		6,1502356		5,02904226	
	4,34120935		6,14258376		5,57855304	

4,70347058	6,28722111	5,15221396
4,62029274	5,80717714	4,92240162
4,45107734	5,99485986	4,94389595
4,42389684	6,36320098	5,29516519
4,49439924	6,15166606	5,02027516
4,51592512	5,96382555	4,83343697
4,31288097	5,91832317	5,06078317
4,5296123	6,07597551	5,09045602
4,02793816	5,7785598	5,2429078
4,32944574	6,00079634	5,11733349
4,75276095	5,9620649	5,42995802
4,25523929	6,04445251	5,13938199
4,41528879	6,00057755	5,52235397
3,98233854	6,31225982	5,1332633
4,61087753	5,64583873	5,16954941
4,20479315	5,98959143	5,34544505
4,24564702	6,11564146	5,35242915
4,12115051	6,20842747	5,29074222
4,71703342	5,99060799	5,33975874
4,51831254	5,85779703	5,48813214
4,33973584	5,9063498	5,18830703
4,57455077	5,8517317	5,70249652
4,34634196	6,04814865	5,09976277
4,24457726	6,16528686	5,33929518
4,46922842	6,08764932	4,86428105
4,05992364	6,02080812	6,10526392
4,14658063	5,74805092	4,87289547
4,43384702	5,56852134	5,18891872
4,83144963	6,86299494	5,61042961
4,43071699	6,08898119	5,471675
4,37643628	6,42354625	5,32668096
4,37125184	5,95806078	5,72651924
4,5746254	6,01175868	5,37274569
4,39749216	6,43593992	5,98817901
4,57524667	5,91862846	5,13501711
4,50073374	6,19966137	4,86058202
4,39579356	7,17707504	5,51360132
4,43604895	6,38580029	5,38028453
4,04689231	5,7918632	5,16221323
4,2884278	5,89937567	4,78028513
4,05864716	6,11196371	4,93185395
5,21992097	6,3668875	4,9587556
4,13128041	6,00947538	5,27182657
4,64102804	6,3102242	5,13713871
4,6518084	6,06361601	5,02540412
4,18371901	6,47313284	4,5178675
4,34016728	6,13709132	5,12846695
4,41320675	6,10448502	5,06386163
4,72079902	6,10898547	4,74584462
4,17985488	6,20415282	5,19366299

4,42900949	6,48471701	5,53364637
4,6283509	6,56308399	5,50184125
4,45357992	5,99363602	4,90514559
4,27205805	6,22950496	5,05412809
4,26854869	6,22363318	5,38546725
4,50123715	6,04287435	5,22293179
4,2095786	6,29074128	5,36282302
4,40145634	6,1369188	4,92682161
4,29342623	5,63304548	4,98746653
4,12004716	6,04468057	5,41411583
4,56377731	6,04119022	4,96655584
4,22188645	5,89426347	5,59146846
4,20344475	5,96416731	5,41796682
4,60123926	6,03416038	5,22180536
4,96026625	6,01941005	4,94368059
4,44650393	6,21118326	5,29948154
4,39887746	5,99230455	5,33829016
4,83449672	6,07820717	5,42468417
4,15252148	6,24183901	5,4500938
4,52461004	6,72077818	4,85465096
4,23945353	5,88042834	5,46713289

PDAC	Controls	PDAC	Controls	PDAC	Controls	PDAC
**ATG2B**	ATG3	**ATG3**	ATG4A	**ATG4A**	ATG4B	**ATG4B**
5,34437561	8,57863249	8,46600948	7,9564133	7,21843373	5,60318893	6,17854656
5,64813532	8,51485384	8,323169	7,63622774	7,05522586	6,14716791	5,3511324
5,4526634	8,76698946	8,69663244	8,36360836	6,87496636	5,80233226	5,71876082
5,50720768	8,65288647	8,48834676	7,86799464	7,0221224	6,17018654	5,85564347
5,9680664	8,76984275	8,47522294	8,56525268	7,13952137	5,58957163	5,61843254
5,78332431	8,65366463	8,69381692	8,22900124	7,13510027	5,64415285	6,36926413
5,5158089	8,72227001	8,6661208	7,83485441	7,22406078	6,06455075	5,74730501
5,52138971	8,49919733	8,56085924	7,57303026	7,17669285	6,18345571	5,697759
5,37033824	8,54691719	9,1304731	7,31122741	7,29299542	6,36445018	5,80790692
5,84144815	8,81670426	8,46596602	8,51610494	6,96173427	5,60324688	6,12151887
5,71069601	8,57571423	8,46217524	8,07891483	7,30899128	6,08554304	5,98895827
5,05467664	8,74968545	8,77785094	8,27383083	7,19789753	5,31022426	6,06574674
5,81363121	8,53200595	8,69441861	7,69742144	6,88505789	6,18868848	6,08018909
5,49013194		8,57597074		7,08648077		6,13137053
5,7167456		8,66415393		7,47968271		5,66571432
5,41016415		8,45599719		7,38020391		6,31100185
5,97401729		8,36159234		6,95650925		5,87156596
5,70550245		8,61815535		7,20801994		5,73869546
5,62148382		8,66856496		7,1122035		5,85673467
5,75406108		8,46585411		6,98585801		5,73637464
5,31402992		8,8296318		7,25984898		6,00174919
5,6584009		8,73579149		7,08784309		6,40045373
5,48255657		8,56493294		7,51607349		6,02202477
5,99953307		8,4551866		7,13216602		6,07544791
5,54854227		9,12999965		7,24618553		5,82875615
6,05160554		8,63844673		7,02565091		6,15269394
5,56858946		8,72844321		7,50010175		5,76516663
5,44891698		8,78254747		6,43537483		5,83102812
5,09150784		8,45839471		7,20799509		5,77826362
5,79400828		8,60235751		7,21611206		6,02124821
5,84583954		8,59833255		7,19657665		5,96773738
5,50049366		8,88538922		7,20000919		5,93449195
5,96674639		8,80270932		7,08969687		5,70329912
6,15018098		8,40474141		7,06292785		6,32630145
5,41644804		8,75195629		7,76517555		5,60273584
5,47306996		8,62814275		7,03388226		6,02661135
5,85352369		8,16394024		7,10431927		5,88645117
5,59882341		8,93263582		7,3739911		5,92035482
5,43855845		8,68143309		7,50051013		5,78178581
5,74141502		8,51725634		7,53613797		5,8668472
6,33517869		8,56368663		7,3440683		6,02605523
5,6978549		8,65250269		7,21950194		6,19822585
5,20624441		9,09993734		7,51686469		6,12568504
6,0078499		8,61039589		6,93320666		6,05279633
5,22724303		8,71362751		7,21447174		5,98314064
5,13617574		9,07554103		6,9739124		6,25575979
5,77685211		8,63463324		7,20268323		6,61501584

5,70450785	8,73081646	7,14215172	5,62850136
5,72350504	8,51612912	7,16322916	5,7015136
5,53799654	8,86201531	7,44441725	5,71961253
5,97661743	8,33532051	6,94406459	6,46091342
5,47483488	8,62089578	7,08664229	5,82999916
5,58570719	8,6665289	7,22810238	5,67777575
5,94087292	8,73653402	7,64857481	6,05429676
5,82035096	8,42166351	7,16330339	6,50210691
5,33421232	8,60032879	7,35596253	5,94937072
5,50933857	8,59693788	7,29094815	6,11115641
5,46645081	8,42939761	7,21920145	6,01953038
6,01695404	8,42629176	7,15581329	6,94040958
5,67221414	8,54549877	7,00738858	5,67787734
5,33004952	9,05365366	7,11520155	5,7762825
6,19667168	8,36162698	6,97953306	6,06252725
5,62950506	8,71639547	7,32654836	5,93330902
5,53112731	8,68941447	6,89562905	5,71808358
5,82926061	8,44633397	6,87547375	6,4697342
5,29168028	9,01263459	7,48969325	5,92498712
5,57800608	8,65385762	7,23801326	5,46397835
5,90823594	8,46550659	7,07565631	6,29167671
5,35680626	8,67734895	8,04835608	6,24643348
6,20509999	8,58279642	7,14189162	6,06257012
5,62085391	8,86112865	7,26175056	5,94817295
6,13978359	8,4905184	7,43713018	5,59609455
5,47837989	8,87684205	7,25340834	6,60217635
5,38444292	8,68266611	7,16313604	5,66299364
5,97031548	8,16223543	7,34284135	6,37076079
5,2769664	8,90578345	7,30641491	6,1023663
6,19397361	8,60085612	6,97719994	6,53509021
5,68285022	8,55043934	7,15014437	6,09065737
5,86558646	8,40003699	7,04958867	6,62862215
5,34272477	8,71143216	7,0181882	5,78385433
5,64539583	8,38592547	6,53976659	6,23689783
5,85581643	8,73774473	7,04165277	6,47379319
6,02673801	8,58810344	7,89360056	5,75474471
5,74375952	8,04426237	6,71493472	6,35233177
5,38631444	8,31540323	7,24853198	5,87644777
5,91682297	8,42084134	7,26481258	6,43616432
5,82868572	8,65476953	7,62356908	5,74621046
6,08522975	7,95371878	6,78643418	6,09783177
5,54096639	9,00570393	7,39029264	5,73216774
5,82804152	8,51068747	7,04431692	5,92042355
5,98730684	8,18567808	6,88570624	6,35502508
5,47831756	8,61680974	6,9597219	5,88470652
5,9060534	8,66507659	7,88040085	5,65486566
6,18409791	8,36246389	6,65631333	6,26962122
5,42851757	8,82896833	6,88674603	5,91278484
5,96285528	8,50865637	7,63255811	5,74774335
6,04836492	8,58887419	7,29190107	6,3078832

5,28966213	8,88868577	6,94912776	6,12005544
5,3505262	8,90172031	7,67785773	5,8698847
5,77929545	8,56184507	7,1496008	5,82059982
5,84613673	8,5659062	7,32631082	5,91848631
5,87159082	8,29146024	7,25343757	6,10395212
5,70773203	8,50358482	7,39913589	5,8720483
5,92423256	8,40493576	7,24739696	6,19944274
5,60674869	8,60758495	7,28572363	5,92619355
5,66840487	8,61908564	7,15805546	5,87704457
5,96107393	8,28821652	7,30727388	6,21814993
5,43103125	8,84652392	7,19735673	5,7200958
5,73609876	8,80784918	7,67158319	5,87136438
6,13003246	8,4120611	6,88691677	6,24365868
5,77715762	8,65397753	7,19894272	5,97090804
5,77439435	8,79328728	7,12203025	5,70766528
5,85242003	8,8854289	7,07385346	6,24773428
5,28260606	8,5867165	6,4678484	5,69779574
5,51103483	9,03622514	7,32263126	5,94594596
5,52525734	8,51854713	6,85597442	5,95439808
6,98284142	8,63384096	7,53480598	6,04878272
5,47792068	8,66906527	7,11761512	6,09945624

Controls	PDAC	Controls	PDAC	Controls	PDAC	Controls
ATG5	**ATG5**	ATG7	**ATG7**	ATG9A	**ATG9A**	ATG9B
7,98462123	8,38893955	4,75903598	5,2958097	5,71657376	5,33524284	4,31193851
7,9120078	7,87522374	4,28243033	5,14939619	5,54426788	5,31703787	3,89862514
8,23145844	7,8567257	5,03685558	5,23041643	5,42740122	5,10281107	4,09532453
7,34728687	7,64082525	4,91374311	5,42850604	5,84858481	5,38374272	4,22633819
7,76112024	7,79663199	4,71198077	4,90999644	6,41859876	5,15881887	4,82514334
8,07132472	7,95363795	4,18460356	5,34376095	5,66745523	5,34420289	4,33183702
7,64011902	7,89199781	4,71233706	5,05690024	6,00257783	5,27701408	4,5013378
7,71066958	8,3795698	4,91610798	5,33616129	5,64087832	5,33370433	4,43774402
7,53561442	7,91270503	5,38583614	5,00487511	5,69635756	5,30732129	4,23484831
7,96206747	7,61439955	4,57057333	5,10100453	5,87753965	5,19768677	4,52537062
7,58012718	7,86942713	4,76729709	5,07992994	5,88314895	5,35284937	4,5071753
8,04607976	8,3298059	4,42644183	5,16712869	5,64661632	5,32511294	4,42274453
7,56794617	8,08662039	4,82894134	5,41672008	5,84364784	5,10477589	4,29634259
	8,29546093		5,24702458		5,17515034	
	8,16653904		5,08682791		5,49155638	
	7,79804945		5,39116407		5,76501168	
	7,8162602		5,47256916		5,4031769	
	8,18593425		5,09116717		5,23989818	
	7,94633107		5,05773693		5,19755077	
	8,11311219		5,09809309		5,38941399	
	7,89218388		5,1977242		5,13435883	
	8,09444106		4,94807193		5,45123065	
	8,32121436		5,11134275		5,36218828	
	7,68748578		5,38584491		5,36502285	
	7,95448755		5,08872152		5,20309102	
	8,04062375		5,5704622		5,24489653	
	7,88866643		5,60508662		5,41412921	
	8,10077488		5,24745674		5,1508426	
	7,99780565		5,16781287		5,60540088	
	7,7567327		5,83758394		5,06616367	
	8,10119804		5,03898894		5,40436522	
	8,15534346		5,27202668		5,22552788	
	8,93295061		4,98964902		5,70572078	
	7,71203927		5,23531867		5,25136104	
	7,92741986		4,72952504		5,2138946	
	7,94492847		5,22094227		5,15243819	
	7,73567306		4,59274624		5,15491817	
	8,29879135		5,17245029		5,25705047	
	7,9716395		4,89854917		5,44111955	
	8,23355624		5,13111887		5,31544333	
	7,7531503		5,0328705		5,40794384	
	8,54102571		5,62622977		5,48746512	
	7,83382069		5,42625611		5,43378671	
	7,57904396		5,11423154		5,21103612	
	8,45368185		5,3101166		5,33170123	
	7,98736852		4,77734037		5,48733428	
	8,1904022		5,58019892		5,4258723	

8,41429583	5,19909513	5,22555133
8,22502789	5,68352003	5,40837061
8,22651057	5,17002535	5,29439448
7,80803316	5,5568729	5,15949578
8,42650297	5,286525	5,43595738
8,15368739	5,63582809	5,06875143
8,22087688	5,3632582	5,22914296
7,95061342	5,24049305	5,41028613
7,87527584	5,10380053	5,40379789
8,29866835	5,39709802	5,50970892
8,17965413	5,01800372	5,47830869
8,17801789	5,52817892	5,29252858
7,89723601	5,38307311	5,13803924
8,52944192	4,95556812	5,39258815
7,82316615	5,52169564	5,23420893
8,11822535	5,24990588	5,15459083
8,57375521	5,59981399	5,35345427
7,97382555	5,21861119	5,41445751
8,06880956	5,09659059	5,49711442
8,52983795	5,39061736	5,16092452
7,7131726	5,27214253	5,35960504
8,81217533	5,26430308	5,49593359
7,76721973	5,62566804	5,41139664
8,22989556	5,42260838	5,15040442
8,288254	5,15656051	5,35847353
7,64162714	4,85200915	5,47755399
8,13396171	5,47597692	5,30145206
7,65313315	4,90377776	5,76264901
8,25734405	7,2742778	5,3645759
8,09791054	4,82254801	5,13644085
7,95915219	5,2698859	5,52835424
7,69769512	5,64010448	5,21234254
7,90918041	5,46385774	5,43523733
8,01715035	5,50787725	5,41528049
8,38604274	5,14724403	5,31375863
8,15039397	5,14210502	5,39084624
7,19415199	5,62823938	5,21702585
8,51686394	5,31743674	5,30908022
7,54261063	5,44066539	5,30880773
8,12636607	5,1919119	5,26631986
8,03267728	4,99358962	5,25929897
8,21198144	5,51480104	5,16464817
8,51857394	5,29604039	5,24799428
7,90915702	5,64708499	5,39963499
8,24223533	5,38031329	5,49031483
8,12322956	4,96815157	5,56301919
7,96158957	5,74451818	5,27494496
8,16642705	5,44113426	5,08470323
8,18434979	4,80567799	5,4064202
7,75964518	5,57393054	5,30930962

8,18449242	5,31022068	5,19719231
8,56879473	5,34805357	5,58311932
8,12292069	5,46194086	5,37510274
8,52284743	5,3894882	5,33362159
7,85757751	4,8823855	5,13945174
8,32955705	4,86831032	5,22349512
7,7361756	4,98273466	5,19679078
8,11372	5,44539131	5,33160825
8,10123977	5,40446046	5,39914959
7,92031445	4,92134575	5,10209177
8,14951649	5,5046363	5,03686921
8,13783171	4,7152994	5,23615183
7,66716915	5,21491639	5,46592183
7,86030695	5,48733346	5,27938082
8,12997385	5,67412743	5,12795774
8,02298111	5,97736205	5,39371657
8,52475695	5,32670467	5,21931972
8,08200635	4,86074784	5,30386564
8,42819719	5,49060821	5,35835772
7,78896761	4,91379031	5,40870133
8,35277125	5,29100637	5,4931287

PDAC	Controls	PDAC	Controls	PDAC	Controls	PDAC
**ATG9B**	**BECN1**	**BECN1**	**LAMP1**	**LAMP1**	**LAMP2**	**LAMP2**
3,76451591	8,86900221	8,82075097	9,62779933	10,2288021	7,16836947	6,86315106
4,08234642	8,98946268	8,95495512	9,78254807	9,84754235	7,37787971	6,87223801
3,64682367	8,9221939	8,75122459	9,71855967	9,8855891	7,18676038	7,30208543
3,78188211	8,96133942	8,650662	9,89296802	10,411475	7,34459943	6,96343608
3,60777693	9,00120356	8,83459635	10,2676018	10,3786838	6,92275776	8,14372405
3,80284686	8,93241934	8,93056616	9,92817238	9,80262685	7,085712	7,21862434
3,87975403	9,13061793	8,93306986	9,75068011	10,4030183	7,03019894	7,60264409
3,83271141	8,82762158	8,72709636	9,95725958	10,1787562	7,20082189	7,50474733
3,71995059	9,41339625	9,0349147	10,5861404	9,46045317	6,74022223	7,28625401
3,60448369	9,26848191	8,57631342	10,0910735	9,70581618	6,89078492	7,54624268
3,94395064	9,06939244	8,8267817	10,1139208	10,1468222	7,20715894	7,17085557
3,97311806	8,99216406	8,43122565	10,1353722	10,2534595	7,11908099	7,58413021
3,85348962	9,07345543	9,00005606	10,0267633	10,238939	7,33272452	7,41377694
3,69039466		8,86445878		10,1846245		7,56080598
4,26644427		8,99443308		10,5545458		7,47029732
4,0437865		9,7249029		10,7933949		7,23371167
3,7884228		8,79585521		9,98003108		6,91387541
4,08721405		8,74923051		10,4474489		7,44751362
3,8082521		8,81349559		10,2657272		7,46893578
3,85353005		8,81402307		10,2314802		7,36656548
3,67689205		8,48210905		10,00317		7,25160643
3,93476722		8,82749893		9,74874374		6,65286837
3,92534156		8,87324391		10,3577451		7,3697829
3,63460793		8,51849278		10,0058859		6,84333204
3,86579317		8,73129044		9,52496995		6,87600401
3,88041346		8,64094735		9,82289733		7,26951163
3,97326209		8,87320324		10,1550039		7,50539544
3,90105157		8,63408576		10,5932512		6,46487154
3,81823761		8,42653048		9,99834621		7,34259916
3,75967228		8,66895405		9,9343551		7,33841855
3,78196616		8,6121947		9,75921115		7,48245925
3,6644591		8,73689998		9,96573237		7,08379992
4,11095007		9,14552612		9,65332859		7,41568744
3,94200963		8,35312246		10,15018		6,8365518
3,79548643		8,84137345		10,1946146		6,96854515
3,87537974		8,77061537		10,4447922		7,3033412
4,26935989		8,93353725		9,6958409		6,81473255
3,64549426		9,07123314		10,0916576		7,44086228
3,95365309		9,00932247		10,3514171		7,57584551
3,79779906		8,87823194		10,4234872		7,55755736
4,06785964		9,05969131		9,77349976		7,36852033
3,98433562		8,94998436		10,1208321		6,90587394
3,93845331		8,43135273		9,66458821		7,12971953
3,94731074		8,57513336		10,0005849		7,44353179
3,82294392		8,99091309		10,5437584		7,0455484
3,92979038		8,9485534		10,2226841		7,30019066
3,85868362		8,89531871		9,56039883		6,92367947

3,73074389	8,76831595	9,99428057	7,28277389
3,85715622	8,66830995	9,86221219	7,44797438
3,71852376	8,94080853	9,90941894	7,42899633
3,70081042	8,63034108	9,76936685	7,4255279
4,02254943	8,49535947	9,70421191	7,51518548
3,86887415	8,56677028	10,1407353	7,21976791
3,78391271	8,95614371	9,86459645	7,80957006
4,09779549	8,73798007	9,8342913	7,26372781
3,96933617	8,87341448	10,4698095	7,22872778
4,00604478	9,02572255	10,39687	7,36283031
3,88422378	8,81964424	9,95283107	7,47282809
4,06680411	9,13211659	10,3382339	7,16024335
3,60563407	8,94407642	10,059788	6,97743972
3,70411499	8,86762666	10,2837201	7,91526263
3,75109453	8,58408884	9,67108966	7,04537631
3,6925669	8,82271086	10,0804587	6,98398968
3,78500328	8,75984789	9,48036196	7,26863974
4,37096036	8,67517622	9,94615598	7,26149212
3,9416813	9,37804021	10,215486	6,82481994
3,96360709	8,72938802	9,9179229	6,85249832
3,77785405	8,70301372	9,64732553	7,01342362
3,94607064	8,96818893	10,0083331	7,73971422
3,86442075	8,8442316	9,50816833	7,51301443
3,90029622	8,87392917	9,86770053	7,46727066
4,01613851	8,69460529	9,50130357	7,59911249
4,14984971	9,10522735	9,6720737	6,41389296
3,83621505	8,8434417	10,6099273	7,35831876
3,98043375	9,12524505	10,0930754	7,47999417
3,89175682	8,4960692	9,04241202	7,21369052
4,84713498	8,76714412	9,69519228	7,12942627
3,94662081	8,604012	10,0168112	7,04945649
4,52827524	9,00311786	10,0623492	7,04547722
3,79416457	8,92187006	10,3124873	7,20483391
4,30644987	8,79044053	9,04426831	6,92122933
3,93633567	9,17600886	9,60887417	6,9584651
3,96352792	9,06889852	9,97990968	7,43019329
3,76503546	8,85516608	9,97151312	6,86369479
3,71353331	8,75395842	9,83792592	7,24293642
3,67379289	9,03713944	9,95676701	7,19923604
3,60674316	8,67217514	10,2864287	7,15897845
3,68262788	8,84499492	9,56188453	7,02712004
3,903409	9,0338434	10,089303	7,19821751
3,92148057	8,87736259	10,0183415	7,25570664
3,86780286	9,06349691	10,049818	7,07698815
3,70367322	8,64600375	9,8330866	6,95037867
4,39264985	8,711376	10,0068702	7,21928415
3,6886456	8,5106865	9,70027194	7,12384746
3,75353429	8,4242319	9,84314736	7,34440447
3,87105228	8,77150132	9,94108577	7,20460677
3,83607303	8,64703178	9,69181893	7,24879724

4,06239721	8,90789251	9,97711142	7,42423346
3,99418549	8,36826877	9,72697354	7,46835697
4,13631165	8,606336	9,55218782	7,36941476
3,84355402	9,15782028	10,7500475	7,34717783
3,82433358	8,80779025	9,77841789	7,18644458
3,79051356	8,85463959	10,0564472	7,01543915
3,80715105	8,75305516	10,0130538	7,06874534
3,50401713	8,96051187	10,4403978	7,50542022
3,74116324	9,29480257	9,36361837	7,05274564
3,6632241	8,80226563	9,8596363	6,86615738
3,61968222	8,80754112	10,1189817	7,36404152
4,3399607	9,51091085	9,95953566	7,42752076
3,98254876	8,91408707	9,88814489	7,36684102
3,71187991	8,81505279	10,2104695	7,23339582
3,8776824	8,68887208	9,88110128	7,31539908
3,79594494	8,68951839	9,7327936	7,17749981
3,67565152	9,01260389	9,95593168	6,87265901
3,93946735	8,76254823	9,96797265	7,1168405
3,92603382	8,74374208	9,86151064	6,91663473
4,04314424	8,85625386	10,2950784	7,25899017
3,80434777	8,81523251	10,218165	7,03870701

Controls	PDAC	Controls	PDAC	Controls	PDAC
**MAP1LC3A**	**MAP1LC3A**	**MAP1LC3B**	**MAP1LC3B**	**SQSTM1**	**SQSTM1**
6,08808336	4,66869425	7,71351859	8,7786385	9,77659811	10,3259075
5,70618739	4,8574902	7,40304527	8,163622	9,68260916	9,6416233
4,67007799	4,51548699	7,721392	8,93568889	9,78426436	10,2619573
4,74890202	4,48319855	7,48534949	7,82735429	9,67079	10,1415431
5,07699415	4,57203356	7,32511394	7,94669308	9,75936555	10,1785347
4,16266241	4,13595981	7,63052708	8,20775161	9,798601	10,3243118
5,00743612	4,19737994	7,77090664	9,13681193	9,76096297	10,2761147
5,25635033	4,29056329	7,0182027	8,77207056	9,95309265	10,2446432
4,61026945	3,77680588	7,64849198	8,90784073	9,97286388	10,1844737
6,62234492	4,47807371	7,33989398	8,46564839	9,64091331	9,85057325
5,65430741	5,09477278	7,73190149	8,3194609	9,78704288	9,61664417
6,06389671	4,40264584	7,57100753	8,69962872	9,80621718	9,50714285
5,82314302	4,16327925	7,7760169	8,34092446	9,84356548	9,96837607
	4,16418737		8,78539972		9,81407862
	4,80827802		8,382626		9,84589148
	4,789261		9,03811659		10,2449005
	4,41883751		8,68532062		9,99425291
	4,38212398		8,73534541		10,1132405
	4,61418283		8,72641136		10,0877363
	4,53633715		8,61432061		10,2112034
	4,01084979		8,95559866		9,80269008
	4,73302342		8,79440743		10,3161206
	4,45027665		8,40081604		9,84285735
	4,79252476		8,70589901		9,96918444
	4,00149847		8,14216431		10,128415
	4,29286809		8,65448282		9,91978974
	4,5669582		8,42381615		10,0274985
	4,29599175		7,98890169		9,97348962
	4,70141867		8,53721205		9,78922983
	4,58363351		8,85861034		10,1491159
	4,31700992		8,94813312		10,1543306
	4,10477777		8,77068598		9,91614587
	4,55502346		8,6251216		10,3520943
	4,57860424		8,58125209		9,85194328
	3,73934945		8,21800145		10,4289455
	4,73904308		8,80102378		10,1600121
	4,10975003		8,40995044		9,95162095
	4,44606112		8,74119972		9,98610772
	4,32267828		8,87112038		10,1535657
	4,43450313		8,35163826		9,98721013
	4,46143701		8,45253376		10,1159447
	4,32682348		8,64544854		10,5971396
	4,54265423		9,27067837		10,3567645
	4,06902654		9,0238534		10,4678691
	3,75773537		8,61980417		10,5069168
	4,65885994		8,26085197		10,7741287
	4,43737287		8,66240644		10,5953093

4,33172759	8,85782779	9,81580886
4,66175834	8,63065001	9,66974096
4,23182726	8,64978109	10,0974262
4,26595361	8,45631668	10,1852815
4,18537513	8,6450691	9,90011421
4,78121205	8,64106573	9,83401476
4,02402847	8,59440052	10,9099537
4,10501742	8,11786345	9,52761293
4,54888251	8,66908564	9,99983936
4,64696138	8,87943863	10,4818232
4,53334412	8,59681219	9,97102482
4,67974529	8,80286859	10,1275428
4,12689508	8,71505459	9,86369314
4,34551272	9,02936178	10,6412079
4,50688744	8,34901626	9,95300339
4,93928016	8,84524467	9,67769379
4,08911396	9,09509133	10,1688343
4,48628069	8,73452002	10,2834153
3,98728091	8,84604076	9,86702782
4,02847457	8,47933564	10,5413104
4,41131541	8,35022815	9,8548854
4,55835819	9,28557691	11,0550427
4,57075771	8,42385457	10,3765719
4,51372593	8,74728371	10,1729847
4,71642344	8,36069473	9,81337137
4,73361393	8,37503299	10,1757938
4,12739254	8,77937925	9,71601982
4,85479136	8,41361234	10,2086833
3,9283881	8,85121619	10,8846414
3,98384792	8,57334127	9,64268648
4,43737287	8,8534651	10,1657866
3,99238628	8,25005539	9,66819463
4,5659063	9,21496826	9,92258545
3,9746126	8,31536351	10,3939455
4,57645717	8,25587512	10,6187213
4,57645717	8,17826496	9,8452542
5,59515184	8,80859632	10,2914614
4,73503796	9,05893672	10,5276091
3,93684072	8,75838527	10,2210366
5,04415971	8,41724662	9,78283917
4,16662281	8,549633	9,68396891
4,32993784	8,61259376	10,0939335
4,42703084	8,66303621	9,82944381
3,93700781	8,62089793	9,9170979
4,38880095	8,74037819	10,1996434
5,56291846	8,1284033	9,58926867
5,03586999	8,34180219	10,7387651
4,14499694	8,71462644	10,1153897
4,72745439	8,60590768	9,90018003
4,18329205	8,42071389	9,85468352

4,33131743	8,92953246	10,4790532
4,25912446	8,46465544	10,2110963
4,63669757	8,59088857	9,58664256
4,43809952	8,91893651	10,2589435
5,01288277	8,80648169	10,3966903
4,04696065	8,7022233	9,91167314
4,43737287	8,22703026	10,0788528
4,63946094	8,66541952	10,1556501
4,19923834	8,68475913	10,3835986
4,43481141	8,42968637	10,0027777
4,22162387	9,00433163	10,6171356
3,84464258	8,79710486	9,82011452
4,68415924	8,36101153	10,2720481
4,33067523	8,58925753	9,65380943
4,41137423	9,04734159	9,90738629
4,42525642	8,67616266	9,86407388
4,30846401	8,43023635	10,2858042
4,43509867	8,50429785	9,80512106
4,36482891	8,54568035	10,0891412
4,48930505	7,95501945	9,67599789
4,58254086	8,99240304	10,3107377

Supple. Fig. 7A GSE62452

Controls	PDAC	Controls	PDAC	Controls	PDAC	Controls
ATG10	**ATG10**	ATG12	**ATG12**	**ATG13**	**ATG13**	**ATG14**
3,83166	4,36533	4,8287	5,42019	5,78572	6,09229	5,26037
3,30832	4,08558	5,11273	6,26565	5,51767	6,45572	5,33018
4,31011	3,94637	5,216	4,94968	5,66345	5,97178	5,73219
4,65654	3,65333	5,50567	5,45304	5,65978	5,57813	5,30229
4,45426	4,61088	5,42656	5,65647	5,7621	5,92941	5,63266
3,41038	4,60865	5,23077	5,87054	5,8286	5,78886	5,45119
4,26909	4,14773	5,28568	5,55409	6,14665	5,7755	5,22951
4,63901	4,48011	5,49824	5,90266	5,22928	5,79492	5,67661
3,95485	4,01203	5,80234	5,56166	5,73211	6,04686	5,54568
4,66747	4,26722	5,26549	5,48215	5,96283	5,99026	5,66986
4,26914	5,01429	5,33052	6,06696	5,6329	5,8834	5,00637
4,67333	4,73258	5,42084	5,36171	5,74207	5,75205	5,47534
3,92729	4,41229	5,43947	5,76616	5,71993	5,94316	5,32576
4,96917	4,59395	5,88872	5,97794	5,87417	6,13697	5,75599
4,45342	4,802	5,59988	6,5246	5,7697	5,78218	5,28783
3,70031	4,64173	5,16018	5,50007	5,70149	6,02292	5,31391
4,27935	3,94801	5,57319	5,44577	5,77839	6,01926	5,65417
4,66501	4,35253	5,0169	5,24923	5,83672	5,88314	5,38641
5,05541	4,3688	5,80983	5,32352	5,9873	6,14427	6,1272
4,62935	4,92158	5,48087	6,58983	5,8883	5,81156	5,31315
4,51416	4,72041	5,00354	5,42901	5,95464	5,75409	5,76868
3,64728	4,67511	5,10877	5,79379	5,6102	5,87106	4,81585
4,26054	4,91128	5,21038	5,63383	5,7781	5,81666	5,19697
4,90058	4,8503	5,46781	5,64062	5,64885	5,79024	5,51324
4,50082	4,36644	5,41659	6,10811	5,81963	6,2424	5,64178
4,20871	4,42857	5,4247	5,28157	5,90023	6,18977	5,60193
4,57583	4,61858	5,79154	5,60475	5,73243	6,17667	5,91367
4,44258	4,99504	5,4751	5,71307	4,72341	5,89313	4,88901
4,34763	4,48259	5,19604	5,6608	5,8183	5,8869	4,84642
4,05451	4,59254	5,65362	5,69271	6,21643	5,86322	5,20415
4,20036	4,62472	5,68194	5,82884	5,79111	5,8731	5,57534
5,4147	5,09459	5,86305	5,63092	5,71627	5,92618	5,49292
4,6185	4,88414	5,40038	5,46088	5,79346	5,70462	5,58788
4,22051	3,83323	5,51491	5,75419	5,68408	6,18175	5,47846
4,64638	4,88031	5,61	5,95745	5,92393	6,22815	5,90617
5,07595	4,62378	5,77013	5,49677	5,90764	6,0573	5,68083
5,24054	4,90303	5,65744	5,51615	5,89792	5,95228	5,6665
5,28485	4,68165	5,89518	5,3783	5,96388	5,80635	5,59151
4,95348	4,73204	5,10128	5,70999	5,86475	6,10269	5,64247
4,2708	4,42397	5,6741	5,81055	5,81162	6,05426	5,67544
4,39724	4,05026	5,55928	5,27921	5,9642	5,92034	5,67383
4,4743	4,73684	5,51444	6,18697	5,99995	6,40251	5,81574
4,66892	4,90638	5,53349	5,89136	5,98304	5,743	5,57948
3,56808	4,11284	5,37738	5,22898	5,83594	5,74569	5,20438
5,02465	4,4414	5,56882	6,16707	6,05216	5,96386	5,71649
4,72951	4,11872	5,17803	5,25928	5,35597	5,23923	4,64752
4,61436	4,73218	5,24322	5,68167	6,38819	6,24022	5,35367

3,87048	4,76306	5,12689	6,08412	5,64975	6,02342	4,52416
4,11531	5,10372	5,32055	5,79645	6,58039	6,26183	5,8488
4,02759	4,3806	5,44462	5,66642	6,30712	6,30675	5,97707
4,29878	4,22194	5,28511	6,95545	5,34619	6,11152	4,7971
4,7727	4,93345	6,15233	5,41847	5,27475	6,19577	5,94541
4,45727	4,63541	5,37342	5,77212	5,35996	6,05808	5,07074
4,07534	4,35791	5,54684	6,49823	5,838	5,5801	5,57947
4,22027	4,81082	4,9013	5,85749	6,33531	6,35271	5,17037
4,30804	3,67787	5,0942	5,18632	5,84214	5,98752	5,48416
4,68893	4,88629	5,32569	5,81522	6,00901	6,01217	5,56398
4,81173	4,47918	5,28329	5,27313	5,56182	5,83664	4,90379
4,40093	4,48924	5,5821	5,74785	5,60177	5,27078	5,63165
3,74513	4,60107	5,85784	6,69907	5,74883	5,63167	5,34677
4,70339	4,74145	4,67743	6,09261	6,03593	5,11036	4,74614
	5,12477		6,19137		5,9465	
	4,1523		5,53193		5,98175	
	4,30637		5,583		6,25834	
	4,55407		5,62504		4,61214	
	4,83126		5,50514		5,76946	
	4,89125		6,10405		5,80056	
	4,76235		5,42614		6,15211	
	4,05874		5,38644		5,82323	

PDAC	Controls	PDAC	Controls	PDAC	Controls	PDAC
**ATG14**	ATG16L1	**ATG16L1**	ATG3	**ATG3**	ATG4A	**ATG4A**
5,40511	4,24313	5,318	6,1577	6,20957	5,01668	4,26338
5,42466	4,55285	6,26774	5,58807	6,82271	4,1765	4,80595
6,79626	4,89292	5,68195	6,26852	6,05019	4,20497	3,50164
5,41073	5,19443	4,73029	6,34804	6,13921	4,08892	4,15275
5,47157	4,52204	4,73501	6,26956	6,20448	4,26263	3,89477
5,00997	4,21697	5,05138	5,57033	6,4436	4,04545	4,42332
5,93473	4,41075	4,89273	6,3795	6,10424	4,74857	3,95447
5,64087	4,8935	5,42492	6,18828	6,73131	4,26579	4,89834
5,50086	4,54073	5,32558	6,50151	6,65463	4,5638	4,38483
5,55865	4,89074	5,08502	6,22118	6,55701	4,44125	4,28898
5,78912	4,9437	5,17007	6,26508	6,25526	4,80843	4,46061
5,4704	5,01874	5,10388	6,33827	6,3663	4,15355	4,69883
5,81389	4,15912	5,20673	6,59543	6,09788	4,68278	4,16915
6,52289	4,40574	4,96885	6,56462	5,79325	4,92937	3,95332
5,38026	5,29731	5,26182	6,7496	7,03123	4,39296	5,24214
6,14966	4,45115	5,34071	6,25183	6,29705	4,88441	4,44832
5,53356	4,66703	4,19696	6,39045	6,46935	4,89074	4,74591
5,46636	4,62027	4,29958	6,55331	6,72959	4,65573	4,69795
5,72519	4,73151	5,19699	6,50662	6,2566	4,45854	4,17868
6,06505	4,72471	5,02848	6,43865	6,00606	4,5248	3,91067
5,93896	4,65286	5,2355	5,98504	6,50741	4,14677	4,46936
5,53427	4,26854	4,87244	6,55454	6,48619	4,41649	4,20499
5,94104	4,28585	5,18592	6,4066	6,51607	4,70287	4,42524
5,75581	4,9025	5,29469	6,30981	6,30954	4,58785	4,36345
5,72428	4,46759	5,25582	6,80465	6,89829	5,15003	4,24856
5,55627	5,12726	4,87266	6,20774	6,26042	4,0842	4,19451
5,62907	5,38437	5,28008	6,73454	6,26416	5,35521	4,60648
5,70219	3,88147	5,25499	5,59274	6,71308	3,92115	4,33885
5,80106	4,1177	4,97862	6,85654	6,37659	5,23143	4,19833
5,78212	4,22052	5,22164	6,74664	6,57909	5,45683	4,50748
5,66023	4,66136	5,38226	6,26528	6,19095	4,1986	4,15624
5,68659	5,33035	4,85933	6,81147	6,31694	4,50031	4,22849
5,20412	4,32078	5,07799	6,52105	6,26115	4,92877	4,4656
5,26412	4,83392	4,97575	6,20291	6,38676	4,34253	4,71613
6,20985	4,99418	5,35528	6,31453	6,41155	4,4197	4,51029
5,62378	4,36436	5,22236	6,72018	6,26119	4,88956	4,1993
5,53611	4,29301	4,86981	6,43499	6,46477	4,51442	4,8914
5,54251	4,81739	4,69644	6,58992	6,53112	4,59884	4,78973
5,79163	5,02475	5,04712	6,15835	6,2148	4,4727	4,20753
5,23083	4,78321	4,99222	6,32652	6,38131	4,25572	4,47728
5,31056	4,57504	4,23779	6,73054	6,21607	5,17685	4,72025
6,13408	4,59394	6,07869	6,80316	6,39188	5,53653	4,40821
5,80511	4,70728	5,39494	6,20825	6,49275	4,81062	4,26675
5,31692	4,2266	5,06085	5,9424	6,24663	3,9257	4,49427
5,53513	4,68155	5,37959	6,36253	6,19622	4,42263	4,64415
5,71908	4,82732	5,77804	6,09234	5,68157	4,56375	3,86832
5,9483	4,65935	5,72923	6,19684	6,56674	4,53787	4,02484

6,65411	3,88351	5,33265	5,90789	6,92194	4,33419	4,75002
6,07321	4,74602	5,56569	6,09767	6,63322	4,61037	4,35392
5,75676	4,82641	5,19928	6,6398	6,40192	5,16034	4,63306
5,87424	4,12487	5,16824	6,50619	6,72783	5,14821	4,38291
6,21615	4,94434	5,13315	6,98787	6,31388	4,27249	4,35542
6,18008	4,21291	5,04124	5,91174	6,3137	4,51001	4,41593
6,19707	4,3891	4,95778	6,52612	7,00553	4,94074	4,61659
5,99802	4,88737	5,57104	6,032	6,92223	4,57252	4,58142
5,53546	4,69172	4,54547	6,06222	6,07144	4,3221	4,21076
5,71918	5,01014	5,86201	6,26671	6,49801	4,32519	5,28076
5,09421	4,07242	4,95208	6,35175	6,24545	4,41501	4,39341
5,55064	4,34856	4,43829	6,20783	6,56994	4,68648	4,4484
5,74734	5,1944	5,44228	6,73388	6,40524	4,01443	4,07199
6,03159	4,59581	5,2281	5,72053	6,76993	4,81547	4,12232
6,08594		5,53373		7,07976		4,26305
5,63223		4,92233		6,49487		4,38951
5,55424		5,33847		6,48704		4,3061
5,10715		3,89129		6,12819		4,50718
5,88791		4,42852		6,4613		4,43015
6,14908		4,93602		6,88877		4,20223
5,27979		5,11834		6,20256		4,34112
5,19964		4,38163		5,76898		4,53411

Controls ATG4B	PDAC **ATG4B**	Controls ATG5	PDAC **ATG5**	Controls ATG7	PDAC **ATG7**	Controls ATG9A
5,6937	6,19114	5,94862	6,23194	4,6772	5,88709	5,05609
6,00417	6,30965	5,75089	6,52493	5,93064	5,90219	4,81097
5,93722	7,028615	6,03421	6,25707	6,13999	5,74939	5,3133
5,772245	5,69405	6,11577	6,03431	5,95406	5,66923	4,99231
5,672025	5,597995	6,12702	6,55361	5,90137	5,6771	5,1926
6,251605	5,641955	5,51831	6,31338	5,6872	5,77281	5,41262
5,70727	5,850965	6,17792	5,89399	5,8024	5,5762	5,14341
5,63308	5,625065	6,03328	6,63959	5,85906	5,52567	5,27112
5,74508	5,50816	6,13655	6,57858	6,01669	5,75577	5,13789
6,141875	5,895935	5,88706	6,40055	6,40964	6,02706	5,21397
5,35195	5,59191	5,90239	6,2042	4,93819	6,40516	5,1087
5,652005	5,66528	6,10296	6,31226	6,02469	5,91625	5,15981
5,70424	5,887935	6,16937	6,07684	4,78832	5,89295	5,18355
5,28741	6,21453	6,61904	6,59366	5,89169	5,26687	4,74207
5,82426	5,49727	6,2497	6,66306	6,35604	6,06479	5,13662
5,597155	6,159085	5,71339	6,3695	4,87004	6,34172	5,11467
5,351995	5,7374	6,03834	5,81569	5,216	5,66402	5,10182
5,475305	5,434125	6,04549	6,24145	5,17101	5,27041	5,14905
6,042115	6,234815	6,43867	6,01221	6,5229	6,42713	5,13466
5,87147	5,87523	5,96287	6,12801	6,16709	5,96968	5,15237
5,80674	5,912705	5,99914	6,37353	6,13895	6,56574	5,107
6,13831	5,743575	5,44874	6,09983	5,8792	5,96401	5,08739
5,52872	5,998655	5,61742	6,44513	4,70881	6,37041	5,05756
5,94302	5,870485	6,18242	6,37201	5,73561	6,08873	5,13342
5,211365	6,232785	6,19813	6,45222	5,00142	6,13732	5,20412
5,76223	5,915935	6,13607	6,30372	5,97428	6,14767	5,28649
5,9755	5,814615	7,12494	6,1975	5,72054	6,0232	5,52278
5,280055	5,99167	4,74679	6,26268	4,69294	6,3301	5,05835
5,30992	5,824	6,09445	5,78997	4,98259	6,17037	5,44035
5,486755	5,85205	6,35527	6,6712	5,13236	6,2806	5,12218
5,767515	6,20764	6,0102	6,26391	6,26753	5,9694	5,06663
6,07213	5,89894	6,07799	6,27843	5,70678	6,15126	5,28471
6,000515	5,73303	6,06175	6,5178	5,44885	5,90247	5,06808
5,724095	6,020795	5,9724	6,11214	6,12002	5,87838	5,16228
5,94183	6,034835	6,08625	6,20819	6,30525	6,36642	5,08459
5,61376	6,15321	6,36875	5,99204	5,76457	6,17681	4,85159
5,714675	5,82172	6,18129	6,51603	6,13447	5,8597	4,99459
5,69918	5,99874	6,71262	6,30951	6,39831	6,09138	4,79507
5,959525	5,66777	6,433	6,12606	5,99327	6,2318	5,21243
5,70247	5,95782	6,33183	6,51958	6,06928	6,07103	5,1484
5,743195	6,25228	6,32583	5,99585	5,44562	5,04117	4,89831
5,731195	6,540305	6,68425	6,54958	5,031	6,13417	5,05613
6,00926	5,89783	6,22596	6,61094	5,91716	6,28332	5,16249
6,2308	6,437795	5,94025	5,88923	5,58263	5,98219	5,13715
6,253475	6,10488	6,52456	6,23746	6,3541	5,96348	5,1849
5,72728	6,04982	5,53367	6,27912	5,06996	6,01143	5,45995
5,84767	5,932695	6,01951	6,13623	6,25731	6,6051	5,61845

5,838	6,19099	5,376	7,03647	4,58258	6,15954	4,67305
6,1482	5,761855	6,33261	6,40175	6,50746	6,65517	5,58547
5,76407	6,26575	6,38628	6,16588	5,54177	6,11674	5,03403
5,60563	5,76859	5,90985	6,41975	4,64458	5,67295	4,82517
5,562545	6,333435	6,59755	6,56716	5,82738	6,24086	4,95612
5,76217	6,27193	5,85354	6,7171	5,14377	6,22803	4,81649
5,3665	5,596985	5,91061	7,15105	5,18511	5,91981	5,02164
6,03152	6,035955	5,72275	6,43262	5,67427	6,22549	5,07005
5,78916	6,037475	5,52281	6,02822	5,64889	6,21455	5,20743
5,78348	5,8155	6,59438	6,79702	6,273	6,00514	5,55376
5,187685	5,883005	5,59588	6,01071	5,24526	6,05063	4,96451
5,672235	5,69453	6,01143	5,94429	5,78007	5,43055	4,83915
6,085895	5,881155	6,45928	6,9039	6,18448	6,1107	5,06324
5,80728	5,75083	5,68908	6,68369	5,51367	5,68144	5,18539
	6,261565		6,76423		6,22729	
	5,856045		6,19091		6,23441	
	5,83082		5,93066		5,86619	
	5,698335		5,98312		4,9273	
	5,79076		6,38433		5,71753	
	5,685765		6,67552		6,36754	
	5,89168		6,09225		6,34743	
	5,6831		5,54288		5,45387	

PDAC<br>**ATG9A**	Controls<br>ATG9B	PDAC<br>**ATG9B**	Controls<br>**BECN1**	PDAC<br>**BECN1**	Controls<br>**LAMP1**	PDAC<br>**LAMP1**
4,95894	4,75057	4,67382	5,42689	5,61652	8,07929	8,21265
5,77153	4,29959	5,65509	5,18981	5,28481	7,92254	7,86201
5,73525	4,27984	4,64438	5,32029	5,71553	8,07606	8,34205
5,02773	4,06981	4,35081	5,24368	5,43847	7,75114	8,03437
5,31352	4,25563	4,56168	5,14557	5,22015	8,0108	7,79388
5,22819	4,46981	4,18145	5,22206	5,4349	8,35254	8,1454
5,21271	4,30063	4,64867	5,70652	5,45782	7,92599	8,13814
5,24046	4,35396	5,12609	5,17564	5,46646	7,79257	7,69511
5,26338	3,9757	4,06756	5,39391	5,48903	8,13734	7,8057
5,02321	4,10806	3,98925	5,65089	5,8028	8,04846	7,89236
5,08811	4,42375	4,21121	5,33144	5,45034	8,05332	7,72781
5,22135	4,26067	4,45967	5,37679	5,30923	7,82932	7,69197
5,11904	4,35229	4,77878	5,43693	5,48282	8,00457	8,10727
5,39241	4,21567	4,73578	5,32771	5,45777	7,63482	8,0405
4,81024	4,19728	3,93106	5,33339	5,4107	8,60397	8,02968
5,26092	4,55552	4,15523	5,31699	5,71119	8,28485	8,22373
5,32307	4,05027	4,20783	5,57737	5,52174	8,10738	8,1946
5,23604	4,00137	4,34096	5,56839	5,5445	8,18773	8,07191
5,32421	3,83085	4,0715	5,45196	5,48232	8,19893	8,16558
5,14625	4,00999	4,05356	5,54525	5,6632	8,16947	7,99847
5,02847	4,2983	3,84357	5,25773	5,52362	8,17993	7,90645
5,12581	4,41152	4,08529	5,34258	5,47439	8,12855	8,07355
4,94833	4,56436	3,87631	5,25709	5,47872	8,11583	8,24993
5,10007	4,02912	3,93509	5,51058	5,48732	8,16589	8,19063
5,24024	4,24589	4,8527	5,42764	5,49129	8,08809	7,75281
5,24367	4,33632	4,35457	5,22237	5,55937	8,10074	8,15983
5,52002	4,02549	4,0145	5,52462	5,82411	7,77608	8,04825
5,33375	5,22345	3,8414	5,04726	5,66947	7,57522	8,3783
5,03098	4,19208	4,54471	5,20401	5,19484	8,09048	8,3217
5,20205	4,7556	4,42157	5,60726	5,61694	8,19768	8,16776
5,36997	4,32002	4,56834	5,52586	5,74153	8,09592	8,26892
5,23545	3,95868	4,28995	5,52214	5,57091	7,96477	8,38468
5,15516	4,35346	4,61387	5,41116	5,44572	8,0612	7,85713
5,08309	3,86917	4,62624	5,35117	5,43784	8,19102	7,87543
5,21737	4,03363	4,18251	5,30671	5,64381	8,09876	7,90213
5,22508	4,18641	4,27566	5,64489	5,45801	8,01634	8,54748
4,95796	4,15517	4,11812	5,46522	5,58527	7,96085	8,19273
4,98594	3,75214	4,05554	5,54006	5,6745	7,9963	8,00749
5,19506	4,19306	5,18718	5,05774	5,53992	7,8485	8,23357
5,27384	4,0374	4,34215	5,42845	5,51159	8,18164	8,34566
5,00108	4,23125	4,58644	5,74125	5,43887	8,19605	8,12869
5,56388	4,52641	5,05277	5,35593	5,16594	8,12612	8,25902
5,21211	4,20004	4,00458	5,4828	5,75244	8,01331	8,21404
5,35627	3,9506	4,25373	5,34129	5,24288	8,16421	8,35161
5,00155	4,20015	4,57292	5,55211	5,46166	8,02377	8,19821
5,02816	4,77983	4,20247	5,41806	5,28037	7,16211	7,74864
5,44499	4,20341	4,88373	5,61192	5,76605	8,21943	8,19331

5,20247	5,25566	4,2196	5,27774	5,92018	7,55723	8,14108
5,38316	3,9438	3,90286	5,47885	5,47519	8,31069	8,40783
5,0777	4,1582	4,94244	5,74948	5,40969	8,4352	8,11244
5,07822	5,06401	4,15461	5,16406	5,43181	7,62421	8,38821
5,28613	4,34628	3,90616	5,42549	5,62504	7,85258	8,43518
5,22329	4,891	4,29946	5,02156	5,66139	8,19495	8,32546
4,53927	4,6237	4,63083	5,79993	5,6879	8,24848	8,13276
5,2782	4,40191	3,7994	5,44393	5,76642	8,28682	8,34888
5,44646	4,43982	4,10217	5,51868	5,20186	8,07271	8,27249
5,66373	3,9526	4,07558	5,53813	5,37187	8,20234	8,2288
5,28637	4,42363	4,15371	5,54708	5,14282	7,68702	8,28176
4,65916	4,25272	3,94615	5,28982	5,19	7,77885	7,68911
5,18135	4,21973	4,05429	5,34847	5,61861	8,2954	7,91461
4,84069	4,21378	4,11816	5,24767	5,19064	8,11356	7,96981
5,10057		4,04217		5,75862		8,04998
5,2347		4,02391		5,60832		8,10334
5,50715		4,92513		5,91612		8,09201
4,75054		5,00151		5,1596		7,24799
4,56625		4,35595		5,22913		7,97135
4,95045		4,17677		5,32712		8,24505
5,31497		4,03431		5,12414		8,05115
5,17674		4,54396		5,36781		8,00312

Controls	PDAC	Controls	PDAC	Controls	PDAC	Controls
**LAMP2**	**LAMP2**	**MAP1LC3A**	**MAP1LC3A**	**MAP1LC3B**	**MAP1LC3B**	**SQSTM1**
7,28569	7,37522	4,67593	4,21409	6,46313	5,88716	6,25242
7,15452	7,75156	4,3072	4,30176	6,49029	6,30951	6,56481
7,23231	6,43844	4,77275	4,91745	6,59141	6,14149	6,6242
6,96534	7,09637	4,21137	4,45381	6,43762	7,11361	6,28438
7,55093	7,45746	4,62189	4,21264	6,62288	6,40398	6,00391
6,99484	7,78456	5,16872	4,51651	6,26477	6,5565	7,0365
7,68006	7,38344	4,19461	4,17533	6,29803	6,4762	6,94964
7,16034	7,39568	4,2027	4,31795	6,75301	7,16675	6,32194
7,64733	7,65361	4,43485	4,05058	7,14521	6,41781	6,99044
7,65709	7,72906	4,06856	3,97528	6,09753	6,74717	6,74562
7,39737	7,68071	4,11813	3,85816	6,19618	6,17802	6,84051
7,17476	7,54867	4,39218	4,30357	6,74594	6,57584	6,63894
7,70466	7,6566	4,65848	4,39156	6,37911	6,35726	6,2517
8,11841	7,9655	4,26078	4,22549	6,70334	6,53705	6,27723
7,88086	8,5398	4,30206	4,00469	6,40718	6,54216	6,87332
7,50892	7,40841	4,59654	4,23295	6,25545	6,62156	6,11281
7,57116	7,68293	4,44624	4,35066	6,63319	6,65684	6,14012
7,75899	7,76203	4,42551	4,72743	6,40528	6,84413	6,41188
8,14752	7,40568	4,16285	4,17859	6,2677	6,12953	6,3796
7,89351	7,50784	4,3425	4,20751	6,40077	5,86004	6,75487
7,57544	7,37344	4,45636	4,49197	6,47794	6,18798	6,74892
7,26034	7,7471	4,70718	4,40759	6,88242	6,16168	6,89769
7,01537	7,84314	4,72169	4,43561	6,16812	6,17852	6,29771
7,75328	7,78243	4,41001	4,19206	6,6884	6,63958	6,543
7,60828	7,356	4,57163	4,14843	6,49257	6,88813	6,53093
7,47425	7,5294	4,34755	4,36847	6,63831	6,64486	6,77466
7,65206	7,61286	4,66707	4,59687	6,21419	6,49616	5,97251
6,59605	7,81865	4,69069	4,15737	5,91174	6,07293	6,18226
7,67574	7,43677	4,79948	4,43112	6,66653	6,50194	6,74198
7,63305	7,51349	4,69269	4,33647	6,3623	6,3703	6,12504
7,64435	7,55878	4,34416	4,32559	6,31496	6,45523	6,5769
7,85829	7,82534	3,95524	4,12488	6,36647	6,55557	6,58364
7,65286	7,18592	4,38407	4,24904	6,16042	6,57785	6,43153
7,66162	7,40725	4,55296	4,50997	6,04508	6,28979	7,05713
7,69121	7,9168	4,68054	4,28872	6,33267	6,43805	6,53789
8,05452	7,54396	4,38476	3,72985	6,40368	6,38969	5,98254
7,58919	8,03391	4,52602	4,17797	6,72818	6,09861	6,40787
8,39327	7,95291	4,08731	4,40892	6,80743	6,04094	6,56575
7,76553	7,72347	4,22297	4,46549	6,10148	6,48148	6,61805
7,6894	8,27952	4,28219	4,64482	6,46538	6,53204	6,5701
8,06798	7,42351	4,34448	4,17531	6,20077	6,02467	6,08492
7,93406	7,95405	4,52288	4,12567	6,43317	6,3996	6,30943
7,70435	7,64163	4,17601	4,14444	6,28766	6,45382	6,44239
7,45103	7,48481	4,52987	4,37143	6,63941	6,61212	6,52446
8,15224	7,60019	4,1385	4,27624	6,36143	6,60253	6,96626
6,55847	6,55869	4,36256	4,50626	7,27983	6,31709	6,63678
7,90119	7,92226	3,99323	3,85098	6,03804	6,78279	6,78938

6,43159	8,12451	5,13916	4,37353	6,52976	6,53184	5,73376
8,26568	8,05509	4,48106	4,14785	6,04857	6,81731	7,05548
7,86964	7,59666	4,57631	4,59064	6,51135	6,40338	6,51947
7,14855	7,84125	5,16885	4,97766	6,20833	6,94293	5,89762
7,81693	8,14953	4,11073	4,20691	6,22525	6,20665	6,39089
6,98331	7,65218	4,45227	4,47863	6,68737	6,02094	6,28517
7,46916	8,19263	4,5458	4,27799	6,63486	6,36643	6,5772
7,51625	8,05219	4,37315	3,86388	6,57095	6,26871	6,9818
7,34063	7,77767	4,43257	4,48208	6,1503	6,21624	6,83887
8,19342	8,33235	4,2527	3,92386	6,2544	5,96452	6,56895
7,15869	7,68477	4,69596	3,95964	6,72971	6,34583	6,7068
7,44766	7,79797	4,73317	4,49239	6,82195	6,7433	6,26457
7,64781	8,35365	4,22632	4,54378	7,27492	6,44315	6,78692
6,99019	7,57603	4,18898	4,40684	6,17551	6,46649	6,44924
	8,2802		4,09845		6,4944	
	8,08388		4,03444		6,10778	
	7,90842		4,32387		5,9926	
	6,60763		4,57474		6,48397	
	7,75313		4,15739		6,57976	
	7,92408		4,20863		6,80442	
	8,06306		4,29448		6,61628	
	6,90132		4,16838		6,17544	

PDAC
**SQSTM1**
6,79785
7,01211
7,04715
6,85943
6,4311
6,76884
6,78092
7,12836
6,57145
6,99131
6,45886
6,34016
6,64419
6,97602
6,73545
6,65322
6,04149
6,3705
6,7893
7,0474
6,30989
6,78864
6,73153
6,6947
7,53181
6,68067
6,4951
6,59756
7,12096
6,90354
6,68983
6,93349
6,60388
6,52164
6,98233
6,6402
6,67042
6,77599
7,12775
6,41356
5,99483
7,35517
6,36781
6,57463
6,77839
6,66252
7,07528

6,81471
6,17659
7,05278
6,95635
6,56021
6,87043
6,6029
7,03406
7,1326
7,07315
6,4957
6,4667
7,18848
6,32392
6,51889
6,5742
6,70578
5,81512
6,22755
6,41128
6,74127
6,61673

Supple. Fig. 7B GSE15471

Control ATG5	PDAC Atg5	Control BECLIN1	PDAC BECLIN1	Control LAMP2	PDAC LAMP2	Control LC3B
7,428473	7,410547	6,78167	6,568439	8,655197	8,941969	9,223273
7,485524	7,572092	6,731189	6,733182	8,6407	9,073276	9,027329
6,948749	7,768761	6,459689	6,748488	8,672541	9,02062	7,932327
6,843474	7,546379	6,720195	6,668525	8,860093	8,951564	7,820573
6,955489	7,514865	6,81589	6,753853	8,701956	8,775059	8,053624
6,928521	7,503115	6,780003	6,699864	8,619782	8,827747	7,93888
7,05738	7,398125	6,685357	6,587394	8,559202	9,165722	7,730088
7,18439	7,266597	6,612749	6,45246	8,024456	8,797205	9,187367
6,640364	7,021856	6,727666	6,681367	8,64473	8,923067	8,347809
7,176798	7,284297	6,502826	6,75416	8,795231	8,553844	9,592856
7,359224	7,305876	6,814888	6,536739	8,445447	8,684826	9,11823
7,912536	7,640276	6,823172	6,722081	8,717984	8,825585	8,927675
7,81497	7,003157	6,974929	6,53076	9,022046	9,232127	8,87563
7,741964	6,881765	6,879846	6,502003	8,880916	8,819621	8,984503
6,926222	7,28007	6,645186	6,93898	8,493064	8,810241	8,530747
7,513763	7,444481	6,388457	6,314279	8,655928	8,639402	9,221911
7,123935	7,349933	6,705843	6,463082	8,879708	9,295075	8,348697
7,157465	7,195706	6,664927	6,685743	8,783455	9,157821	8,300674
6,772883	7,576151	6,80276	6,591669	8,470434	9,38911	8,203079
6,691917	7,191678	6,627497	6,503194	8,699554	9,179751	8,01861
7,350988	7,326157	6,502204	6,61265	8,871376	8,742568	8,870842
7,380518	7,38994	6,296861	6,654891	8,825869	9,221788	9,342161
7,097376	7,886996	6,565779	6,78112	8,806311	9,737446	8,093878
6,968488	7,442104	6,359838	6,728303	8,824346	8,978703	8,477338
7,111198	7,857395	6,724798	6,636915	8,677733	9,073494	8,048705
6,799356	7,371251	6,603583	6,656583	8,639478	8,927213	7,984119
7,278762	7,36704	6,727089	6,68664	8,991471	8,54292	8,430919
6,988224	7,27005	6,742206	6,724693	8,898641	9,006427	8,170275
7,401475	7,746308	6,712174	6,669007	8,294304	9,028923	9,175004
7,310574	7,689754	6,604565	6,704695	9,387271	8,865595	9,225983
7,703317	7,130075	6,807478	6,667756	8,611836	8,744083	8,53525
7,930062	7,580139	6,939394	6,68804	8,604945	8,607784	8,564323
7,138085	7,326774	6,669735	6,773296	8,724501	9,272309	8,109084
6,78952	6,967832	6,500608	6,698042	8,710176	9,000046	7,958508
6,833155	7,165157	6,898208	6,70562	8,674403	9,083979	7,786009
5,331276	7,485055	5,71519	6,542326	5,773001	8,771533	6,075614
6,849644	7,157173	6,50286	6,625674	8,40521	8,575991	8,044807
6,875115	7,285732	6,752151	6,670319	8,488217	8,893129	7,941707
7,318519	7,005601	6,590051	6,436691	8,203063	8,715071	8,986547

PDAC LC3B	Control p62	PDAC p62
9,591459	6,966724	7,195358
9,489085	7,170123	7,346204
9,476961	7,436549	7,337122
9,35507	7,342786	7,358122
9,275806	7,343932	7,344387
9,162753	7,345701	7,207111
9,008071	7,108156	7,616097
9,662798	6,979062	7,134595
9,033166	7,415571	7,305755
9,250983	7,472714	7,329902
9,261524	7,235678	7,366752
9,261195	7,050091	7,325028
9,362896	7,073753	7,39262
9,178645	7,271273	7,302457
8,935018	7,562744	7,981413
9,262382	6,946259	7,073411
9,163842	7,012809	7,144622
8,868568	7,251087	7,443406
9,272828	7,552467	7,318148
8,963648	7,182735	7,189747
8,911352	7,129283	7,200647
9,131058	7,475205	7,634905
9,311001	7,238385	7,69738
9,008115	7,182068	7,43189
9,458609	7,241547	7,527384
8,977838	7,555415	7,303243
9,408224	7,373115	7,228549
9,164809	7,146164	7,393649
9,405808	7,165034	7,184876
8,752723	7,231148	7,440966
8,628532	7,203906	7,281763
9,036436	7,06757	7,76948
9,069677	7,370916	7,282616
8,419668	7,384	7,217462
9,177495	7,242597	7,418948
8,916135	7,236151	7,143667
9,210276	7,525883	7,347467
9,242675	7,234007	7,054096
8,667617	7,050834	7,162365

Supple. Fig. 7C

GSE62165	P-wert	Fold changes	P-Wert -LOG10	Fold changes LOG2	GSE15471	P-wert
**1CD274**	0,36821278	0,98170845	0,43390114	-0,02663346	**2CD274**	0,6492613
**1TLR4**	3,0633E-09	1,1978538	8,513815	0,26045183	**2TLR4**	0,00014292
**1ATG10**	0,17063858	1,02332764	0,76792278	0,03326812	**2ATG10**	0,00211148
**1ATG12**	1,3976E-05	0,94640607	4,85462607	-0,07946876	**2ATG12**	0,00015394
**1ATG16L1**	7,3456E-09	1,09807235	8,13396977	0,13497312	**2ATG13**	0,00054403
**1ATG2B**	6,1439E-16	0,8726279	15,2115585	-0,19656149	**2ATG14**	3,383E-07
**1ATG3**	0,63385719	0,99660334	0,19800858	-0,00490869	**2ATG16L1**	7,2471E-06
**1ATG4A**	2,461E-17	0,89970586	16,6088922	-0,15247467	**2ATG3**	0,16868743
**1ATG4B**	0,25494159	1,01590935	0,59355932	0,02277168	**2ATG4A**	1,0563E-10
**1ATG5**	0,00110364	1,03514562	2,95717161	0,04983374	**2ATG4B**	0,35487194
**1ATG7**	4,2338E-08	1,1152491	7,37327224	0,15736599	**2ATG5**	0,00536921
**1ATG9A**	2,899E-18	0,91984921	17,5377453	-0,12053071	**2ATG7**	5,2483E-08
**1ATG9B**	1,0575E-12	0,89213598	11,9757113	-0,16466446	**2ATG9A**	0,00493313
**1BECN1**	0,0011772	0,97688433	2,92914807	-0,03374035	**2ATG9B**	8,8488E-05
**1LAMP1**	0,98389132	1,00018466	0,00705287	0,00026638	**2BECN1**	0,86120493
**1LAMP2**	0,17152285	1,0150481	0,76567801	0,02154809	**2LAMP1**	0,68320508
**1MAP1LC3A**	1,1078E-13	0,82781829	12,9555483	-0,27261397	**2LAMP2**	0,00034647
**1MAP1LC3B**	4,3714E-25	1,14150137	24,3593784	0,19093259	**2MAP1LC3A**	0,00032766
**1SQSTM1**	0,00053346	1,03180427	3,27289719	0,04516932	**2MAP1LC3B**	5,4151E-08
**1CARD16**	3,1791E-21	1,2696978	20,4976924	0,34448516	**2SQSTM1**	0,01059869

Fold changes	P-Wert -LOG10	Fold changes LOG2	GSE56560	P-wert	Fold changes	P-Wert -LOG10
1,008831	0,18758048	0,01268451	**3CD274**	0,001868299	1,26015309	2,72855372
1,15581076	3,84490503	0,20890521	**3TLR4**	9,1177E-09	1,23976287	8,04011242
0,97780507	2,67541399	-0,03238121	**3ATG10**	0,18163939	1,03396746	0,74078996
1,03875585	3,81264604	0,05485661	**3ATG12**	0,02603803	1,04142825	1,58439193
0,98585392	3,26437726	-0,0205542	**3ATG13**	0,00154781	1,04570786	2,81028338
1,0787181	6,47069836	0,1093179	**3ATG14**	6,421E-05	1,08896295	4,1923951
1,06150657	5,13983465	0,0861133	**3ATG16L1**	1,1776E-05	1,10292031	4,92900938
1,01128416	0,77291728	0,01618844	**3ATG3**	1,3458E-05	1,07558048	4,87101904
0,93645579	9,97619717	-0,09471721	**3ATG4A**	0,07031596	1,0346942	1,1529461
1,00439431	0,44992834	0,00632577	**3ATG4B**	0,00024918	0,96358592	3,60348412
1,03410964	2,27009	0,04838916	**3ATG5**	0,00128335	1,05710154	2,89165381
1,06291046	7,27998015	0,08802007	**3ATG7**	5,6818E-07	1,09536963	6,24551145
0,97882167	2,30687749	-0,03088205	**3ATG9A**	0,76968848	1,0025062	0,11368501
0,93511779	4,05311326	-0,09678	**3ATG9B**	0,00078776	0,91749213	3,10360616
0,9989976	0,06489349	-0,00144688	**3BECN1**	0,18858131	1,0172648	0,72450135
1,00216234	0,16544891	0,00311623	**3LAMP1**	0,13829882	0,98690842	0,85918152
1,03938229	3,46033592	0,05572638	**3LAMP2**	0,0001576	1,09013411	3,80243961
0,97231679	3,48457926	-0,04050165	**3MAP1LC3A**	0,00089235	0,9331308	3,0494643
1,08362507	7,26639364	0,11586568	**3MAP1LC3B**	6,8967E-05	1,08056421	4,1613561
1,01294382	1,97474774	0,01855416	**3SQSTM1**	0,04282999	1,02852322	1,36825206

Fold changes LOG2	GSE62452	P-wert	Fold changes	P-Wert -LOG10	Fold changes LOG2
0,33359901	**4CD274**	1,4953E-05	1,09924221	4,82526257	0,13650931
0,3100642	**4TLR4**	0,071407	1,03477524	1,14625923	0,04931744
0,04819078	**4ATG10**	0,08582888	1,02733753	1,06636657	0,03891025
0,05856345	**4ATG12**	1,5801E-06	1,05559978	5,80131429	0,07806296
0,06447986	**4ATG13**	0,02145779	1,02063489	1,6684151	0,02946686
0,12295487	**4ATG14**	1,639E-05	1,05122789	4,78542994	0,07207545
0,14132856	**4ATG16L1**	9,0883E-11	1,10500798	10,0415165	0,14405679
0,10511548	**4ATG2A**	0,89987605	1,00094485	0,04581731	0,00136248
0,04920445	**4ATG2B**	8,7213E-07	0,94714148	6,05941681	-0,07834816
-0,05351479	**4ATG3**	0,18476252	1,01124966	0,73338613	0,01613922
0,08011396	**4ATG4A**	0,00240001	0,9594053	2,61978713	-0,05978768
0,13141779	**4ATG4B**	0,00028634	1,03049936	3,54312189	0,04334361
0,00361117	**4ATG5**	5,5119E-05	1,04125933	4,25869902	0,05832943
-0,12423232	**4ATG7**	8,0546E-05	1,05657878	4,09395609	0,07940034
0,02469527	**4ATG9A**	0,13142454	1,01133937	0,88132352	0,0162672
-0,01901187	**4ATG9B**	0,41175819	1,01181142	0,38535775	0,01694043
0,12450563	**4BECN1**	0,00674337	1,01658396	2,17112323	0,02372937
-0,09984877	**4LAMP1**	0,19656687	1,00655352	0,70648967	0,00942388
0,1117848	**4LAMP2**	0,07506688	1,01678923	1,12455165	0,02402065
0,04057437	**4MAP1LC3A**	0,00053827	0,96503831	3,26900184	-0,05134188
